# Transport of enzymatic activity across liquid-liquid interfaces using dynamic assemblies of magnetic particles via field-modulated interactions

**DOI:** 10.1038/s41467-026-73696-8

**Published:** 2026-05-26

**Authors:** Shilu Zhu, Shuwei Shen, Min Ye, Yang Zhang, Zhiyuan Zheng, Jie Gao, Ru Zhang, Zhongliang Lang, Peng Yao, Mingzhai Sun, Luke P. Lee, Ronald X. Xu

**Affiliations:** 1 School of Biomedical Engineering, Division of Life Sciences and Medicine, University of Science and Technology of China, Hefei, Anhui, 230026 P.R. China; 2 Suzhou Institute for Advanced Research, University of Science and Technology of China, Suzhou, Jiangsu, 215123 P.R. China; 3 Department of Precision Machinery and Instrumentation, School of Engineering Science, University of Science and Technology of China, Hefei, P.R. China; 4 School of Microelectronics, University of Science and Technology of China, Hefei, P.R. China; 5 Renal Division and Division of Engineering in Medicine, Department of Medicine, Brigham and Women’s Hospital, Harvard Medical School, Boston, MA USA; 6 Department of Bioengineering, Department of Electrical Engineering and Computer Science, University of California at Berkeley, Berkeley, CA USA; 7 Institute of Quantum Biophysics, Department of Biophysics, Sungkyunkwan University, Suwon, Korea

**Keywords:** Materials science, Engineering, Physics

## Abstract

Biological systems dynamically grow high-aspect-ratio architectures from a site, enabling traversal of phase boundaries and functional execution. Emulating this growth strategy in synthetic systems could yield functional microsystems for operation across interfaces. However, engineering such bio-inspired growth to proceed out of plane from a substrate in synthetic colloidal assemblies remains challenging, as it requires overcoming gravitational collapse while maintaining structural coherence during extension. Here, we present a field-driven particle system that achieves gravity-resisting growth of high-aspect-ratio structures via frequency-modulated magnetic and hydrodynamic interactions. This growth is enabled by combining static and oscillating magnetic fields, which guide the assembly of magnetic particles into dynamic structures exhibiting a distinct segmented, seaweed-like morphology. These architectures are reconfigurable, stabilizable, programmably actuatable, and capable of penetrating a perfluorohexane–water interface. When functionalized with enzymes, the growing structures act as micro-transporters, delivering catalytic activity across the interface and triggering detectable reactions in both bulk two-phase and microfluidic chip systems. This work establishes a field-driven assembly-to-function approach that integrates structural growth, phase-boundary penetration, and triggered functionality, enabling active microsystems capable of interfacial transport and functional execution.

## Abstract (web summary)

Biological systems form structures across interfaces to perform functions, serving as inspiration for synthetic replicas. Here, the authors demonstrate that dynamic magnetic assemblies can transport enzymatic activity across liquid-liquid interfaces and trigger reactions.

## Introduction

The dynamic growth of high-aspect-ratio structures from a site is a widespread strategy in biology, enabling organisms to traverse phase boundaries and execute critical functions. Pollen tubes extend from germination sites on pollen grains to deliver genetic material through tissues^1^; fungal hyphae elongate from growing tips to secrete enzymes across air gaps onto new substrates^2^; bacterial pili assemble from membrane sites to probe surfaces for adhesion and DNA uptake^3^. Engineering such bio-inspired growth to proceed out of plane from a substrate in synthetic systems would represent a route toward functional microsystems capable of operating across interfaces and within stratified environments.

Field-driven self-assembly of colloidal particles offers a versatile platform to realize such bio-inspired growth. By harnessing external fields (e.g., magnetic^4–7^, electric^8–11^, acoustic^12–14^, optical^15–17^, or chemical^18^), researchers can guide components into organized structures. These approaches are broadly classified into two regimes: static self-assembly and dynamic self-assembly^19,20^. The former is governed by equilibrium thermodynamics and yields stable architectures (e.g., crystalline superlattices) but inherently lacks the dynamic responsiveness required for continuous structural evolution. In contrast, the latter operates under non-equilibrium conditions, dissipating energy to form transient, reconfigurable structures capable of complex behaviors such as collective motion and task execution^20,21^. This paradigm has enabled remarkable advances in microrobotics^22–25^, reconfigurable metamaterials^26^, adaptive chemical networks^27^, and biomedical engineering^28–32^.

Despite this progress, dynamic colloidal systems have remained largely confined to two dimensions, forming structures such as in-plane vortices^22,33–35^, chains^11,23,36^, or ribbon-like assemblies^24,37–39^ on planar substrates or at interfaces. This spatial limitation originates from the inherent absence of out-of-plane interactions and the corresponding assembly pathways. Even when three-dimensional (3D) dynamic assemblies are achieved, they typically form within a bulk phase from suspended particles^40–42^, rather than through programmable growth upward from a substrate, a process which is essential for creating synthetic systems that can bridge and interact across distinct phases. Consequently, for systems starting with sedimented particles, achieving programmable 3D assembly introduces the fundamental challenge of overcoming gravitational collapse. Recent advances using modulated magnetic fields have enabled controlled vertical assembly^43^, yet resulting structures remain compact with limited aspect ratios. Scaling to high-aspect-ratio regimes introduces further complexity: gravitational forces impede continuous vertical elongation, while the increasing aspect ratio itself amplifies mechanical instabilities during dynamic reconfiguration. Together, these factors make the gravity-resisting growth of dynamic, high-aspect-ratio architectures from a substrate remain a challenge in active colloidal matter.

**Fig. 1:**
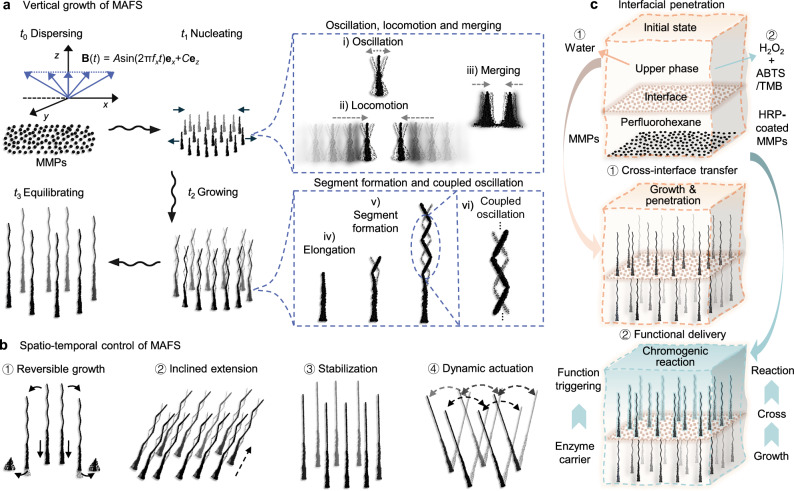
Gravity-resisting assembly, dynamic control, and functional delivery of magnetically assembled filamentary structures (MAFS) via magnetic field modulation. **a** Dynamic self-assembly of magnetic microparticles (MMPs) and vertical growth of MAFS under the oscillating magnetic field, progressing through locomotion, merging, elongation, and segment formation. **b** Programmable structural control via magnetic field modulation, demonstrating four operational modes: reversible growth, inclined extension, post-assembly stabilization, and dynamic actuation. **c** Interfacial penetration across a water/PFH interface (orange arrow), and enzyme-catalyzed chromogenic reaction (green arrow) triggered by HRP-modified MMPs upon contact with H_2_O_2_ and a chromogenic substrate in the upper phase.

In this work, we report a field-driven particle system that achieves gravity-resisting growth of high-aspect-ratio structures and enables functional delivery across liquid–liquid interfaces (Fig. 1). By synergistically combining static and oscillating magnetic fields, the system drives spontaneous growth, ultimately forming magnetically assembled filamentary structures (MAFS) with segmented, seaweed-like morphologies in dynamic equilibrium. These architectures are capable of reversible growth, inclined extension, static stabilization, and programmable actuation via field modulation. Furthermore, they can be steered to penetrate a perfluorohexane (PFH)-water interface, thereby transferring the majority of the constituent microparticles (≈ 1 μm in diameter) from the substrate into the upper phase. By decorating particles with the enzyme horseradish peroxidase (HRP), the growing MAFS act as micro-transporters that deliver catalytic activity into a target phase containing H_2_O_2_ and ABTS, triggering a localized macroscopic color change upon penetration. Extending this capability to a microfluidic chip, we further demonstrate that MAFS can serve as a switch for remote reaction initiation in a chip-integrated format. Our work thus establishes a field-driven assembly-to-function platform that integrates structural growth, phase-boundary penetration, and functional execution, providing a foundation for microsystems capable of operating across stratified environments.

## Results

### Vertical growth of MAFS

**Fig. 2:**
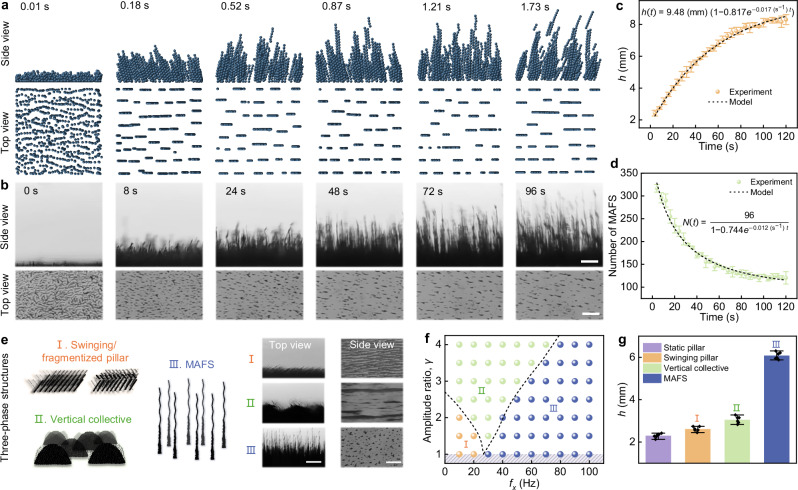
Vertical growth of MAFS. **a** Numerical simulation of MAFS growth dynamics under an oscillating magnetic field (*f*_*x*_ = 90 Hz, *B*_*z*_ = 5 mT, *γ* = 3). **b** Time-lapse imaging of MAFS evolution, captured from side and top views under identical field conditions as in (**a**). Scale bar, 2 mm. **c**,** d** Temporal evolution of structural height (**c**) and count (**d**) during dynamic assembly, quantified from side and top-view imaging in (**b**). Data are presented as mean ± SD (*n* = 3 independent measurements). **e** Schematic illustrations (left) and experimental snapshots (right; side and top views) of three distinct field-induced magnetic assemblies: phase I (low-frequency, pillar-like structures), phase II (medium-frequency, vertical collectives), and phase III (high-frequency, MAFS). Scale bar, 2 mm. **f** Phase diagram categorizing the three magnetic structures as functions of amplitude ratio (*γ*) and oscillation frequencies (*f*_*x*_), at a fixed vertical field strength (*B*_*z*_ = 5 mT). The purple shaded region denotes the parameter regime (*γ* < 1) where the transverse field strength is insufficient to initiate vertical growth. **g** Quantitative comparison of vertical extension across four experimental configurations: (i) a static 5 mT *B*_*z*_ field only, and oscillating fields (with *B*_*z*_ = 5 mT, *γ* = 3) at (ii) *f*_*x*_ = 1 Hz, (iii) *f*_*x*_ = 30 Hz, and (iv) *f*_*x*_ = 90 Hz. Data are presented as mean ± SD (*n* = 6 independent measurements). Source data are provided as a Source data file.

Building upon numerical simulations that revealed vertical growth dynamics under oscillating magnetic fields (Fig. 2a), we developed an electromagnetic actuation (EMA) system (Supplementary Fig. 1) to facilitate the gravity-resisting growth of MAFS through dynamic self-assembly. The oscillating magnetic field (Supplementary Fig. 2) consists of a constant vertical component (*B*_*z*_ = *C*) and a time-varying horizontal component [*B*_*x*_ = *A*sin(2π*f*_*x*_*t*)], where the frequency *f*_*x*_ and the amplitude ratio *γ* = *A/C* serve as key control parameters. Within a specific frequency range, this field configuration induces spontaneous self-assembly of dispersed magnetic particles on the substrate into high-aspect-ratio MAFS through continuous vertical elongation. Real-time observations (Fig. 2b, Supplementary Movie 1) reveal a stepwise assembly process: initial nucleation occurs immediately upon field application via particle aggregation, followed by progressive vertical extension through progressive incorporation of adjacent magnetic units, ultimately reaching dynamic equilibrium (*t* ≈ 96 s). Quantitative temporal analysis demonstrates a nonlinear height progression with decreasing growth rates (Fig. 2c), while the coalescence-driven structural evolution is evidenced by a 62% reduction in the areal density of MAFS (Fig. 2d).

The dual-axis magnetic configuration is critical for achieving programmable vertical growth. Systematic control experiments confirm that isolated field components fail to sustain directional growth: a purely vertical field (*B*_*z*_ only) yields static pillar-like aggregates, while a horizontal field (*B*_*x*_ only) produces chain-like assemblies confined to the substrate plane (Supplementary Fig. 3). Alternative field configurations from prior studies similarly exhibit constrained growth morphologies^43,44^. The emergent vertical growth capability stems from the dynamic interplay between vertical and horizontal magnetic fields, where the oscillation frequency (*f*_*x*_) and amplitude ratio (*γ*) cooperatively govern the structural evolution. Three distinct magnetic structures emerge based on these parameters (Fig. 2e, Supplementary Movie 2): (I) low-frequency swinging or fractured pillars, (II) medium-frequency vertical collectives, and (III) high-frequency MAFS.

Figure 2f presents the phase diagram of magnetic structures generated by the tailored magnetic fields with varying frequency and amplitude ratio combinations. In phase I (low-frequency regime), magnetic pillars exhibit field-synchronized oscillations with swing amplitudes modulated by the *γ* ratio. During oscillations, these pillars achieve uniform heights when aligned with the vertical magnetic field, whereas their heights become angle-dependent when tilted to specific orientations (Supplementary Fig. 4). As the frequency increases, the resulting higher angular velocity strengthens hydrodynamic damping effects over magnetic dipole interactions^37^, causing the pillars to fragment into disordered, large-scale pillar-like assemblies (Supplementary Fig. 5, Supplementary Movie 3). Phase II (intermediate frequencies) exhibits more pronounced fragmentation due to competing hydrodynamic, magnetic, and gravitational torques, which drive the reorganization of fragmented pillars into vertically oscillating colloidal collectives (Fig. 2e, Supplementary Fig. 6) resembling those observed in dual-axis oscillating fields^43^. These collectives achieve greater vertical extensions than phase I pillars at equivalent *γ* conditions. Near the phase II-III transition boundary, a transient growth phenomenon occurs: adjacent pillars fuse temporarily with the primary structure, then abruptly detach, ultimately leading to a cessation of structural evolution (Supplementary Fig. 7, Supplementary Movie 4). Beyond the start frequency *f*_s_, at which this transient behavior ceases and stable vertical growth initiates, field‑induced magnetic interactions drive spontaneous self‑assembly and sustain continued growth. Additionally, the phase diagram reveals a defined non-growing regime (*γ* < 1), marked by a purple shaded area, which delineates the parameter space where the transverse field strength is insufficient to initiate any sustained vertical growth.

Analysis of the phase diagram under a reduced vertical field (*B*_*z*_ = 3 mT) reveals a systematic leftward shift in regime boundaries (Supplementary Fig. 8), facilitating MAFS formation at lower driving frequencies compared to the *B*_*z*_ = 5 mT condition. However, the resultant structures exhibit diminished vertical extension due to weaker magnetic interaction. Below this value (*B*_*z*_ < 3 mT), vertical growth is suppressed as the magnetic energy becomes insufficient to counteract gravitational sedimentation. Additionally, quantitative comparison across four experimental configurations demonstrates the structural elongation of MAFS relative to other morphologies: (i) a static 5 mT *B*_*z*_ field alone, and oscillating fields (*B*_*z*_ = 5 mT, *γ* = 3) at (ii) *f*_*x*_ = 1 Hz, (iii) *f*_*x*_ = 30 Hz, and (iv) *f*_*x*_ = 90 Hz. MAFS achieve a vertical extension 2.7 times that of static pillars, 2.3 times the maximum height of swinging pillars, and twice the elongation of vertically oscillating collectives (Fig. 2g). This performance establishes MAFS as a distinct non-equilibrium morphology under optimized field parameters, demonstrating significant vertical extension tunability via dynamic field modulation.

### Controlled growth of MAFS

**Fig. 3:**
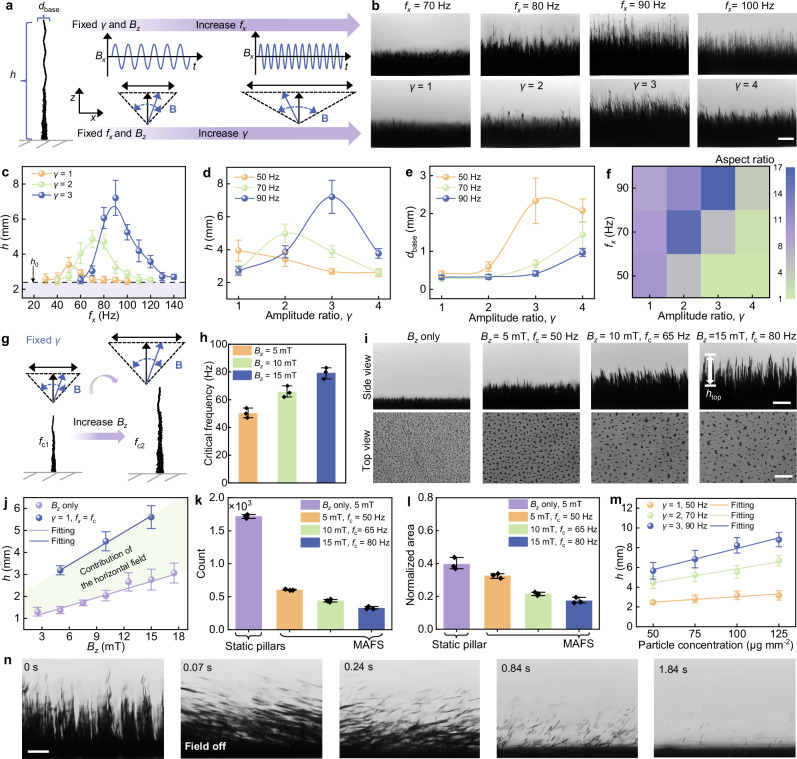
Controlled vertical growth of MAFS. **a** Schematic of parametric control via magnetic field frequency (*f*_*x*_) and amplitude ratio (*γ*). **b** Representative images showing dynamic equilibrium states of MAFS under varying frequencies (*B*_*z*_ = 5 mT, *γ* = 3) and amplitude ratios (*B*_*z*_ = 5 mT, *f*_*x*_ = 90 Hz). Scale bar, 2 mm. **c** Frequency-dependent morphological evolution of MAFS under oscillating magnetic fields. **d** Height of MAFS as functions of amplitude ratio *γ*. Data in (**c**) and (**d**) are presented as mean ± SD (*n* = 3 independent measurements). **e** Base distance (*d*_base_) of MAFS as functions of amplitude ratio *γ*. Data are presented as mean ± SD (*n* = 5 independent experiments). **f** Aspect ratio (*h*/*d*_base_) of MAFS as a function of amplitude ratio *γ*. **g** Schematic of vertical magnetic field strength (*B*_*z*_) modulation. **h** Critical frequencies versus vertical magnetic field strength at amplitude ratio *γ* = 1. Data are presented as mean ± SD (*n* = 3 independent measurements). **i** Side and top views of static pillars and MAFS at varying *B*_*z*_ intensities. Scale bar, 2 mm. **j** Height comparison between structures formed under static fields and oscillating fields (*γ* = 1, *f*_*x*_ = *f*_c_) across varying *B*_*z*_ values, with blue shading highlighting oscillation-enhanced growth. **k** Inverse correlation between *B*_*z*_ intensity and structural density (total count of magnetic pillars and MAFS derived from **i**). **l** Total area occupied by MAFS (top view in **i**), normalized to the total field of view. **m** Particle concentration-dependent heights of MAFS for tested *γ* and *f*_*x*_ values. Data in (**j**–**m**) are presented as mean ± SD (*n* = 3 independent measurements). **n** Time-lapse of structural collapse post-field removal, revealing the critical role of gravity once the stabilizing magnetic field is removed. Scale bar, 2 mm. Source data are provided as a Source data file.

To enable programmable vertical growth control of MAFS, we conducted controlled experiments to separately analyze the effects of magnetic field parameters and non-magnetic environmental factors. Our investigation focused on three key magnetic field parameters: oscillation frequency (*f*_*x*_), amplitude ratio (*γ*), and vertical magnetic field strength (*B*_*z*_). We first examined the effect of independent modulation of the magnetic-field frequency and amplitude ratio on the vertical growth of MAFS (Fig. 3a). The experimental results revealed dual critical behavior with distinct thresholds in frequency (*f*_c_) and amplitude ratio (*γ*_c_). Specifically, MAFS height exhibited non-monotonic dependencies on both *f*_*x*_ and *γ*, peaking at intermediate values before declining (Fig. 3b, Supplementary Fig. 9). For *γ* values of 1, 2, and 3, vertical growth initiated at distinct start frequencies (*f*_s_ = 30, 40, and 60 Hz, respectively), reaching maximum heights of 3.39, 4.87, and 7.21 mm at their corresponding critical frequencies (*f*_c_ = 50, 70, and 90 Hz; Fig. 3c). This frequency-dependent response demonstrates that higher *γ* values promote MAFS extension, particularly in high-frequency regimes.

We further investigated the influence of *γ* on the vertical growth of MAFS under fixed driving frequencies. At *f*_*x*_ = 50 Hz, increasing *γ* from 1 to 4 reduced MAFS height from 3.95 to 2.59 mm, reflecting phase-specific initiation thresholds. Notably, for *γ* ≥ 3, magnetic structures stabilized in phase II states, exhibiting a stagnant baseline height (*h*_0_ ≈ 2 mm; Fig. 3d, Supplementary Fig. 9). Conversely, at *f*_*x*_ = 70 and 90 Hz, MAFS attained peak morphological development at their respective critical *γ* values (*γ* = 2 and 3). The lateral dimensions followed similar trends, with the base distance (*d*_base_) generally increasing with *γ* (Fig. 3e) except at *γ* = 4 and *f*_*x*_ = 50 Hz, where lateral edge aggregation dominated over vertical extension (Supplementary Fig. 9). MAFS exhibited height-proportional aspect ratios (*h*/*d*_base_) extending beyond 16 in taller configurations (Fig. 3f), demonstrating the potential of our bottom-up assembly approach to controllably achieve high-aspect-ratio dynamic architectures.

The vertical magnetic field strength (*B*_*z*_) critically modulates MAFS growth dynamics, as schematically shown in Fig. 3g. Controlled evaluations at three field intensities (5, 10, and 15 mT) with their respective critical frequencies (50, 65, and 80 Hz; Fig. 3h) revealed a monotonic increase in MAFS height (Fig. 3i). Side-view imaging quantitatively compared structural differences across conditions through the *h*_top_/*h* ratio, which increased from 0.25 to 0.67 (Supplementary Fig. 10). This morphological transition proceeds via magnetic dipole-mediated coalescence, where smaller assemblies merge into larger vertical structures. A comparative analysis between static pillars and oscillating field-generated MAFS under identical *B*_*z*_ conditions yielded two key effects: (i) a sustained height increase of 1.82 to 2.84 mm under oscillating fields relative to static conditions (green zone in Fig. 3j), and (ii) progressive structural consolidation, evidenced by a decreasing areal density of MAFS and reduced occupied area (Fig. 3k, l and Supplementary Fig. 11). These results not only confirm that the vertical magnetic-field strength (*B*_*z*_) regulates structural morphology, but also show that the addition of an oscillating field enables a considerably larger vertical extension than the static field alone.

To establish a generalized scaling relationship for this dynamic assembly, we systematically extended the parametric study to include the vertical field strength *B*_*z*_ (3, 4, and 5 mT) across a range of amplitude ratios *γ* (Supplementary Fig. 12a). The extracted start (*f*_s_) and critical (*f*_c_) frequencies for each condition (*B*_*z*_, *γ*) are mapped in Supplementary Fig. 12b, illustrating their systematic dependence on the control parameters. Normalizing the driving frequency *f*_*x*_ by its corresponding *f*_c_ reveals a clear scaling behavior: the operational window for MAFS growth collapses to the range the normalized range of 0.4 < *f*_*x*_/*f*_c_ < 2.0 (Supplementary Fig. 12c). This generalized scaling law links the extent of the operational frequency range to the resulting growth height across different field conditions, providing a universal criterion for this non-equilibrium assembly.

Beyond magnetic-field effects, we systematically examined three non-magnetic parameters governing vertical MAFS growth: interface-substrate separation distance (affecting meniscus curvature), particle concentration, and gravitational interactions. Initial experiments began with the air-water interface positioned well above the substrate, enabling unconstrained MAFS development near the fluid boundary. Progressive lowering of the interface induced concave meniscus formation (purple line in Supplementary Fig. 13a), with MAFS maintaining conformal contact while vertical growth ceased above the water surface. Under these conditions, structural height became exclusively governed by the interface-substrate separation distance. Further reduction of the interface led to extreme confinement, generating optically dense configurations that completely blocked side-view illumination (Supplementary Fig. 13a, b). This light-blocking phenomenon suggests potential utility in dynamically tunable microfluidic optics^43^. MAFS also exhibited environmental adaptability by successfully bridging immiscible aqueous phase boundaries (Supplementary Fig. 13c), demonstrating its operational robustness in heterogeneous fluidic environments.

Our analysis identified particle concentration as a key governing factor in growth dynamics, exhibiting near-linear correlations with maximum attainable heights across three distinct parameter conditions: (i) *γ* = 1, *f*_*x*_ = 50 Hz; (ii) *γ* = 2, *f*_*x*_ = 70 Hz; and (iii) *γ* = 3, *f*_*x*_ = 90 Hz (Fig. 3m). Growth capability displayed marked variability, achieving the peak height increment under high-frequency, large-amplitude conditions (*γ* = 3, *f*_*x*_ = 90 Hz), where intensified magnetic dipolar interactions promoted enhanced vertical particle assembly. This pronounced frequency-amplitude synergy conclusively establishes that optimized field parameters can substantially amplify the sensitivity of MAFS to particle concentration gradients. The dominant influence of gravity was further evidenced by rapid structural collapse upon magnetic field termination (Fig. 3n and Supplementary Movie 5). Complete structural disintegration occurred within two seconds, with constituent particles and intermediate assemblies undergoing gravitationally driven sedimentation. This instantaneous collapse highlights the critical role of gravity in opposing magnetic interactions during vertical assembly. Additionally, systematic experiments with magnetic nanoparticles (100, 300, and 500 nm) revealed size-dependent self-assembly behavior. Larger particles (500 nm) sedimented rapidly due to gravity, while smaller particles (100–300 nm) remained suspended (Supplementary Fig. 14a). This directly affected vertical growth, with 500 nm particles assembling more quickly and reaching greater heights than the smaller particles (Supplementary Fig. 14b, d, e). In addition, the smaller particles remained suspended under magnetic excitation, as the induced fluid disturbances counteracted sedimentation (Supplementary Fig. 14c). Consequently, sub-500 nm nanoparticles showed limited suitability for sustained dynamic high-aspect-ratio growth in the MAFS. Collectively, these observations provide fundamental mechanistic insights into the competing interactions dictating the non-equilibrium self-assembly of MAFS, revealing a delicate interplay between magnetic dipole interactions and gravitational potential in governing structural stability.

### Growth mechanisms and segmented morphology

The vertical growth of MAFS emerges from a dual-field strategy that exploits the frequency-dependent competition between time-averaged magnetic and hydrodynamic interactions to overcome gravitational sedimentation and shear-induced fragmentation. This non-equilibrium self-assembly is governed by this interplay, tuned by the combined static (*B*_*z*_) and oscillating (*B*_*x*_) fields. The system exhibits a fundamental frequency-dependent transition: at low frequencies, magnetic units closely follow the field, generating large-amplitude motion that excites strong, long-range hydrodynamic interactions, leading to lateral instabilities and tilt. Above a frequency threshold, the rapid oscillation averages out the transverse magnetic interaction. This shift suppresses hydrodynamic interaction and makes time-averaged magnetic interactions dominant, which reinforce alignment and attraction along the static *B*_*z*_ axis. The resulting balance provides (i) dynamic stabilization (suppressed hydrodynamic interaction reduces lateral fluctuations) and (ii) directional guidance (enhanced time-averaged magnetic interaction promotes vertical dipole–dipole cohesion), enabling sustained vertical growth.

**Fig. 4:**
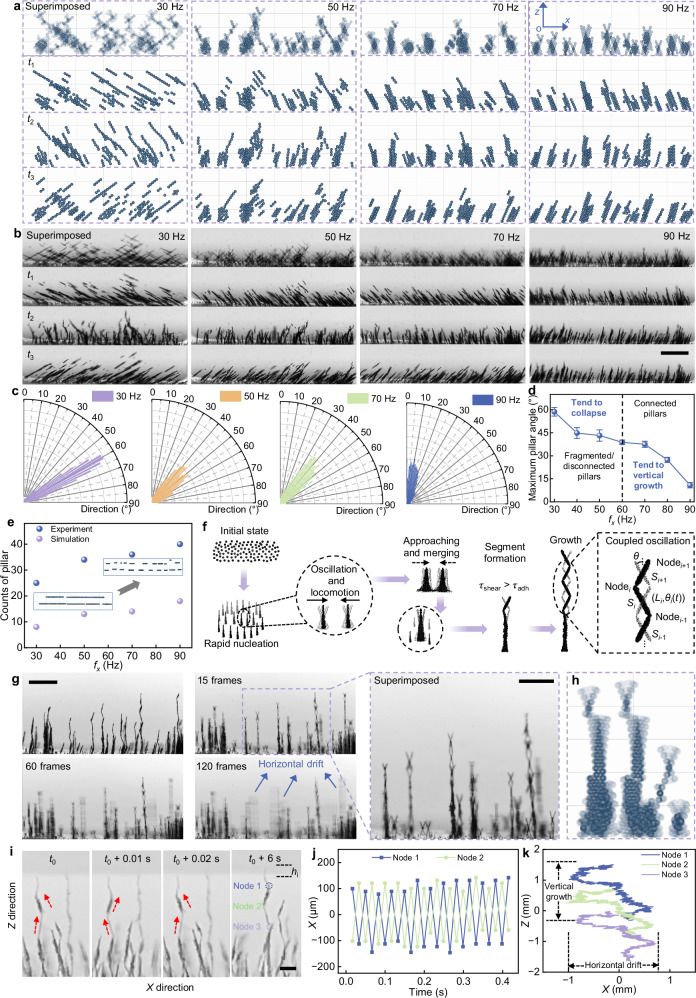
Frequency-modulated growth mechanism and segmented morphology. **a**,** b** Simulated (**a**) and experimental (**b**) particle assemblies at different frequencies (30, 50, 70, and 90 Hz), showing high consistency between theory and experiment. Scale bar, 2 mm. **c** Maximum tilt angle of magnetic pillars relative to the vertical axis under different frequencies (30, 50, 70, and 90 Hz). **d** Inverse correlation between pillar angle and frequency, with low frequencies (purple region) favoring horizontal expansion and high frequencies (green region) promoting vertical alignment. Dashed line at 60 Hz marks the transition between regimes. Data are presented as mean ± SD (*n* = 3 independent measurements). **e** Frequency-dependent pillar count. Increasing frequency shifts morphology from fewer, larger pillars to more, smaller pillars. **f** Schematic diagram illustrating the growth mechanism of MAFS, including five key developmental phases: rapid nucleation, oscillation while locomotion, approaching and merging, segment formation, and sustained vertical growth. **g** Snapshots and superimposed images with varying frame counts (15, 60, and 120 frames; 60 fps) demonstrate the transient segmented structure of MAFS, the X-shaped vertical stacking pattern in dynamic visualization, and the resulting lateral drift. Scale bar, 2 mm. **h** Simulated result of the X-shaped vertical stacking of MAFS, showing time-resolved pattern formation through simulated frame overlays. **i** Time-lapse microscopy sequence capturing segmented MAFS development during vertical growth, highlighting characteristic high-frequency oscillatory behavior. (*h*_i_ indicates the increased height within 6 s). Scale bar, 500 μm. **j** Phase desynchronization between two adjacent nodes (nodes 1 and 2 in **i**). **k** Trajectory plots (*xz*-plane) for three nodal junctions connecting four segments of MAFS (marked in **i**). Source data are provided as a Source data file.

To elucidate this mechanism, we conducted a detailed micro-scale analysis. Combined simulations and experiments across frequencies (30–90 Hz) show high consistency (Fig. 4a, b, Supplementary Movie 6). At lower frequencies (30 and 50 Hz), hydrodynamic interaction-dominated dynamics cause magnetic pillars to undergo cyclic fragmentation and reorganization, forming tilted, X-shaped structures that appeared as staggered stacks in superimposed images (Fig. 4c). Quantitative analysis confirms an inverse relationship between frequency and tilt angle (Fig. 4d): low frequencies favor horizontally expanded assemblies (purple region), while high frequencies (70 and 90 Hz), where time-averaged magnetic interactions dominate, promote vertical elongation (green region). Furthermore, high-frequency conditions generate more, smaller assemblies (Fig. 4e), an outcome that arises from the competition between the driving and resisting torques^45^. This configuration promotes the collision-induced merging key to high-aspect-ratio MAFS growth.

Based on the above analyses, we present a schematic of the dynamic assembly process for the MAFS (Fig. 4f). In the high-frequency regime (*f*_*x*_ > *f*_s_), the transverse magnetic moment component exhibits a decaying response, which is characterized by a frequency-dependent phase lag relative to the driving field (Supplementary Fig. 15, Supplementary Note 1). This dynamic response underpins the time-averaged magnetic interaction-dominated state, suppressing large-scale oscillations and governing the nucleation of transient structures (Fig. 4f). This process is further corroborated by our simulations, which capture the vertical growth dynamics of the assembling particles (Supplementary Fig. 16). Under high-frequency excitation, the particle-assembled primary structures undergo sustained low-amplitude vibrations, where their inherent structural asymmetries give rise to asymmetric flow fields through the fluid-structure interaction near the substrate^46,47^. The resulting flow asymmetry produces a net lateral driving force that propels the structures into directed translational motion (Fig. 4f, top view in Supplementary Fig. 17), comparable to the wobbling of nanoeels induced by structural oscillation^48^. This autonomous mobility facilitates collision-induced coalescence between neighboring architectures (Fig. 4f, Supplementary Figs. 17 and 18). Through successive collision and merging events, the system achieves progressive vertical elongation of the structural units, ultimately leading to the formation of dynamically stabilized MAFS.

Guided by these mechanistic insights, we developed a frequency-modulated vertical growth model, which describes how the frequency-dependent collision-coalescence governs the distinct growth dynamics (e.g., growth velocity and final height) of MAFS (Supplementary Note 2). The time-lapse trajectory overlays visualize the distinct microscopic collision-fusion activity patterns at different frequencies (Supplementary Fig. 19). Crucially, the net decrease in areal density from initial to equilibrium state, which peaks at the critical frequency (Supplementary Fig. 20), directly quantifies the resultant trend of structural consolidation across frequencies. By integrating this model with the experimental characterization of vertical growth below and above the critical frequency (Supplementary Fig. 21a–e), we established the frequency-dependent exponential scaling laws governing the assembly (Supplementary Fig. 21f). The results reveal that dynamic equilibration accelerates at subcritical frequencies, corresponding to reduced growth capability, whereas frequencies exceeding the critical value sustain stable equilibration timescales (Supplementary Fig. 22a–c). Moreover, the average vertical growth velocity follows an inverse frequency dependence, with a distinct growth-phase transition emerging at the critical frequency (Supplementary Fig. 22d–f).

Notably, the vertical extension of MAFS exhibits a unique segmented morphology, where the superimposed images clearly reveal a series of vertically stacked X-shaped units (Fig. 4g, h, Supplementary Fig. 23, and Supplementary Movie 6), distinct from continuous pillar-like architectures. As the structure elongates vertically, high-frequency excitation induces progressive nodal formation along its longitudinal axis, driven by a dynamic mismatch between relatively stabilized basal anchoring and inertia-dominated apical oscillations. This behavior resembles the beating motion of magnetically driven artificial microswimmers^49^ or elastic structures^50^, where internal stress propagation generates bending waves toward the tip. During elongation, its natural frequency decreases until it matches the driving frequency, triggering resonance. This excites higher-order vibration modes that concentrate shear stress (${\tau }_{{{{\rm{s}}}}{{{\rm{h}}}}{{{\rm{ear}}}}}$) at nodal points, where ${\tau }_{{{{\rm{s}}}}{{{\rm{h}}}}{{{\rm{ear}}}}}$ exceeds the magnetic adhesion strength (${\tau }_{{{{\rm{adh}}}}}$), resulting in localized shear banding and subsequent segmentation into shorter sections^51^. These segments subsequently stabilize by detuning from resonance (Supplementary Note 3), which reveals a general dynamic adaptation strategy: the growing structure self-organizes into discrete units to avoid destructive resonance, thereby maintaining mechanical stability under persistent external actuation. Throughout this evolution, each stabilized segment acts as an independent magnetic domain, exhibiting local high-frequency oscillations phase-locked to the external field while displaying inter-segment phase desynchronization due to mechanical decoupling (Fig. 4i–k). Despite local decoupling, long-range magnetic interactions maintain global coupling among segments (Supplementary Note 4). This results in a design principle of localized mechanical flexibility combined with global magnetic coherence, enabling the architecture to withstand localized fluid disturbances while maintaining its overall structural integrity.

### Reversible growth and inclined extension

**Fig. 5:**
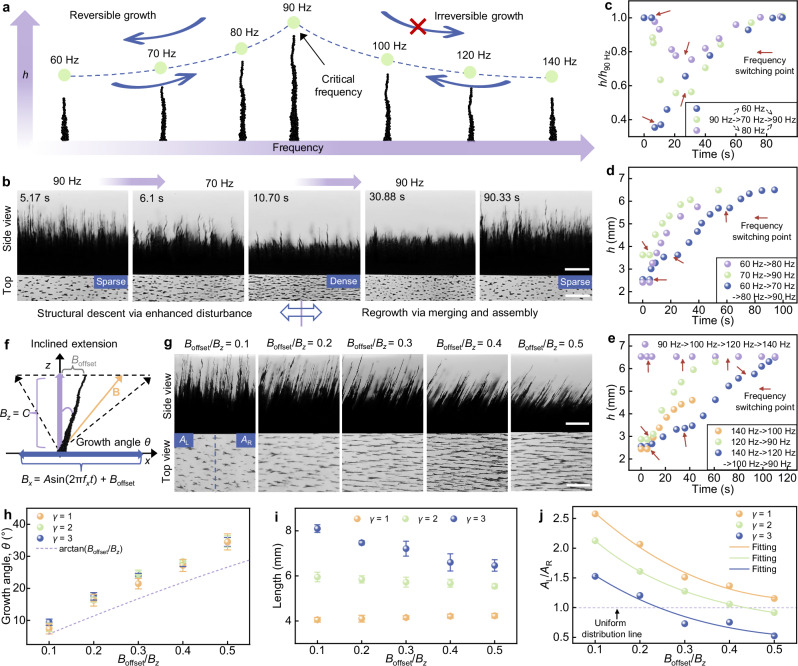
Field-programmable reversible growth and inclined extension. **a** Schematic of the dynamic growth reversibility control strategy. **b** Time-resolved cyclic reconfiguration under frequency switching (periodic 90 Hz $\leftrightarrow$ 70 Hz transitions). Scale bar, 2 mm. **c** Growth transition dynamics during subcritical frequency switching. **d** Dynamic response of MAFS under low-to-high frequency switching in the subcritical range (≤ 90 Hz). **e** Bidirectional frequency-switching dynamics in MAFS across the supercritical regime (≥ 90 Hz). **f** Magnetic field configuration for offset-modulated inclined extension. **g** Inclined growth characteristics versus *B*_offset_/*B*_*z*_ ratios (*γ* = 3). *A*_L_ and *A*_R_ indicate the left and right regions occupied by MAFS in top-view. Scale bar, 2 mm. **h** Experimental versus predicted angular relationship (*θ* = arctan(*B*_offset_/*B*_*z*_)) across varying *B*_offset_/*B*_*z*_ ratios. **i** Structural length variation with tilt angle. Data in (**h**) and (**i**) are presented as mean ± SD (*n* = 4 independent measurements). **j** Spatial distribution patterns as a function of inclination angle. Dashed line marks equilibrium uniformity threshold. Source data are provided as a Source data file.

The development of systems with dynamically programmable architectures demands precise control over bidirectional morphological transitions. Here, we demonstrate the reversible growth behavior of MAFS through frequency-modulated magnetic field cycling (*γ* = 3, *B*_*z*_ = 5 mT), enabling on-demand structural reconfiguration. By operating at the critical frequency (*f*_c_ = 90 Hz) for maximum growth, we achieved reversible height modulation through controlled switching between *f*_c_ and subcritical (60–80 Hz) or supercritical (100–140 Hz) frequencies (Fig. 5a). Time-resolved analysis (Fig. 5b, Supplementary Movie 7) revealed distinct frequency-dependent collapse kinetics: decreasing the frequency from 90 to 70 Hz triggered gradual root disassembly, while returning to *f*_c_ induced rapid regrowth, showing well-controlled reversibility. The collapse rate exhibited a positive correlation with frequency differential ($\Delta$*f*_*x*_), reaching a maximum during the 90-to-60 Hz transition (Fig. 5c). This behavior arises from $\Delta$*f*_*x*_-dependent amplification of lateral perturbations, leading to destabilization of the structure. By systematically applying three frequency modulation protocols, shifting from low to high frequencies within the subcritical to critical regime ( ≤ *f*_c_), we established precise dynamic control over MAFS (Fig. 5d). The $\Delta$*f*_*x*_-dependent transition dynamics demonstrated here provide a generalizable strategy for programming reversible growth in metastable architectures. Supercritical transitions (from 90 to 100–140 Hz) maintained structural height while enhancing vertical alignment (Fig. 5e, Supplementary Fig. 24). This stabilization arises because, at frequencies substantially exceeding the critical threshold, the rapid attenuation of particle magnetic moments causes the system to effectively experience only the time-averaged field component (equivalent to a vertical static field), thereby maintaining pre-assembled structures. This also explains why assembling from dispersed particles at these frequencies can only achieve structures with limited height. However, downward frequency transitions within supercritical range (blue, orange, and green curves in Fig. 5e) exhibited tunable height modulation, revealing operationally critical asymmetric response characteristics. Collectively, these results establish MAFS platform as a model system for reversible growth control in programmable matter, where bidirectional frequency modulation orchestrates both structural reconfiguration and dynamic stabilization.

To enable spatially controlled three-dimensional growth of MAFS, we introduced a magnetic field offset (*B*_offset_) within oscillating field configurations (Fig. 5f). By modulating the *B*_offset_/*B*_*z*_ ratio, we achieved controlled structural inclination while preserving gravitational resistance. This strategy permits both direct self-assembly of dispersed magnetic microparticles into angled architectures and dynamic reorientation of preformed vertical structures. The tilt angle (*θ*) followed a predicted relationship *θ* $=$ arctan(*B*_offset_/*B*_*z*_), establishing a fundamental parameter for spatial programming. Initial experiments with oblique self-assembly (*B*_offset_ = 1 mT, *B*_*z*_ = 5 mT, *γ* = 3, and *f*_*x*_ = 90 Hz) revealed gravity-resistant tilted structures formed via coordinated particle assembly (Supplementary Fig. 25, Supplementary Movie 8). Systematic variation of *B*_offset_ (0.5–2.5 mT) at fixed *B*_*z*_ (5 mT) generated discrete inclination angles across amplitude ratios (*γ* = 1–3) at their respective critical frequencies. As shown in Fig. 5g, h and Supplementary Fig. 26, structural inclination closely matched the predicted angular relationship, displaying a clear *B*_offset_/*B*_*z*_ dependency (0.1–0.5 range) with negligible *γ* influence.

For structures grown under tilted conditions, the length remained stable for *γ* = 1 (4.14 ± 0.1 mm) and *γ* = 2 (5.72 ± 0.2 mm) across the tested range of inclination angles (*B*_offset_/*B*_*z*_ = 0.1–0.5). At *γ* = 3, a measurable reduction in length was observed, decreasing from 8.09 to 6.46 mm (20.1% reduction) with increasing tilt angle (Fig. 5i). Spatial distribution analysis, quantified via top-view hemispheric partitioning (left *A*_L_ vs. right *A*_R_ regions), revealed distinct equilibrium patterns. Vertical growth (*θ* = 0°) yielded uniform distributions (*A*_L_/*A*_R_ ≈ 1), whereas slight rightward inclination (*θ* > 0°) induced leftward accumulation (*A*_L_/*A*_R_ > 1) (Fig. 5j). We identified secondary uniformity regime governing distribution polarity inversion: below critical *θ* thresholds, structures populated the left regions, while surpassing these values prompted rightward dominance. Notably, systems with higher *γ* required smaller *θ* to achieve uniformity, suggesting *γ*-dependent gravitational stability thresholds influenced by structural center-of-mass variations.

Dynamic reorientation capabilities were demonstrated through controlled manipulation of preformed vertical structures (Supplementary Fig. 27, Supplementary Movie 9). Gradual introduction of *B*_offset_ (*B*_offset_/*B*_*z*_ = 0.1) induced smooth leftward tilting (0–8 s) without structural collapse. Subsequent angular modulation (20–67 s) and full orientation reversal (76–99 s) maintained structural integrity, though abrupt right-left transitions resulted in partial collapse (25% failure at 99–114.86 s). This failure likely arose from fluid dynamic stresses exceeding the structural yield threshold during rapid reconfiguration. These results demonstrate precise spatial control over MAFS through magnetic field modulation, enabling programmable tilt angles and dynamic reorientation while maintaining structural stability.

### Post-assembly stabilization and dynamic actuation

**Fig. 6:**
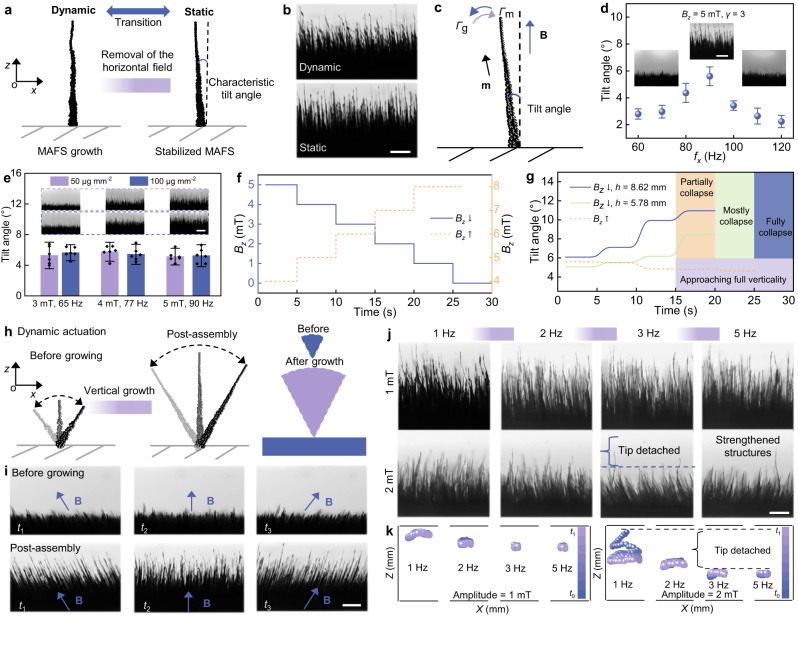
Post-assembly stabilization via magnetic locking and dynamic actuation. **a**, **b** Schematic (**a**) and experimental images (**b**) of the transition between dynamic and static stabilized MAFS. Scale bar, 2 mm. **c** Torque equilibrium analysis of tilted MAFS, illustrating the balance between magnetic torque (${\varGamma }_{{{{\rm{m}}}}}$) and gravitational torque (${\varGamma }_{{{{\rm{g}}}}}$) for static stabilization. **d** Frequency-dependent tilt angle distribution of stabilized structures after terminating the horizontal field at dynamic equilibrium (*B*_*z*_ = 5 mT, *γ* = 3). **e** Parametric analysis of tilt angles under different particle concentrations (50 and 100 μg mm^−2^) and vertical field strengths (*B*_*z*_ = 3, 4, and 5 mT) at critical frequencies (65, 77, and 90 Hz, *γ* = 3). Data in (**d**) and (**e**) are presented as mean ± SD (*n* = 6 independent measurements). **f** *B*_*z*_ modulation protocol for stability control. **g** Structural transitions of stabilized MAFS in response to variations in *B*_*z*_ as defined in (**f**). **h**,** i** Schematic (**h**) and time-lapse images (**i**) of low-height magnetic pillars and MAFS actuation after vertical growth, demonstrating the capability of cross-scale and post-assembly actuation. Scale bar, 2 mm. **j** Morphological response of actuated stabilized MAFS under varying actuation frequencies (1, 2, 3, and 5 Hz) and field amplitudes (1 and 2 mT) at a constant vertical field strength of *B*_*z*_ = 5 mT. Scale bar, 2 mm. **k** Motion trajectory of the MAFS tip under steady-state conditions at different frequencies and driving amplitudes. Source data are provided as a Source data file.

The controlled transition between dynamic and static configurations in magnetic structures could enable on-demand reconfiguration of material properties, with potential applications in soft robotics, adaptive metamaterials, and biomedical devices. To explore this capability, we evaluated the stabilization behavior of MAFS. The removal of horizontal field components while preserving vertical field triggered instantaneous dynamic-to-static configuration transitions (Fig. 6a). These stabilized high-aspect-ratio vertical magnetic architectures cannot be realized in conventional magnetic composite systems or directly assembled via bottom-up approaches under equivalent vertical magnetic field conditions. Notably, upon removal of the horizontal field, the MAFS exhibited a characteristic structural inclination (Fig. 6b and Supplementary Movie 10), as its magnetic moments aligned with the vertical field direction, resulting in zero magnetic torque. Consequently, gravitational effects induce this inclination, which is particularly pronounced in ultra-high slender structures composed of numerous magnetic particles. As the inclination develops, the MAFS experience magnetic torque, eventually reaching equilibrium when magnetic and gravitational torques precisely balance (Fig. 6c). Post-stabilization tilt angles (2°–6°) displayed frequency-dependent maxima at critical frequencies corresponding to specific *B*_*z*_ intensities, with angular reductions observed in both subcritical and supercritical regimes (Fig. 6d). This may be attributed to a comparatively elevated center of mass in critical-frequency configurations relative to other operational conditions. Parametric evaluation across particle concentrations (50 and 100 μg mm^−2^) and *B*_*z*_ strengths (3, 4, and 5 mT) at their respective critical frequencies (65, 77, and 90 Hz) (Supplementary Fig. 28) revealed negligible tilt angle variations (Fig. 6e), implying morphological stability under fixed amplitude ratios (*γ* = 3).

In contrast to magnetic polymer composites^52,53^, the MAFS, composed exclusively of magnetic particles, exhibit distinct structural dynamics. To quantify their stability thresholds, we systematically reduced the vertical magnetic-field intensity (blue line in Fig. 6f). Despite initial tilt variations (5.06° and 6.09°), both structures demonstrated consistent behavior during field reduction (from 5 to 3 mT), maintaining structural integrity while undergoing progressive inclination. Further reduction triggered a cascade of structural failures, progressing from partial collapse to complete disintegration (Fig. 6g, Supplementary Fig. 29). Conversely, incremental field intensity (orange dotted line in Fig. 6f) reduced tilt angles toward vertical alignment through enhanced magnetic torque (orange dotted line in Fig. 6g, Supplementary Fig. 30).

As shown in our experiments (Fig. 2e, f), low-frequency regimes produce field-responsive magnetic pillars with constrained height limitations. To enable large-scale structural actuation, we implemented our vertical dynamic growth strategy and subsequently validated the dynamic actuation capabilities of stabilized MAFS (Fig. 6h, i, Supplementary Movie 10). By superimposing precisely controlled alternating fields onto the static vertical field, we achieved programmable swaying motions, systematically characterizing their actuation profiles through modulation of frequency and amplitude. At low stimulation amplitudes (1 mT), the structures maintained structural integrity across a wide frequency range, displaying two characteristic response regimes: (i) low-frequency synchronized motion and (ii) amplitude-attenuated vibrations at higher frequencies (Fig. 6j, k, Supplementary Fig. 31, and Supplementary Movie 11). Increasing the amplitude to 2 mT induced structural metamorphosis, initiating with tip detachment at 1 Hz and progressing to sustained, highly disturbed flows beyond 3 Hz. This frequency-dependent behavior highlights the potential of MAFS for tunable dynamic applications, such as fluid pumping and mixing^54^. Moreover, the transition from low-aspect-ratio magnetic pillars to high-aspect-ratio MAFS, enabled by the vertical growth process, provides a means for actuation across length scales.

### Interfacial penetration and functional delivery

**Fig. 7:**
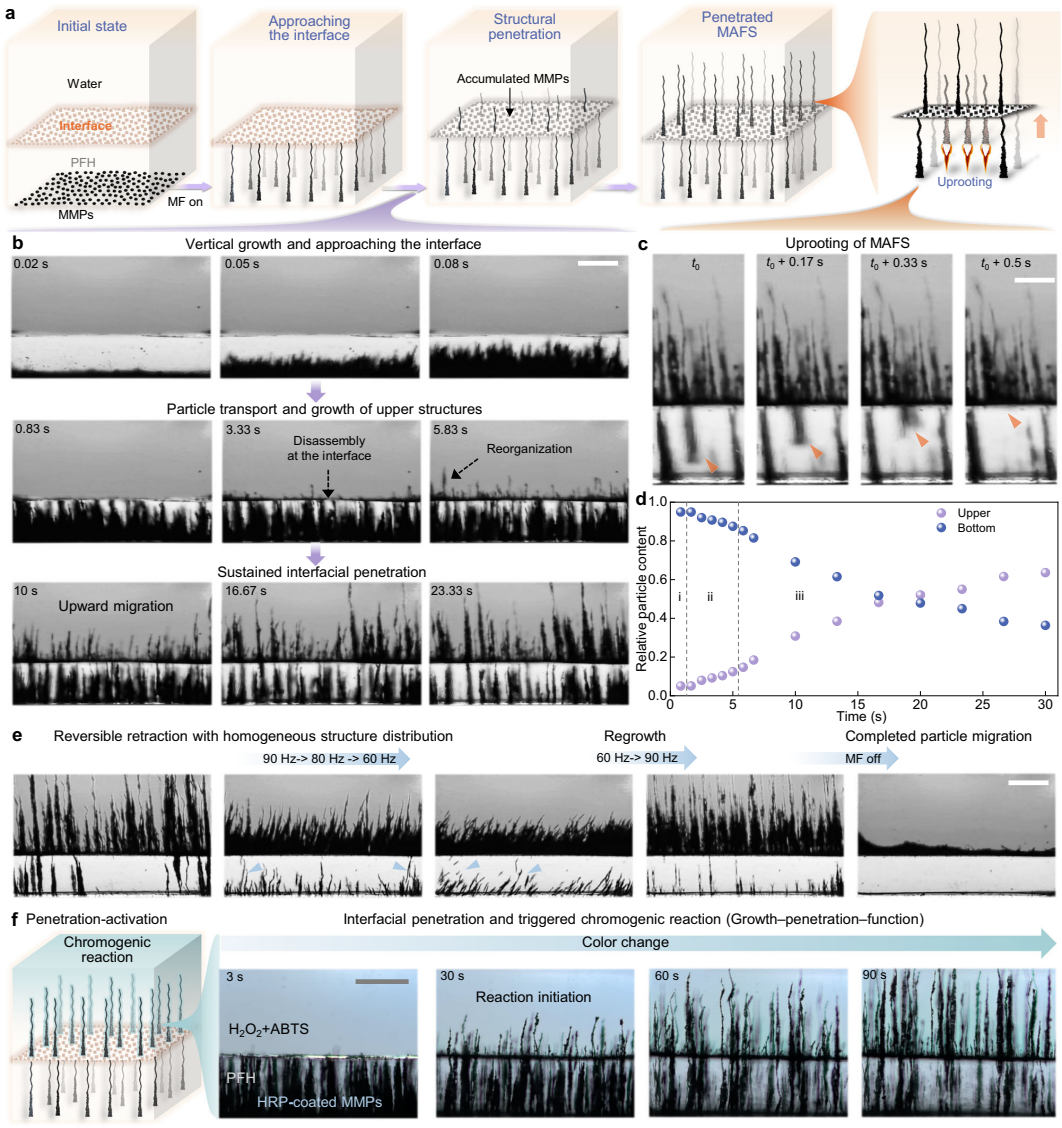
Interfacial penetration and functional delivery of MAFS. **a** Schematic of MAFS crossing the PFH-water interface through dynamic self-assembly, high-aspect-ratio growth, and interfacial energy cascades under the applied magnetic field (MF). **b** Time-lapse images of the crossing process, from vertical growth and approach toward the interface, through penetration attempts (tip breakup with particle dispersion) and subsequent growth of upper structures, to continuous upward transport of structures from the PFH phase into the aqueous phase. Scale bar, 2 mm. **c** Time-lapse images capturing the dynamic process of entire MAFS being uprooted and transferred into the aqueous phase. Scale bar, 1 mm. **d** Quantitative analysis of magnetic particle redistribution over time. Vertical dashed lines separate three dynamic regimes: (i) growth and approach, (ii) penetration attempts with possible fragmentation, and (iii) sustained upward transport, consistent with the time‑lapse images in (**b**). The curves show accumulation in the aqueous phase (increase) and depletion in PFH (decrease). **e** Subsequent migration triggered by modulating the magnetic field frequency (reducing then increasing), demonstrating persistent upward transport of residual magnetic units. Scale bar, 2 mm. **f** Localized chromogenic reaction triggered by the interfacial penetration of MAFS, using HRP-modified particles and an H_2_O_2_/ABTS upper phase. Scale bar, 2 mm. Source data are provided as a Source data file.

To explore the interfacial penetration and functional delivery capabilities of MAFS, inspired by biological systems, we employed an immiscible bilayer system of PFH and an aqueous phase. Initially, we focused on its penetration and transfer capabilities (Fig. 7a). Dried magnetic particles were dispersed in the lower PFH phase, and an aqueous layer was carefully added above to establish a defined PFH/water interface (*t* = 0.02 s in Fig. 7b). Upon applying our vertical-growth magnetic actuation strategy, the magnetic particles rapidly assembled into the roots of MAFS and extended toward the PFH-water interface (*t* = 0.02–0.08 s in Fig. 7b and Supplementary Movie 12). However, as the elongating structures approached the interface, their vertical elongation abruptly halted for approximately 5 seconds (*t* = 0.83–5.83 s in Fig. 7b), demonstrating the resistance posed by interfacial barriers (interfacial tension ≈ 55 mN m^−1^). During this transient arrest, the MAFS tip exhibited high-frequency oscillations to generate localized shear, attempting to overcome interfacial resistance through coordination with upward mechanical thrust. Breakthrough occurred once the force generated by the assembly overcame the interfacial barrier, enabling the hydrophilic particles to escape from PFH confinement and integrate into the aqueous domain. This barrier-breaking event led to two distinct outcomes: (1) discrete particles from structural tips detached from the magnetic confinement and adsorbed onto the interface (disconnected from the sub-interface structural domains), or (2) the entire structure was uprooted and pulled toward the interface (Fig. 7c), followed by rapid structural fragmentation and interfacial spreading as the system minimized free energy. These dual pathways resulted in a predominance of particle spreading and accumulation at the interface during this stage, accompanied by minor localized particle reassembly events that were entirely decoupled from the underlying structural domains (Supplementary Movie 12).

When interfacial particle coverage reached a critical threshold, two concurrent phenomena emerged: (1) intact MAFS from the lower phase were dragged upward without fragmentation, maintaining their structural integrity, and (2) when these rising structures encountered pre-existing MAFS at the interface, they directly merged, forming thicker and more robust architectures. This transition was facilitated by magnetic dipolar interactions, which, operating within an interface whose local properties and energy landscape were progressively altered by accumulating particle^55,56^, enabled coherent upward motion and merging rather than fragmentation. During this process, over half of the magnetic particles from the lower phase were rapidly transferred (*t* = 23 s in Fig. 7b, d), and over time, the vast majority of particles originally dispersed in the PFH phase were transported to the upper aqueous layer, leaving only a residual fraction in the lower phase.

Subsequent reduction of the magnetic field frequency enabled uniform interfacial distribution of the assembled structures. Even under these conditions, residual magnetic units continued migrating upward, driven by a long-range attractive force generated from the high-density interfacial particle accumulation. This self-sustaining magnetic gradient effectively depleted the lower phase, as confirmed by the minimal residual structures observed when vertical assembly was reactivated (Fig. 7e, Supplementary Movie 13). The system thus achieved near-complete particle transfer through this cascading recruitment process.

The interfacial penetration capability of MAFS can be attributed to three synergistic properties emerging from its dynamic self-assembly: sustained actuation from continuous growth, efficient energy focusing via high-aspect-ratio geometry, and self-amplifying interfacial recruitment. First, sustained actuation is provided by the continuous, field-driven growth. Unlike static structures, MAFS dynamically elongate under the magnetic field. Such out-of-equilibrium assembly process can act as integrated actuators^22,57^, whereby continuous growth converts field energy into a sustained mechanical thrust directed against the interface. Concurrently, high-frequency tip oscillations generate localized shear that actively perturbs the interfacial layer, further facilitating penetration. Second, the high-aspect-ratio geometry focuses the actuation energy. This slender form converts magnetic energy predominantly into vertical displacement, concentrating stress at the advancing tip while minimizing lateral dissipation. This directed progress is reminiscent of principles from the hydrodynamics of slender bodies at low Reynolds numbers, where geometric anisotropy favors directional motion^46^. Third, and most distinctively, the system features a self-amplifying, positive-feedback loop at the interface. The initial transfer of particles to the interface alters its local properties. Since adsorbed colloidal particles are known to reduce effective interfacial tension and create energy gradients^55,56^, this primary event lowers the local energy barrier for crossing. Consequently, subsequent transfers are progressively facilitated, driving the observed cascade of near-complete particle recruitment from the lower phase (Supplementary Fig. 32). This synergy establishes a strategy for designing active systems capable of programmed traversal across immiscible interfaces.

To directly couple interfacial penetration with a tangible functional output, we engineered MAFS to act as biocatalytic micro-transporters. Particles were functionalized with HRP, and the upper aqueous phase was replaced with a solution containing H_2_O_2_ and the chromogenic substrate ABTS. Upon field-controlled penetration of the PFH/(H_2_O_2_-ABTS) interface, the HRP-loaded MAFS delivered enzymatic activity into the target phase, triggering an immediate localized macroscopic chromogenic reaction that manifested as a sharp color change precisely co-localized with the penetration site (Fig. 7f, Supplementary Movie 14). This result demonstrates that interfacial penetration is not merely a physical transfer but can be linked to a subsequent chemical function.

### Microfluidic integration for controlled enzymatic reactions

**Fig. 8:**
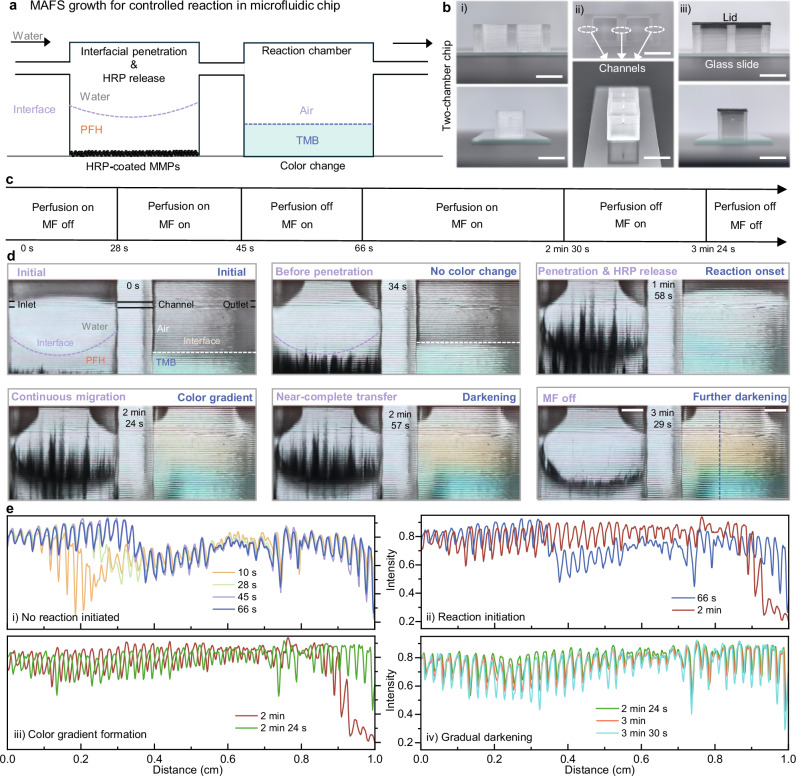
Microfluidic integration of MAFS for controlled enzymatic reactions. **a** Schematic of the two-chamber chip. The left chamber contains the PFH phase with HRP-functionalized magnetic particles and an upper aqueous phase; the right chamber contains the TMB substrate. The chambers are connected by a microchannel. **b** Photograph of the fabricated two-chamber microfluidic chip. Scale bar, 1 cm. **c** Experimental timeline showing the control strategy of perfusion (syringe pump on/off) and magnetic field (MF) application (on/off). **d** Time-lapse images (purple text, left‑chamber state; blue text, right‑chamber state) showing the reaction progression in the right chamber according to the timeline in (**c**). Before or shortly after MAFS penetration, no reaction occurs in the right chamber upon perfusion. Following successful interfacial penetration, a progressive color change emerges in the right chamber, subsequently forming a vertical gradient (from light green to blue to yellow) and intensifying over time, indicating sustained enzyme delivery and reaction. Scale bar, 2 mm. **e** Vertical color intensity profiles along the dashed line (purple) in (**d**) at selected time points, capturing four key stages: no reaction initiated, reaction initiation, gradient formation, and gradual darkening. Source data for Fig. 8 are provided as a Source data file.

To further demonstrate the functional utility of MAFS, we integrated the system into a two-chamber microfluidic chip (Fig. 8a, b, Supplementary Fig. 33). The chip featured a left chamber containing the PFH/aqueous two-phase system with HRP-functionalized particles, a right chamber containing the chromogenic substrate TMB, and a connecting microchannel. This design enabled the investigation of MAFS-mediated enzyme delivery for controlled reactions in a physically separate compartment.

We first conducted a control experiment without applying a magnetic field to prevent MAFS formation or interfacial penetration. Under continuous perfusion at 500 μL min^−1^, the TMB solution in the right chamber gradually lightened due to dilution, with no colorimetric reaction observed (Supplementary Fig. 34a). Quantitative color intensity analysis confirmed this dilution trend in the absence of enzymatic activity (Supplementary Fig. 34b), establishing that no non-specific enzyme transfer occurred. Next, we examined the full functional sequence under magnetic-field guidance, following the timeline illustrated in Fig. 8c. During the initial perfusion period (0–28 s, perfusion on, magnetic field off), MAFS remained inactive, and no color change was detected in the right chamber (Fig. 8d and Supplementary Movie 15). Upon activating the magnetic field (28–45 s, perfusion on, field on), MAFS initiated grow and began penetrating the interface. However, even with ongoing perfusion, no reaction was observed in the right chamber at this early stage (Fig. 8d, 0 and 34 s). This lack of response is attributed to the limited quantity of HRP delivered during the initial phase of interfacial crossing; only a small number of particles had crossed into the upper aqueous phase, resulting in enzyme concentrations below the detection threshold upon perfusion.

To allow sufficient enzyme accumulation, we temporarily paused perfusion while maintaining the magnetic field (45–66 s, perfusion off, field on). During this interval, MAFS continued to grow and traverse the interface, progressively releasing HRP into the upper aqueous phase of the left chamber. When perfusion was resumed (66 s to 3 min 24 s, perfusion on, field on), a distinct color change emerged in the right chamber. A light blue color first appeared at the chamber bottom (Fig. 8d, 1 min 58 s), signaling the arrival of enzyme-containing solution. Subsequently, a vertical color gradient developed along the channel, marking the spatial progression of the enzymatic reaction (Fig. 8d, 2 min 24 s). As MAFS continued to transfer into the upper phase and release enzyme, the color intensity in the right chamber progressively deepened (Fig. 8d, 2 min 57 s). The reaction progression was marked by four distinct stages in the vertical color intensity profile: baseline (no color change), reaction initiation (color change at the bottom), color gradient formation (spatial variation), and gradual darkening (increasing product concentration) (Fig. 8e). These results demonstrate that MAFS act as an enzymatic switch: its magnetic-field-triggered interfacial penetration gates the delivery of biochemical cargo, and the accumulated enzyme can be transported through microfluidic flow to initiate and modulate reactions in a remote compartment.

## Discussion

This work introduces a particle system that achieves gravity-resisting growth of high-aspect-ratio structures and enables functional delivery across immiscible liquid–liquid interfaces. By leveraging frequency-modulated magnetic and hydrodynamic interactions, the system guides magnetic particles into dynamic assemblies with seaweed-like morphologies capable of withstanding gravitational collapse and fluid disturbances. While prior systems have utilized time-averaged interactions from multi-axis^58^, time-dependent fields to generate complex patterns within a bulk fluid or at an interface, they largely remain confined to 2D organization or to 3D aggregates within a single medium under negligible gravitational influence. These systems generally lack the ability for persistent vertical extension from a substrate. In contrast, our system leverages the principle of field-programmed time-averaged interactions to enable the bottom-up, persistent assembly of structures directly from a dense particle layer against the influences of gravity and fluid disturbances. Through magneto-hydrodynamic tuning via oscillating field frequency, these interactions simultaneously promote magnetic particle aggregation for elongation and confer sufficient structural stiffness to resist buckling, enabling the growth of dynamic, high-aspect-ratio, segmented architectures unattainable in suspension-based or interface-confined systems.

Furthermore, our system establishes a programmable, non-equilibrium assembly pathway that differs from classical field-induced structuring phenomena, such as the Rosensweig instability in ferrofluids^59^ or the static-field-driven bottom-up assembly of pillars from sedimented microparticles^44^. Whereas such equilibrium or quasi-equilibrium processes are constrained by static force balances, limiting structures to low aspect ratios, our synergistic use of static and oscillating fields drives continuous, energy-dissipative growth from the substrate. Importantly, this dynamic growth phase is seamlessly integrated with post-assembly functionality. By removing the oscillating field, the resulting structure can be transitioned into a stable, locked configuration (a potential sensor array^60^), and subsequently re-actuated on demand by reapplying a low-frequency oscillation. Thus, within a single experimental setup, the same field modalities (i.e., a static vertical field and a horizontal oscillating field) support three distinct operating regimes through variation of the oscillating field parameters: dynamic growth (high frequency, high amplitude), static stabilization (oscillating field off), and dynamic actuation (low frequency, low amplitude). This unified framework enables a seamless transition from assembly to stabilization to dynamic actuation.

Beyond structural control, the particle system accomplishes field-guided penetration across immiscible liquid–liquid interfaces for functional delivery. In a bulk two-phase system, HRP-functionalized particles enable the growing structures to act as micro-transporters, delivering catalytic activity across the PFH-water interface to trigger a localized macroscopic chromogenic reaction. This capability is further extended to a microfluidic chip, where the assembled structure functions as an enzymatic switch, enabling controlled reaction initiation using TMB as a chromogenic substrate to gate cargo delivery into a separate compartment. These demonstrations collectively embody functional delivery, amplified macroscopic output, and operation in compartmentalized systems, suggesting potential applications in multiphase reaction engineering, microreactor design, and lab-on-a-chip systems. The strategy of field-guided material delivery across an interface to trigger a localized response provides a conceptual framework that could inspire future designs for functional delivery in more complex environments.

In summary, we have developed a particle system that integrates structural assembly, phase-boundary penetration, and functional execution. The platform thereby advances the field of active matter toward the creation of microsystems capable of integrating structural growth with functional execution in multiphase environments.

## Methods

### Materials

Magnetite (Fe₃O₄) particles (average diameter ≈1 μm) were purchased from Nangong Lijia Metal Materials Co., Ltd. (Nangong, China) and characterized for magnetic properties through hysteresis-loop measurements (Supplementary Fig. 35). Prior to experimentation, the particles underwent a purification process consisting of sequential ethanol washes to remove surface contaminants, followed by oven drying at 60 °C.

Polyethylene glycol (PEG; *M*_w_ 8000 g mol^−1^, >99%) and polyvinyl alcohol (PVA; viscosity 5.2–6.0 mPa s, ≈99% hydrolyzed) were purchased from Shanghai Macklin Biochemical Co., Ltd. Perfluorohexane (PFH; *M*_w_ 338.04 g mol^−1^, ≥98%) and horseradish peroxidase (HRP; ≥250 Umg^−1^, RZ ≥ 3.0) were obtained from Shanghai Aladdin Biochemical Technology Co., Ltd. 2,2′-azino-bis(3-ethylbenzothiazoline-6-sulfonic acid) (ABTS; *M*_w_ 548.68 g mol^−1^, ≥98%) and 3,3′,5,5′-tetramethylbenzidine (TMB; *M*_w_ 240.34 g mol^−1^, >99%) chromogenic substrate were also purchased from Shanghai Macklin Biochemical Co., Ltd. Polydimethylsiloxane (PDMS; Sylgard 184) was supplied by Dow Corning (Midland, MI, USA).

### Magnetic actuation system

A custom-designed magnetic actuation system was employed to investigate particle dynamics under controlled field conditions. The system generated programmable oscillating magnetic field sequences through a LabVIEW-controlled interface, enabling precise temporal and spatial modulation of field parameters. The core component consisted of a triaxial Helmholtz coil array (Supplementary Fig. 1j, Supplementary Table 1) capable of producing spatially uniform magnetic fields up to 20 mT along three orthogonal axes. Field generation was achieved through a National Instruments USB-6229 data acquisition (DAQ) card, with output signals amplified by three high-precision OPA549 power modules (Supplementary Fig. 1k).

For magnetic actuation, a precisely weighed mass of purified particles was dispersed in deionized water within a 3.5 cm diameter Petri dish to create a well-defined aqueous suspension system. To achieve uniform particle dispersion, a NdFeB permanent magnet was systematically moved beneath the Petri dish to disaggregate particle clusters and concentrate them at the substrate interface. Subsequently, an oscillating magnetic field was applied along the *z*-axis to further break up any remaining aggregates and promote homogeneous distribution at the substrate interface.

### Immiscible liquid–liquid interface

PFH, a compound with both hydrophobic and oleophobic properties, was selected to form an immiscible liquid–liquid interface with an aqueous solution. Due to its higher density compared to water, PFH settled as the lower phase, while the aqueous solution remained in the upper phase. To enable the cross-interface transfer of MAFS, dried magnetic particles were first introduced into a PFH-filled container (1 cm × 1 cm × 1 cm), followed by the gradual addition of the aqueous solution, resulting in the formation of a well-defined interface.

For the chromogenic reaction experiment, particles were functionalized by incubation in an HRP solution, followed by air-drying. These HRP-modified particles were dispersed in PFH as described, and the upper aqueous phase was replaced with a solution containing H_2_O_2_ and the chromogenic substrate ABTS.

### Fabrication of the two-chamber chip

The two-chamber microfluidic chip was fabricated using standard soft lithography techniques (Supplementary Fig. 34). A master mold with channel features (channel diameter: 0.8 mm) was designed using computer-aided design software and fabricated by 3D printing. To create inlet/outlet channels, stainless steel needles were inserted into the mold prior to PDMS casting. PDMS prepolymer mixed with a curing agent (10:1 ratio) was degassed, poured onto the mold, and cured at 60 °C for 3 h. After curing, the needles were removed to form channels, and the PDMS replica was peeled off from the mold. The PDMS replica and a glass slide were treated with oxygen plasma and bonded together to form an irreversible seal. Finally, a 3D-printed lid was placed onto the PDMS slab to create a relatively enclosed two-chamber configuration for fluidic experiments, with the chromogenic substrate TMB injected into the right chamber.

### Applied magnetic field

The superposed magnetic field **B** applied to the system is described by the vector sum of two orthogonal components:

1 $${{{\bf{B}}}}\left(t\right)={B}_{x}\left(t\right){{{{\bf{e}}}}}_{x}+{B}_{z}{{{{\bf{e}}}}}_{z}=A\sin \left(2{{{\rm{\pi }}}}{f}_{x}t\right){{{{\bf{e}}}}}_{x}+C{{{{\bf{e}}}}}_{z}$$

where *A* and *f*_*x*_ represent the amplitude and frequency of the oscillating field component along the *x*-axis, respectively. *C* denotes the constant field strength in the *z*-direction. **e**_*x*_ and **e**_*z*_ are unit vectors in *x* and *z* directions. The amplitude ratio (*γ* = *A/C*) characterizes the relative strength of the alternating to static field components. The resulting time-dependent orientation of the magnetic field is quantified by the oscillating angle *φ*(*t*) and angular velocity *ω*(*t*):

2 $$\varphi \left(t\right)=\arctan \frac{A\sin (2{{{\rm{\pi }}}}{f}_{x}t)}{C}=\arctan [\gamma \sin \left(2{{{\rm{\pi }}}}{f}_{x}t\right)]$$

3 $$\omega \left(t\right)=\frac{{{{\rm{d}}}}\varphi (t)}{{{{\rm{d}}}}t}=\frac{2{{{\rm{\pi }}}}\gamma {f}_{x}\cos (2{{{\rm{\pi }}}}{f}_{x}t)}{1+{{\gamma }^{2}\sin }^{2}(2{{{\rm{\pi }}}}{f}_{x}t)}$$

For a representative case with *f*_*x*_ = 90 Hz, *C* = 5 mT, and *γ* = 3, the temporal evolution of both the field vector and its derived parameters (Supplementary Fig. 2c, d) demonstrate the dynamic field behavior.

To enable precise orientation control of MAFS, we introduced a horizontal field offset (${B}_{{{{\rm{offset}}}}}$) modifying the *x*-component as:

4 $${B}_{x}\left(t\right)=A\sin \left(2{{{\rm{\pi }}}}{f}_{x}t\right){{{\boldsymbol{+}}}}{B}_{{{{\rm{offset}}}}}$$

This adjustment creates a tunable baseline field component that permits controlled deviation from the primary oscillation axis, facilitating directed growth at predetermined angles.

### Simulation of MAFS growth

To simulate the field-directed assembly of magnetic particles into MAFS, we developed a physical model incorporating multiple interaction mechanisms. The system consists of monodisperse spherical particles with radius *a*, density ${\rho }_{{{{\rm{p}}}}}$, and volume ${V}_{{{{\rm{p}}}}}$, initially uniformly dispersed on a substrate. At the core of the model is the magnetic response of individual particles, where the temporal evolution of the magnetic moment (${{{\bf{m}}}}$) is described by Debye relaxation dynamics^61^:

5 $$\frac{{{{\rm{d}}}}{{{\bf{m}}}}}{{{{\rm{d}}}}t}=-\frac{1}{\tau }\left({{{\bf{m}}}}-{{{{\bf{m}}}}}_{{{{\rm{eq}}}}}\right)$$

with $\tau$ representing the characteristic relaxation time, and the equilibrium magnetic moment given by:

6 $${{{{\bf{m}}}}}_{{{{\rm{eq}}}}}=\frac{{V}_{{{{\rm{p}}}}}{\chi }_{0}{{{\bf{B}}}}}{{\mu }_{0}}$$

where ${\mu }_{0}$ is the vacuum permeability and ${\chi }_{0}$ is the effective magnetic susceptibility. The particles interact through dipole–dipole forces, which play a crucial role in their self-organization under applied magnetic fields. The force on the $i$ th particle due to neighboring magnetic moments is:

7 $${{{{\bf{F}}}}}_{i}^{{{{\rm{m}}}}}=\frac{3{\mu }_{0}}{4{{{\rm{\pi }}}}}{\sum }_{j\ne i}\frac{1}{{{r}_{{ij}}}^{4}}[\left({{{{\bf{m}}}}}_{i}\cdot {\hat{{{{\bf{r}}}}}}_{{ij}}\right){{{{\bf{m}}}}}_{j}+\left({{{{\bf{m}}}}}_{j}\cdot {\hat{{{{\bf{r}}}}}}_{{ij}}\right){{{{\bf{m}}}}}_{i}+\left({{{{\bf{m}}}}}_{i}\cdot {{{{\bf{m}}}}}_{j}\right){\hat{{{{\bf{r}}}}}}_{{ij}}-5\left({{{{\bf{m}}}}}_{i}\cdot {\hat{{{{\bf{r}}}}}}_{{ij}}\right)\left({{{{\bf{m}}}}}_{j}\cdot {\hat{{{{\bf{r}}}}}}_{{ij}}\right){\hat{{{{\bf{r}}}}}}_{{ij}}]$$

where ${r}_{{ij}}=\left|{{{{\bf{r}}}}}_{j}-{{{{\bf{r}}}}}_{i}\right|$ is the interparticle distance and ${\hat{{{{\bf{r}}}}}}_{{ij}}={{{{\bf{r}}}}}_{{ij}}/{r}_{{ij}}$ is the corresponding unit vector.

To ensure physical realism, the model accounts for several additional forces. Gravity and buoyancy effects are included through:

8 $${{{{\bf{F}}}}}_{i}^{{{{\rm{g}}}}}=-\left({\rho }_{{{{\rm{p}}}}}-{\rho }_{{{{\rm{f}}}}}\right){V}_{{{{\rm{p}}}}}g{{{{\bf{e}}}}}_{z}$$

where ${\rho }_{{{{\rm{f}}}}}$ is the fluid density, $g$ is the amplitude of the gravitational acceleration, and ${{{{\bf{e}}}}}_{z}$ is the unit vector in the *z* direction. Additionally, a short-range repulsive force ${{{{\bf{F}}}}}_{i}^{{{{\rm{ev}}}}}$ must be included to prevent particles from overlapping^45,62^:

9 $${{{{\bf{F}}}}}_{i}^{{{{\rm{ev}}}}}=\varsigma \frac{3{\mu }_{0}}{4{{{\rm{\pi }}}}{(2a)}^{4}}{\sum }_{j\ne i}{m}_{i}{m}_{j}\exp [-\zeta \left(\frac{{r}_{{ij}}}{2a}-1\right)]{\hat{{{{\bf{r}}}}}}_{{ij}}$$

where $\varsigma$ and $\zeta$ were set 2 and 10, respectively, so that two particles can mechanically contact and the repulsive force can exactly balance the attractive magnetic dipole force. The particle-wall interaction is modeled through a modified Lennard-Jones potential^63^:

10 $${{{{\bf{F}}}}}_{i}^{{{{\rm{w}}}}}=\frac{24\varepsilon }{\sigma }(2{\left(\frac{\sigma }{{z}_{i}-a}\right)}^{12}-{\left(\frac{\sigma }{{z}_{i}-a}\right)}^{6}){{{{\bf{e}}}}}_{z},$$

where $\varepsilon$ and $\sigma$ are the interaction strength the collision diameter between the wall and the particle, respectively, and ${z}_{i}$ is the distance from the wall to the center of the magnetic particle.

The hydrodynamic drag force follows Stokes’ law:

11 $${{{{\bf{F}}}}}_{i}^{{{{\rm{d}}}}}=-6{{{\rm{\pi }}}}\,\eta \,a\left({{{{\bf{v}}}}}_{i}-{{{{\bf{v}}}}}_{{{{\rm{f}}}}}\right)$$

where $\eta$ is dynamic viscosity of the fluid, and ${{{{\bf{v}}}}}_{i}$ and ${{{{\bf{v}}}}}_{{{{\rm{f}}}}}$ are the velocity of the particle and the velocity of the background fluid induced by other particle movements, respectively. The hydrodynamic coupling between particles is governed by the Rotne-Prager-Yamakawa (RPY) tensor, which describes the fluid velocity field at particle-*i*’s position generated by neighboring particles:

12 $${{{{\bf{v}}}}}_{{{{\rm{f}}}}}\left({{{{\bf{r}}}}}_{i}\right)={\sum }_{j\ne i}{{{{\bf{T}}}}}_{{ij}}\bullet {{{{\bf{F}}}}}_{j}^{{{{\rm{d}}}}}$$

where ${{{{\bf{T}}}}}_{{ij}}$ represents the complete hydrodynamic interaction tensor that accounts for both free-space effects and boundary corrections. This tensor can be decomposed into four distinct components^64,65^:

13 $${{{{\bf{T}}}}}_{{ij}}={{{{\bf{T}}}}}_{{ij}}^{{{{\rm{free}}}}}-{{{{\bf{T}}}}}_{{ij}}^{{{{\rm{im}}}}}+{{{{\bf{D}}}}}_{{ij}}{{{\boldsymbol{+}}}}{{{{\bf{S}}}}}_{{ij}}$$

where ${{{{\bf{T}}}}}_{{ij}}^{{{{\rm{free}}}}}=\frac{1}{8{{{\rm{\pi }}}}\eta {r}_{{ij}}}\left(({{{\bf{I}}}}+{\hat{{{{\bf{r}}}}}}_{{ij}}{{\hat{{{{\bf{r}}}}}}_{{ij}}}^{{{{\rm{T}}}}})+\frac{2{a}^{2}}{3{r}^{2}}({{{\bf{I}}}}-3\hat{{{{\bf{r}}}}}{\hat{{{{\bf{r}}}}}}^{{{{\rm{T}}}}})\right)$ is the free-space RPY tensor and ${{{\bf{I}}}}$ is the unit tensor. The image Stokeslet ${{{{\bf{T}}}}}_{{ij}}^{{{{\rm{im}}}}}={{{\bf{T}}}}({{{{\bf{r}}}}}_{i}-{{{{\bf{r}}}}}_{j}^{{{{\rm{im}}}}})$, Stokes doublet (${{{{\bf{D}}}}}_{{ij}}$) and source doublet (${{{{\bf{S}}}}}_{{ij}}$) ensure the no-slip boundary condition at ($z=0$), where ${{{{\bf{r}}}}}_{j}^{{{{\rm{im}}}}}=({x}_{j},{y}_{j},-{z}_{j})$. The complete equation of motion for each particle integrates all force contributions:

14 $${{{{\bf{v}}}}}_{i}={{{{\bf{v}}}}}_{{{{\rm{f}}}}}+\frac{1}{6{{{\rm{\pi }}}}\,\eta \,a}\left({{{{\bf{F}}}}}_{i}^{{{{\rm{m}}}}}+{{{{\bf{F}}}}}_{i}^{{{{\rm{g}}}}}+{{{{\bf{F}}}}}_{i}^{{{{\rm{ev}}}}}+{{{{\bf{F}}}}}_{i}^{{{{\rm{w}}}}}\right)$$

This model establishes a computational framework to investigate the vertical growth dynamics of MAFS under programmable magnetic fields. The simulations were performed in MATLAB with a custom script provided as Supplementary Data 2.

### Video acquisition and data analysis

To enable comprehensive characterization of the dynamic self-assembly process, we implemented a dual-camera imaging system capturing synchronized side and top views of the magnetic particle assembly. This dual-view imaging strategy enabled systematic analysis of particle behavior and interactions under applied magnetic fields. The recorded images and videos were processed and analyzed using ImageJ software (version 2.14.0/1.54f) to perform quantitative and qualitative evaluations of the self-assembly processes.

## Supplementary information

Supplementary Information

Description of Additional Supplementary Files

Supplementary Movies

Supplementary Data 1 (Raw data)

Supplementary Data 2 (code)

Reporting-summary

Transparent Peer Review file

## Source data

Source Data

## Data Availability

The data that support the findings of this study are available from the corresponding authors upon request. Unprocessed raw data are provided as Supplementary Data 1. Source data are provided with this paper.

## Code availability

The custom MATLAB code used for simulations in this study is provided as Supplementary Data 2 and via Zenodo^66^.
