# Supplementary material for: Transport of enzymatic activity across liquid-liquid interfaces using dynamic assemblies of magnetic particles via field-modulated interactions
Source: Nat Commun. 2026 May 26;17:6872. doi: 10.1038/s41467-026-73696-8 (PMC13388707; doi:10.1038/s41467-026-73696-8)
Supplement: Supplementary file 1 — Supplementary Information [file 41467_2026_73696_MOESM1_ESM.pdf]

Supplementary Information

Transport of enzymatic activity across liquid-liquid interfaces using dynamic assemblies of magnetic particles via field-modulated interactions

Shilu Zhu[1,2], Shuwei Shen[1,2], Min Ye[1,2], Yang Zhang[3], Zhiyuan Zheng[3], Jie Gao[1,2], Ru Zhang[1,2], Zhongliang Lang[1,2], Peng Yao[4], Mingzhai Sun[1,2], Luke P. Lee[5,6,7*] and Ronald X. Xu[1,2,3*]*

[1]School of Biomedical Engineering, Division of Life Sciences and Medicine, University of Science and Technology of China, Hefei, Anhui, 230026, P.R. China.

[2]Suzhou Institute for Advanced Research, University of Science and Technology of China, Suzhou, Jiangsu, 215123, P.R. China.

[3]Department of Precision Machinery and Instrumentation, School of Engineering Science, University of Science and Technology of China, Hefei, Anhui, 230027, P.R. China.

[4]School of Microelectronics, University of Science and Technology of China, Hefei, 230026, P.R. China.

[5]Renal Division and Division of Engineering in Medicine, Department of Medicine, Brigham and Women's Hospital, Harvard Medical School, Boston, MA, USA.

[6]Department of Bioengineering, Department of Electrical Engineering and Computer Science, University of California at Berkeley, Berkeley, CA, USA.

[7]Institute of Quantum Biophysics, Department of Biophysics, Sungkyunkwan University, Suwon, Korea.

*Corresponding authors. Email: mingzhai@ustc.edu.cn (M. S.); lplee@bwh.harvard.edu (L. P. L.); xux@ustc.edu.cn (R. X. X.)

Table of contents:

Supplementary Note 1 to 4
Supplementary Fig. 1 to 35
Supplementary Table 1
Supplementary References

Supplementary Notes

Supplementary Note 1. Magnetic moment dynamics and relaxation.

Under the tailored oscillating magnetic field ($\mathbf{B}(t) = A\sin(2\pi f_x t)\mathbf{e}_x + C\mathbf{e}_z$), the evolution of the particle magnetic moment $\mathbf{m}(t)$ can be described by the Debye relaxation model[1]:

$$\frac{\mathrm{d}\mathbf{m}}{\mathrm{d}t} = -\frac{1}{\tau}\big(\mathbf{m} - \chi_0\mathbf{B}(t)\big) \tag{1}$$

where $\tau = \frac{6\eta\mu_0}{\chi C^2}$ is the magnetization relaxation time and $\chi_0 = \frac{4\pi a^3}{3}\chi$ is the volume magnetic susceptibility. By introducing the complex effective magnetic susceptibility ($\chi_{\mathrm{eff}}(2\pi f_x)$) and assuming the alternating magnetic field $B_x(t) = Ae^{\mathrm{i}2\pi f_x t}$, the steady-state solution for the magnetic moment is obtained as $\mathbf{m}_x(t) = \chi_{\mathrm{eff}}(2\pi f_x)\mathbf{B}_x(t)$. Substituting this into Equation (1) yields:

$$\mathrm{i}2\pi f_x\chi_{\mathrm{eff}} = -\frac{1}{\tau}(\chi_{\mathrm{eff}} - \chi_0) \tag{2}$$

From this, the effective magnetic susceptibility ($\chi_{\mathrm{eff}}(2\pi f_x)$) can be derived as:

$$\chi_{\mathrm{eff}}(2\pi f_x) = \frac{\chi_0}{1 + \mathrm{i}2\pi f_x\tau} \tag{3}$$

Given the horizontal magnetic field component ($B_x(t) = A\sin(2\pi f_x t)$), the magnetic moment response of the particle is:

$$m_x^{\mathrm{eff}}(t) = \mathrm{Re}\left[\chi_{\mathrm{eff}}(2\pi f_x)Ae^{\mathrm{i}2\pi f_x t}\right] = \frac{\chi_0 A}{\sqrt{1 + (2\pi f_x\tau)^2}}\sin(2\pi f_x t - \phi) \tag{4}$$

where $\phi = \arctan(2\pi f_x\tau)$ is the phase lag. Therefore, the amplitude of the effective magnetic moment is:

$$m_x^{\mathrm{eff}} = \frac{\chi_0 A}{\sqrt{1 + (2\pi f_x\tau)^2}} = \frac{\chi_0 A}{\sqrt{1 + (f_x/f_{\mathrm{cut}})^2}} \tag{5}$$

where $f_{\mathrm{cut}} = \frac{1}{2\pi\tau} = \frac{\chi C^2}{12\pi\eta\mu_0}$ is the cut-off frequency. When the driving frequency (f_x) exceeds the cut-off frequency (f_{cut}), the horizontal component of the particle's magnetic moment (m_x) begins to exhibit amplitude attenuation and phase lag (Supplementary Fig. 15). Once the amplitude of the magnetic moment decays to a specific range at a defined amplitude ratio (γ), the dynamic assembly process progressively shifts toward the vertical direction. As the frequency further increases, the transverse magnetic moment amplitude attenuates to a negligible value, resulting in an assembled structure equivalent to magnetic pillars formed under the sole influence of a vertical static magnetic field.

Supplementary Note 2. Dynamic vertical growth model.

The vertical growth can be characterized by a continuous collision-fusion process, where the growth efficiency is primarily governed by the frequency-dependent collision efficiency $k(f_x)$. The temporal evolution of structural unit number $N(t)$ during these merging processes follows a modified Smoluchowski Equation[2]:

$$\frac{\mathrm{d}N}{\mathrm{d}t} = -\,k(f_x)\cdot\left(1-\frac{N_{\mathrm{eq}}(f_x)}{N}\right)\cdot\left(\frac{N}{A_{\mathrm{s}}}\right)^2 \tag{6}$$

Here, $N_{\mathrm{eq}}(f_x)$ denotes the equilibrium number of MAFS, while the term $(1-\frac{N_{\mathrm{eq}}(f_x)}{N})$ introduces dynamic collision efficiency modulation during system evolution, reflecting the density-dependent sensitivity of collision rates. A_{s} denotes the substrate area, and $\left(\frac{N}{A_{\mathrm{s}}}\right)^2$ characterizes the two-dimensional collision probability. Assuming longitudinal uniformity of MAFS and mass conservation, the relationship between structural number and heights can be expressed as:

$$N\cdot h = N_0\cdot h_0 = N_{\mathrm{eq}}\cdot h_{\mathrm{max}} \tag{7}$$

where N_0 and h_0 represent the initial number and height at magnetic field activation, respectively, and $h_{\mathrm{max}}(f_x)$ indicates the frequency-dependent maximum attainable height. Solving the differential Equation (6) analytically yields the vertical growth model:

$$\begin{cases} N(t) = \dfrac{N_{\mathrm{eq}}}{1-\left(1-\dfrac{N_{\mathrm{eq}}}{N_0}\right)e^{-N_{\mathrm{eq}}Ct}} \\ h(t) = h_{\mathrm{max}}\left(1-\left(1-\dfrac{h_0}{h_{\mathrm{max}}}\right)e^{-N_{eq}Ct}\right) \end{cases},\ C=\frac{k(f_x)}{A_{\mathrm{s}}^2} \tag{8}$$

In this model, the collision efficiency $k(f_x)$ is determined by magnetic field parameters, including the amplitude ratio and field strength. Using this framework, we have characterized dynamic growth patterns evolving over time at different frequencies (γ = 3), as demonstrated in Supplementary Fig. 21 and Supplementary Fig. 22.

Supplementary Note 3. Segment formation of MAFS.

Under high-frequency oscillating magnetic fields, magnetic particles initially self-assemble into the root structures of MAFS. With continued particle accumulation, the structure undergoes vertical elongation. Upon surpassing a critical height threshold, its morphology transitions abruptly from continuous pillar-like forms to segmented configurations. These segments remain tightly connected through vertical magnetic dipole-dipole interactions, allowing independent vibrations without structural disintegration. The segmentation phenomenon arises from local structural weakening and the system's tendency toward mechanically stable configurations. Specifically, the transition initiates when shear-induced stresses surpass the interparticle magnetic adhesion strength, disrupting the structural continuity. Concurrently, the system's dynamic vibrational modes amplify this effect, promoting the formation of discrete yet magnetically linked segments.

The growing MAFS can be modeled as an elastic beam with time-varying height $h(t)$, governed by magneto-elastic coupling dynamics. The segmentation behavior of MAFS is modeled by Euler-Bernoulli Beam Equation[3,4]:

$$E_{\text{eff}} I \frac{\partial^4 u}{\partial z^4} + \rho_{\text{eff}} A_{\text{sec}} \frac{\partial^2 u}{\partial t^2} + c \frac{\partial u}{\partial t} = F_{\text{drive}}(z, t) \tag{9}$$

where $E_{\text{eff}} = \frac{3\mu_0 \chi^2 B_z^2}{4\pi d^2}$ represents the effective magneto-elastic stiffness, I is the cross-sectional moment of inertia, $\rho_{\text{eff}} A_{\text{sec}}$ is the linear mass density of the structure, A_{sec} is the sectional area, c is the viscous damping coefficient, $u(z, t)$ is lateral displacement at height (z), and $F_{\text{drive}}(z, t)$ is the periodic driving term. Before analyzing the forced vibration, it is essential to first discuss the free vibration behavior of the structure (neglecting the damping effect to facilitate the interpretation of the segment formation of MAFS). Equation (9) can be simplified as follows:

$$E_{\text{eff}} I \frac{\partial^4 u}{\partial z^4} + \rho_{\text{eff}} A_{\text{sec}} \frac{\partial^2 u}{\partial t^2} = 0 \tag{10}$$

Assuming the solution to be in the form of a standing wave ($u(z, t) = U(z)\sin(\omega_n t)$) and substituting it into Equation (10) yields:

$$\frac{\mathrm{d}^4 U}{\mathrm{d}z^4} - \beta_n^4 U = 0, \qquad \beta_n^4 = \frac{\rho_{\text{eff}} A \omega_n^2}{E_{\text{eff}} I} \tag{11}$$

where $\omega_n = 2\pi f_n$ is the natural angular frequency of the n-th vibration mode. Under the assumption of a clamped-free boundary configuration (fixed at the bottom, $z = 0$, and free at the top, $z = h$), the displacement field satisfies the clamped conditions ($U(0) = U'(0) = 0$) at the base and the free-end conditions ($U''(h) = U'''(h) = 0$) at the tip. The resulting natural modal shape function can be written as:

$$U_n(z) = \cosh(\beta_n z) - \cos(\beta_n z) - \frac{\cosh(\beta_n h) + \cos(\beta_n h)}{\sinh(\beta_n h) + \sin(\beta_n h)}(\sinh(\beta_n z) - \sin(\beta_n z)) \tag{12}$$

where $\beta_n h$ satisfies the eigenvalue Equation:

$$\cosh(\beta_n h)\cos(\beta_n h) + 1 = 0 \tag{13}$$

For higher-order modes ($n \geq 2$), the approximate solution is:

$$\beta_n h \approx \frac{(2n-1)\pi}{2} \tag{14}$$

The nodal positions of the n-th vibration mode approximately satisfy:

$$z_k \approx \frac{(2k-1)h}{2(2n-1)}, \quad (k = 1, 2 \ldots, n-1) \tag{15}$$

Therefore, the distance between adjacent nodes is:

$$\Delta z_n = z_{k+1} - z_k = \frac{h}{2n-1} \tag{16}$$

From Equations (11) and (14), the natural frequency can be expressed as:

$$f_n = \frac{(2n-1)^2\pi}{8h^2}\sqrt{\frac{E_{\mathrm{eff}}I}{\rho_{\mathrm{eff}}A}} \tag{17}$$

When the driving frequency (f_x) of the external magnetic field is held constant, the vertical growth of the MAFS increases its height (h), causing its natural frequency to gradually decrease (since $f_n \propto 1/h^2$). As the natural frequency approaches the driving frequency, resonance occurs when $f_n = f_x$, exciting higher-order vibration modes that induce segmentation. This dynamic adaptation suppresses structural resonance through self-organized segmentation. By segmenting into semi-independent vibrating units, each subunit maintains a natural frequency significantly higher than the driving frequency ($f_n \gg f_x$) due to reduced effective height. This prevents resonant amplification by dispersing vibrational energy through localized oscillations of individual segments.

Supplementary Note 4. Coupled oscillation of segmented MAFS.

Consider an individual MAFS composed of N segments, where the i-th segment (S_i) has a length L_i and a time-dependent inclination angle $\theta_i(t)$. Taking all the physical effects into account, the oscillating motion of S_i is governed by:

$$I_i \frac{\mathrm{d}^2\theta_i}{\mathrm{d}t^2} + c_i \frac{\mathrm{d}\theta_i}{\mathrm{d}t} + \Gamma_i^{\mathrm{res}} = \Gamma_i^{\mathrm{mag}}(t) + \sum_j \Gamma_{i,j}^{\mathrm{dipole}} \tag{18}$$

where I_i and c_i are moment of inertia of S_i and fluid drag coefficient, respectively. Γ_i^{res} is the restoring torque generated by the vertical constant field, which is expressed by:

$$\Gamma_i^{\mathrm{res}} = |\boldsymbol{\mu}_i L_i \times \mathbf{B}_z| = \mu_i L_i B_z \sin\theta_i \tag{19}$$

Under the small-angle approximation ($\sin\theta_i \approx \theta_i$), Equation (19) can be simplified to:

$$\Gamma_i^{\mathrm{res}} \approx \mu_i L_i B_z \theta_i = k_i \theta_i \tag{20}$$

where $k_i = \mu_i L_i B_z$ is the rotational stiffness. Meanwhile, the actuating magnetic torque $\Gamma_i^{\mathrm{mag}}(t)$ induced by the horizontal oscillating field is:

$$\Gamma_i^{\mathrm{mag}}(t) = |\boldsymbol{\mu}_i L_i \times \mathbf{B}_x(t)| = \mu_i L_i B_x \sin(2\pi f_x t) \sin\left(\frac{\pi}{2} - \theta_i\right) = \mu_i L_i B_x \sin(2\pi f_x t) \cos\theta_i \tag{21}$$

Applying $cos\theta_i \approx 1$ (small-angle approximation), this further simplifies to:

$$\Gamma_i^{\mathrm{mag}}(t) = \mu_i L_i B_x \sin(2\pi f_x t) \tag{22}$$

Additionally, $\sum_j \Gamma_{i,j}^{\mathrm{dipole}}$ represents the coupled magnetic dipole-dipole torque between segments, which can be expressed as:

$$\sum_j \Gamma_{i,j}^{\mathrm{dipole}} = \sum_j C_{i,j} \sin(\theta_j - \theta_i) \approx \sum_j C_{i,j}(\theta_j - \theta_i) \tag{23}$$

where $C_{i,j} = \frac{3\mu_0 \mu_i \mu_j L_i L_j}{4\pi d_{i,j}^3}$ is the magnetic dipole torque coefficient. Combining Equations (20), (22) and (23), the Equation of oscillating motion (18) becomes:

$$I_i \frac{\mathrm{d}^2\theta_i}{\mathrm{d}t^2} + c_i \frac{\mathrm{d}\theta_i}{\mathrm{d}t} + k_i \theta_i = \mu_i L_i B_x \sin(2\pi f_x t) + \sum_j C_{i,j}(\theta_j - \theta_i) \tag{24}$$

The steady-state solution of Equation (24) can be written as:

$$\theta_i(t) = \Theta_i \sin(2\pi f_x t - \phi_i) \tag{25}$$

where Θ_i and ϕ_i are the oscillating amplitude and phase lag of S_i, respectively. Substituting Equation (25) into Equation (24) yields the amplitude and phase relationships:

$$\Theta_i = \frac{k_i}{\sqrt{(k_i + \Delta k_i - I_i(2\pi f_x)^2)^2 + (2c_i\pi f_x)^2}} \tag{26}$$

$$\phi_i = \arctan\left(\frac{2c_i\pi f_x}{k_i + \Delta k_i - I_i(2\pi f_x)^2}\right) \tag{27}$$

where $\Delta k_i = \sum_j C_{i,j}\big(1 - \cos(\phi_j - \phi_i)\big)$ is the stiffness correction due to inter-segment coupling, which modifies the effective stiffness based on the phase differences between adjacent segments. Consequently, the intrinsic frequency of segment S_i within the coupled system is:

$$\omega_{0,i} = \sqrt{\frac{k_i + \Delta k_i}{I_i}} \tag{28}$$

The segmented MAFS exhibit unique dynamic behavior due to its semi-independent oscillating units. While each segment synchronizes with the external magnetic field at frequency f_x, their responses vary in amplitude (Θ_i) and phase (ϕ_i). This asynchrony between adjacent segments causes the high-frequency vibrating structure to dynamically exhibit a stacked X-shaped morphology. Such behavior not only enhances mechanical robustness but also enables adaptive reconfiguration, highlighting the distinctive properties of MAFS in dynamic fluid environments.

Supplementary Figures

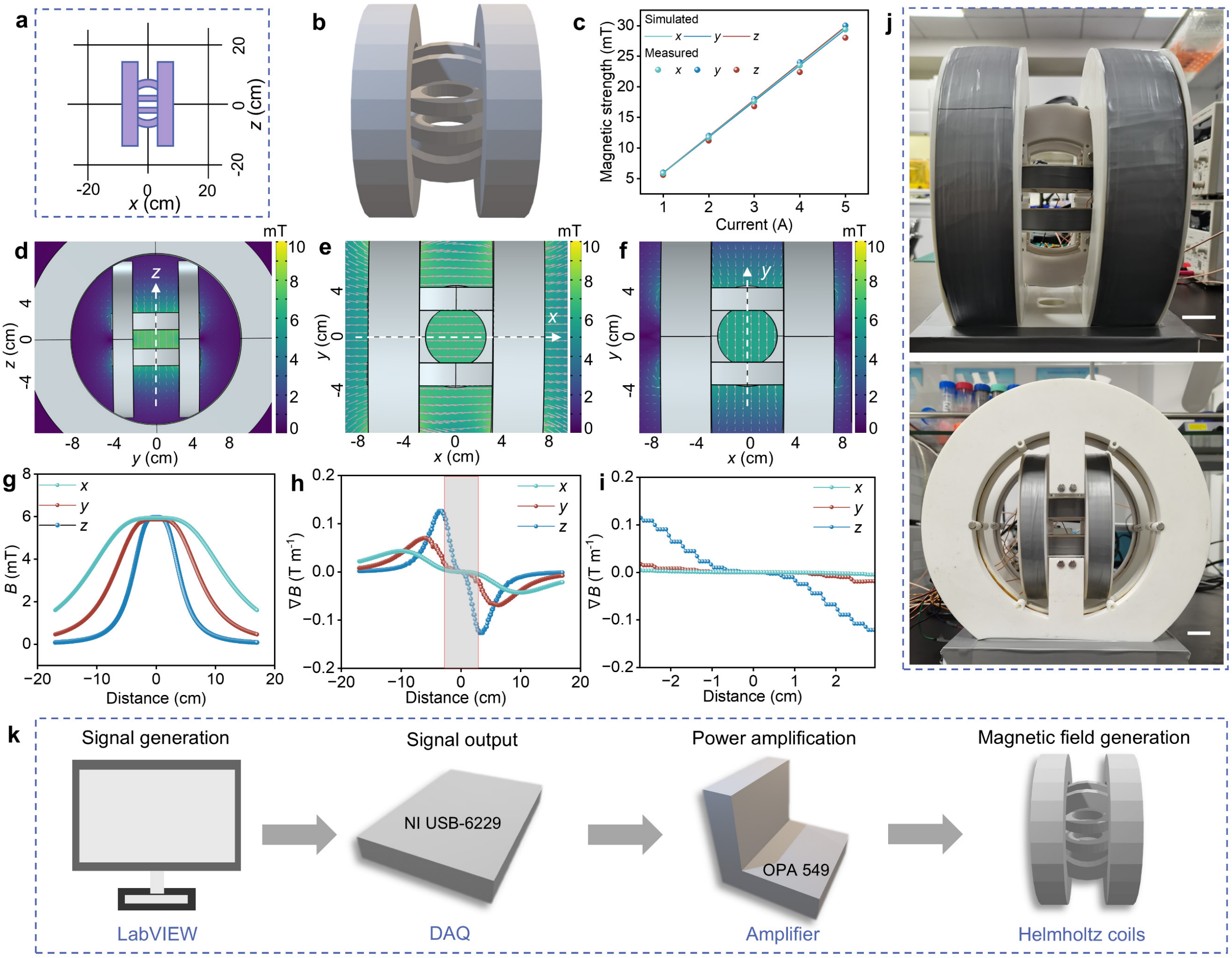

Supplementary Fig. 1 | Design of electromagnetic actuation system. a, b Model of the electromagnetic coils array, showing its spatial configuration. **c** Simulated and measured magnetic field strengths under various output currents. **d-f** Spatial distribution of magnetic flux density within the actuation workspace. **g, h** Vector field analysis showing the magnetic field strength and gradient along *x*/*y*/*z* axes. **i** Enlarged view indicated in **h**. **j** Photograph of the experimental setup for magnetic actuation. Scale bar, 2 mm. **k** Schematic of the magnetic field generation and control. Source data for Supplementary Fig. 1 are provided as a Source Data file.

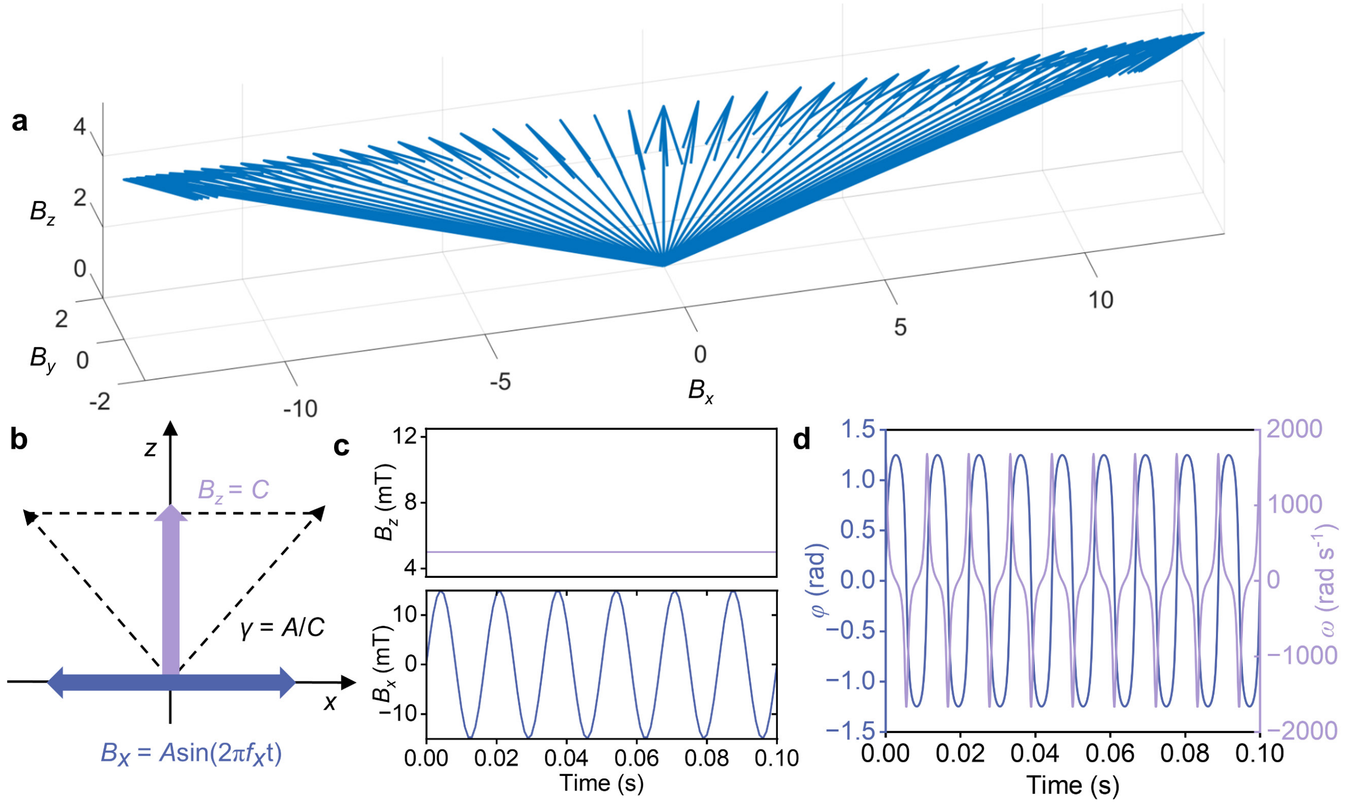

Supplementary Fig. 2 | Applied oscillating magnetic field. a Three-dimensional schematic of the oscillating magnetic field components. **b** Two-dimensional schematic illustrating the oscillating magnetic field components in two directions. The blue arrows represent the oscillating field along the *x*-axis, and the purple arrows denote the constant vertical field. **c** Temporal variation of the magnetic field components in both directions under the conditions B_z = 5 mT, f_x = 90 Hz, and γ = 3. **d** Temporal variation of the magnetic field components in both directions under the conditions f_x = 90 Hz, γ = 3, and B_z = 5 mT. Source data for Supplementary Fig. 2 are provided as a Source Data file.

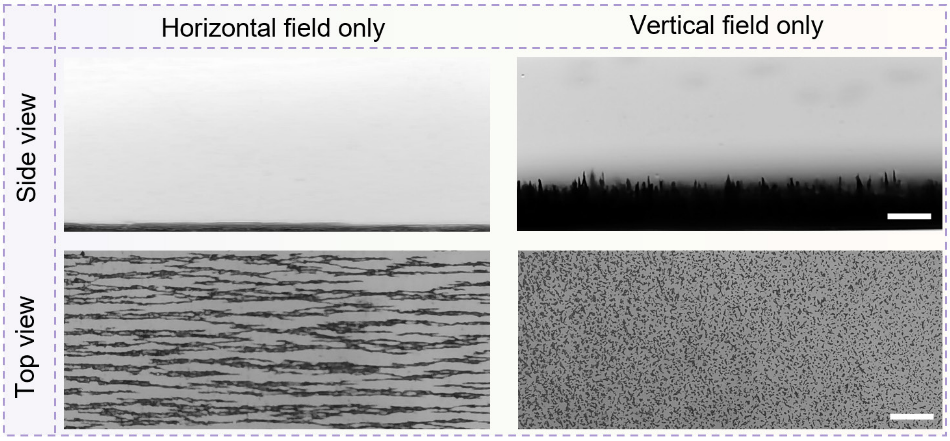

Supplementary Fig. 3 | Magnetic microstructures under individual field configurations: (i) horizontal oscillating field (B_x = 10 mT, f_x = 3 Hz), and (ii) constant vertical field (B_z = 5 mT). Neither field component alone enables sustained vertical growth against gravity: the horizontal oscillating field alone lacks vertical interparticle interactions, while the static vertical field cannot induce time-varying interparticle reorganization required for dynamic structural growth. Scale bar, 2 mm.

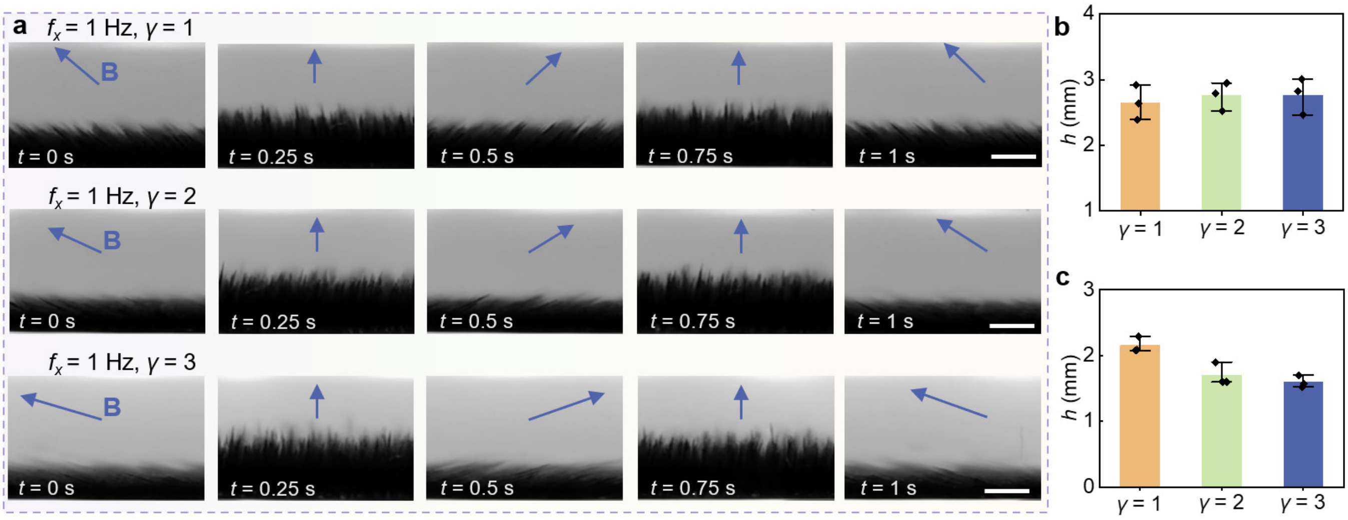

Supplementary Fig. 4 | Swinging behavior of magnetic pillars under oscillating magnetic field with varying amplitude ratios γ = 1, 2, and 3 (f_x = 1 Hz, B_z = 5 mT). a Time-lapse image of the swinging motion of magnetic pillars subjected to the oscillating magnetic fields. **b** Height of magnetic pillars when the magnetic field direction oscillates horizontally to either side. **c** Height of magnetic pillars in the vertical orientation. Scale bar, 2 mm. Data in **b** and **c** are presented as mean ± SD (n = 3 independent measurements). Source data for Supplementary Fig. 4 are provided as a Source Data file.

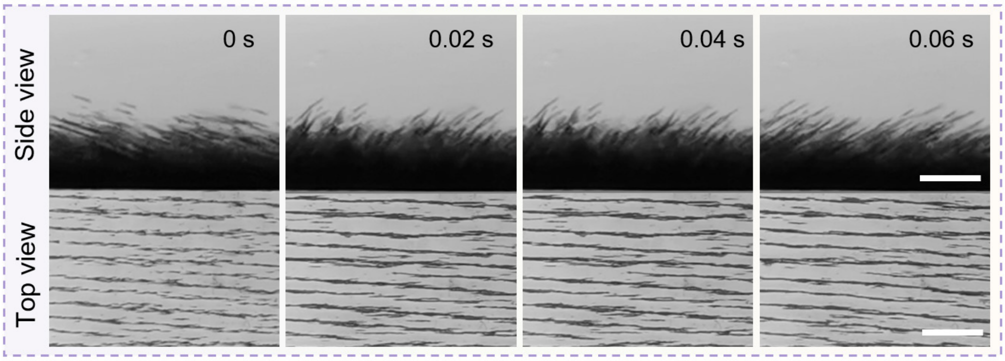

Supplementary Fig. 5 | Fragmentation of magnetic pillars and the formation of uncontrolled, massive pillar-like structures under oscillating magnetic fields with amplitude ratio $\gamma = 2$, $f_x = 10$ Hz, and $B_z = 5$ mT. Under this regime, when the magnetic torque fails to overcome the viscous torque and gravitational torque, fragmentation of the magnetic pillars occurs[5]. Scale bar, 2 mm.

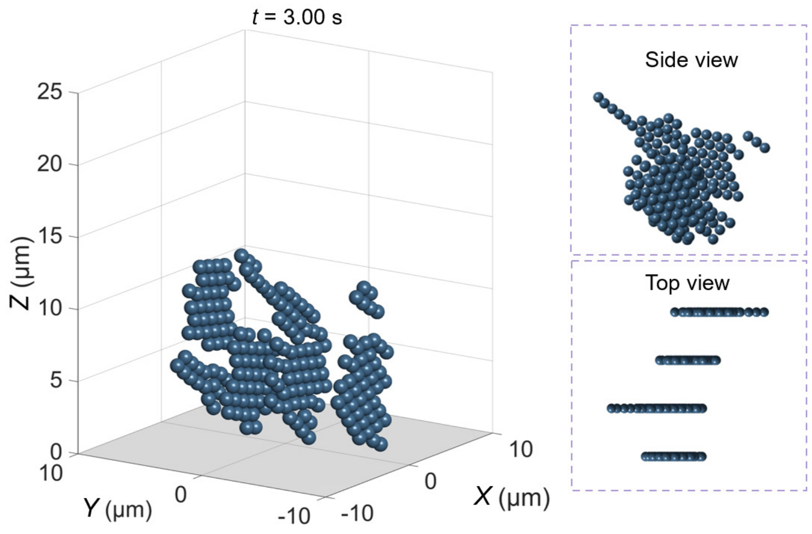

Supplementary Fig. 6 | Representative simulation of vertical collective formation via dynamic particle assembly in the medium frequency range (f_x = 40 Hz, B_z = 5 mT, B_x = 15 mT, N = 200).

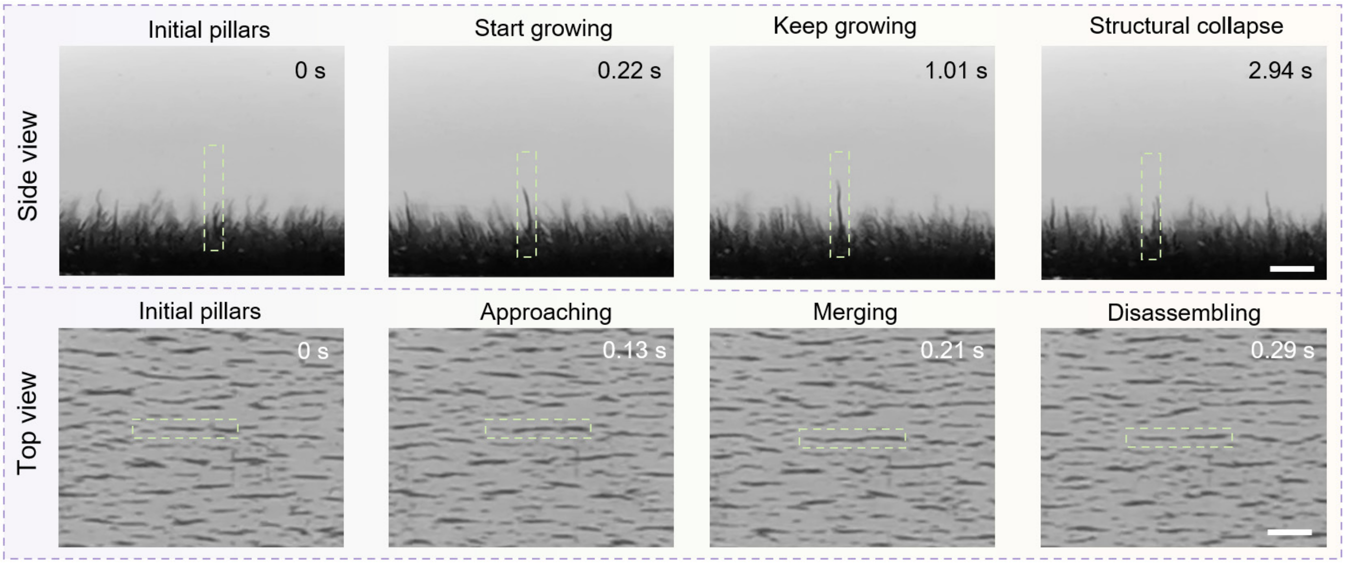

Supplementary Fig. 7 | Critical behavior at the transition between phase I and Phase II. As the frequency of the oscillating magnetic field approaches the critical point, adjacent magnetic pillars briefly ascend alongside the main pillar but subsequently detach, resulting in the interruption of vertical growth. Scale bar, 2 mm.

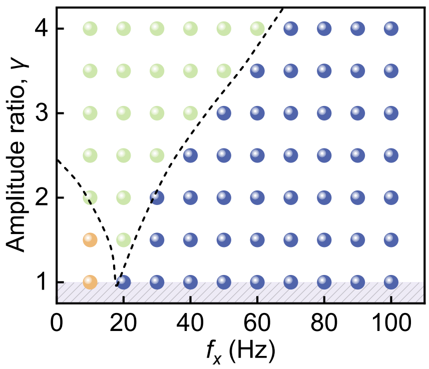

Supplementary Fig. 8 | Phase diagram showing the magnetic structures generated by oscillating magnetic fields at B_z = 3 mT. Reduction of B_z from 5 mT to 3 mT causes leftward phase boundary shifts, and weaker B_z strength inhibits vertical growth due to inadequate magnetic interactions in the vertical direction. The purple shaded region denotes the parameter space ($\gamma < 1$) where the transverse field strength is insufficient to initiate vertical growth. Source data for Supplementary Fig. 8 are provided as a Source Data file.

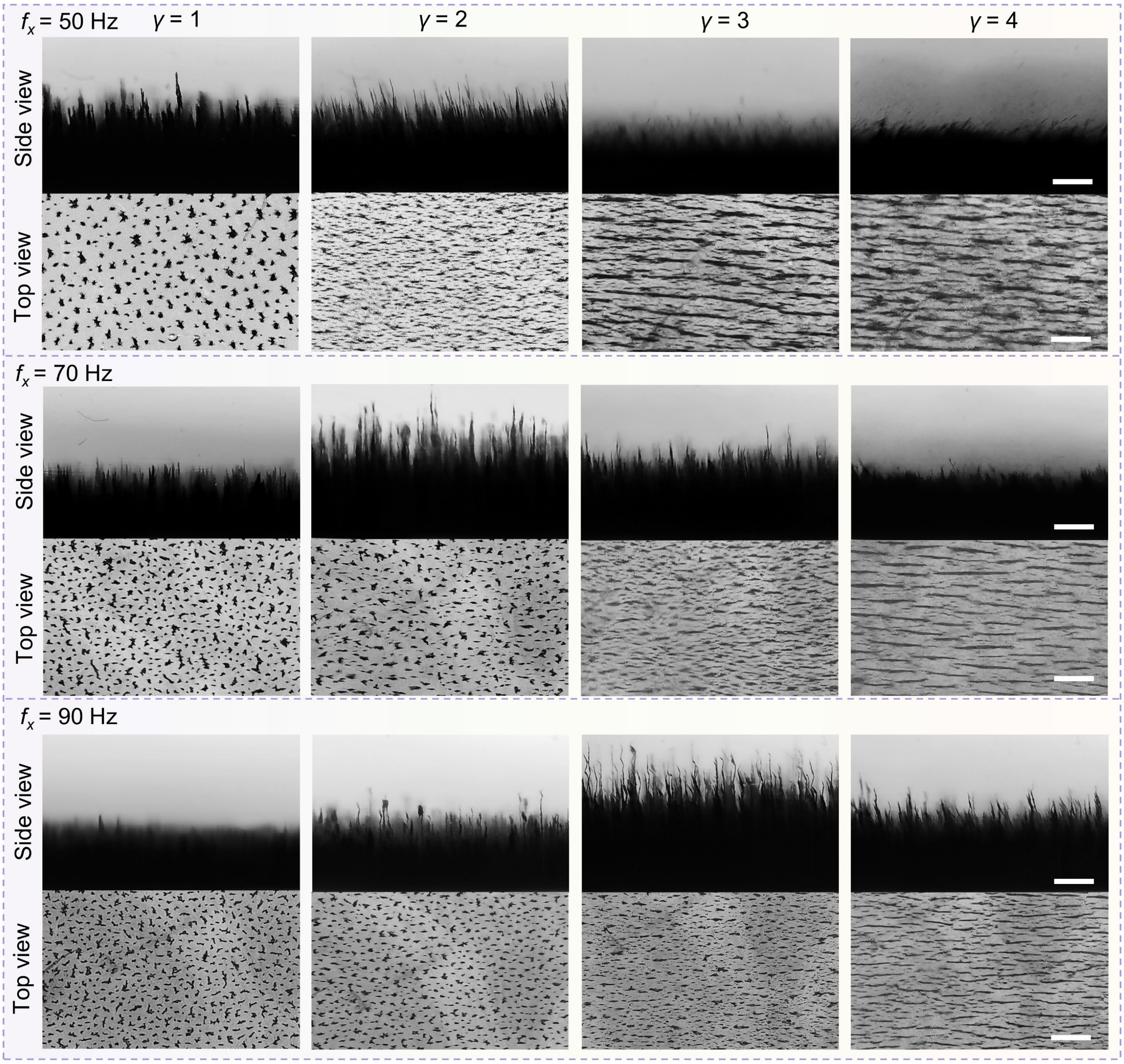

Supplementary Fig. 9 | Influence of oscillating frequency and amplitude ratio on the vertical growth of MAFS. Scale bar, 2 mm.

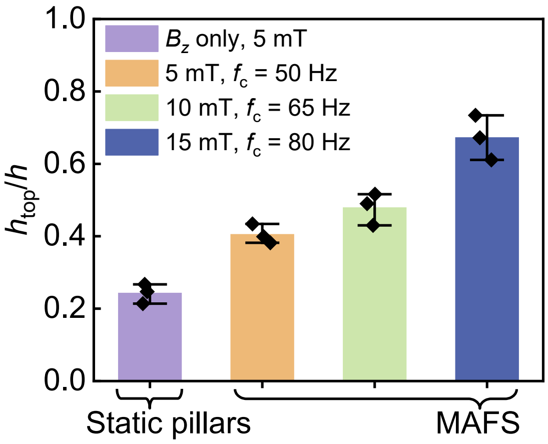

Supplementary Fig. 10 | Oscillation field strength dependence of the height ratio between top section (h_{top}) and total structure height (h) at $\gamma = 1$. h_{top} is defined as the upper segment height as annotated in Fig. 3i. Data are presented as mean ± SD (n = 3 independent measurements). Source data for Supplementary Fig. 10 are provided as a Source Data file.

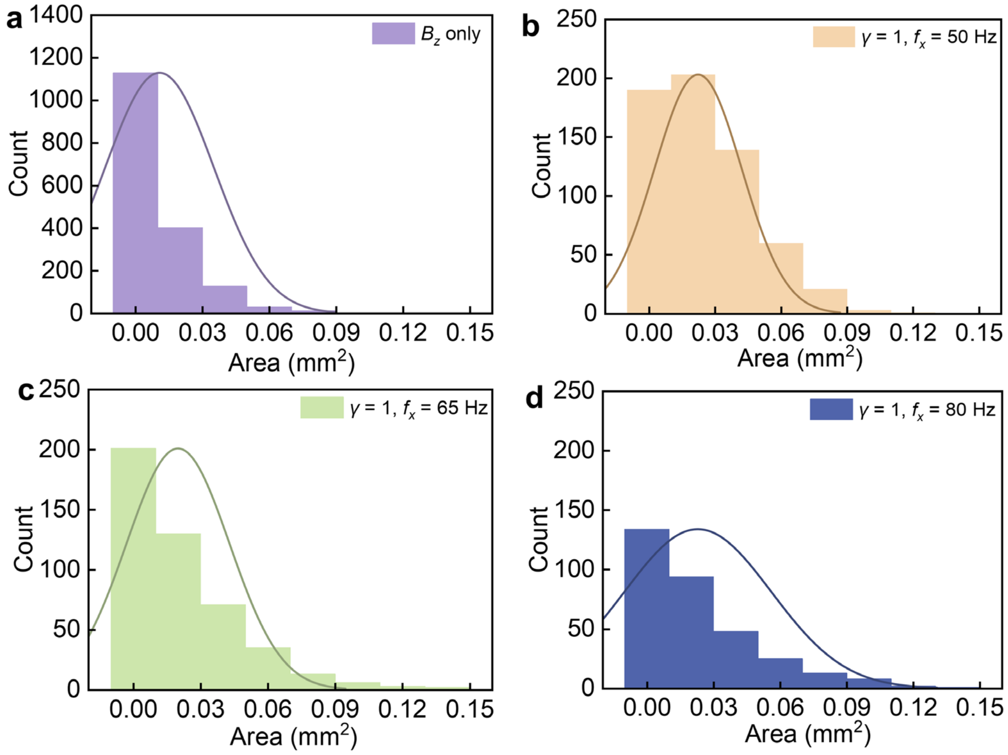

Supplementary Fig. 11 | Variation in MAFS count as a function of occupied area under different vertical magnetic field strengths. a Count of MAFS under a purely vertical magnetic field (B_z = 5 mT). Panels **c-d** show that increasing the field strength (B_z = 5, 10, and 15 mT; γ = 1) together with the corresponding critical frequencies (50, 65, and 80 Hz) reduces the total number of MAFS while promoting the formation of larger structures, indicating a merging behavior. Source data for Supplementary Fig. 11 are provided as a Source Data file.

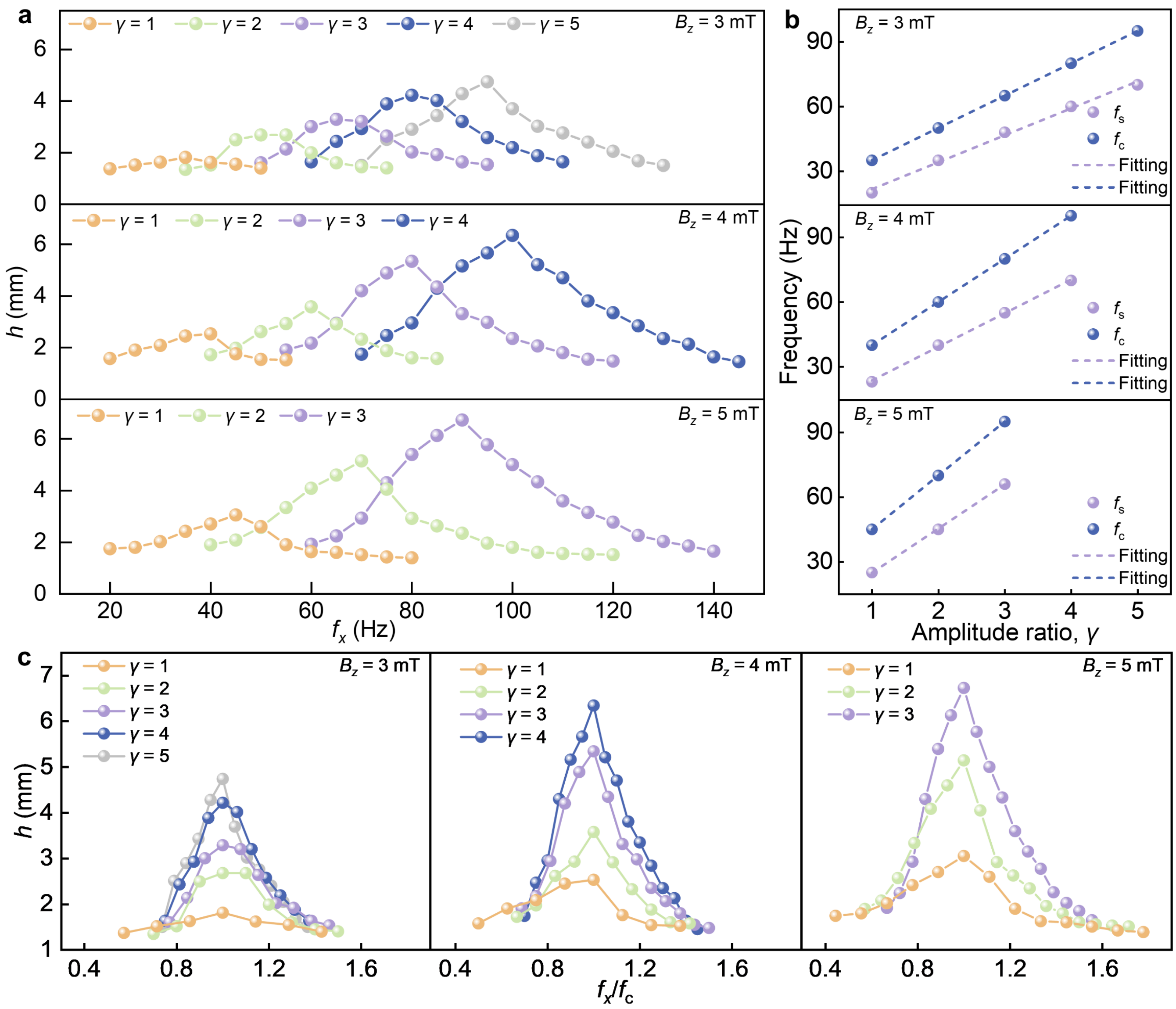

Supplementary Fig. 12 | Parametric study and normalized scaling of MAFS growth. a MAFS height as a function of driving frequency f_x, measured across static field strengths $B_z = 3$, 4, and 5 mT and a range of amplitude ratios γ. **b** Start frequency (f_s) and critical frequency (f_c) extracted for each parameter set (B_z, γ). **c** Normalized diagrams obtained by scaling the driving frequency f_x with the corresponding f_c. The growth region collapses to the normalized window $0.4 < f_x/f_c < 2.0$, revealing a universal scaling law that links the extent of the operational frequency range to the resulting growth height across different field conditions. Source data for Supplementary Fig. 12 are provided as a Source Data file.

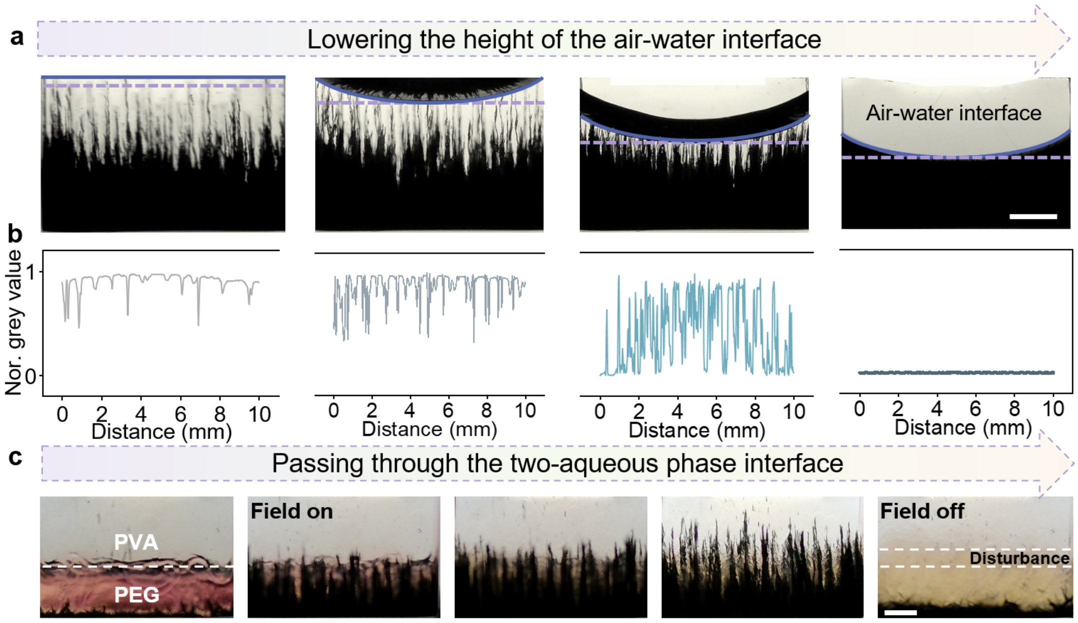

Supplementary Fig. 13 | Interfacial effects on MAFS growth. a Growth constraint imposed by the air-liquid interface, with the purple dashed line indicating the interface tangent. Scale bar, 2 mm. **b** Light transmission intensity profile measured along the tangent line in **a**. **c** MAFS penetration dynamics across a biphasic aqueous interface (lower phase: 10 wt.% PEG; upper phase: 4 wt.% PVA). Scale bar, 2 mm. Source data for Supplementary Fig. 13 are provided as a Source Data file.

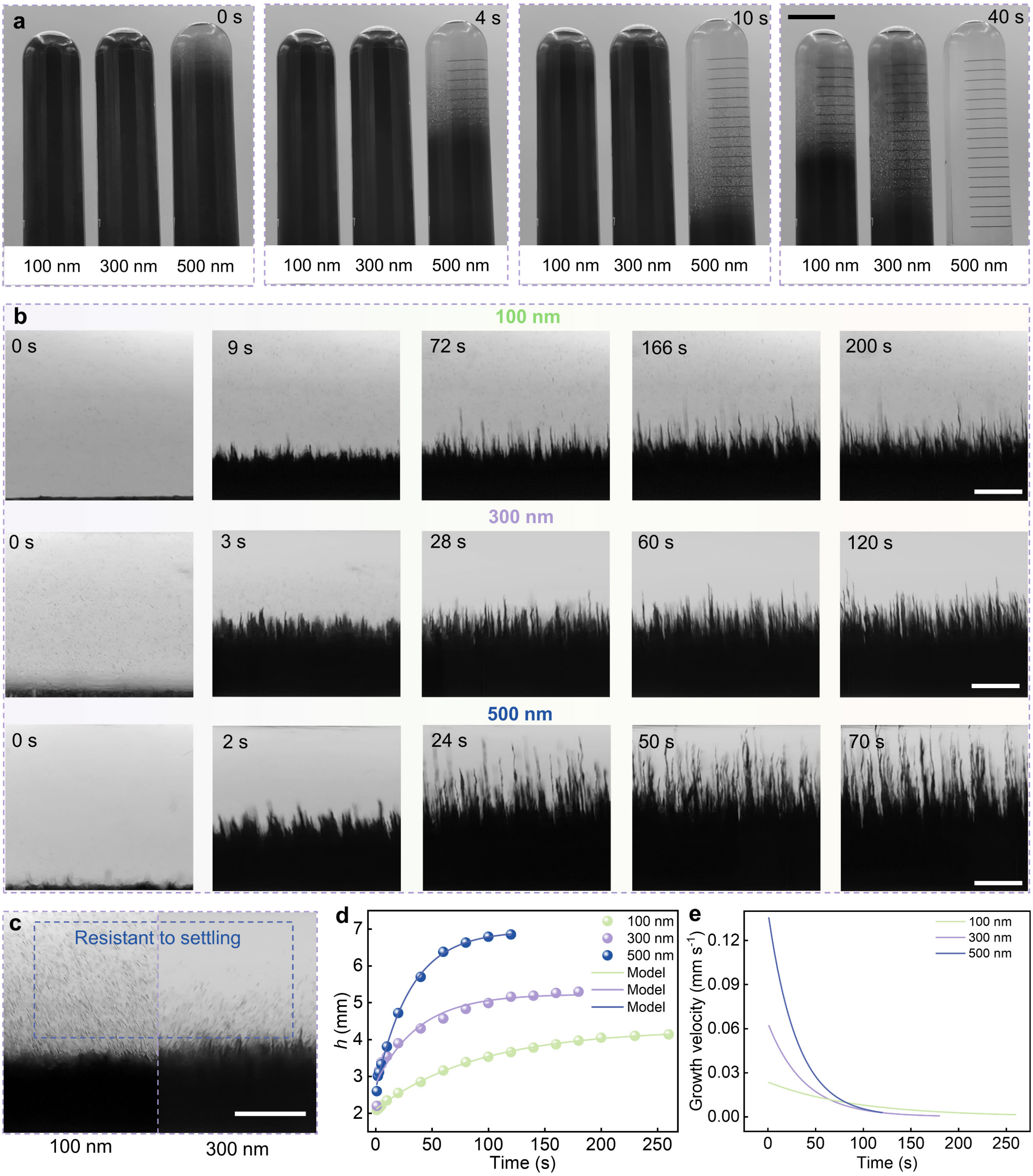

Supplementary Fig. 14 | Dynamic self-assembly behavior of nanoscale magnetic particles. a Sedimentation behavior of Fe_3O_4 particles (100, 300, and 500 nm) in aqueous solution. Scale bar, 2 mm. **b** Time-lapse images of vertical growth for different particle sizes. Scale bar, 2 mm. **c** Particle dynamics under magnetically induced flow, showing that 100 and 300 nm particles are readily suspended in solution due to their enhanced mobility under field-induced disturbance. Scale bar, 2 mm. **d** Temporal evolution of MAFS height at varying particle sizes. **e** Growth velocity versus particle size, highlighting slower kinetics for smaller particles. Source data for Supplementary Fig. 14 are provided as a Source Data file.

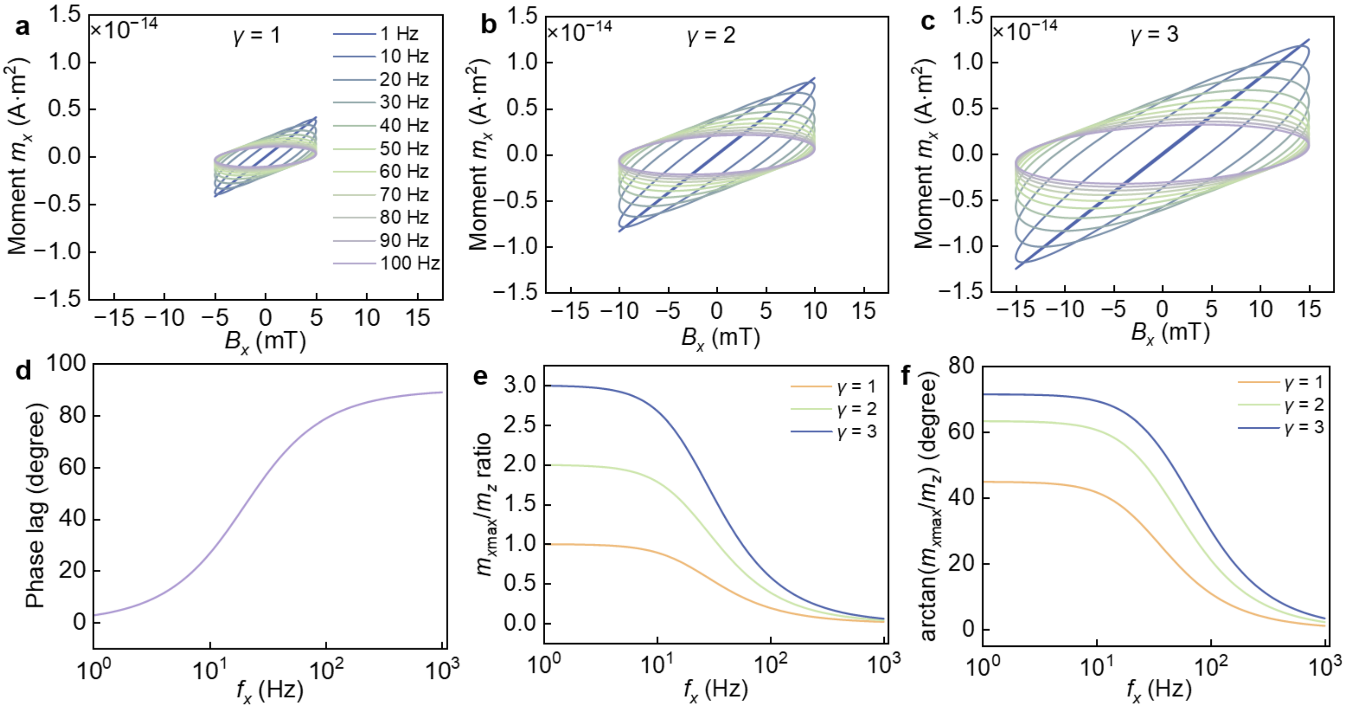

Supplementary Fig. 15 | Magnetic moment relaxation dynamics. a-c Dynamic hysteresis loops calculated using the Debye relaxation model at different frequencies (1–100 Hz; B_z = 5 mT; γ = 1, 2, and 3). **d** Frequency dependence of the phase lag angle between the magnetic moment response and the applied field. **e** Normalized transverse moment amplitude (m_{xmax}/m_z) as a function of frequency (γ = 1, 2, 3). The amplitude remains frequency-independent at low frequencies but decays progressively as frequency increases, eventually approaching zero. **f** Phase response (arctan(m_{xmax}/m_z)) as a function of frequency. Source data for Supplementary Fig. 15 are provided as a Source Data file.

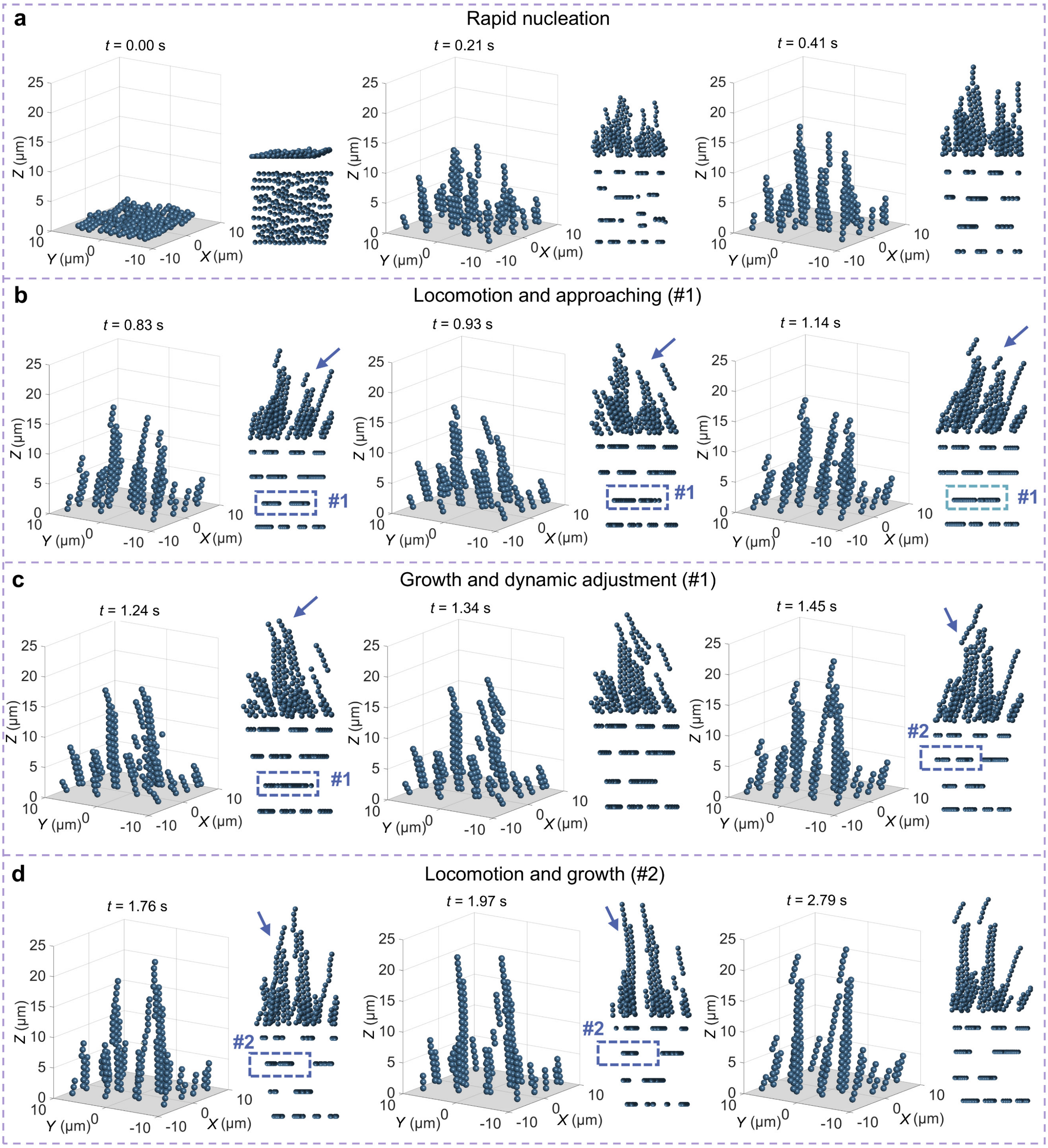

Supplementary Fig. 16 | Simulation of growth process under tailored vertical oscillating magnetic field (B_z = 5 mT, γ = 3, f_x = 90 Hz; 200 particles, diameter: 1 μm). **a** Rapid nucleation of dispersed magnetic particles. **b** Locomotion and approach of primary structures, with #1 marking the merging process of two approaching units. **c** Structural coalescence and growth of two primary units, where #2 denotes indicates another merging event. **d** Subsequent coalescence and elongation of another interacting pair under field guidance.

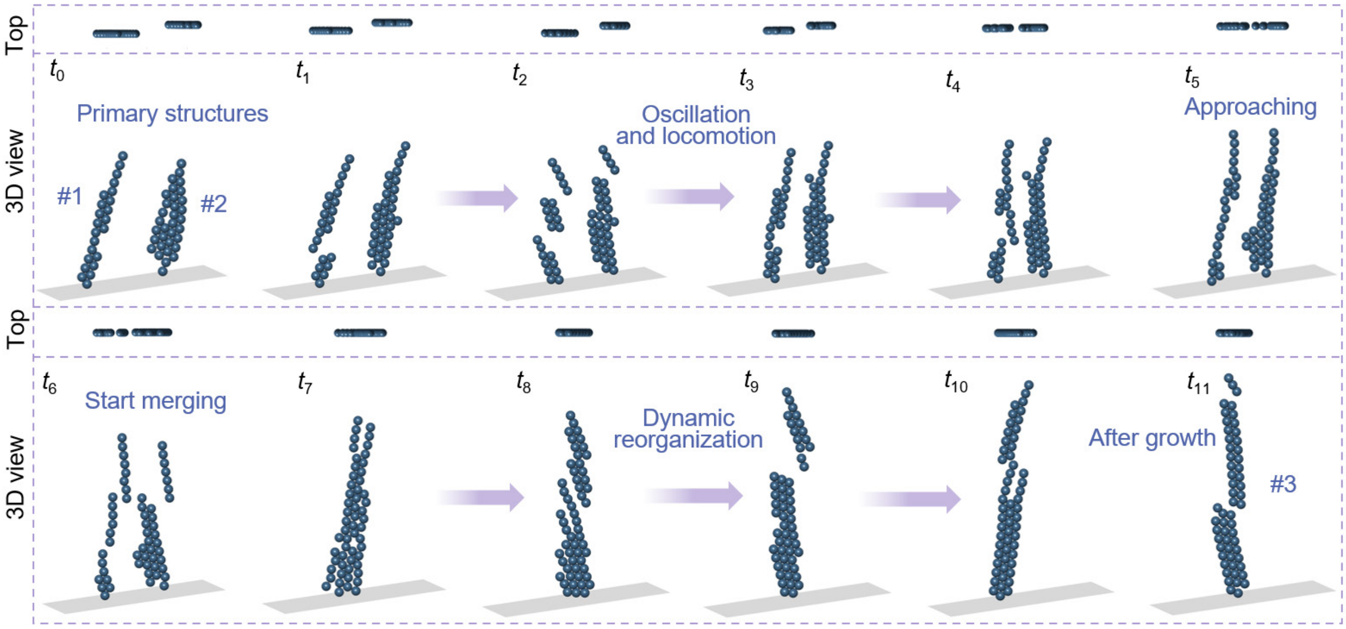

Supplementary Fig. 17 | Simulation of the dynamic growth process from two primary nucleation structures, shown in top and 3D views (f_x = 90 Hz, B_z = 5 mT, γ = 3).

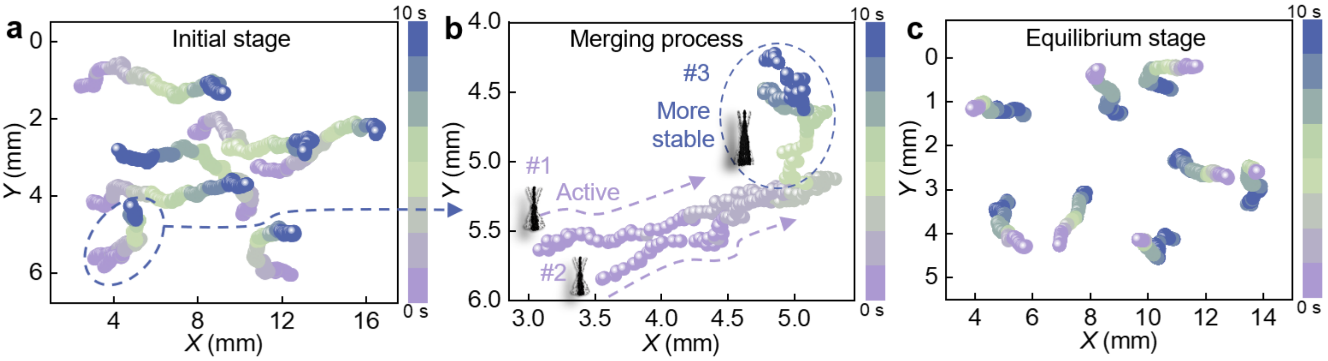

Supplementary Fig. 18 | Planar trajectories of MAFS during vertical growth. a Horizontal trajectory of eight MAFS showing the initial growth phase (0–10 s) during vertical development. **b** Enlarged view of the circled region in **a**, showcasing a representative coalescence event between two adjacent units. **c** Horizontal plane trajectories of eight MAFS showing dynamic equilibrium stage (100–110 s). As the units collided and merged, the MAFS gradually shifted from a relatively active state to a dynamically stable one. Source data for Supplementary Fig. 18 are provided as a Source Data file.

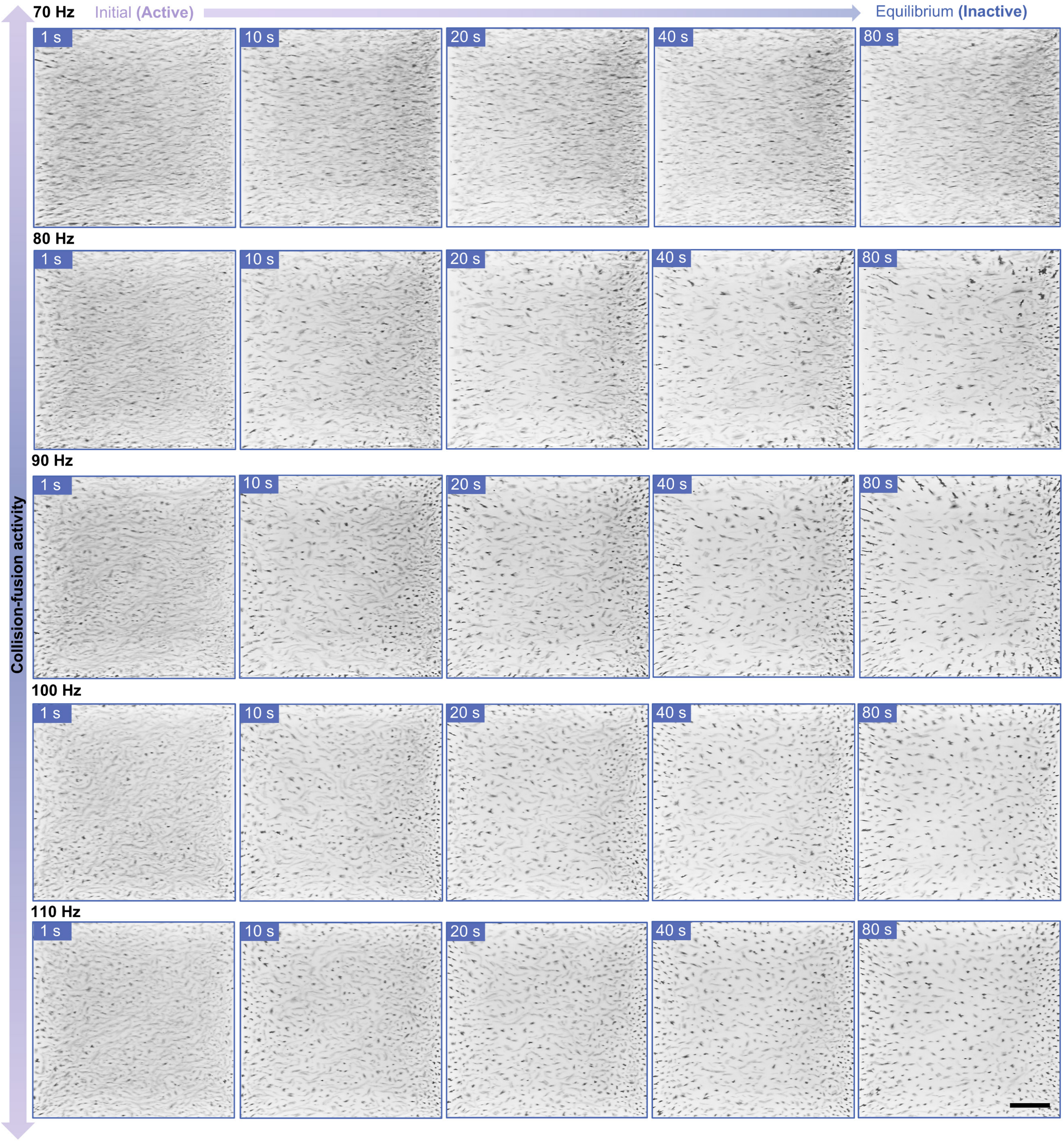

Supplementary Fig. 19 | Time-lapse trajectory overlays of MAFS at varying frequencies, illustrating the evolution of microscopic activity (γ = 3, B_z = 5 mT). Superimposed images (60 frames over 1 s) of MAFS at different time points (1, 10, 20, 40, and 80 s after magnetic field application) under driving frequencies of 70, 80, 90, 100, and 110 Hz. The trajectories visualize the distinct microscopic collision-fusion activity patterns, transitioning from the initial response to a dynamic equilibrium state. Scale bar, 2 mm.

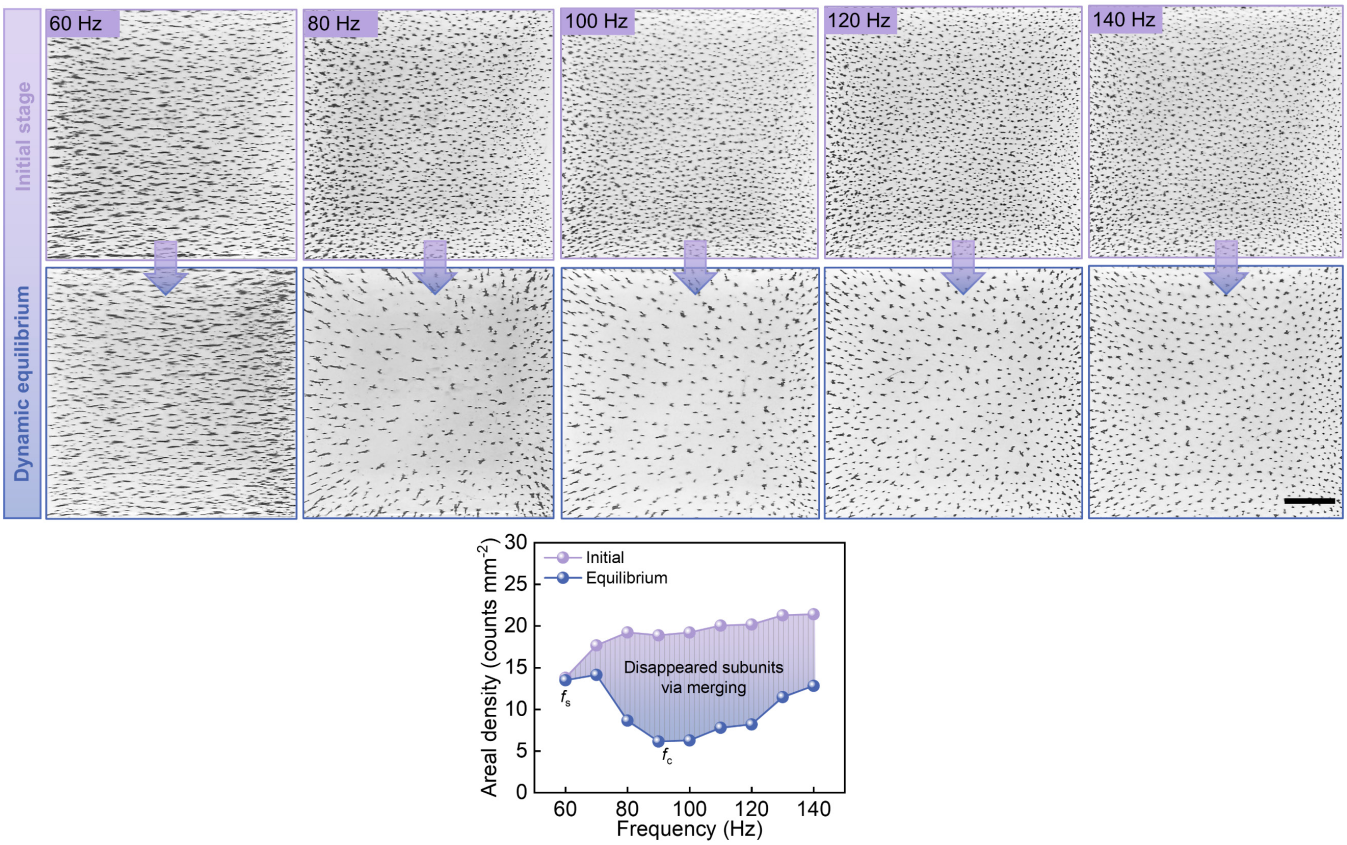

Supplementary Fig. 20 | Evolution of MAFS area density across frequencies, quantifying the phase transition dynamics. Statistical analysis of the area density of magnetic structural units at the moment of magnetic field application (initial state) and at dynamic equilibrium (final state) across a frequency spectrum from 60 to 140 Hz. The data reveal a pronounced reduction in unit density precisely at the critical frequency (γ = 3, B_z = 5 mT), providing direct evidence for the occurrence of high-frequency collision-fusion events at this threshold, which drives the MAFS growth. Scale bar, 2 mm. Source data for Supplementary Fig. 20 are provided as a Source Data file.

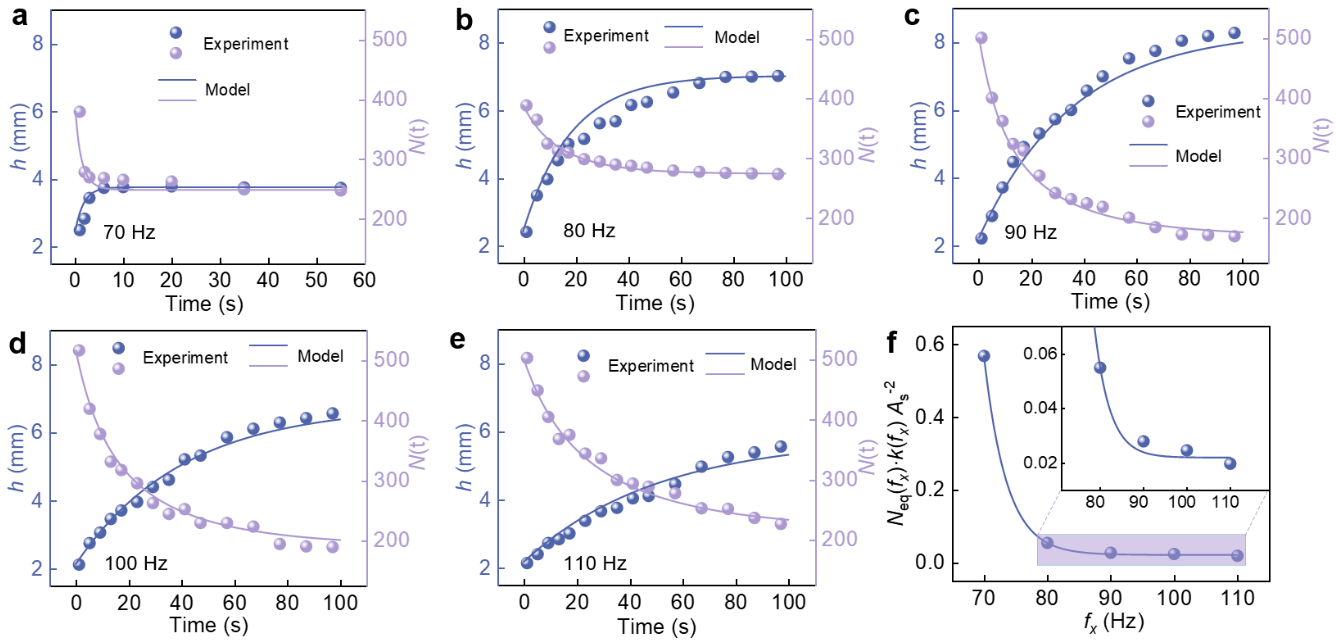

Supplementary Fig. 21 | Modeling and experimental analysis of frequency-controlled MAFS elongation. a-e Comparison of experimentally measured vertical growth dynamics in MAFS with theoretical model predictions across the frequency range of 70–110 Hz. **f** Extracted scaling parameters from the frequency-dependent growth model, demonstrating exponential relationships. Source data for Supplementary Fig. 21 are provided as a Source Data file.

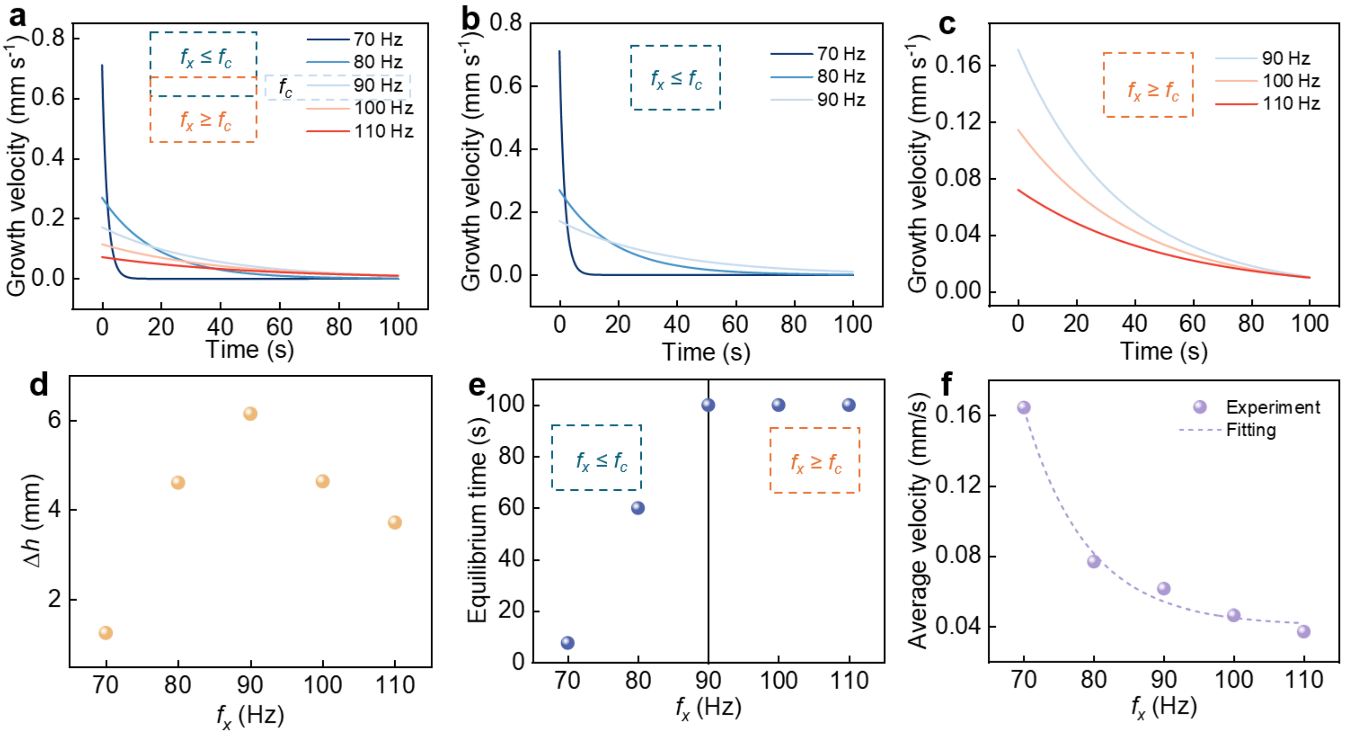

Supplementary Fig. 22 | Frequency-dependent vertical growth dynamics in MAFS. a Time-resolved vertical growth velocity profiles under varying actuation frequencies. **b, c** Distinct growth regimes observed below (**b**) and above (**c**) the critical frequency (f_c = 90 Hz). **d** Frequency-dependent height disparity between steady-state equilibrium and initial transient response post field activation. **e** Characteristic timescale for achieving dynamic equilibrium across different frequencies. **f** Average growth velocity throughout the complete elongation process. Source data for Supplementary Fig. 22 are provided as a Source Data file.

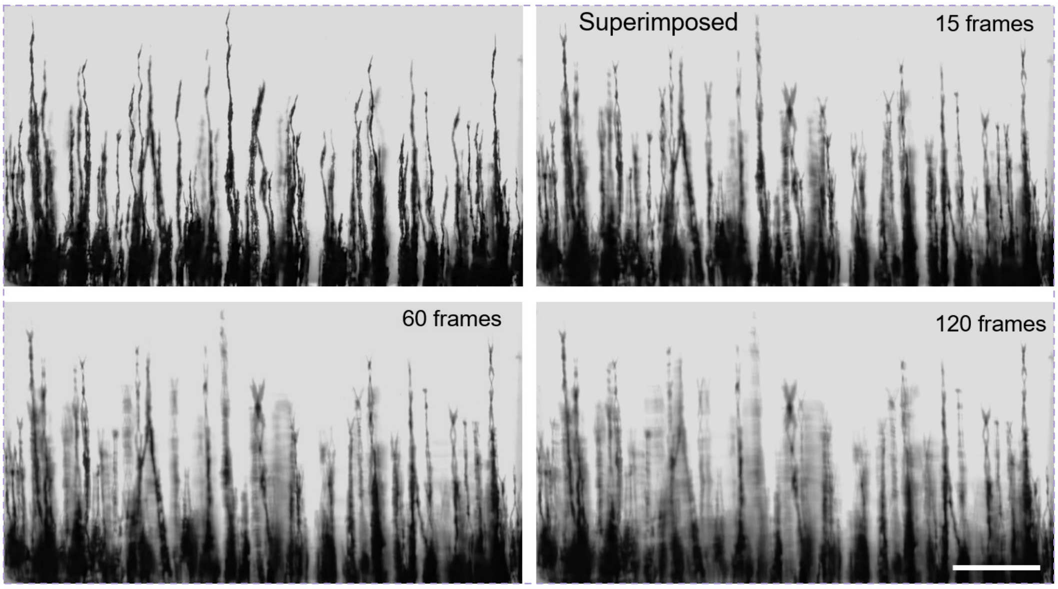

Supplementary Fig. 23 | Snapshots and superimposed images with varying frame counts (15, 60, and 120 frames; 60 fps) demonstrate the transient segmented structure of MAFS, the X-shaped vertical stacking pattern in dynamic visualization, and the resulting lateral drift. The magnetic particle concentration here is higher than in Fig. 4g. Scale bar, 2 mm.

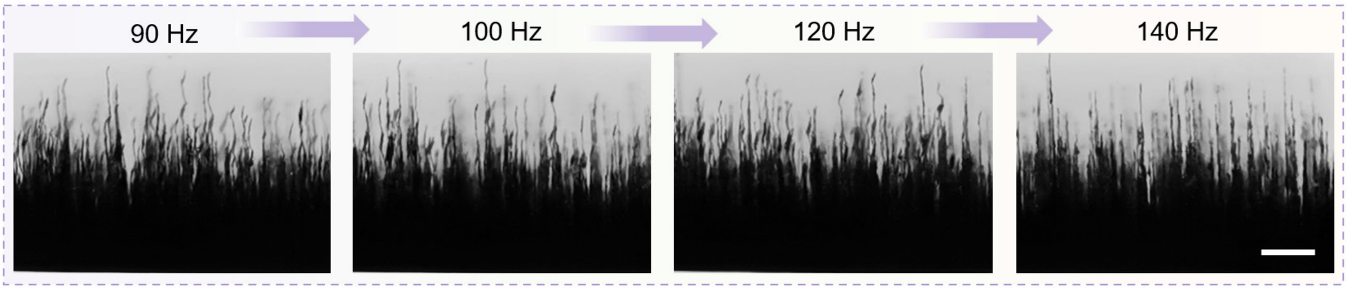

Supplementary Fig. 24 | Structural state transitions of MAFS during progressive frequency increases in the range above the critical frequency. The system maintains structural integrity without collapse under dynamic frequency switching, while exhibiting gradual magnetic configuration stabilization with increasing frequency. Scale bar, 2 mm.

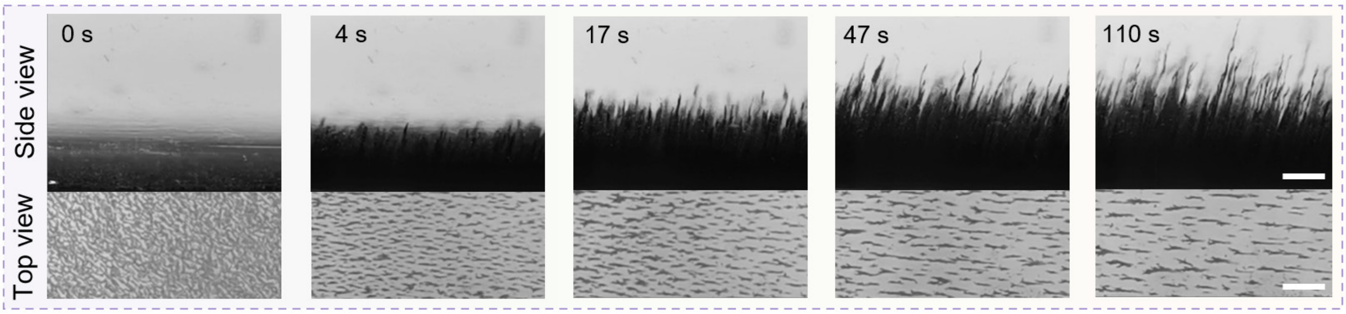

Supplementary Fig. 25 | Time-lapse sequence of the inclined extension dynamics of MAFS under applied magnetic fields with driving parameters B_{offset} = 1 mT, B_z = 5 mT, γ = 3, and f_x = 90 Hz. Scale bar, 2 mm.

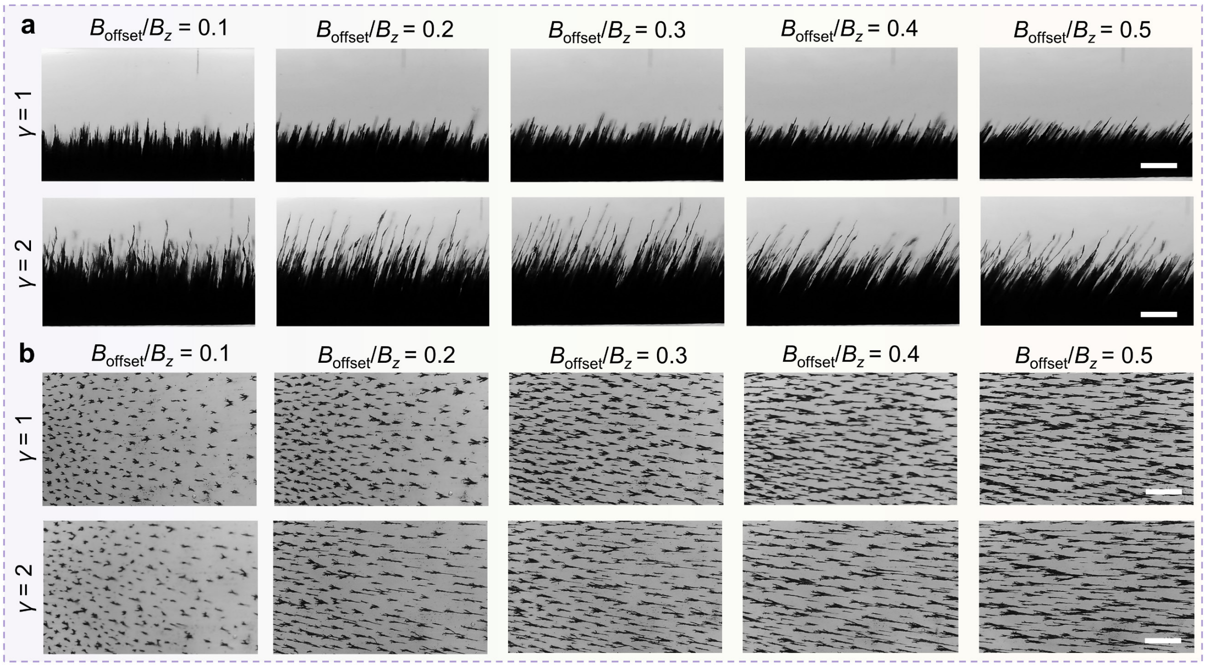

Supplementary Fig. 26 | Inclined extension of MAFS under varying field conditions. a,b Side-view and top-view images showing oblique growth patterns of MAFS at different B_{offset}/B_z ratios (0.1, 0.2, 0.3, 0.4, and 0.5) and amplitude ratios (γ = 1, 2). Scale bar, 2 mm.

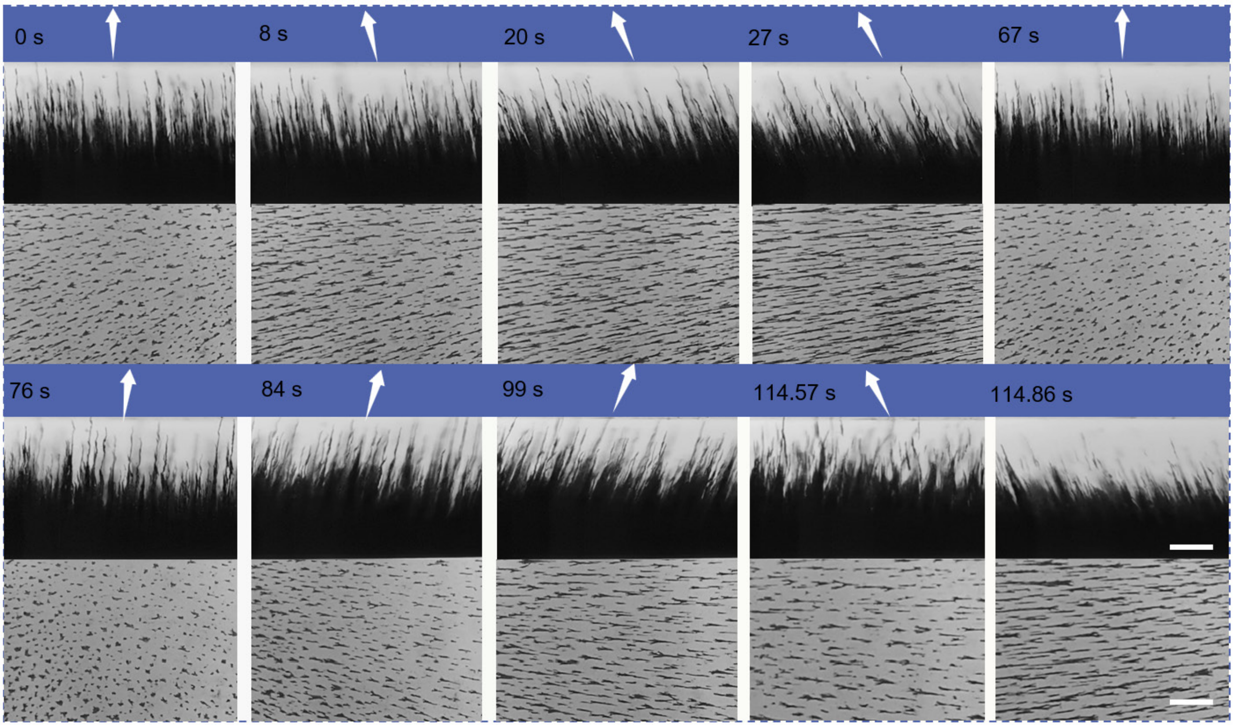

Supplementary Fig. 27 | Dynamic switching of inclination angles in MAFS under controlled dynamic equilibrium conditions (B_z = 5 mT, γ = 3, and f_x = 90 Hz). White arrows indicate the direction of inclination. During dynamic switching, the structure maintains relative stability; however, abrupt large-angle inclination transitions may cause detachment of the tip segment. Scale bar, 2 mm.

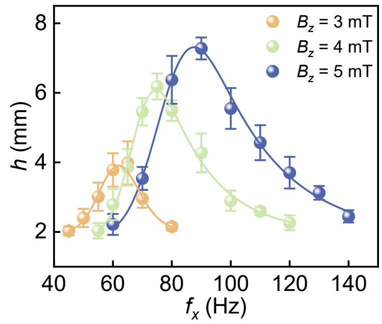

Supplementary Fig. 28 | Vertical growth capability of MAFS under different vertical magnetic field strengths B_z at amplitude ratio $\gamma = 3$. Data are presented as mean ± SD (n = 3 independent measurements). Source data for Supplementary Fig. 28 are provided as a Source Data file.

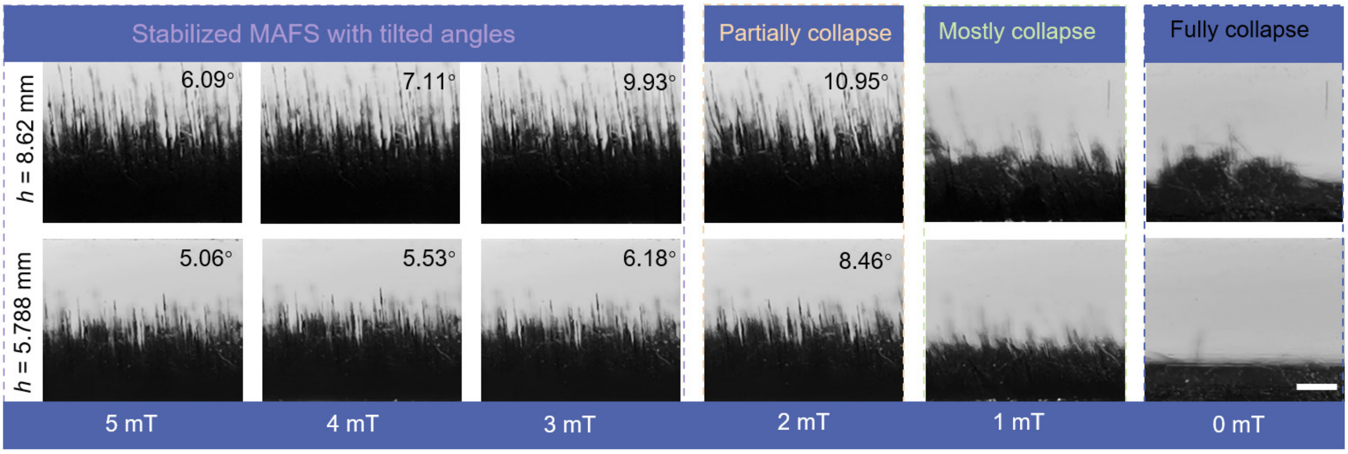

Supplementary Fig. 29 | Structural transitions of stabilized MAFS in response to gradually decreased B_z from 5 to 0 mT. The progressively weakening vertical magnetic field reduces interparticle magnetic dipole interactions, causing structural collapse as gravity gradually becomes dominant. Scale bar, 2 mm.

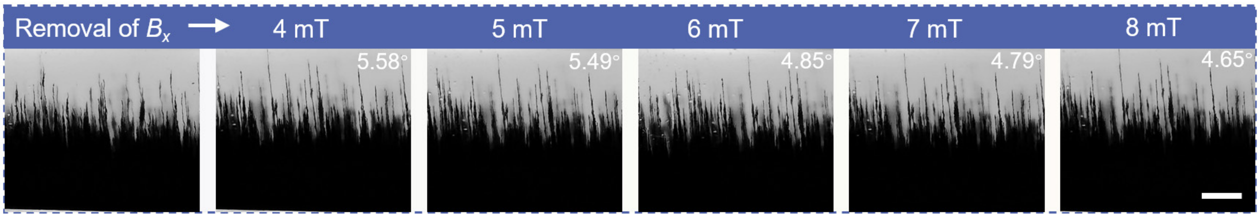

Supplementary Fig. 30 | Structural transitions of stabilized MAFS in response to gradually increased B_z from 4 to 8 mT. Scale bar, 2 mm.

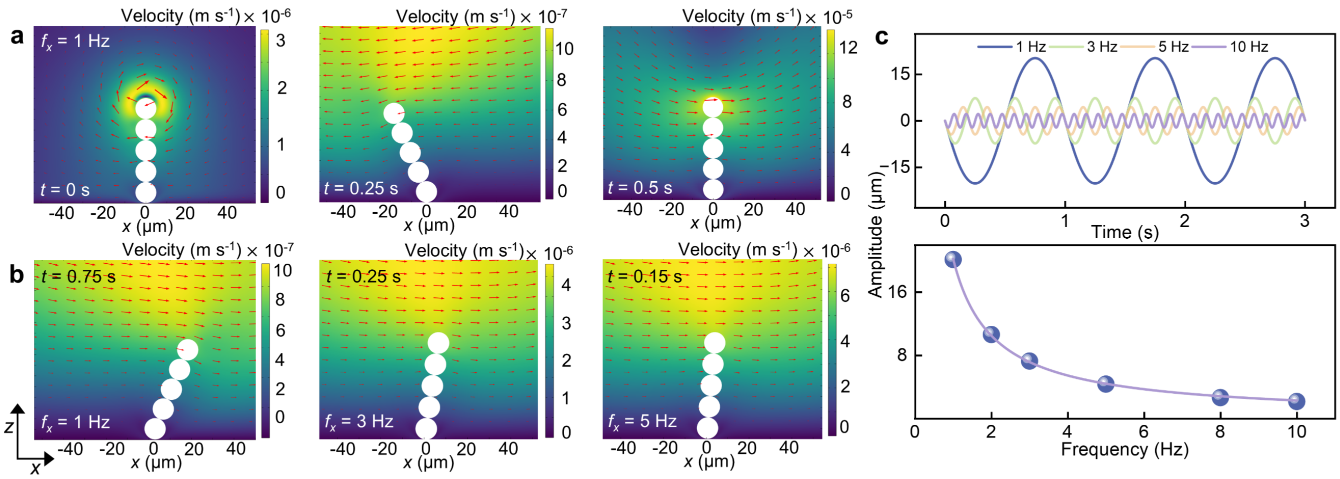

Supplementary Fig. 31 | Numerical simulations of a five-particle magnetic chain under a vertical oscillating magnetic field, revealing frequency-dependent oscillation dynamics. a Time-resolved displacement profiles of the magnetic chain under a 1 Hz actuation frequency. **b** Flow field distribution and oscillation amplitude at $t = 3/4T$ for varying frequencies. The corresponding instants are $t = 0.75$ s (1 Hz), $t = 0.25$ s (3 Hz), and $t = 0.15$ s (5 Hz). **c** Quantitative correlation between oscillation frequency and resultant amplitude derived from the simulations in **a** and **b**. Source data for Supplementary Fig. 31 are provided as a Source Data file.

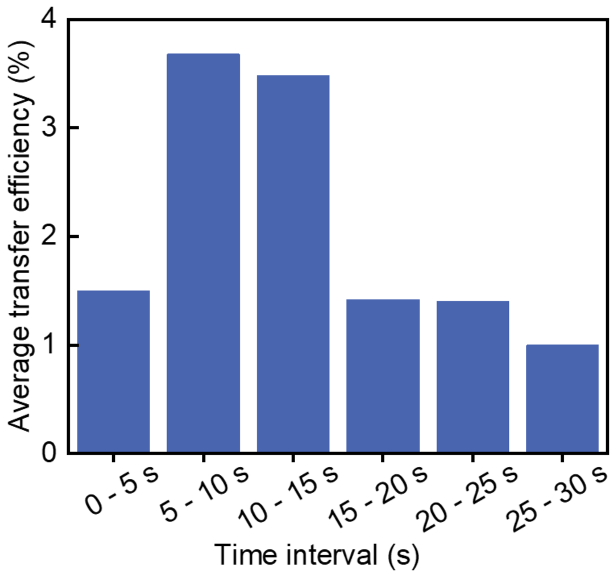

Supplementary Fig. 32 | Time-dependent interfacial mass transfer rates over 30 seconds, segmented into 5-second intervals. Source data for Supplementary Fig. 32 are provided as a Source Data file.

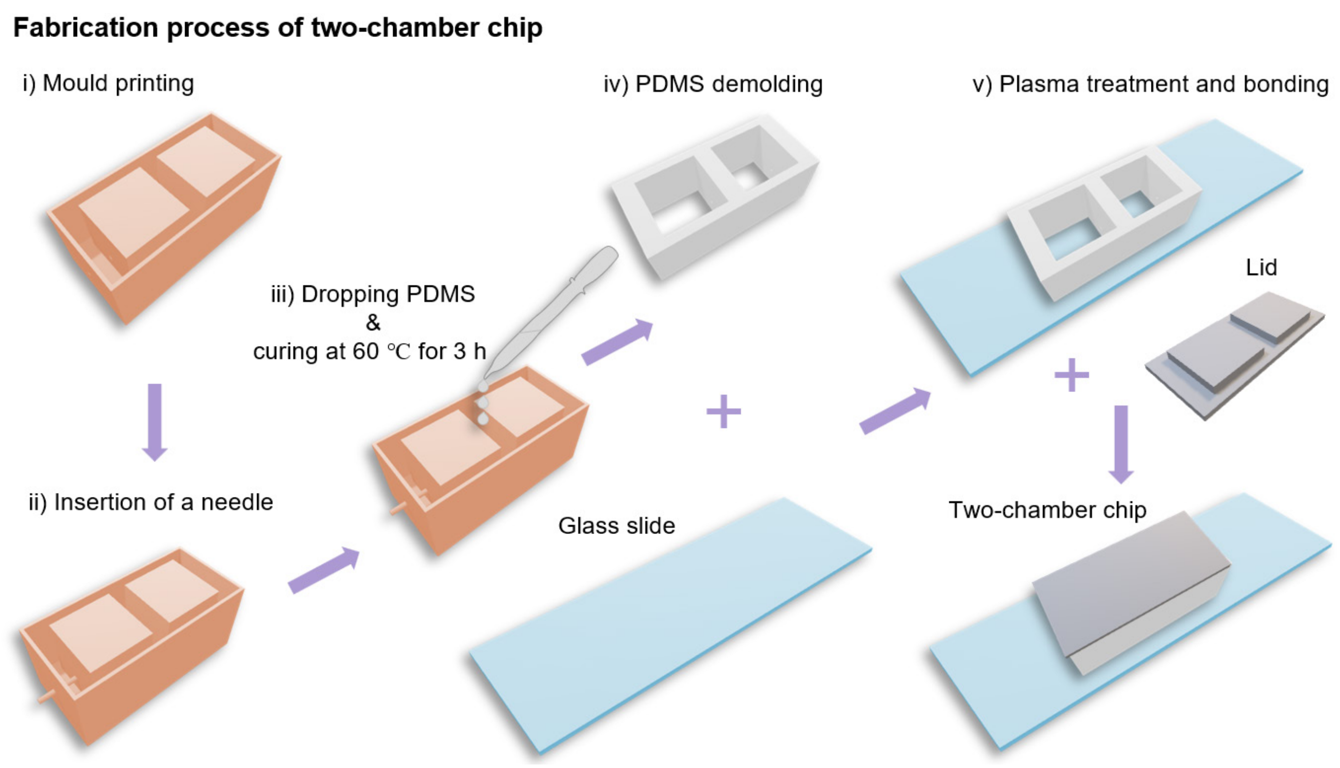

Supplementary Fig. 33 | Schematic illustration of the fabrication process for the two-chamber microfluidic chip. A master mold with channels (0.8 mm diameter) was designed and 3D-printed. Stainless steel needles were inserted into the mold to form inlet/outlet channels prior to PDMS casting. After PDMS pouring and curing at 60 °C for 3 hours, the PDMS replica was peeled off and bonded to a glass slide via oxygen plasma treatment. A 3D-printed lid was then assembled onto the PDMS slab to form the final enclosed two-chamber chip.

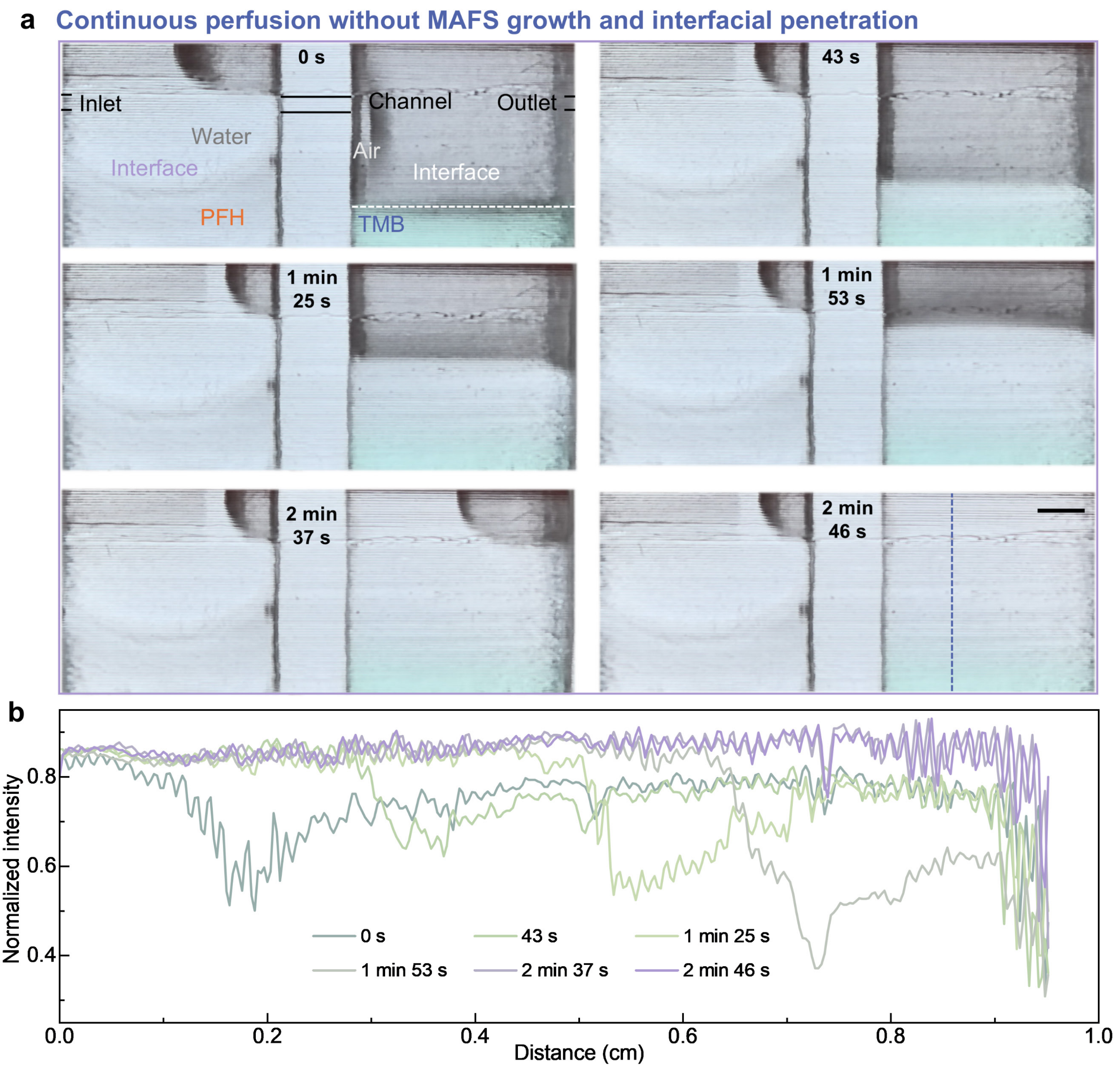

Supplementary Fig. 34 | Continuous perfusion without MAFS growth and interfacial penetration. a Time-lapse images of the right chamber containing TMB substrate under continuous perfusion at a flow rate of 500 μL min^{-1}, in the absence of MAFS growth and interfacial penetration. The TMB solution gradually lightens over time due to dilution, with no colorimetric reaction observed. Scale bar, 2 mm. **b** Quantitative color intensity profiles along the vertical dashed line (purple) in **a** at corresponding time points, showing the progressive dilution of the TMB substrate under continuous perfusion without enzymatic reaction. Source data for Supplementary Fig. 34 are provided as a Source Data file.

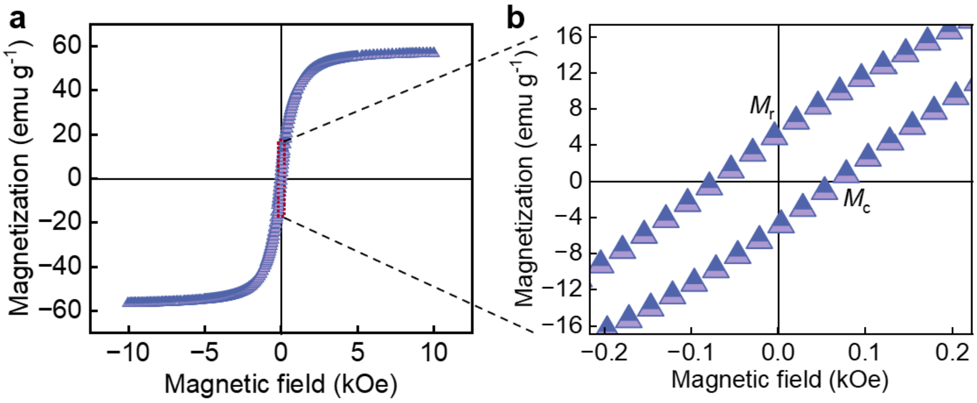

Supplementary Fig. 35 | Hysteresis loop of magnetic microparticles used for MAFS formation. a Hysteresis loop of the magnetic microparticles employed in this study. **b** Enlarged view of the hysteresis loop region highlighted in **a**. Source data for Supplementary Fig. 35 are provided as a Source Data file.

Supplementary Table

Supplementary Table 1. Parameters of the designed electromagnetic coils.

Axis	Coil diameter (mm)	Inner diameter (mm)	Outer diameter (mm)	Num. of turns	Thickness (mm)	Resistance (Ω)
x	1.6	172	252	688	45	4.2
y	1	106	152	432	20	4.2
z	1	55	84	240	17	1.4

Supplementary References

1. Rosensweig, R.E. Heating magnetic fluid with alternating magnetic field. *J. Magn. Magn. Mater.* **252**, 370-374 (2002).
2. Wardzynska, R., Załęska-Chróst B. Computer simulation of the coagulation of suspended solids — the applicability of the müller–smoluchowski theory. *J. Environ. Sci.* **44**, 197-203 (2016).
3. Chan, T.H.T., Ashebo D.B. Theoretical study of moving force identification on continuous bridges. *J. Sound. Vib.* **295**, 870-883 (2006).
4. Stancioiu, D., James S., Ouyang H., Mottershead J.E. Vibration of a continuous beam excited by a moving mass and experimental validation. *J. Phys. Conf. Ser.* **181**, 012084 (2009).
5. Law, J., Chen H., Wang Y., Yu J., Sun Y. Gravity-resisting colloidal collectives. *Sci. Adv.* **8**, eade3161 (2022).
