# Supplementary material for: Transport of enzymatic activity across liquid-liquid interfaces using dynamic assemblies of magnetic particles via field-modulated interactions
Source: Nat Commun. 2026 May 26;17:6872. doi: 10.1038/s41467-026-73696-8 (PMC13388707; doi:10.1038/s41467-026-73696-8)
Supplement: Supplementary file 2 — Description of Additional Supplementary Files [file 41467_2026_73696_MOESM2_ESM.pdf]

Inventory of Supporting Information

1. Supplementary Information

2. Supplementary Movies (1-15)

Supplementary Movie 1. Dynamic self-assembly and vertical growth of MAFS.

This video shows the vertical growth process of MAFS under the conditions of B_z = 5 mT, γ = 3, and f_x = 90 Hz. The sequence captures the initial formation of MAFS, their dynamic merging and subsequent growth, and the progression towards dynamic equilibrium.

Supplementary Movie 2. Three behavioral phases under tailored oscillating magnetic field across different frequency ranges.

This video showcases the distinct phases of magnetic structures under tailored oscillating magnetic fields at varying frequency ranges. It features swinging pillars observed in the low-frequency range, oscillating vertical collectives in the medium-frequency range, and the formation of MAFS in the high-frequency range.

Supplementary Movie 3. Pillar fragmentation and formation of uncontrolled, massive pillar-like patterns.

This video demonstrates that as the oscillating frequency increases, longer swinging pillars become uncontrolled, resulting in the formation of massive pillar-like patterns. This behavior is driven by the enhanced fluid drag at higher frequencies, which disrupts the stability of the pillars and leads to their fragmentation and aggregation into larger structures.

Supplementary Movie 4. Critical behavior at the transition between phase II and phase III.

This video illustrates the critical behavior that occurs when the oscillating frequency is just below the threshold required to induce the formation of MAFS. Under such conditions, adjacent magnetic pillars briefly ascend to merge with the main pillar but subsequently detach, leading to the interruption of vertical growth.

Supplementary Movie 5. Collapse of MAFS and falling of magnetic particles upon removal of

magnetic field.

This video demonstrates that MAFS in dynamic equilibrium collapse instantaneously when the magnetic field is removed, highlighting the crucial role of gravity in maintaining the stability of the entire system.

Supplementary Movie 6. Frequency-dependent tilt angle of magnetic pillar and X-shaped vertical stacking in dynamic visualization.

This video presents both experimental and simulated particle assemblies at varying frequencies (30, 50, 70, and 90 Hz), demonstrating strong agreement between theoretical predictions and experimental results. The dynamic visualization clearly reveals the characteristic X-shaped vertical stacking pattern and the resulting lateral drift phenomenon.

Supplementary Movie 7. Controlled reversible growth of MAFS.

This video demonstrates the controlled reversible growth of MAFS in dynamic equilibrium by modulating the oscillating magnetic field frequency from 90 to 70 Hz and then back to 90 Hz. The modulation induces the descent and subsequent regrowth of the MAFS, showcasing the system's ability to dynamically and reversibly regulate growth.

Supplementary Movie 8. Inclined growth of MAFS.

This video shows that under an oscillating magnetic field with an offset of B_{offset} = 1 mT (B_{offset}/B_z = 0.2, B_z = 5 mT, γ = 3, f_x = 90 Hz), dispersed magnetic particles dynamically self-assemble into tilted MAFS, achieving dynamic equilibrium.

Supplementary Movie 9. Dynamic switching of inclined angles in MAFS while maintaining dynamic equilibrium.

This video demonstrates that MAFS can be dynamically oriented leftward or rightward by adjusting the offset of the oscillating magnetic fields (B_{offset}/B_z ranging from 0.1 to 0.5), simultaneously maintaining dynamic equilibrium.

Supplementary Movie 10. Actuation of low-height pillars and dynamic actuation of stabilized MAFS.

This video demonstrates the actuation of magnetic structures without undergoing a vertical growth process, the stabilization of MAFS after vertical growth, and the subsequent dynamic actuation of these self-standing structures. These observations underscore the essential role of the vertical growth process in enabling the actuation of ultra-high magnetic structures.

Supplementary Movie 11. Dynamic actuation of stabilized MAFS at various actuating amplitudes and frequencies.

This video illustrates the structural transitions of stabilized MAFS actuated under different magnetic field amplitudes (1 and 2 mT) and oscillation frequencies (1, 2, 3, and 5 Hz).

Supplementary Movie 12. Interface-mediated structural transfer of MAFS across immiscible phases.

This video captures the interfacial crossing process of MAFS, beginning with their vertical growth and upward approach toward the interface. Upon contact, two distinct penetration behaviors emerge: either the structure's tip breaks apart, dispersing individual particles, or the entire structure is uprooted, inducing localized interfacial fragmentation. Successfully penetrating structures then undergo continuous upward transport, transitioning from the PFH phase into the aqueous phase. This dynamic sequence highlights the complex interplay between interfacial forces and structural stability during phase boundary crossing.

Supplementary Movie 13. Upward migration of magnetic structures continued despite the change in frequency.

This video demonstrates that reducing the magnetic field frequency resulted in a uniform interfacial distribution of the assembled structures. At the same time, even under these conditions, residual magnetic units persistently migrated upward, propelled by a long-range attractive force originating from the high-density accumulation of interfacial particles.

Supplementary Movie 14. Triggered localized chromogenic reaction upon interfacial penetration of MAFS by using HRP-modified particles and an H_2O_2/ABTS upper phase.

This video captures the interfacial penetration of MAFS and the immediate triggering of a localized chromogenic reaction, facilitated by HRP-modified particles in an H_2O_2/ABTS upper phase.

Supplementary Movie 15. Controlled enzymatic reactions in a two-chamber microfluidic chip using MAFS.

This video shows the evolution of TMB color development in the right chamber. In the left chamber, MAFS growth and interfacial penetration deliver HRP into the upper aqueous phase. Initially, no color change is observed under continuous perfusion. After a temporary halt in perfusion to allow enzyme accumulation, resumed perfusion triggers progressive color development.

3. Supplementary Data (1-2)

Supplementary Data 1: Unprocessed raw experimental images used to extract quantitative information presented in the manuscript, such as the height, base distance, and inclined angle of magnetically assembled filamentary structures (MAFS). These images underlie the measured data shown in the figures.

Supplementary Data 2: Custom MATLAB code for simulating the field-driven dynamic self-assembly and gravity-resisting vertical growth of magnetic microparticles. The model incorporates magnetic dipole–dipole interactions with Debye relaxation, gravitational and buoyancy forces, near-field repulsion, and hydrodynamic interactions via the Rotne–Prager–Yamakawa tensor with wall corrections. The code outputs time-resolved 3D, front-view, and top-view videos, as well as periodic keyframe snapshots.
