# Supplementary material for: Transport of enzymatic activity across liquid-liquid interfaces using dynamic assemblies of magnetic particles via field-modulated interactions
Source: Nat Commun. 2026 May 26;17:6872. doi: 10.1038/s41467-026-73696-8 (PMC13388707; doi:10.1038/s41467-026-73696-8)

Fig. 2

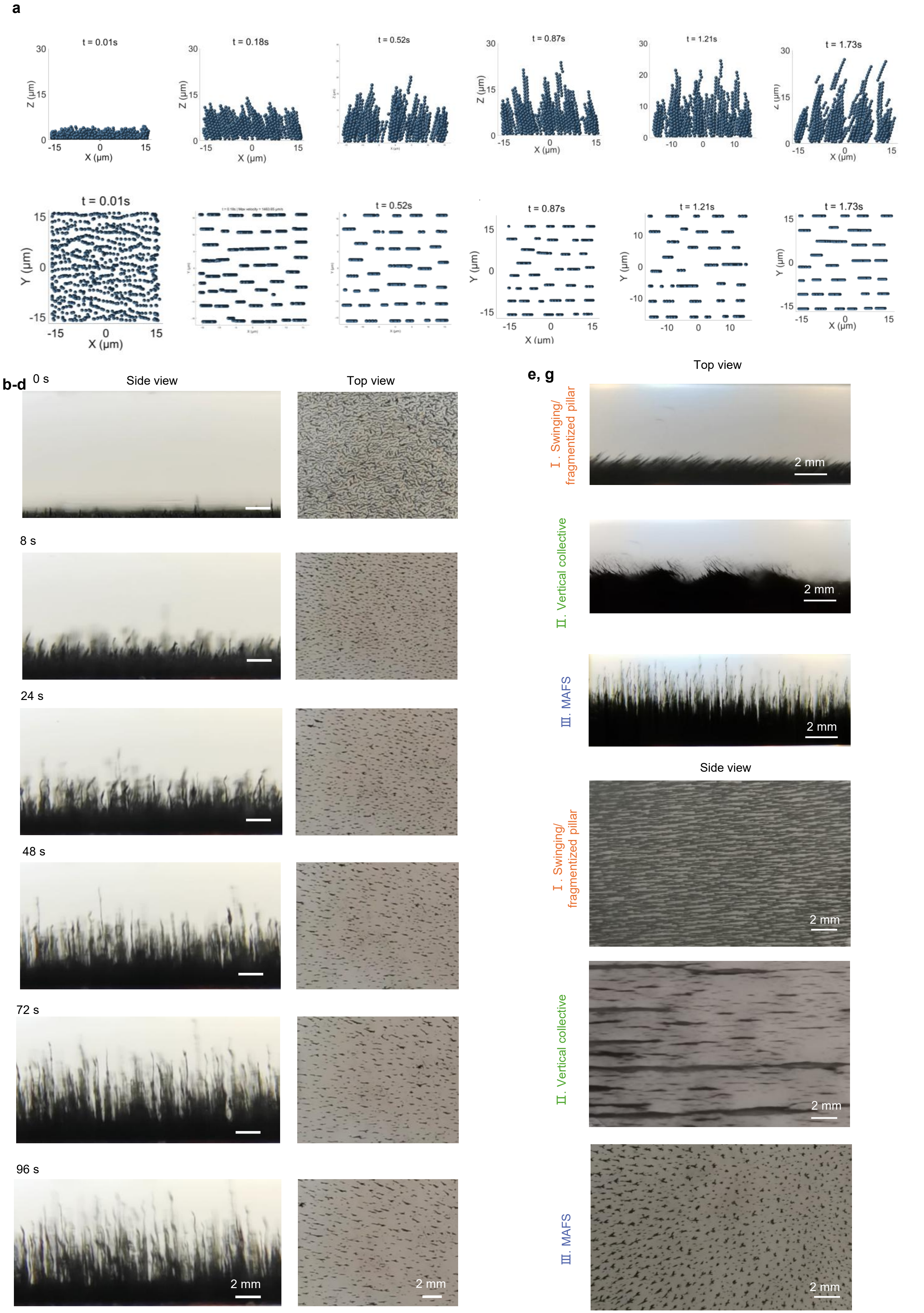

a
t = 0.01s
t = 0.18s
t = 0.52s
t = 0.87s
t = 1.21s
t = 1.73s
Z (μm)
X (μm)
Y (μm)
b-d
0 s
8 s
24 s
48 s
72 s
96 s
Side view
Top view
2 mm
e, g
Top view
Side view
Ⅰ. Swinging/ fragmentized pillar
Ⅱ. Vertical collective
Ⅲ. MAFS
2 mm

Fig. 3

b

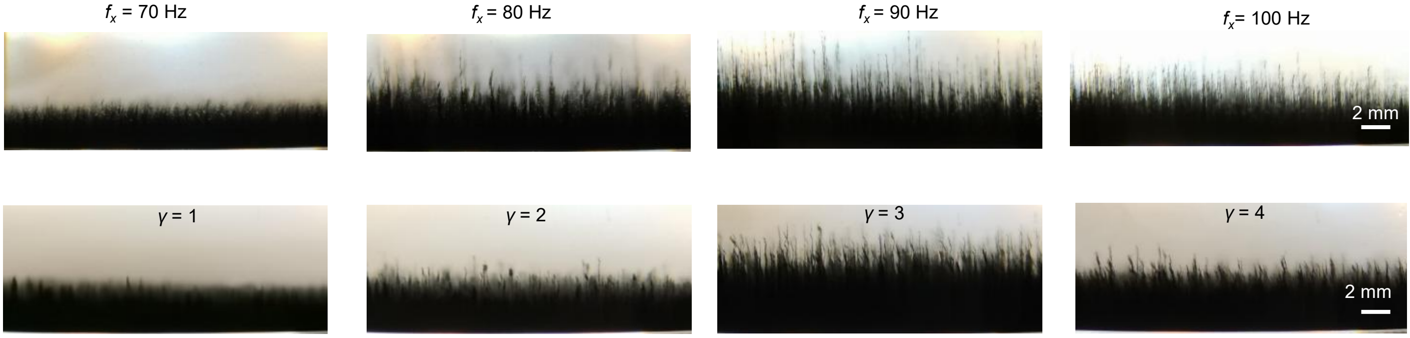

i, j, k, l, Supplementary Fig. 10, 11

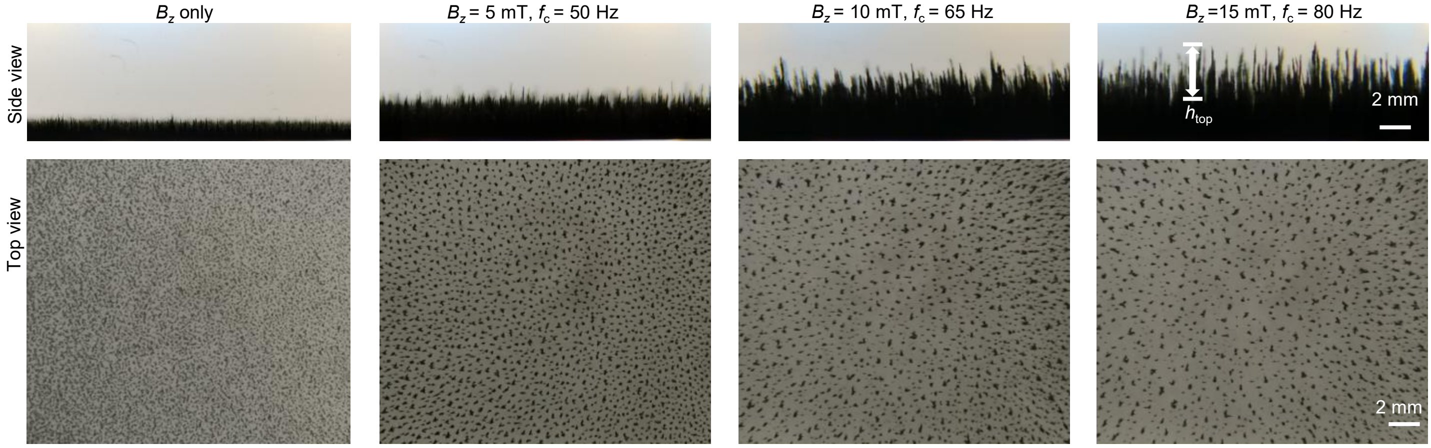

j

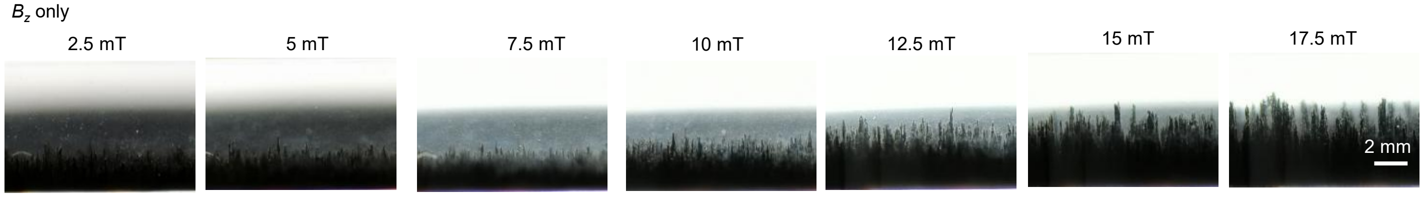

n

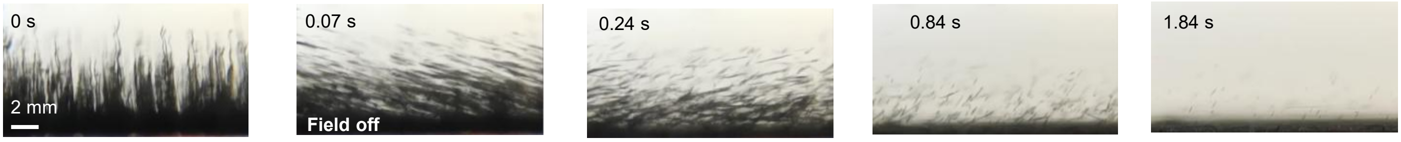

Fig. 3

c

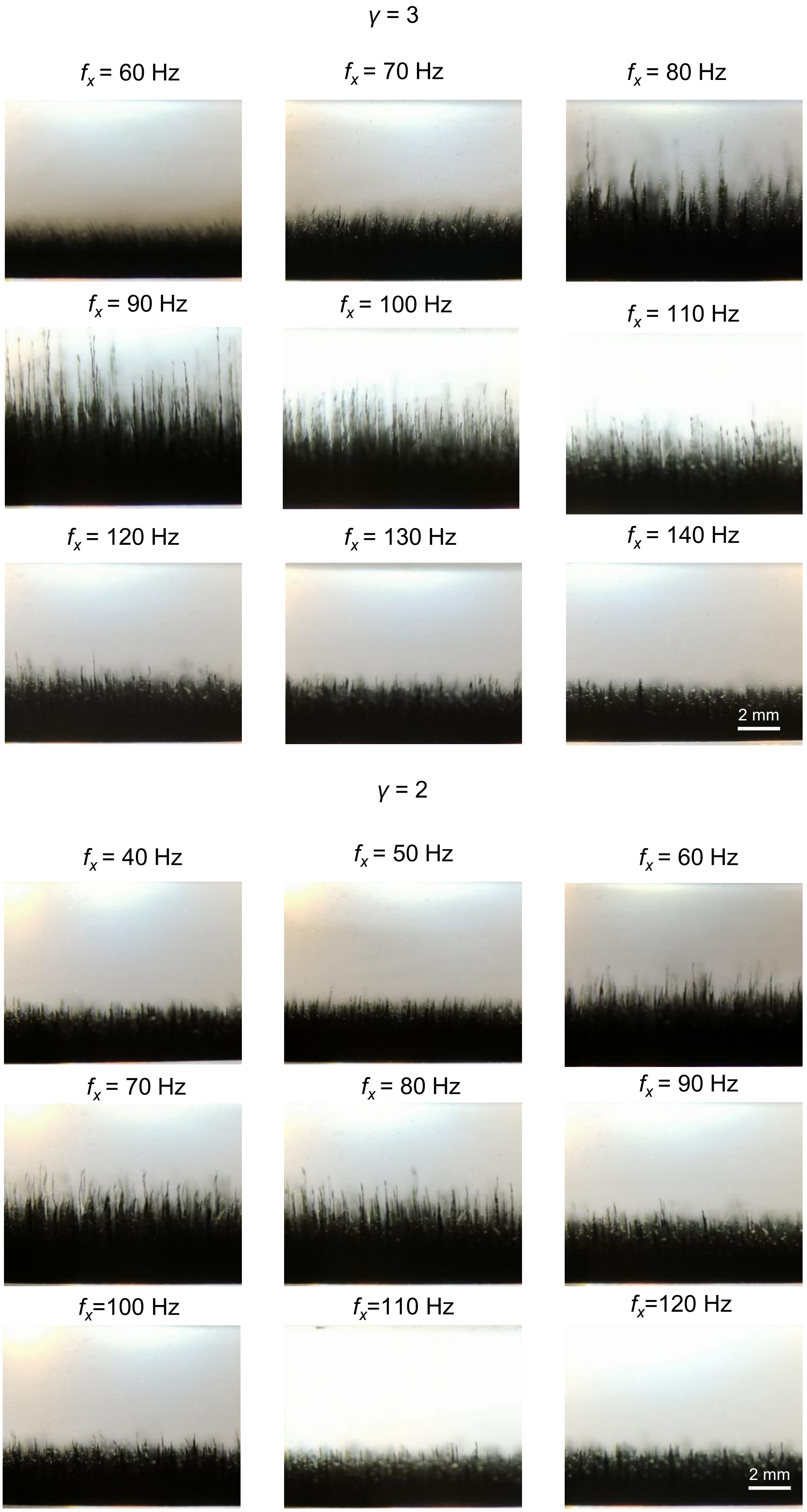

γ = 3
f_x = 60 Hz
f_x = 70 Hz
f_x = 80 Hz
f_x = 90 Hz
f_x = 100 Hz
f_x = 110 Hz
f_x = 120 Hz
f_x = 130 Hz
f_x = 140 Hz
2 mm
γ = 2
f_x = 40 Hz
f_x = 50 Hz
f_x = 60 Hz
f_x = 70 Hz
f_x = 80 Hz
f_x = 90 Hz
f_x=100 Hz
f_x=110 Hz
f_x=120 Hz
2 mm

Fig. 3

c

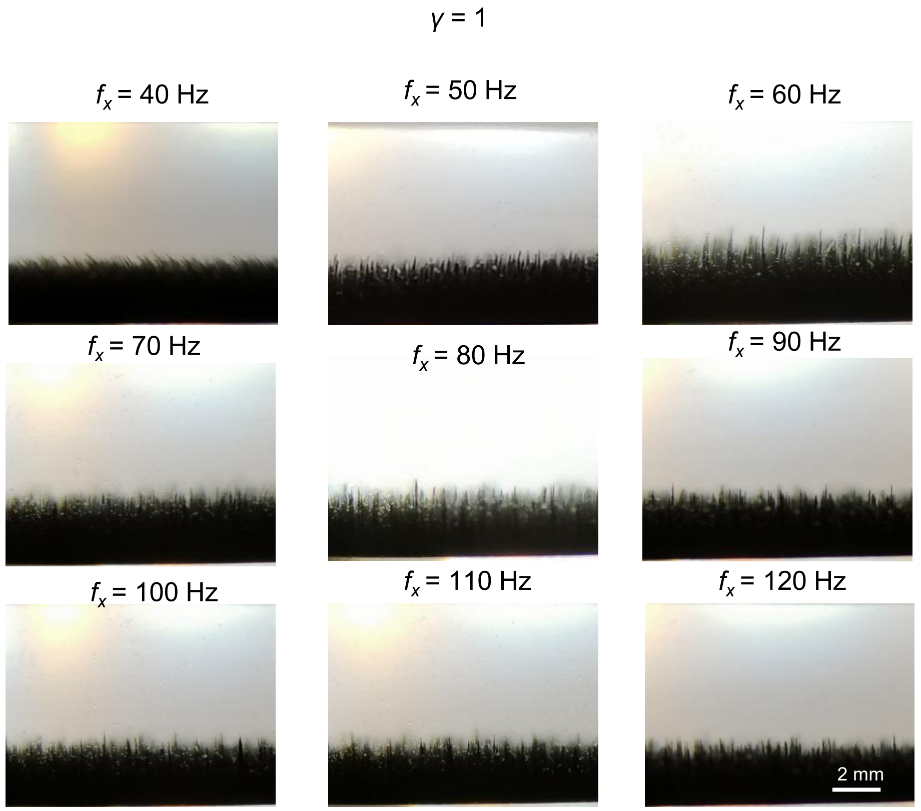

γ = 1
f_x = 40 Hz
f_x = 50 Hz
f_x = 60 Hz
f_x = 70 Hz
f_x = 80 Hz
f_x = 90 Hz
f_x = 100 Hz
f_x = 110 Hz
f_x = 120 Hz
2 mm

m

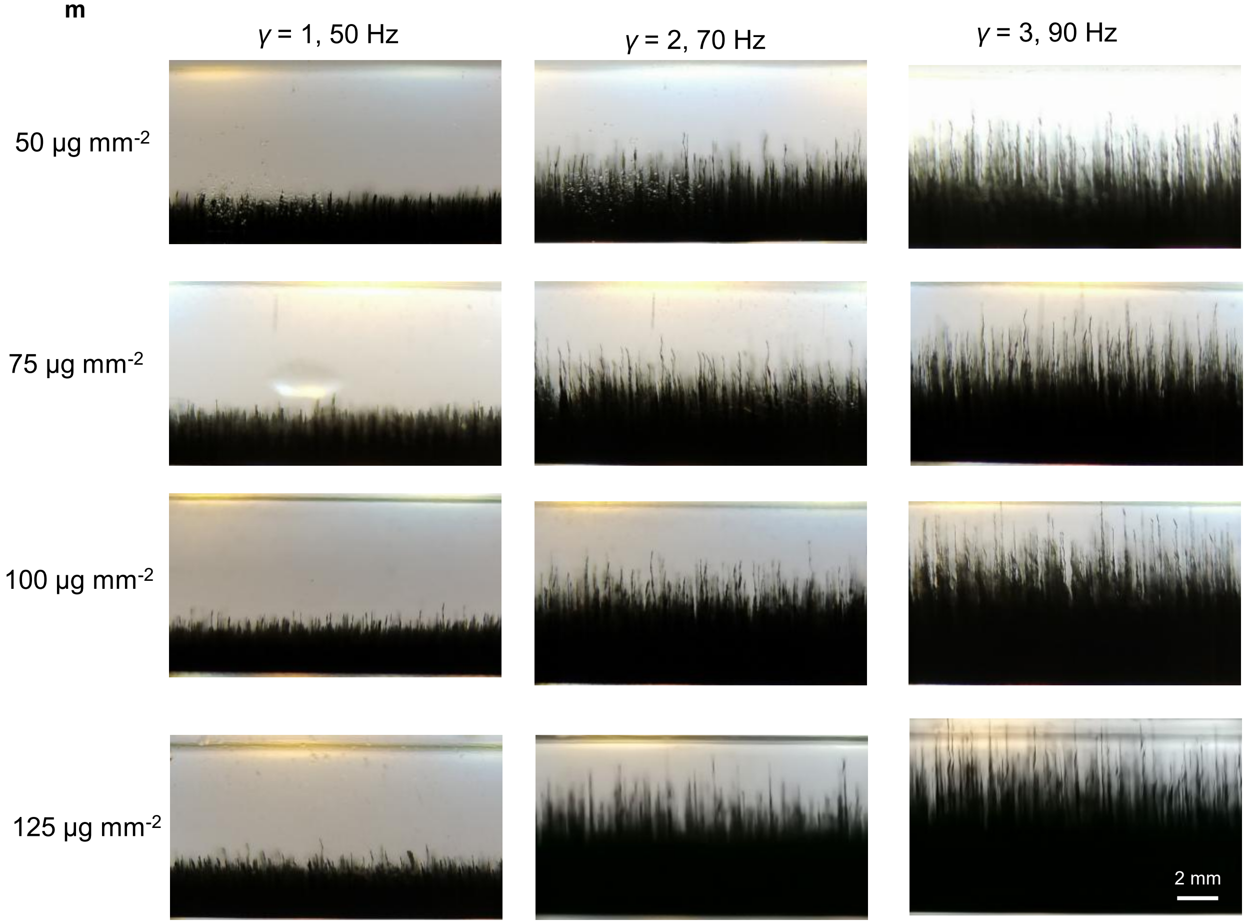

γ = 1, 50 Hz
γ = 2, 70 Hz
γ = 3, 90 Hz
50 μg mm^{-2}
75 μg mm^{-2}
100 μg mm^{-2}
125 μg mm^{-2}
2 mm

Fig. 3 **Supplementary Fig. 9**

d, e,

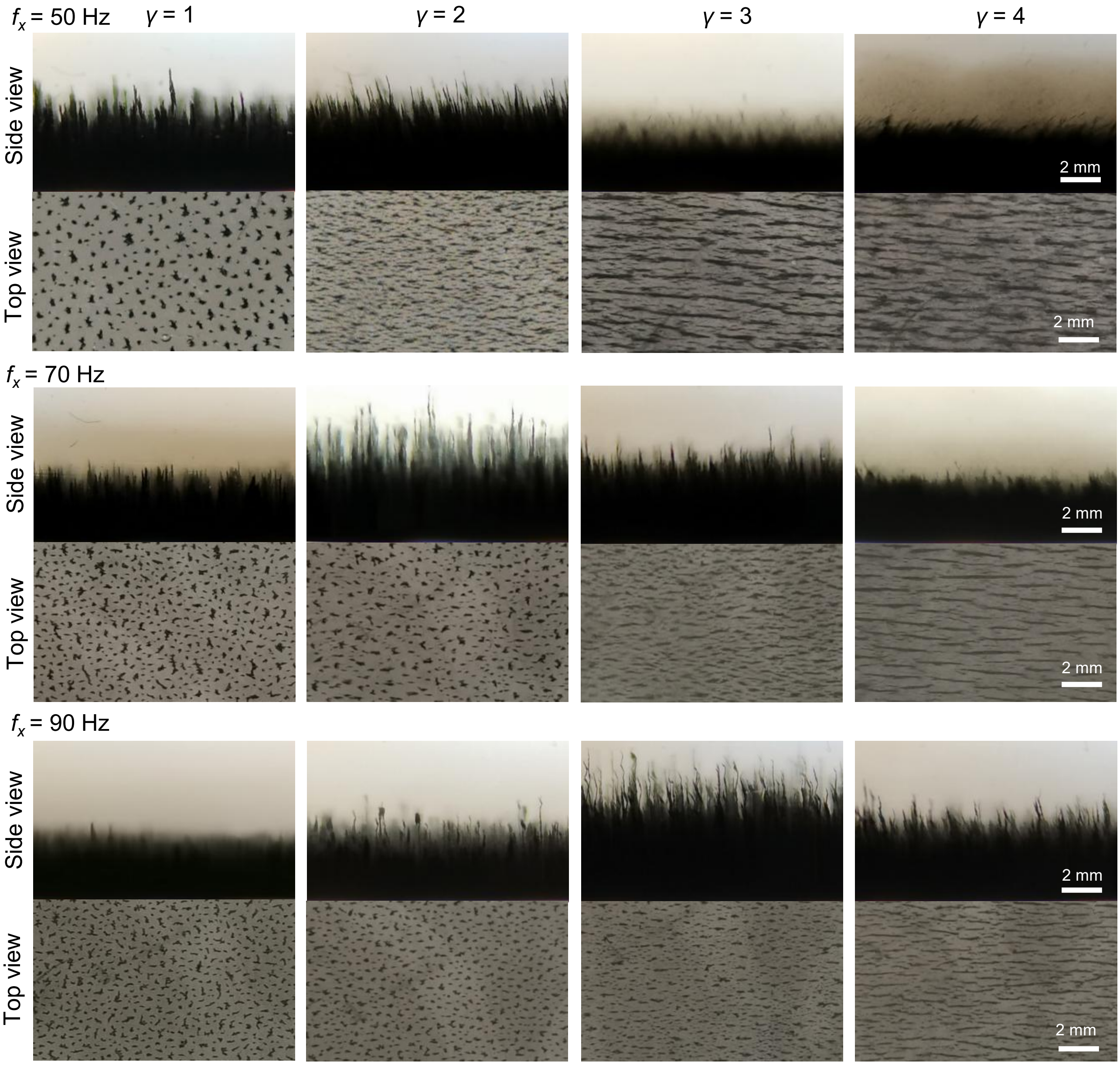

Fig. 4

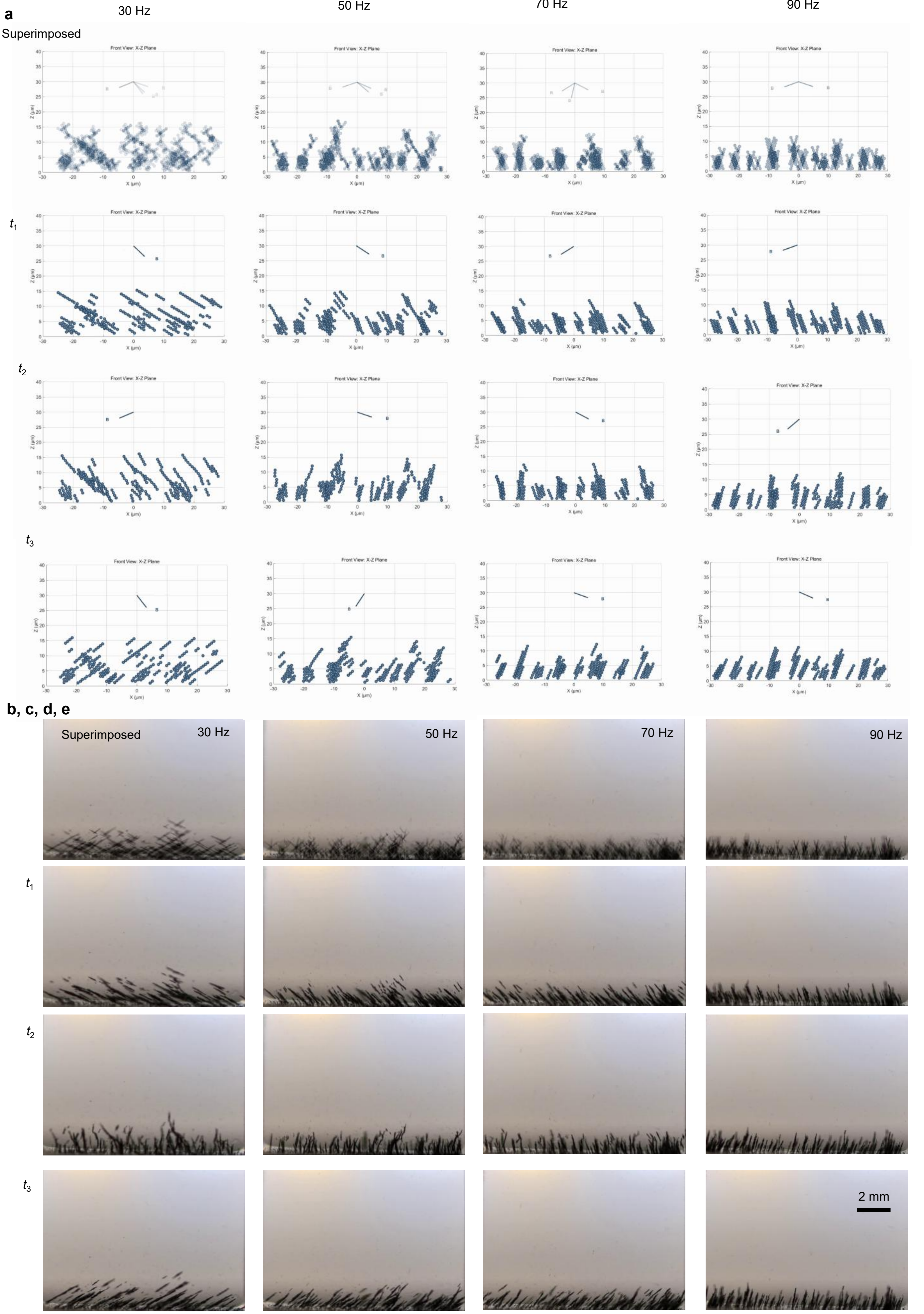
a
30 Hz
50 Hz
70 Hz
90 Hz
Superimposed
Front View: X-Z Plane
Z (μm)
X (μm)
t_1
t_2
t_3
b, c, d, e
Superimposed
30 Hz
50 Hz
70 Hz
90 Hz
t_1
t_2
t_3
2 mm

Fig. 4

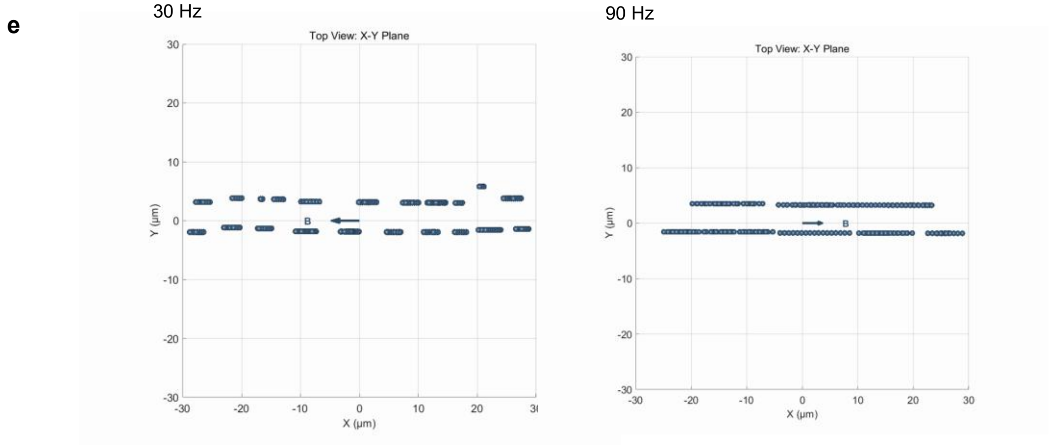

e
30 Hz
Top View: X-Y Plane
Y (μm)
X (μm)
B
90 Hz
Top View: X-Y Plane
Y (μm)
X (μm)
B

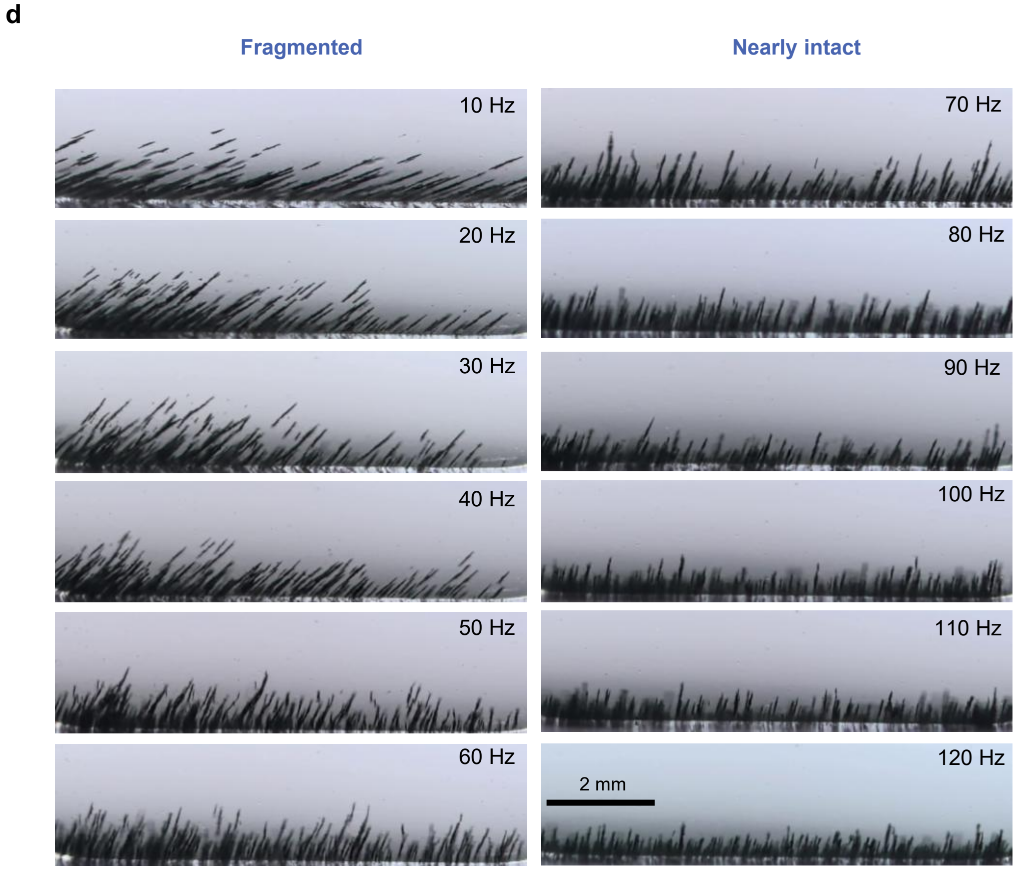

d
Fragmented
Nearly intact
10 Hz
20 Hz
30 Hz
40 Hz
50 Hz
60 Hz
70 Hz
80 Hz
90 Hz
100 Hz
110 Hz
120 Hz
2 mm

Fig. 5

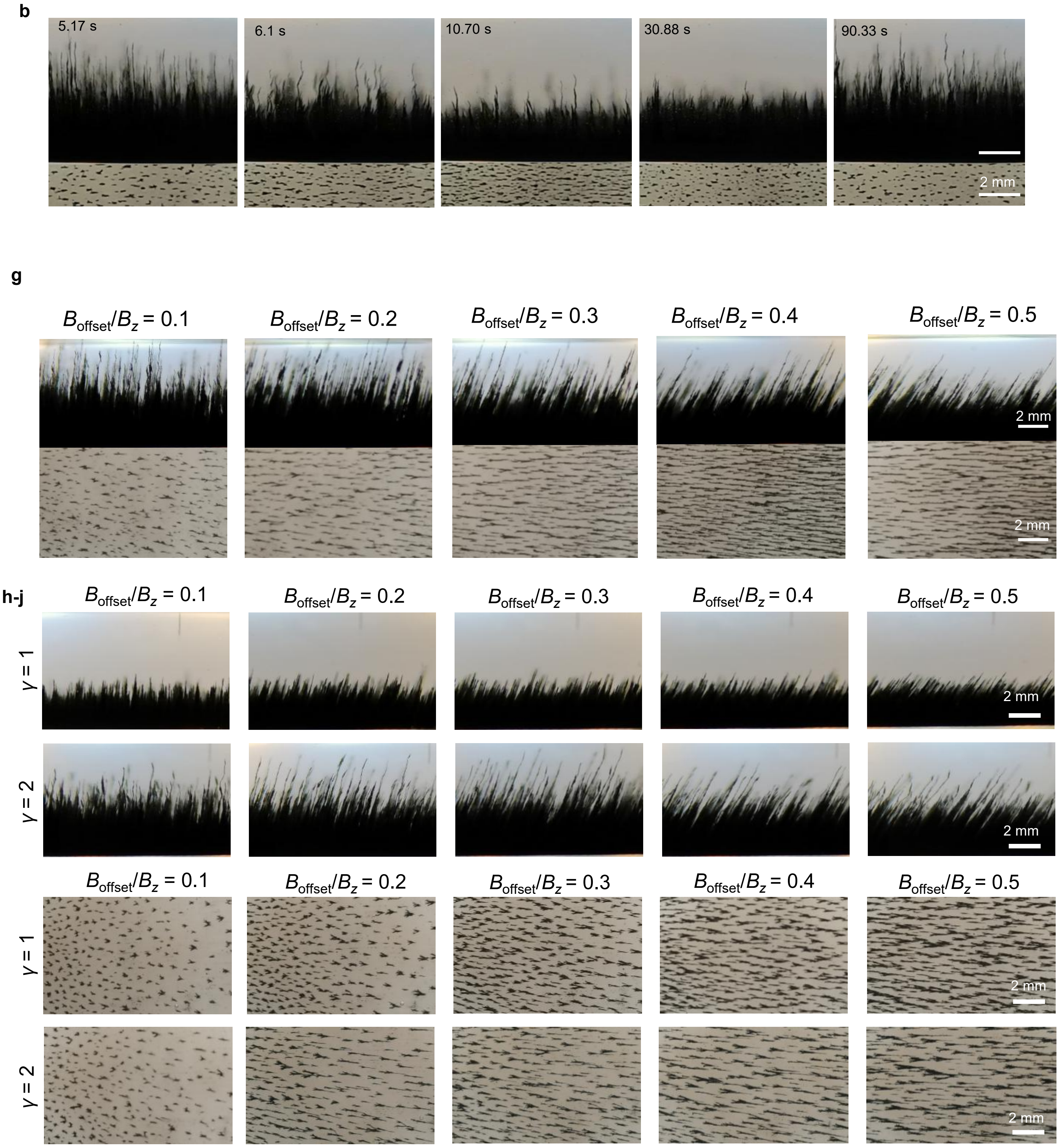
b
5.17 s
6.1 s
10.70 s
30.88 s
90.33 s
2 mm
g
B_{offset}/B_z = 0.1
B_{offset}/B_z = 0.2
B_{offset}/B_z = 0.3
B_{offset}/B_z = 0.4
B_{offset}/B_z = 0.5
2 mm
2 mm
h-j
B_{offset}/B_z = 0.1
B_{offset}/B_z = 0.2
B_{offset}/B_z = 0.3
B_{offset}/B_z = 0.4
B_{offset}/B_z = 0.5
γ = 1
γ = 2
2 mm
2 mm
B_{offset}/B_z = 0.1
B_{offset}/B_z = 0.2
B_{offset}/B_z = 0.3
B_{offset}/B_z = 0.4
B_{offset}/B_z = 0.5
γ = 1
γ = 2
2 mm
2 mm

Fig. 6

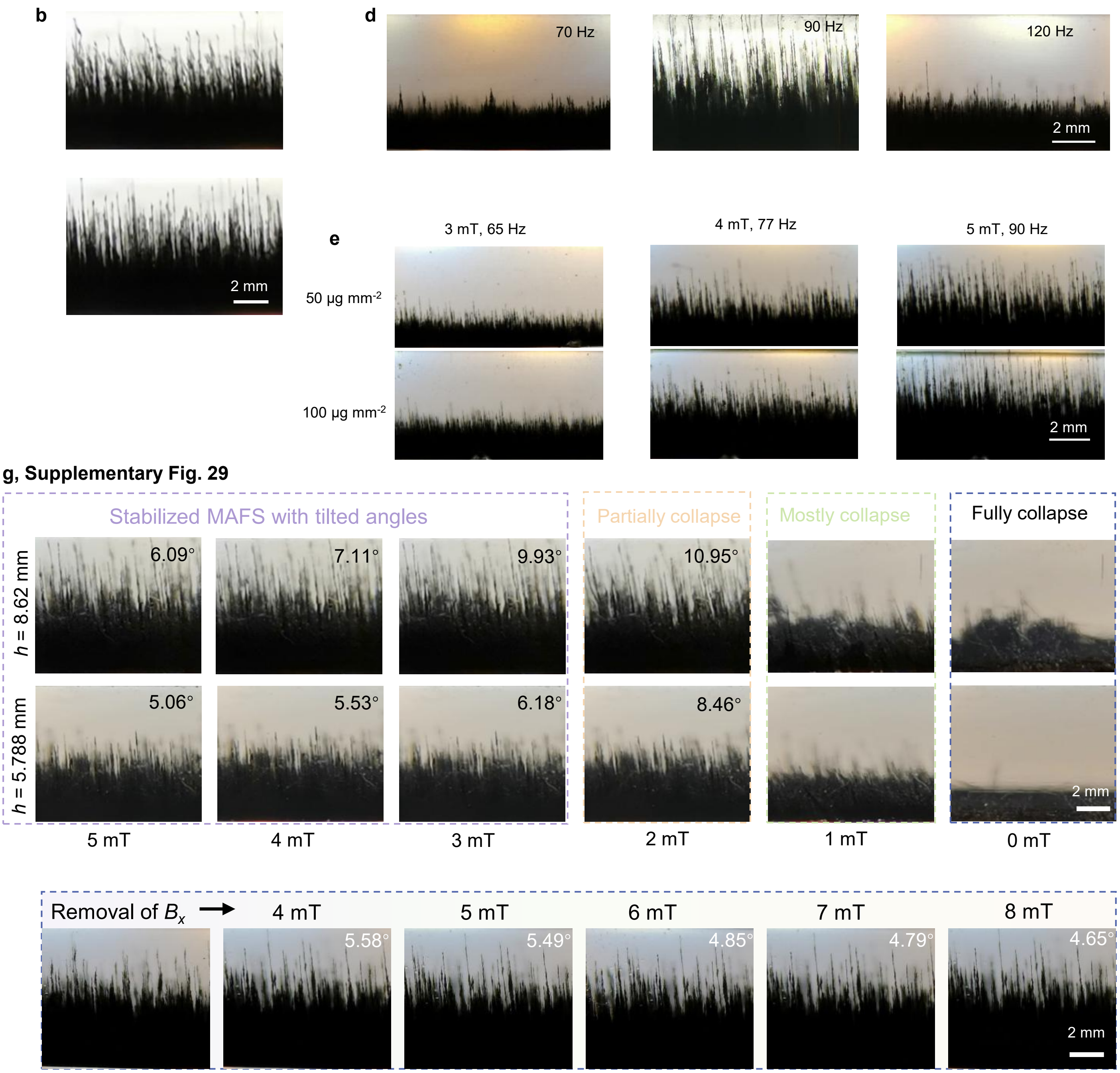
b
2 mm
d
70 Hz
90 Hz
120 Hz
2 mm
e
3 mT, 65 Hz
4 mT, 77 Hz
5 mT, 90 Hz
50 µg mm-2
100 µg mm-2
2 mm
g, Supplementary Fig. 29
Stabilized MAFS with tilted angles
Partially collapse
Mostly collapse
Fully collapse
h = 8.62 mm
6.09°
7.11°
9.93°
10.95°
h = 5.788 mm
5.06°
5.53°
6.18°
8.46°
2 mm
5 mT
4 mT
3 mT
2 mT
1 mT
0 mT
Removal of Bx
4 mT
5 mT
6 mT
7 mT
8 mT
5.58°
5.49°
4.85°
4.79°
4.65°
2 mm

Supplementary Fig. 12

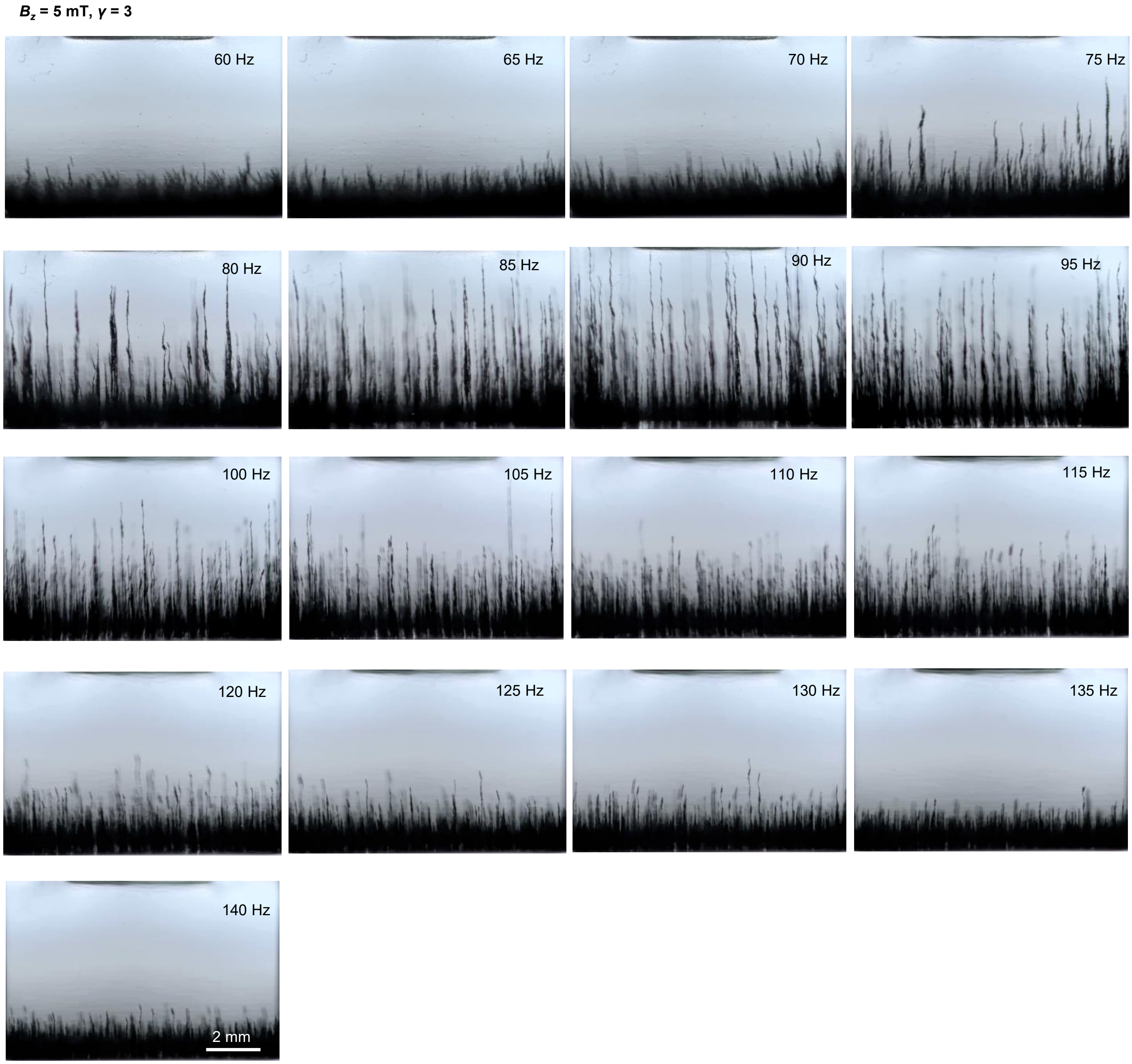

Supplementary Fig. 12

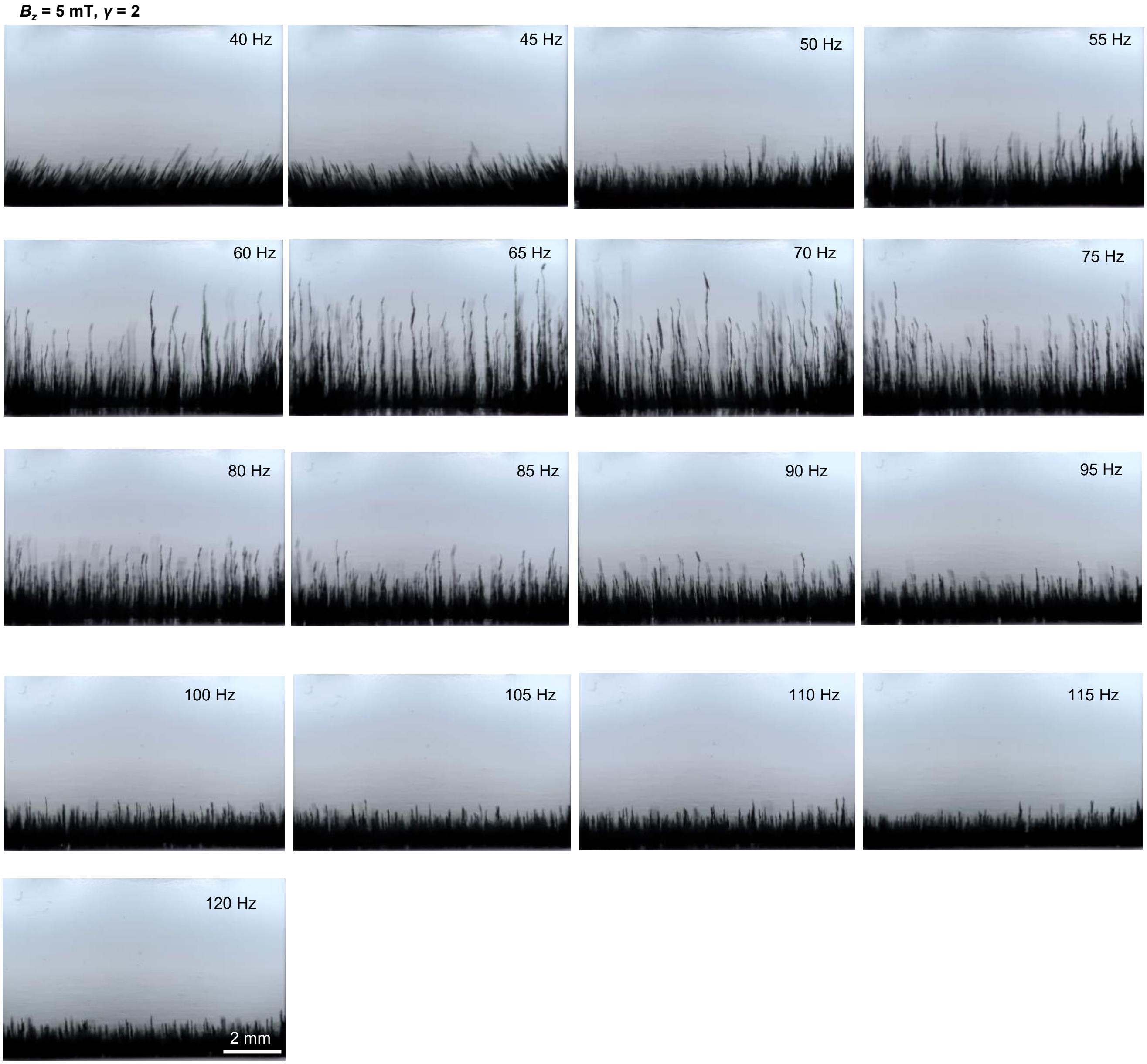

Supplementary Fig. 12

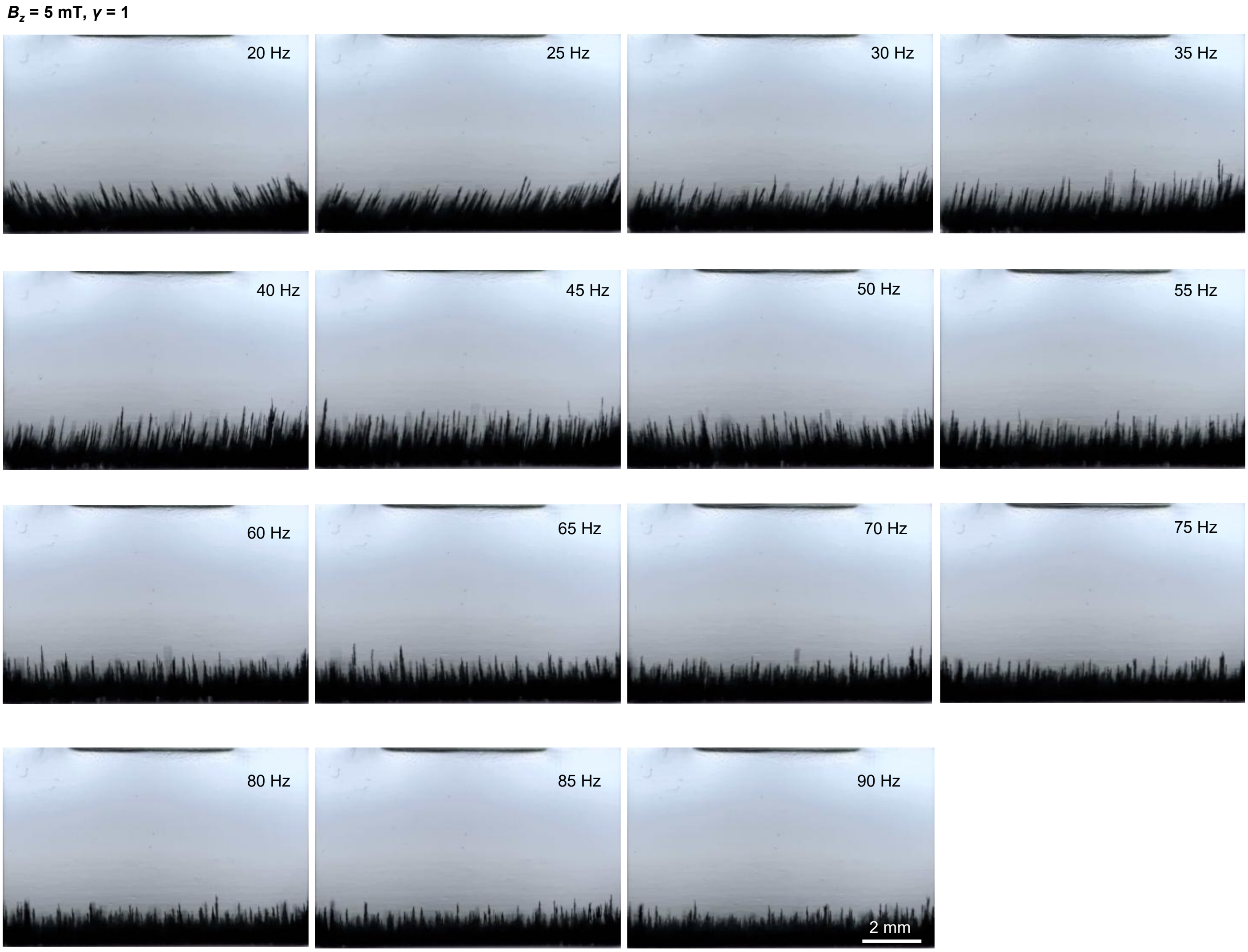

Supplementary Fig. 12

B_z = 4 mT, γ = 4

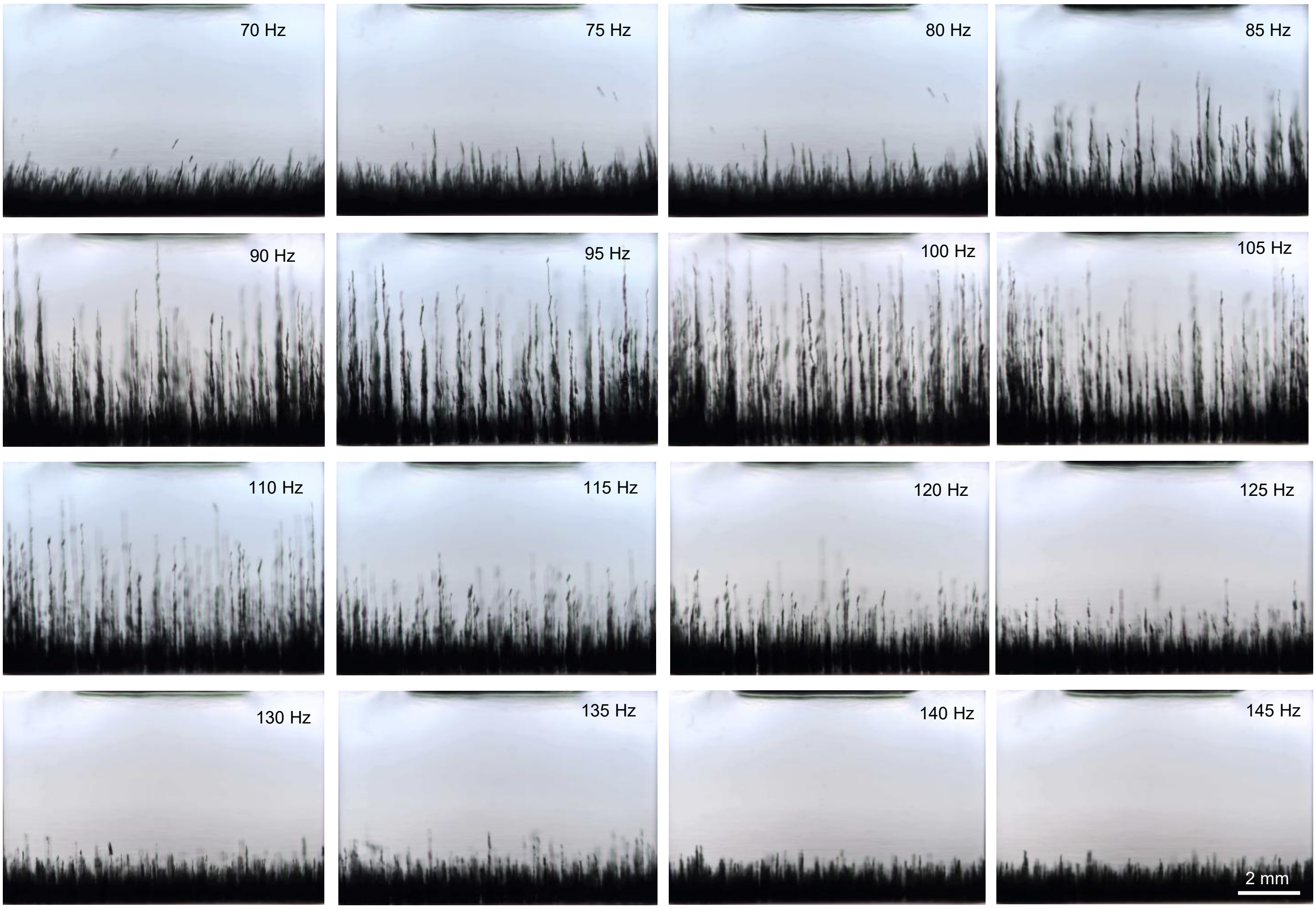

Supplementary Fig. 12

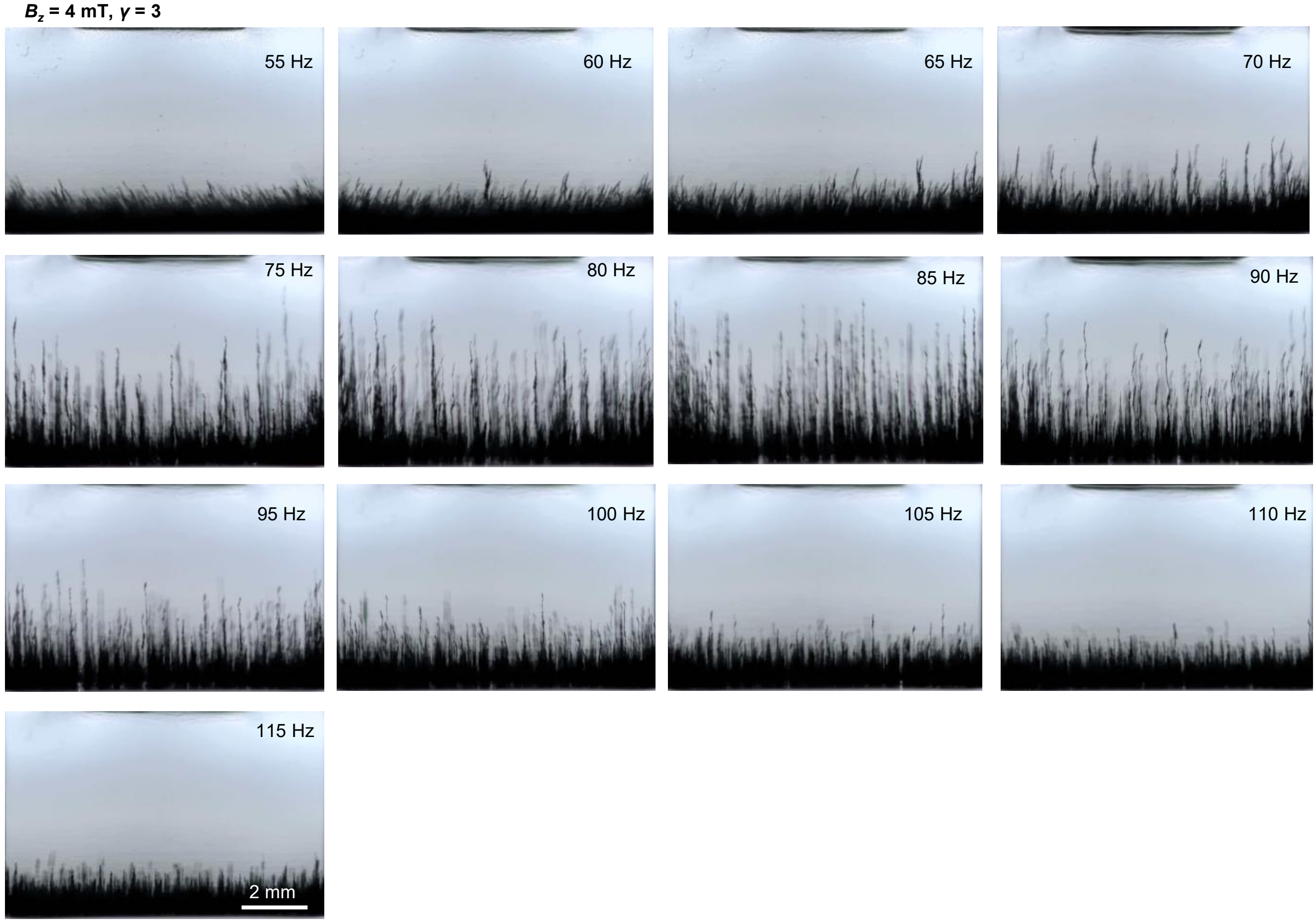

Supplementary Fig. 12

B_z = 4 mT, γ = 2

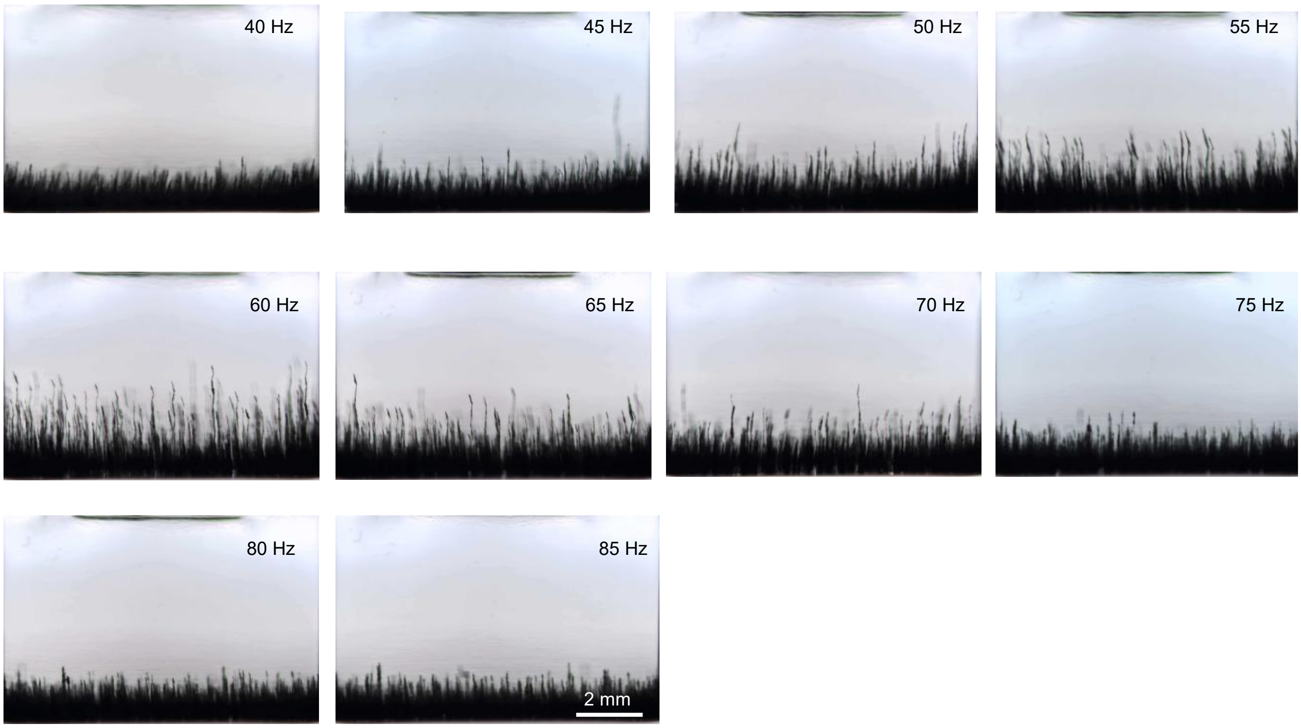

B_z = 4 mT, γ = 1

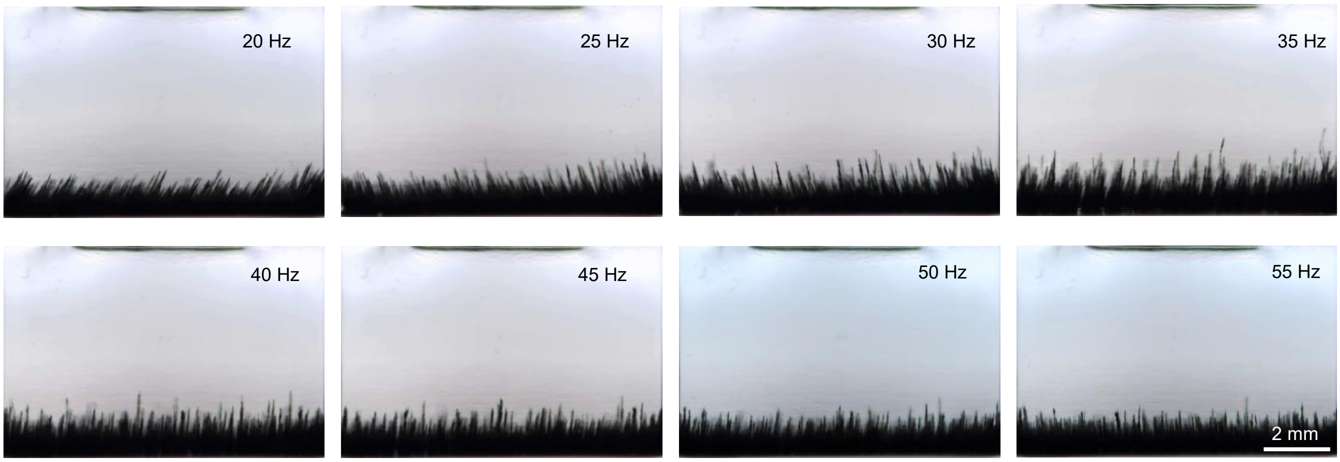

Supplementary Fig. 12

B_z =3 mT, γ = 5

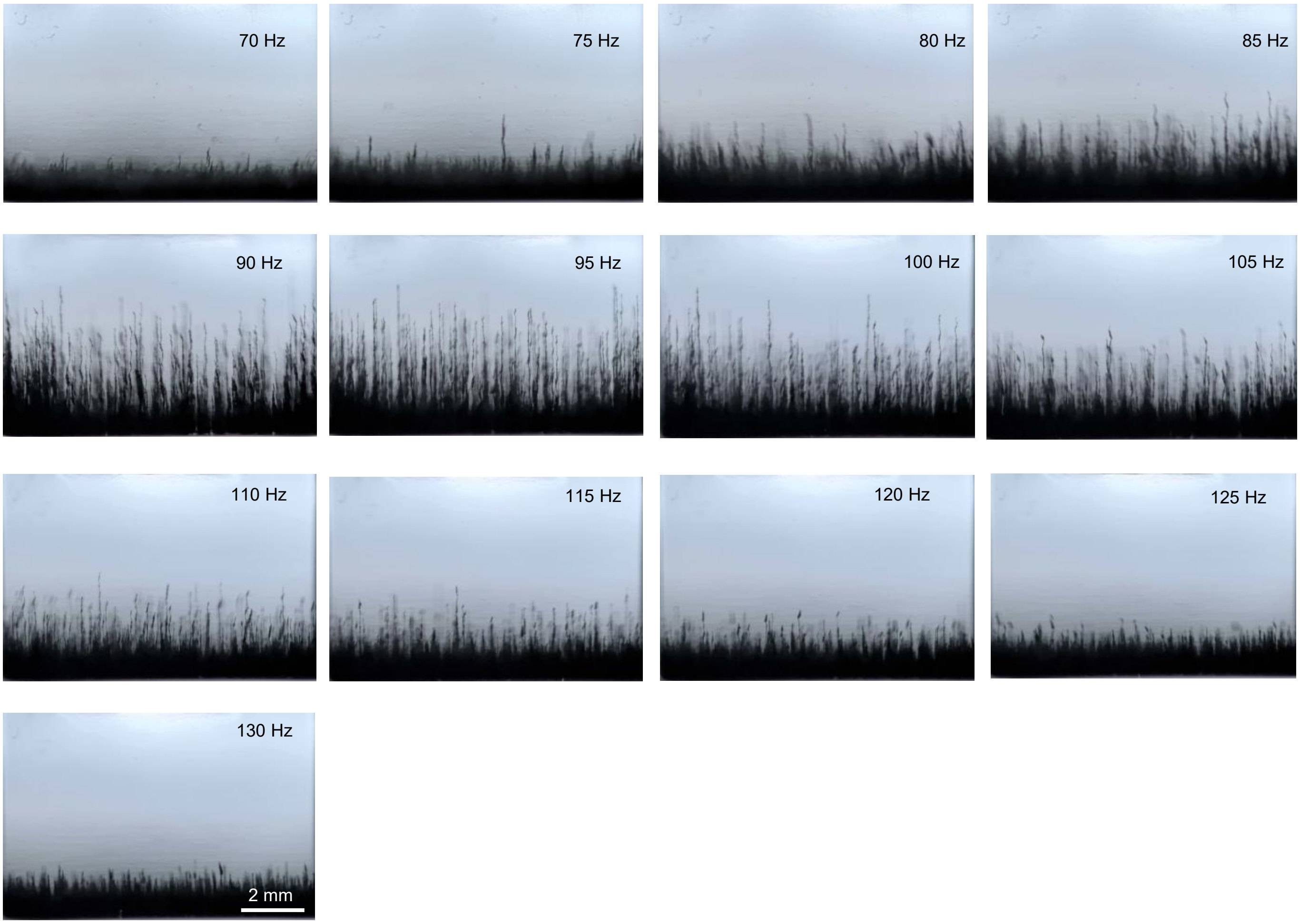

B_z =3 mT, γ = 4

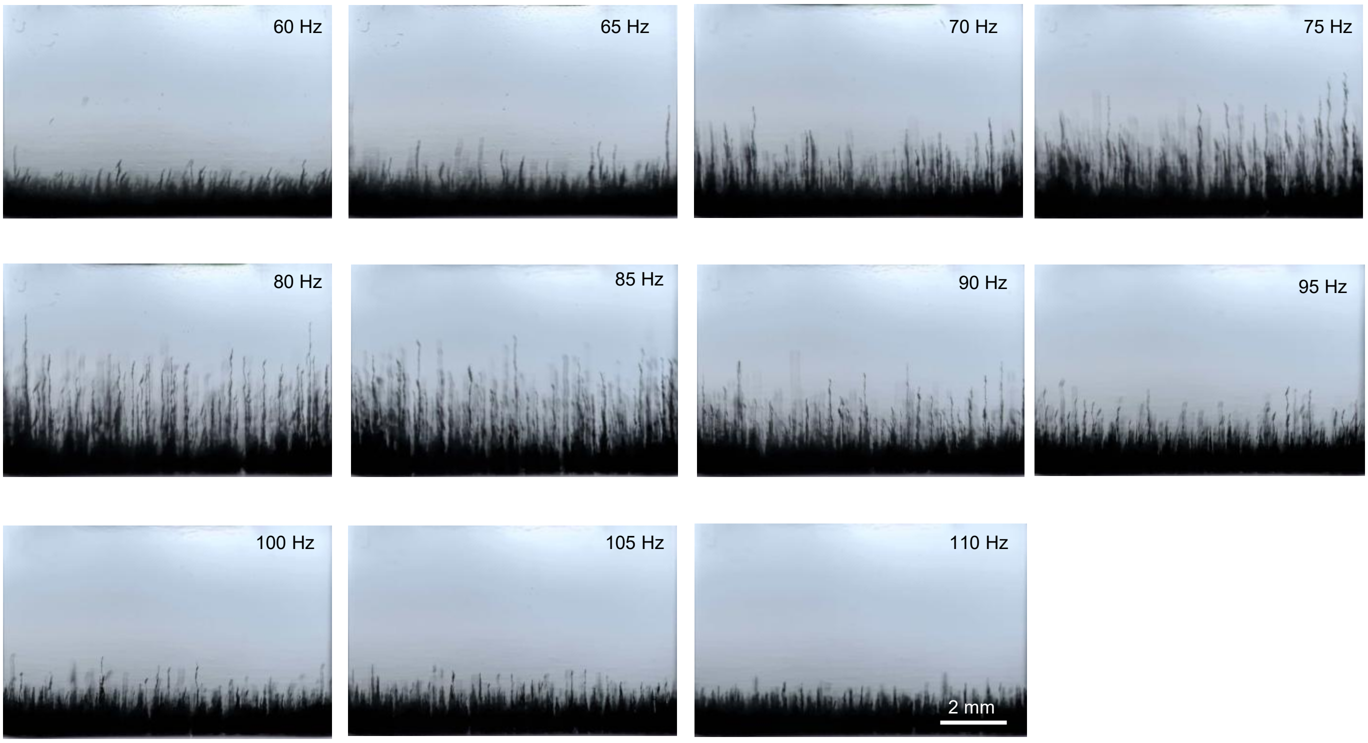

Supplementary Fig. 12

B_z =3 mT, γ = 3

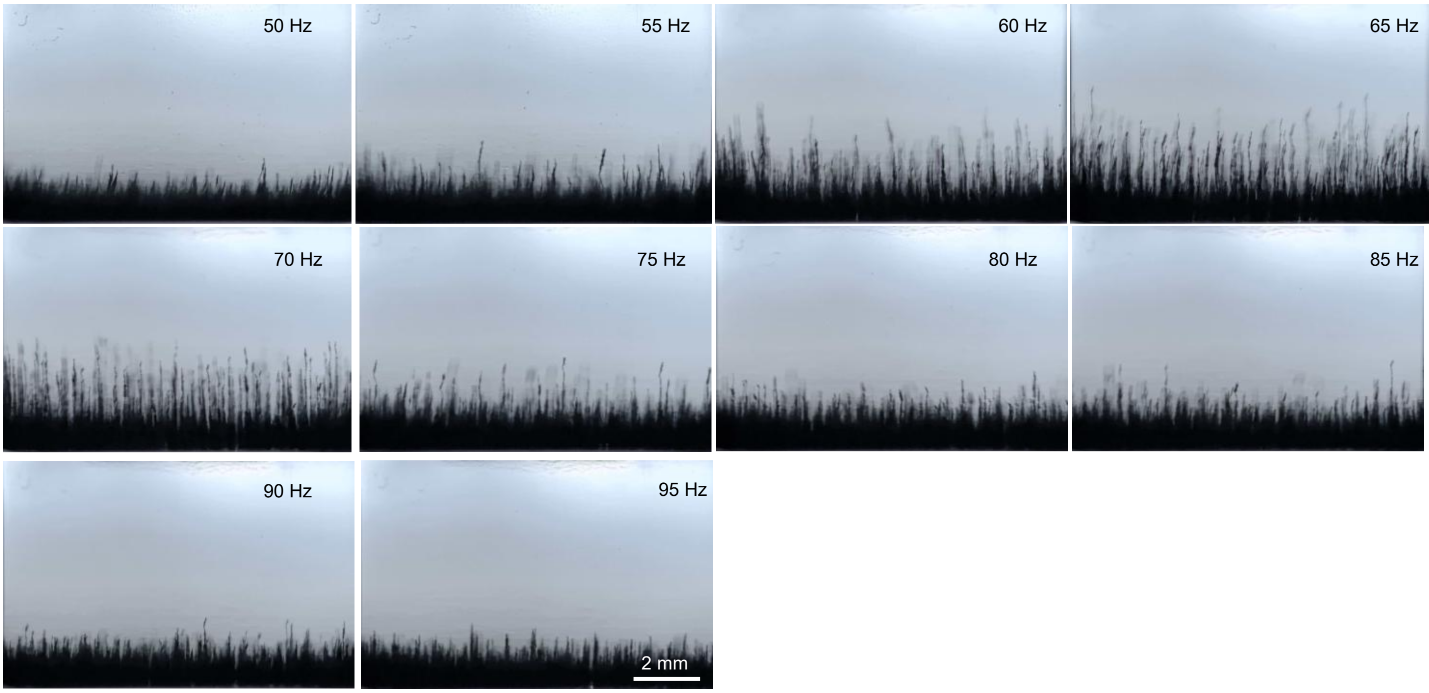

B_z =3 mT, γ = 2

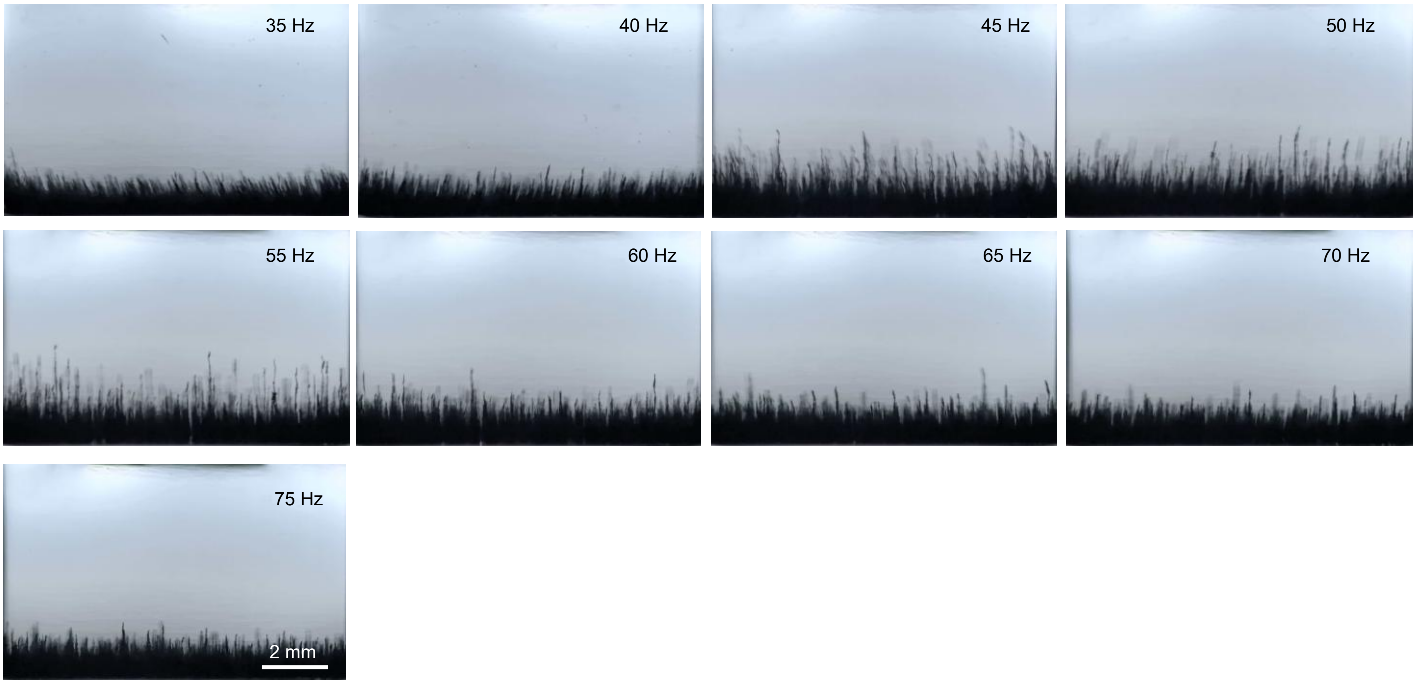

B_z =3 mT, γ = 1

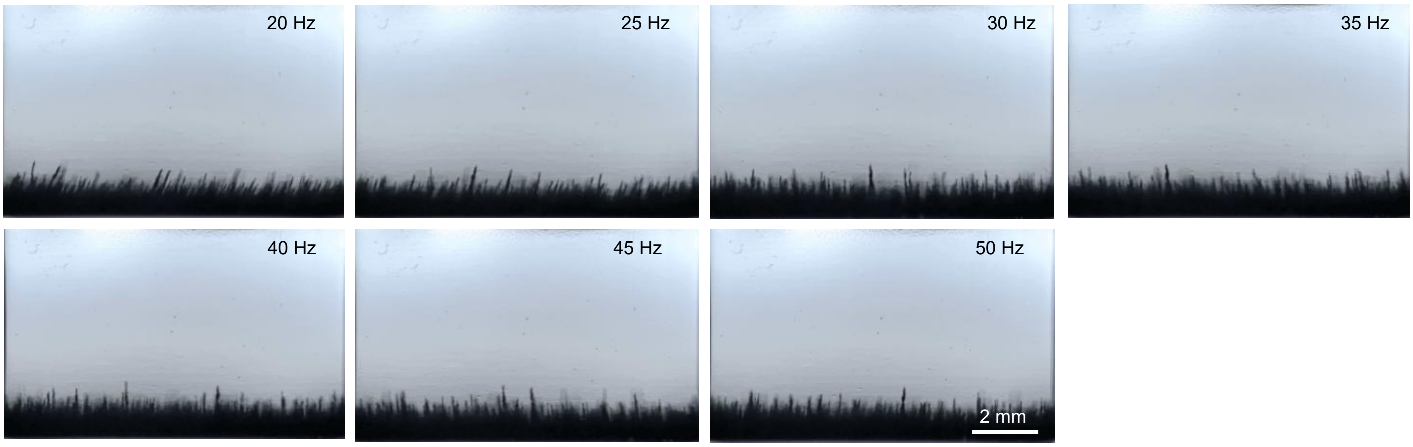

Supplement: Supplementary file 4 — Supplementary Data 1 (Raw data) [file 41467_2026_73696_MOESM4_ESM.zip › Supplementary Data 1/Unprocessed raw data.pdf]
