# Supplementary material for: Transport of enzymatic activity across liquid-liquid interfaces using dynamic assemblies of magnetic particles via field-modulated interactions
Source: Nat Commun. 2026 May 26;17:6872. doi: 10.1038/s41467-026-73696-8 (PMC13388707; doi:10.1038/s41467-026-73696-8)
Supplement: Supplementary file 7 — Transparent Peer Review file [file 41467_2026_73696_MOESM7_ESM.pdf]

nature portfolio

Peer Review File

Transport of enzymatic activity across liquid-liquid interfaces using dynamic assemblies of magnetic particles via field-modulated interactions

Corresponding Author: Professor Ronald Xu

This file contains all reviewer reports in order by version, followed by all author rebuttals in order by version.

Version 0:

Reviewer comments:

Reviewer #1

(Remarks to the Author)
In their manuscript "Gravity-resisting morphogenesis of magnetic seaweed-like structures," the authors report field-driven assembly of magnetic microstructures, which they term M-seaweed. Employing two-axial magnetic field geometry (with one of the axes being a static field and another oscillating) the authors observe and characterize the assembly of the magnetic pillar-like structures assembling along the static field. Since the static field is aligned with gravity, the structures extend vertically. The author refers to this as 'gravity-defying' growth, though that description is somewhat exaggerated. Dual-axis field modulation, spatiotemporal control and structural changes are demonstrated.
Overall, the paper is written in a very descriptive manner, with the focus placed on the absolute values of the parameters used rather than on providing the reader with intuitive or physical insights.
The authors do some analysis/simulations that they show in SI, but do not discuss the implications of those or provide general insights in the main text.
More detailed comments to the point are given below.

1. The field of field-driven dynamic assembly has been in active development for many years, and there is a great number of works in the field where researchers dynamically assembled (using magnetic or electric fields) and investigated structures not bound to only planar geometries. Complex and non-trivial 3d dynamic patterns dynamically assembling in the field-driven systems have been reported. The introduction would greatly benefit from providing a better overview of the current state of the field, in contrast to just making a statement that "the third dimension introduces new opportunities for structural complexity," which leaves the reader with an impression that it has not been previously investigated/explored. Here are just a few examples:
- JE Martin, et al, Physical Review E 61 (3), 2818 (2020)
- ME Leunissen, et al , Advanced Materials, 21, 3116–3120 (2009)
- T. Mohoric et al, Langmuir 32, 20, 5094–5101 (2016)
- JE Martin, Soft Matter 12 (25), 5636-5644 (2016)
- A. Demortière, et al., Nat Commun 5, 3117 (2014)

Also, two-axial and tri-axial time-dependent fields and resulting time-averaged interactions (used in the current manuscript) have been in use by a few groups before to promote the complexity of structures and out-of-equilibrium dynamics. Here is the review paper on the subject: J. Martin et al, Reports on Progress in Physics 76 (12), 126601 (2013).

2. The growth of vertical structures, spikes, made of particles suspended in a fluid in a static magnetic field is similar in nature to the Rosensweig instability observed in ferrofluids (also comprised of magnetic particles, but much smaller, dispersed in a fluid). The vertical spikes in ferrofluids are also stable while a static field is applied.

3. The phase boundaries in the diagram in Fig. 2f show a "deep" around 25Hz. What is special about this frequency. Could it be related to the critical frequency when particles develop lag with respect to the external field? It seems to be the case. It would be instructive to normalize these frequencies by that critical frequency to bring some sort of generalization of the reported results. The same comment is valid for the rest of the figures.

4. The observed patterns (similarly to previously reported systems using a similar approach) are results of the interplay between hydrodynamic and time-averaged magnetic infractions, rather than just “the interplay between a high-frequency horizontal oscillating field and a static vertical field… ”. Some intuitive insights into how frequency changes that interplay (and as a result the whole dynamics of the system) would be valuable for the reader. For instance, rapid oscillations (above critical frequency where particles develop a phase lag with the field) probably lead to the suppression of the hydrodynamic interactions and time-averaged magnetic interactions become dominant in the structure formation, etc.

5. What is f_s in the experiment and how does it relate to the cutoff frequency (critical frequency),f_c ?

6. What are “supercritical frequencies” introduced on page 15?

7. On page 22 authors claim that the system “… sustained high-turbulence flows below 3Hz”. I suspect the authors mean “chaotic flows” instead of “turbulence”. To support the claim of the turbulence, authors should provide the energy spectra for the flows with characteristic exponent analysis.

In summary, the authors performed interesting experiments and characterization of the out-of-equilibrium magnetic colloidal system driven by a two-axial field. The results contribute to the field. The paper lacks novelty and is not suitable for Nature Communications in my opinion. It is better suited for Communications Physics or specialized journals once authors address the deficiencies of their presentation.

Reviewer #2

(Remarks to the Author)
Review for:
Gravity-resisting morphogenesis of magnetic seaweed-like structures
In this paper, the authors propose an innovation in the common approach for magnetic field assisted self-assembly of colloidal particles. The authors expose the particles to a complex field that features a permanent component in the Z direction and a fluctuating component on the XY plane.
The resultant structure is a linear array of particles with periodic kinks they dub an “M-Seaweed”. The addition of the fluctuating component enables the structure to reach far greater heights than one that forms under a permanent uniaxial field. An additional advantage of modulation in the permanent and fluctuating components is for modification of the M-Seaweeds properties. Several examples include an overall structure transition between three distinct morphologies, height and density modifications and the creation of a controlled slanted angle between the structures and the substrate.
The manuscript is rich with information, giving a full description of the “M-Seaweed” structures and their potential.
The following notes arose while reading the manuscript and require improvements from the authors:
MAJOR:
At its current state, the paper reads as a part of an internal conversation inside the self-assembly field. The broader implications of this work beyond its immediate community do not arise from the experiments themselves, and receive very little mention during the discussion. While there are some technical advances, It is unclear what the major impact of the work is. This has not been demonstrated. The authors are required to meaningfully link their work to the broader scientific world, where one would expect a convincing demonstration of the work.
MINOR:
There is significant imbalance between the “results” and “discussion” segments. While the results are comprehensive, the discussion is relatively information poor and serves mostly to present the study’s conclusions.
Furthermore, there are multiple claims presented in the results that are made without experimental or literary substantiation. To remedy both issues in the same time, the authors should move the following claims to the discussion segment and include supporting citations (and/or present theoretical frameworks).
The claims are as follows:
• The various sentences scattered throughout the paper describing the advantages of the M-Seaweed should be combined in the discussion and presented in relation to existing structures.
• “Growth mechanism and segmented morphology” subsection: Most of this subsection constitutes discussion and would benefit greatly from additional citations.
• Line 445 “As demonstrated in previous studies” – cite the studies
• Line 509, sentence that starts with “This transition…” – back this claim with a citation or a relevant calculation.
• From line 522 until the end of the results – this entire section needs to be backed by relevant citations.

Following the improvements outlined above this paper qualifies for publication in Nature Communications.

Version 1:

Reviewer comments:

Reviewer #1

(Remarks to the Author)

The revised manuscript is improved. In response to the reviewers' recommendations, the authors have included an overview of the relevant field, further elaborated on their results in the revised manuscript, and offered at least some insight into the underlying observations. As pointed out in my previous report (with corresponding references), similar in nature structures were previously observed in 2 and 3-axial fields, albeit without involving a penetration through an interface. The results, nevertheless, are interesting and contribute to the field of out-of-equilibrium magnetic colloidal assembly. In my opinion, the level of novelty does not meet the standard expected for Nature Communications. The manuscript is better suited for Comm. Materials or Comm. Physics.

Reviewer #2

(Remarks to the Author)
I appreciate that the authors have made revisions and substantially improved the manuscript. In the original version, it was not clearly explained what specific problem the work addresses. The authors have now expanded on their initial study, and several sections have been rewritten to respond to the comments from the first round of review.
My main concern is that the revisions have introduced a number of overstated claims, such as “Our work thus establishes a new paradigm for field-driven magnetic materials” or “a field-driven morphogenesis-to-function paradigm.” Phrases like “on-demand functional execution” read more like marketing language than scientific description.
Along similar lines, while the method used to form the structures and their potential applications are interesting, it seems overstated to claim that these structures are promising for targeted delivery. In practice, one would first need to deliver the magnetic particles and any associated cargo, and then implement a magnetic field system to achieve interfacial penetration and delivery.
I also find the term “gravity-resisting morphogenesis” misleading. “Morphogenesis” is a well-established term primarily used in developmental biology, and the present work is not related to biological morphogenesis.
I would recommend that the authors:
• Avoid using the term “morphogenesis.”
• Tone down the claims about establishing a new paradigm and broad utility.
• Focus instead on what is unique about the growth and assembly process, how it differs from previous assembly studies, and then present the interesting example without exaggeration.
Finally, since the revised manuscript emphasizes assembly across liquid–liquid interfaces and presents a compelling potential application in the conclusions—specifically, a “platform that could be extended to … the tailored delivery of biomolecular cues (e.g., growth factors) across biological membranes for targeted biological modulation”—I recommend that the authors demonstrate such an application, perhaps in a model lab-on-a-chip system. Including such a proof-of-concept experiment would significantly strengthen the paper. As it currently stands, the magnetic assembly is interesting, but the reader is left uncertain about its practical utility.

Version 2:

Reviewer comments:

Reviewer #1

(Remarks to the Author)
The authors have further improved the manuscript following the latest revision. With the added experiments and data, the paper now meets the bar for publication in Nature Communications.

Reviewer #2

(Remarks to the Author)
The authors have revised their manuscript. I am grateful that the authors have toned down the hyperbole that accompanied several overstated claims in the previous version of their manuscript. The title has also been adapted and the confusing terminology concerning morphogenesis has been removed. This is a major improvement. The authors have also taken my suggestion to heart to demonstrate that the magnetic structures can indeed deliver something 'useful' across an interface. Originally, the paper contained claims of growth factors in biological systems and/or delivery in the context of therapeutics or "targeted biological modulation". The demonstrated example falls short of the originally claimed ability of M-seaweed to modulate biological function. I still do not really see that the method will be useful for biology, given its complexity and the magnetic colloidal structures that are most likely not desirable in most biological experiments. Nevertheless, I commend the authors for including the demonstration and for undertaking a string of experiments that are now presented in a new section of the paper. The authors have in their latest revision shown that their method permits the delivery of an enzyme across an interface. This demonstration is a valuable addition to the paper. I have no further comments and I think it is now time for a wider audience to judge the merits of the presented method.

Open Access This Peer Review File is licensed under a Creative Commons Attribution 4.0 International License, which permits use, sharing, adaptation, distribution and reproduction in any medium or format, as long as you give appropriate credit to the original author(s) and the source, provide a link to the Creative Commons license, and indicate if changes were made.
In cases where reviewers are anonymous, credit should be given to 'Anonymous Referee' and the source.
The images or other third party material in this Peer Review File are included in the article's Creative Commons license, unless indicated otherwise in a credit line to the material. If material is not included in the article's Creative Commons license and your intended use is not permitted by statutory regulation or exceeds the permitted use, you will need to obtain permission directly from the copyright holder.
To view a copy of this license, visit https://creativecommons.org/licenses/by/4.0/

Response Letter

Reviewer #1

In their manuscript "Gravity-resisting morphogenesis of magnetic seaweed-like structures," the authors report field-driven assembly of magnetic microstructures, which they term M-seaweed. Employing two-axial magnetic field geometry (with one of the axes being a static field and another oscillating) the authors observe and characterize the assembly of the magnetic pillar-like structures assembling along the static field. Since the static field is aligned with gravity, the structures extend vertically. The author refers to this as 'gravity-defying' growth, though that description is somewhat exaggerated. Dual-axis field modulation, spatiotemporal control and structural changes are demonstrated.

Response: We thank the reviewer for the insightful comment regarding the use of the term "gravity-defying" in our original manuscript. The reviewer is correct to note that a more precise description is warranted. Our intent was to emphasize that the vertical growth and subsequent interfacial crossing of the structures occur against gravity, requiring the system to overcome gravitational settling. We agree that "gravity-defying" could be perceived as overstating the case.

In direct response to this comment, we have thoroughly revised the manuscript. Specifically:

1. The Abstract and Introduction have been entirely rewritten to provide a more precise and balanced framework, eliminating any potentially exaggerated phrasing and strengthening the logical narrative leading to our work.

2. Importantly, this revision process provided an opportunity to integrate new experimental data that significantly strengthens our central claim. We have now included a compelling functional demonstration where the M-Seaweed, upon crossing the PFH-(H_2O_2/ABTS) interface, delivers an enzyme horseradish peroxidase (HRP) to trigger a localized chromogenic reaction (**Revised Fig. 1 and Revised Fig. 7**). This experiment concretely shows the system's capability to perform a defined "task" upon phase-boundary penetration, moving beyond structural formation to functional execution.

We believe these revisions have not only addressed the terminology concern but have also substantially enhanced the manuscript's clarity, rigor, and impact. We thank the reviewer for prompting these improvements.

Revised Abstract:

~~Field-driven dynamic self-assembly has revolutionized the development of responsive and reconfigurable materials, yet bottom-up gravity-resisting growth, especially in the case of dynamic high-aspect-ratio structures, remains a challenge due to the competing requirements of~~

gravitational resistance and structural metastability. Here, we present Magnetic Seaweed (M-Seaweed), a reconfigurable microparticle system that achieves gravity-resistant morphogenesis of segmented seaweed-like structures through frequency-modulated magnetic moment relaxation bottom-up assembly. By leveraging high-frequency oscillations to activate time-varying magnetic interactions while preserving directional guidance via static fields, the system drives spontaneous sprouting-like growth, ultimately forming high-aspect-ratio architectures in dynamic equilibrium. Through precise dual-axis field modulation, we demonstrate spatiotemporal control over structural dynamics, including vertical growth, inclined extension, reversible morphological transitions, and dynamic-to-static switching for post-assembly actuation. Furthermore, M-Seaweed achieves gravity-defying bottom-up transfer across immiscible interfaces through continuous dynamic vertical growth. This work overcomes the trade-off between gravitational resistance and structural stability in dynamic self-assembly, establishing a new platform for field-directed reconfigurable materials and active interfacial systems.

The ability to actively traverse phase boundaries to perform functions, a hallmark of many biological systems, inspires the design of synthetic counterparts, which hold promise for applications in targeted delivery and adaptive sensing. However, achieving such systems, particularly within stratified liquids, presents a significant challenge, demanding dynamic architectures that combine continuous gravity-resisting growth, interfacial penetration, and post-crossing action. Here, we report "Magnetic Seaweed" (M-Seaweed), a field-driven microparticle system that integrates this entire functional cascade. Using a synergistic combination of static and oscillating magnetic fields, we guide magnetic microparticles to assemble into dynamic, high-aspect-ratio, seaweed-like structures that exhibit gravity-resisting growth upward from a substrate. These dynamic architectures are reconfigurable, stabilizable, and programmably actuatable, and can be steered to penetrate a perfluorohexane-water interface. By functionalizing particles with an enzyme, the growing M-Seaweed structures act as micro-transporters, delivering catalytic activity across the interface to trigger a localized, macroscopic chromogenic reaction upon penetration. Our work establishes a field-driven morphogenesis-to-function paradigm, bridging structural growth, phase-boundary penetration, and triggered function toward intelligent microsystems for targeted delivery and adaptive sensing.

The last paragraph of the revised Introduction:

To address these challenges, we developed Magnetic Seaweed (M-Seaweed), a microparticle system that achieves gravity-resisting morphogenesis via coordinated magnetic field modulation (Fig. 1a). By combining high-frequency oscillating fields (inducing time-varying magnetic interactions) with static field-guided directional control, this system generates high-aspect-ratio (>16), segmented seaweed-like architectures, with dynamic visualization clearly capturing X-shaped vertical stacking patterns (Fig. 1b). Mechanism analysis and computational simulation reveal that frequency-dependent magnetic moment relaxation governs the assembly process by regulating the inter-unit interactions, directly influence the growth dynamics. Precise field

parameter modulation enables programmable control over vertical growth, angular extension, and reversible structural transitions, establishing a spatiotemporally tunable platform for gravity-resistant morphogenesis (Fig. 1c). Post assembly stabilization is achieved by maintaining the vertical field component after cessation of oscillating excitation, locking dynamic assemblies into static, stable configurations at sub-centimeter scales. Further field modulation allows post-assembly actuation (controlled swaying motions), suggesting potential applications in fluid manipulation (e.g., pumping or mixing). Moreover, M-Seaweed accomplishes **gravity-defying** bottom-up transport across perfluorohexane (PFH)-water immiscible interfaces through the synergistic interplay of continuous dynamic growth, geometry-enabled interfacial penetration, and self-amplifying interfacial recruitment (Fig. 1d). This work provides a strategy for designing reconfigurable functional materials and active interfacial transport systems.

Here, we report "Magnetic Seaweed" (M-Seaweed): a field-driven microparticle system that achieves gravity-resisting morphogenesis of segmented seaweed-like structures, active interfacial penetration, and post-penetration functional triggering (Fig. 1). By synergistically combining static and oscillating magnetic fields, the system drives spontaneous sprouting-like growth, ultimately forming high-aspect-ratio architectures in dynamic equilibrium (Fig. 1a). These dynamic architectures are reconfigurable, stabilizable (i.e., lockable into static configurations), and programmably actuatable through field modulation (Fig. 1b). They can also be steered to penetrate a perfluorohexane (PFH)-water interface, thereby transferring the majority of the constituent microparticles from the substrate into the upper phase (Fig. 1c). Crucially, we demonstrate that this interfacial crossing can be functionally harnessed by decorating particles with the enzyme horseradish peroxidase (HRP): the growing M-Seaweed structures act as micro-transporters, delivering catalytic activity into a target phase containing H_2O_2 and ABTS (a chromogenic substrate) to trigger a localized, macroscopic color change upon penetration. Our work thus establishes a new paradigm for field-driven dynamic materials, bridging structural growth, phase boundary penetration, and on-demand functional execution, and opening pathways toward intelligent microsystems capable of operating across stratified environments

Revised Fig. 1

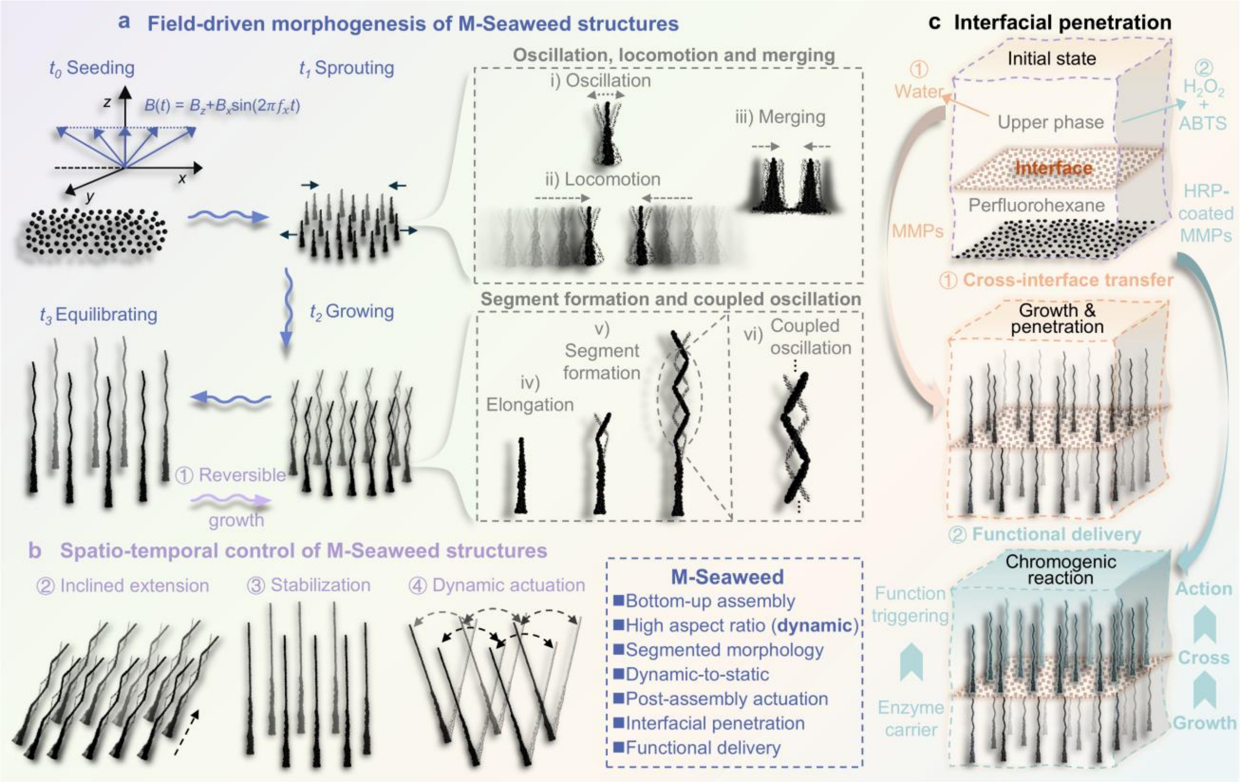

Fig. 1 | Magnetic field-modulated gravity-resisting morphogenesis, dynamic control, and functional delivery of M-Seaweed. a Dynamic self-assembly and vertical growth of M-Seaweed structures under the oscillating magnetic field, with key morphological transitions including locomotion, merging, elongation, and segment formation. **b** Programmable structural control via magnetic field modulation, demonstrating four operational modes: reversible growth, inclined extension, post-assembly stabilization, and dynamic actuation. **c** Interfacial penetration across a water/PFH interface (orange arrow), and the triggered chromogenic reaction using HRP-modified particles with an H_2O_2/ABTS upper phase (green arrow).

Revised Fig. 7

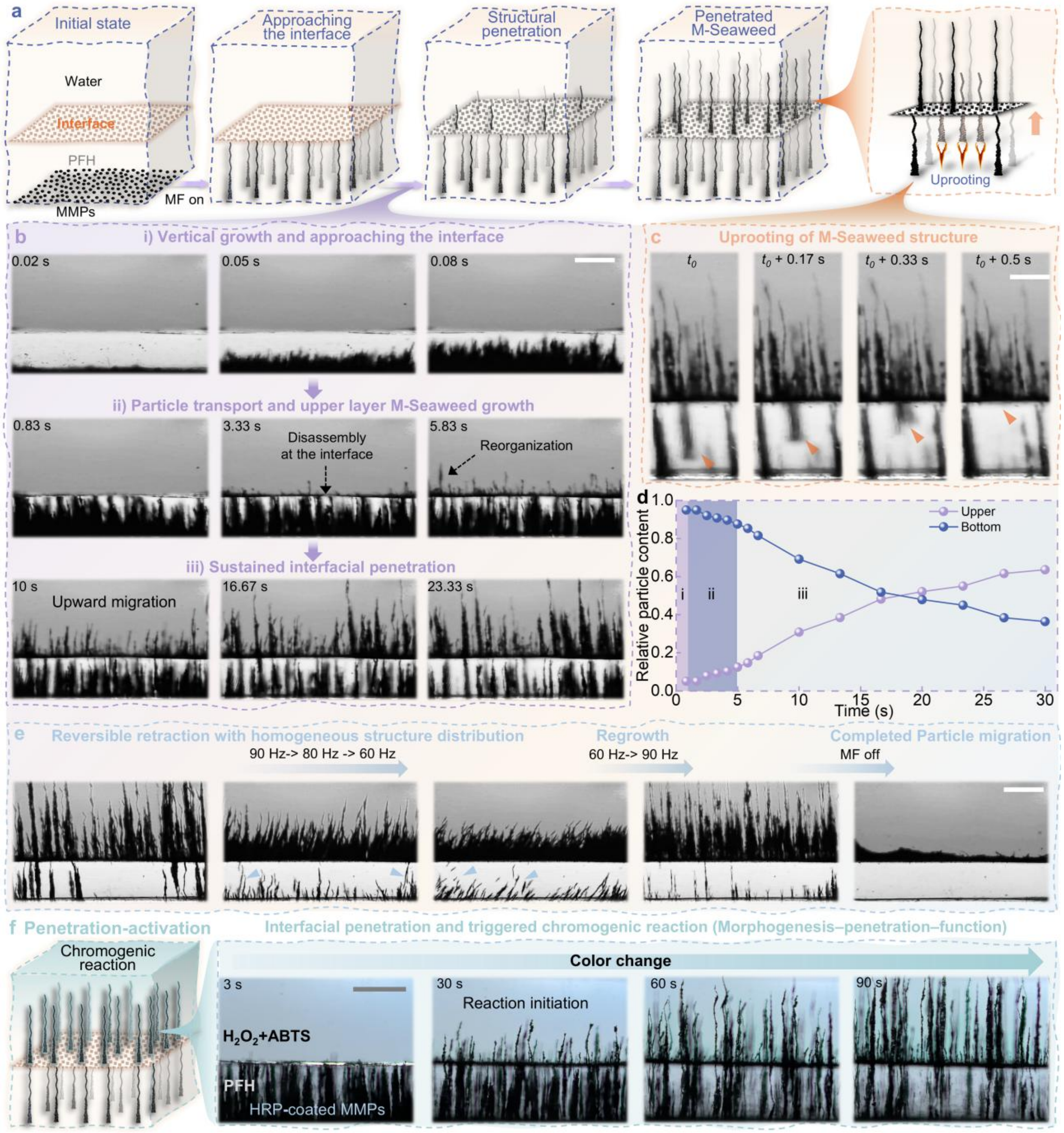

Fig.7 | Interfacial penetration and functional delivery of M-Seaweed. a Schematic of the M-Seaweed…, **b**… **e**…**f** Localized chromogenic reaction triggered by the interfacial penetration of M-Seaweed structures, using HRP-modified particles and an H_2O_2/ABTS upper phase. Scale bar, 2 mm.

Overall, the paper is written in a very descriptive manner, with the focus placed on the absolute values of the parameters used rather than on providing the reader with intuitive or physical insights.

Response: We sincerely thank the reviewer for this overall perspective on the manuscript's style. We agree that achieving a balance between descriptive reporting and providing deep physical insight is crucial. Our intent in presenting absolute parameter values in the main text was to ensure precise experimental reproducibility and to offer direct, actionable guidance for implementing the spatiotemporal control and functional demonstrations (e.g., interfacial crossing and enzymatic delivery).

More importantly, we fully acknowledge the reviewer's emphasis on extracting generalized physical insight, and we are grateful for the specific suggestion in **comment 3**, which directly addressed this point. As detailed in our response below (see **comment 3**), we have conducted an extensive additional parametric study and performed the suggested normalization analysis. The additional data presented in Supplementary Fig. S12 reveals a universal scaling law that collapses our data across parameters. This analysis moves beyond description to identify the underlying dimensionless criterion governing the growth window, substantially strengthening the physical insight of the study.

In the revised manuscript, we have integrated this normalized, generalized scaling analysis into the Supplementary Information as the foundational physical framework. The main text retains the dimensioned parameter descriptions to ensure clarity and reproducibility of the core experimental findings. We believe this revised structure successfully balances the need for precise experimental description with the provision of deeper physical understanding, thanks to the reviewer's valuable feedback.

The authors do some analysis/simulations that they show in SI, but do not discuss the implications of those or provide general insights in the main text.

Response: We thank the reviewer for highlighting the need to better integrate the insights from our supplementary analysis and simulations into the main text. We agree that drawing out the general physical principles from this supporting work is crucial. In response, we have substantially revised the “Growth mechanisms and segmented morphology” section to explicitly state and discuss the key implications derived from our analysis/simulations.

The revised text now frames the entire growth process around a fundamental frequency-dependent competition between hydrodynamic and time-averaged magnetic interactions. This overarching insight directly originates from the analysis in Supplementary Note S1. The phase lag defined there is not merely a detail but the physical origin of the time-averaged magnetic interaction-dominated state: it explains the condition under which the rapid field oscillation “averages out,” allowing the static-field-aligned dipole interactions to dominate vertical growth while suppressing disruptive hydrodynamic interaction.

Furthermore, the main text now draws upon these supplementary information to explain key aspects of M-Seaweed morphogenesis:

Based on the magnetic moment response model defined in **Supplementary Note S1**, our particle dynamics **simulations** incorporate the phase lag and amplitude attenuation of the magnetic moment. These **simulations** capture the key processes of M-Seaweed morphogenesis and reveal the frequency-dependent characteristics, which align closely with experimental observations (Fig. 4a, b). The simulations show that at lower frequencies, where the magnetic moment closely follows the field, large-amplitude motion enhances hydrodynamic interactions, leading to tilted, fragmented structures. Conversely, at higher frequencies, the pronounced phase lag suppresses hydrodynamic effects and promotes time-averaged magnetic interactions, resulting in stable vertical growth. This agreement between simulation and experiment confirms that the frequency-dependent shift in interaction balance governs the transition from lateral instability to directional elongation.

The frequency-modulated growth model (**Supplementary Note S2**) and the accompanying experimental analysis provide a generalized, quantitative framework that moves beyond observation to explain the underlying growth kinetics. This model explicitly links frequency-dependent collision efficiency to the macroscopic growth outcomes. In direct response to the reviewer's suggestion for deeper insight, **we performed new experiments to validate this link**. These include time-lapse visualizations of the distinct microscopic collision-fusion activity patterns across frequencies (Supplementary Fig. S19) and a quantification of structural consolidation via the net decrease in areal density, which peaks at the critical frequency (Supplementary Fig. S20). By integrating this model with the experimental growth characterization (Supplementary Fig. S21a-e), we establish frequency-dependent exponential scaling laws (Supplementary Fig. S22f) that universally describe how collision kinetics govern both the growth velocity and the final attainable height of M-Seaweed structures.

The mechanism for the segmented morphology is explained by referencing the resonance and shear banding analysis (**Supplementary Note S3**), transforming a descriptive observation into a consequence of dynamic mechanical instability. The elastic beam model is not merely a fit to the observed segmentation. It reveals a general self-organization mechanism based on dynamic resonance avoidance. As the structure grows, its natural frequency decreases. When it matches the driving frequency, resonance excites higher-order bending modes that induce shear banding at nodal points, leading to segmentation. The key insight is that this segmentation is the system's adaptive response to prevent destructive resonance; by breaking into shorter units, each segment raises its natural frequency above the drive, achieving stable, detuned oscillations. This explains why the segmented morphology emerges as a stable, non-equilibrium configuration.

Supplementary Note S4 (Coupled oscillation of segmented M-Seaweed architectures): The coupled oscillator model provides a general framework for understanding the dynamic coordination within a segmented active structure. It explains how individual segments can

oscillate with independent phases (due to mechanical decoupling and fluid drag) while remaining globally coordinated through long-range magnetic dipole-dipole interactions. The resulting insight is a design principle of decoupled mechanics but coupled magnetism, which allows the structure to be locally flexible and responsive to fluid forces without losing its global architectural coherence and capacity for directed motion. This principle is crucial for the structure's robustness in fluid environments.

In summary, by systematically integrating the insights from our supplementary analysis and simulations—spanning the origins of time-averaged interactions, collision-driven growth kinetics, and the formation of the segmented architecture—we have transitioned from a descriptive account to a mechanistic and predictive understanding of M-Seaweed morphogenesis. These elements collectively establish a coherent physical framework that explains not only how the structures form and grow, but also why they exhibit their unique dynamic stability and functional capabilities. We believe this significantly strengthens the physical insight into our work. The key additions and clarifications discussed above have been incorporated into the revised "Growth mechanisms and segmented morphology" section of the main text, as detailed in our **response to Comment 4**.

More detailed comments to the point are given below.

1. The field of field-driven dynamic assembly has been in active development for many years, and there is a great number of works in the field where researchers dynamically assembled (using magnetic or electric fields) and investigated structures not bound to only planar geometries. Complex and non-trivial 3d dynamic patterns dynamically assembling in the field-driven systems have been reported. The introduction would greatly benefit from providing a better overview of the current state of the field, in contrast to just making a statement that "the third dimension introduces new opportunities for structural complexity," which leaves the reader with an impression that it has not been previously investigated/explored. Here are just a few examples:

- JE Martin, et al, Physical Review E 61 (3), 2818 (2000)

- ME Leunissen, et al , Advanced Materials, 21, 3116–3120 (2009)

- T. Mohoric et al, Langmuir 32, 20, 5094–5101 (2016)

- JE Martin, Soft Matter 12 (25), 5636-5644 (2016)

- A. Demortière, et al., Nat Commun 5, 3117 (2014)

Response: We sincerely thank the reviewer for this important observation and for providing key references. We agree that our original statement did not sufficiently acknowledge the breadth of prior work on 3D field-driven assemblies, which could give a misleading impression.

In direct response, we have substantially revised this section of the Introduction. Rather than making a broad statement about unexplored opportunities, we have reframed the narrative to more accurately define the specific research niche our work addresses. We now explicitly recognize the existence of sophisticated 3D dynamic patterns while articulating a more precise limitation: the lack of structures capable of dynamic, high-aspect-ratio growth and active functional penetration across an interface.

The reviewer's suggested literatures have been instrumental in this refinement. We have integrated these references into the revised text to support a key point (now in the sentence beginning "Even when three-dimensional dynamic assemblies are achieved…"). There, we cite relevant works to substantiate the observation that existing 3D assemblies are often phase-confined or lack the persistent growth required for the interfacial crossing and functional execution that are central to our study.

We believe this revised framework provides a more balanced and accurate overview of the field, sharply defining the unmet challenge that motivates our work. Thank you for this constructive suggestion, which has undoubtedly strengthened the scholarly foundation of our manuscript.

The third paragraph of the revised Introduction:

Although dynamic colloidal systems have achieved sophisticated organization, their behavior remains largely confined to two-dimensional or quasi-two-dimensional landscapes, forming in-plane vortices[22,33-35], chains[11,23,36], or ribbon-like assemblies[24,37-39] on planar substrates or at liquid–air interfaces. These configurations inherently lack the out-of-plane structural integrity and the fundamental assembly pathways needed for sustained vertical extension. Even when three-dimensional dynamic assemblies are achieved, they typically either form within a bulk phase (e.g., from particles suspended in a polymer solution[40-42]) rather than growing upward from a substrate while resisting gravity, or they lack the robustness and control required to penetrate a distinct liquid phase. Therefore, the integrated realization of continuous gravity-resisting growth, controlled interfacial penetration, and subsequent functional triggering within one platform remains a systems-level challenge.

The following references have been added to the main text:

40. Demortière, A., Snezhko A., Sapozhnikov M.V., Becker N., Proslier T., Aranson I.S. Self-assembled tunable networks of sticky colloidal particles. Nat. Commun. 5, 3117 (2014).

41. Leunissen, M.E., Vutukuri H.R., van Blaaderen A. Directing colloidal self-assembly with biaxial electric fields. Adv. Mater. 21, 3116-3120 (2009).

42. Martin, J.E., Venturini E., Odinek J., Anderson R.A. Anisotropic magnetism in field-structured composites. Phys. Rev. E. 61, 2818-2830 (2000).

Also, two-axial and tri-axial time-dependent fields and resulting time-averaged interactions (used in the current manuscript) have been in use by a few groups before to promote the complexity of structures and out-of-equilibrium dynamics. Here is the review paper on the subject: J. Martin et al, Reports on Progress in Physics 76 (12), 126601 (2013).

Response: We sincerely thank the reviewer for highlighting the important review by J. Martin et al., Rep. Prog. Phys. 76, 126601 (2013) and for raising the point regarding the use of time-dependent fields. We have studied this review carefully and agree that it excellently summarizes a foundational principle in the field: the use of multi-axial, time-dependent magnetic fields to create complex time-averaged interactions and drive colloidal systems into non-equilibrium states.

The review, along with the body of work it summarizes, primarily focuses on systems where magnetic particles are suspended within a bulk fluid (often density-matched) or confined at a liquid-air interface. In these configurations, gravitational settling is typically mitigated or inconsequential, allowing the field-generated time-averaged interactions to act on freely diffusing or interfacial-trapped particles. This leads to the formation of diverse patterns (e.g., chains, asters) within a single, continuous phase where gravity is not a dominant factor governing the assembly outcome.

In contrast, our M-Seaweed system introduces a fundamentally different paradigm: bottom-up, persistent morphogenesis from a substrate against gravity. Our particles are not suspended in a stabilizing medium; they begin assembled on a surface and are driven to grow continuously into dynamic, high-aspect-ratio, segmented architectures, a novel "seaweed-like" morphology not reported before. This gravity-resisting, extensional growth itself is a key outcome enabled by our specific field parameters. In addition, this architecture is a quintessential non-equilibrium state: removal of the magnetic field leads to the immediate collapse of the entire structure under the dominant influence of gravity, underscoring that its existence and coherence are wholly sustained by the continuous energy input against gravity.

More importantly, this unique structural capability enables a functional sequence that goes beyond pattern formation within a phase. We demonstrate that the growing structure can actively traverse an immiscible liquid-liquid interface and, crucially, trigger a defined chemical reaction (enzymatic color change) in the target phase upon crossing.

Therefore, the principle of time-averaged interactions is leveraged here within a distinct implementation and objectives: to program sustained bottom-up morphogenesis from a substrate, which yields the dynamic, high-aspect-ratio architecture. This structure is not merely a dynamic pattern but a coherent, growing entity whose form and dynamics inherently enable subsequent phase-boundary penetration and triggered function. Thus, our work establishes a form of field-

driven assembly where programmed structural growth is intrinsically linked to functional capability, demonstrating a complete sequence from morphogenesis to functional triggering.

We thank the reviewer again for prompting us to articulate the unique contributions of our work, which we have now more explicitly framed in the revised Discussion section.

The second paragraph of the revised Discussion:

Our approach enables sustained, directional growth that bridges distinct phases, a capability that has been largely inaccessible to field-driven dynamic assemblies. Whereas prior systems, which often leverage time-averaged interactions from multi-axial[58], time-dependent fields to create complex patterns within a bulk fluid or at an interface, have largely been confined to two-dimensional organization or to three-dimensional aggregates within a single medium under negligible gravitational influence, they have generally lacked the capacity for persistent vertical extension from a substrate across interfacial boundaries. In contrast, the assembly of M-Seaweed applies this principle of field-programmed time-averaged interactions to a distinct paradigm: the bottom-up, persistent morphogenesis of structures directly from a dense particle layer against the dominant influence of gravity. These tuned interactions, mediated hydrodynamically and magnetically via the oscillating field frequency, simultaneously promote magnetic units bonding for elongation and confer sufficient structural stiffness to resist buckling under gravitational and hydrodynamic loads, enabling the growth of dynamic, high-aspect-ratio, segmented architectures not accessible in suspension-based or interface-confined systems.

The following references have been added to the main text:

58. Martin, J.E., Snezhko A. Driving self-assembly and emergent dynamics in colloidal suspensions by time-dependent magnetic fields. Rep. Prog. Phys 76, 126601 (2013).

2. The growth of vertical structures, spikes, made of particles suspended in a fluid in a static magnetic field is similar in nature to the Rosensweig instability observed in ferrofluids (also comprised of magnetic particles, but much smaller, dispersed in a fluid). The vertical spikes in ferrofluids are also stable while a static field is applied.

Response: We sincerely thank the reviewer for raising the insightful comparison to the classical Rosensweig instability in ferrofluids, a foundational phenomenon in the study of field-structured magnetic materials. The analogy is appreciated and provides a valuable opportunity to clarify the specific advances of our M-Seaweed system.

However, while the final static morphology may bear a visual resemblance, the mechanism of formation, the resultant structural properties, and the achievable functionalities in our M-

Seaweed system are fundamentally distinct and represent a significant advance. We respectfully clarify the key differences below:

1. Particle scale and medium: The Rosensweig instability typically occurs in ferrofluids, where nanoscale magnetic particles are stably dispersed in a carrier liquid. In contrast, our M-Seaweed system employs micrometer-scale particles that are not suspended in a density-matched fluid but start from a dense layer on a substrate. This fundamental difference in the starting condition precludes the equilibrium fluid-instability mechanism and instead requires a programmed, energy-dissipative growth process to achieve gravity-resisting vertical extension.

2. Growth mechanism and dynamics: The Rosensweig peaks form via a spontaneous, equilibrium-driven fluid instability upon applying a threshold static field. Our stabilized M-Seaweed structures, however, are not formed by a static field alone. They are the product of a defined two-step field sequence: first, a synergistic oscillating/static field drives a continuous, non-equilibrium, bottom-up growth process; second, the oscillating component is switched off to "lock" the already-formed, high-aspect-ratio structure into a static configuration. This programmable transition from dynamic assembly to static stabilization is a core innovation.

3. Structural properties: The high aspect ratio of our stabilized structures originates from the unique non-equilibrium growth process. During this dynamic phase, the architecture develops as a segmented, seaweed-like structure. Upon stabilization, this pre-formed, high-aspect-ratio morphology is preserved. The achieved aspect ratios are substantially greater than the limited aspect ratios characteristic of Rosensweig peaks or of static-field-assembled pillars formed via bottom-up assembly from a sedimented particle layer. Furthermore, we quantify and control their post-stabilization mechanics, such as the tilt angle arising from the balance of magnetic and gravitational torques, a level of mechanical analysis not typically applied to fluid instabilities.

4. Functional capabilities: The "locked" M-Seaweed structure is not an endpoint. Its stability is reversibly controllable. The structure can be mechanically loaded by reducing the vertical field and then recovered by restoring the field. More importantly, as demonstrated in subsequent sections, the same structure can be reactivated into dynamic actuation. This creates a complete workflow: programmable growth followed by static stabilization, and finally on-demand re-actuation/function.

In summary, while the Rosensweig instability provides a useful reference point for field-induced vertical structuring, our work moves beyond this equilibrium paradigm. We introduce a programmable, non-equilibrium morphogenesis pathway that creates uniquely high-aspect-ratio, mechanically well-defined structures, which can be dynamically assembled, statically locked, and functionally deployed. This capability to seamlessly bridge dynamic and static regimes within a single, reconfigurable micro-architecture opens distinct avenues in adaptive micromachines and functional active matter.

We are grateful for the reviewer's insightful comment, which prompted us to articulate these distinctions more clearly. The key comparative points regarding particle scale, growth mechanism, structural properties, and functional capabilities highlighted above have been incorporated into the revised Discussion section to better contextualize the advance of our work.

The third paragraph of the revised Discussion:

Crucially, our system establishes a programmable, non-equilibrium morphogenesis pathway that fundamentally differs from classical field-induced structuring phenomena, such as the Rosensweig instability in ferrofluids[59] or the static-field-driven bottom-up assembly of pillars from sedimented microparticles[43]. While these equilibrium or quasi-equilibrium processes are limited to forming structures with low aspect ratios, our synergistic use of static and oscillating fields drives continuous, energy-dissipative growth from the substrate. This enables the creation of segmented, high-aspect-ratio architectures not constrained by the static force balances that cap conventional assemblies. Furthermore, this dynamic growth phase is seamlessly integrated with post-assembly functionality. The resulting structure can be transitioned into a stable, locked configuration, which could serve as a static sensor probe[60], and then dynamically re-actuated on demand. Thus, within a single experimental setup, identical field modalities first govern the bottom-up assembly, can then be reconfigured to stabilize the structure, and can subsequently be used to actuate the entire architecture as a coherent, functional entity for tasks such as fluid manipulation.

The following references have been added to the main text:

43. Liu, X. et al. Magnetic field–driven particle assembly and jamming for bistable memory and response plasticity. Sci. Adv. 8, eadc9394 (2022).

59. Cowley, M.D., Rosensweig R.E. Interfacial stability of a ferromagnetic fluid. J. Fluid Mech. 30, 671-+ (1967).

60. Han, J. et al. Actuation-enhanced multifunctional sensing and information recognition by magnetic artificial cilia arrays. Proc. Natl. Acad. Sci. U. S. A. 120, e2308301120 (2023).

3. The phase boundaries in the diagram in Fig. 2f show a "deep" around 25Hz. What is special about this frequency. Could it be related to the critical frequency when particles develop lag with respect to the external field? It seems to be the case. It would be instructive to normalize these frequencies by that critical frequency to bring some sort of generalization of the reported results. The same comment is valid for the rest of the figures.

Response: We sincerely thank the reviewer for this insightful and constructive comment regarding the "deep" in the phase diagram (Fig. 2f) and the suggestion to examine frequency normalization. The reviewer's observation prompted us to conduct a more systematic

investigation, which has significantly strengthened our analysis and the generality of our conclusions.

1. Regarding the "deep" in the phase diagram:

We thank the reviewer for prompting a closer examination of the phase boundary. The question regarding the "deep" near 25 Hz led us to revisit the experimental parameter space with higher resolution, particularly at low amplitude ratios ($\gamma = B_x / B_z$). This refined analysis revealed that our original diagram did not fully represent the system's behavior when the horizontal driving field is weak. We have therefore updated the phase diagram (Revised Fig. 2f) to explicitly include the region where effective growth does not occur. Specifically, we find that for $\gamma < 1$, the transverse field strength is too weak to drive the sustained vertical growth of the M-Seaweed structure, regardless of the driving frequency. This non-growing regime is now clearly indicated by a purple shaded area in the revised figure, with the caption stating: "The purple shaded region denotes the parameter regime ($\gamma < 1$) where the transverse field strength is insufficient to initiate vertical growth." We appreciate the reviewer's observation, which helped us correct and clarify this aspect of the phase diagram.

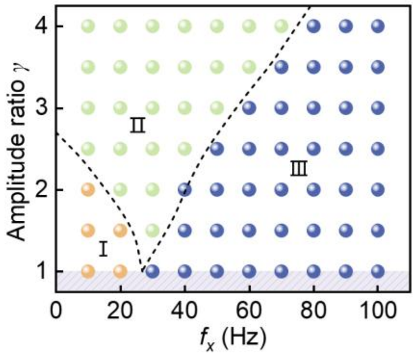

Rrevised Fig. 2f. Phase diagram categorizing the three magnetic structures as functions of amplitude ratio (γ) and oscillation frequencies (f_x), at a fixed vertical field strength (B_z = 5 mT). The purple shaded region denotes the parameter regime ($\gamma < 1$) where the transverse field strength is insufficient to initiate vertical growth.

Revised main text on page 7:

Above this frequency threshold, field-induced magnetic interactions drive spontaneous self-assembly, enabling stable vertical growth of M-Seaweed structures. Additionally, the phase diagram reveals a defined non-growing regime ($\gamma < 1$), marked by a purple shaded area, which delineates the parameter space where the transverse field strength is insufficient to initiate any sustained vertical growth.

2. Frequency normalization and generalized scaling:

We fully agree with the reviewer on the value of a normalized analysis for identifying universal scaling laws. To address this point, we performed a more systematic set of experiments to

explicitly define a characteristic system frequency and test the proposed normalization.

We extended our parameter survey by investigating three static field strengths ($B_z = 3$, 4, and 5 mT) across a range of amplitude ratios (γ), as detailed in the new Supplementary Fig. S12a. From this dataset, we extracted the characteristic onset (f_s) and critical (f_c) frequencies for each condition (B_z, γ), which are mapped against the control parameters in Supplementary Fig. S12b. Following the reviewer's suggestion, we then normalized the driving frequency (f_x) by the determined f_c for each condition (B_z, γ).

The resulting normalized plots (Supplementary Fig. S12c) confirm the utility of this approach. They reveal a clear scaling behavior: the operational window for M-Seaweed growth consistently converges to the normalized range of $0.4 < f_x / f_c < 2.0$. This provides a generalized scaling law that effectively predicts the permissible frequency “breadth” (the operational range) and its functional “depth” (impact on growth height) across different field parameters (B_z, γ), which was the insightful generalization sought.

In the revised manuscript, we retain the original, dimensioned diagrams in the main figures as they most directly map to the experimental parameters used in spatio-temporal control and our functional demonstrations (e.g., interfacial crossing). The new, comprehensive normalized analysis is presented in the supplement as the foundational physical scaling. We thank the reviewer for this suggestion, which has strengthened the generality and physical insight of our work.

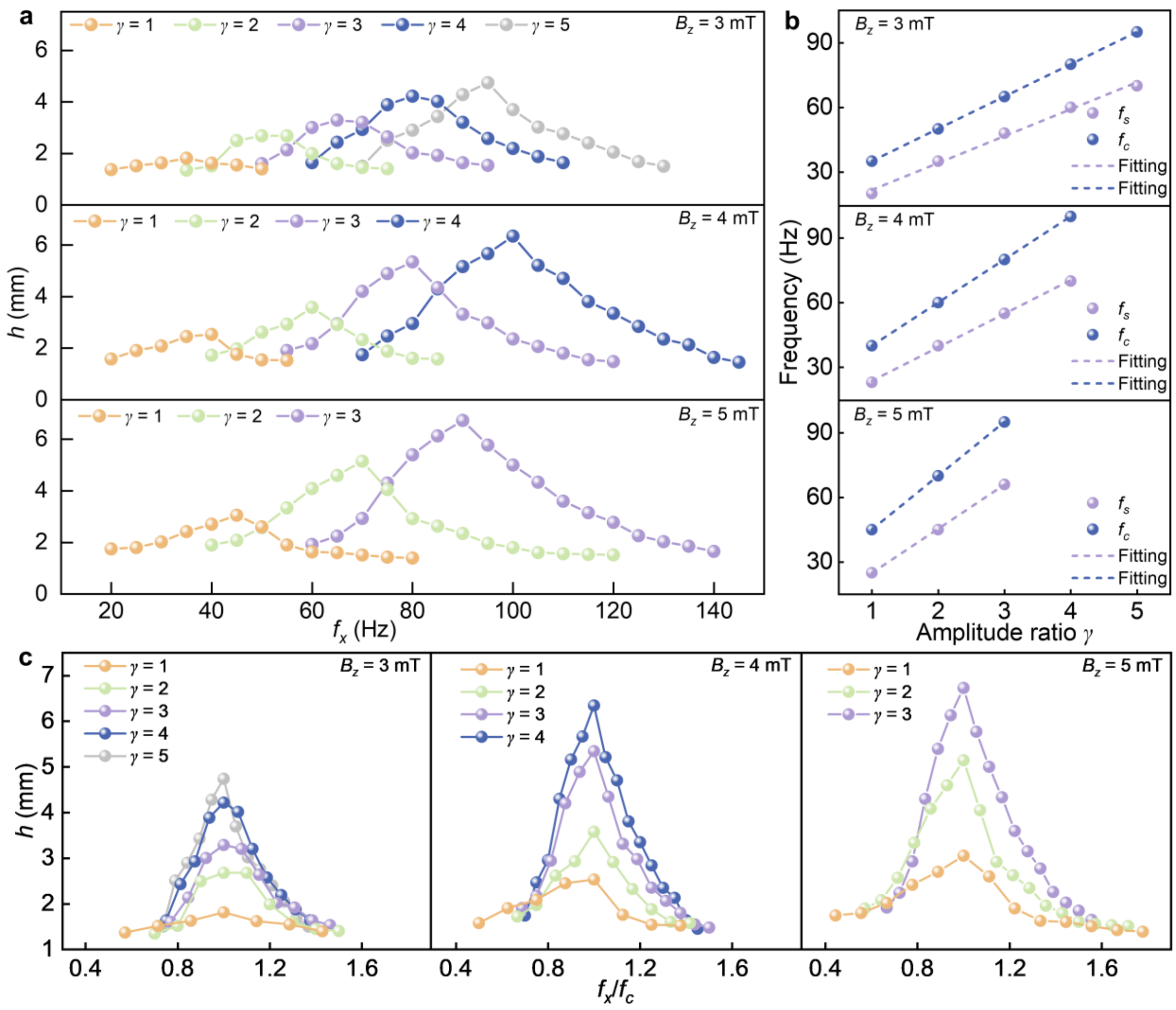

Fig. S12. Parametric study and normalized scaling of M-Seaweed growth. a M-Seaweed height as a function of driving frequency f_x, measured across static field strengths $B_z = 3$, 4, and 5 mT and a range of amplitude ratios γ. **b** Onset frequency (f_s) and critical frequency (f_c) extracted for each parameter set (B_z, γ). **c** Normalized diagrams obtained by scaling the driving frequency f_x with the corresponding f_c. The growth region collapses to the normalized window $0.4 < f_x / f_c < 2.0$, revealing a universal scaling law that links the extent of the operational frequency range to the resulting growth height across different field conditions.

Revised main text on page 10:

…These results not only confirm the regulatory role of vertical magnetic field strength (B_z) in structural morphology but also highlight the superior effectiveness of oscillating fields in facilitating vertically extended architectures.

To establish a generalized scaling relationship for this dynamic assembly, we systematically extended the parametric study to include the vertical field strength B_z (3, 4, and 5 mT) across a range of amplitude ratios γ (Supplementary Fig. S12a). The extracted onset (f_s) and critical (f_c) frequencies for each condition (B_z, γ) are mapped in Supplementary Fig. S12b, illustrating their systematic dependence on the control parameters. Normalizing the driving frequency f_x by its

corresponding f_c reveals a clear scaling behavior: the operational window for M-Seaweed growth collapses to the normalized range of $0.4 < f_x / f_c < 2.0$ (Supplementary Fig. S12c). This generalized scaling law links the extent of the operational frequency range to the resulting growth height across different field conditions, providing a universal criterion for this non-equilibrium morphogenesis.

Beyond magnetic field effects, we systematically examined three non-magnetic parameters governing vertical M-Seaweed growth…

4. The observed patterns (similarly to previously reported systems using a similar approach) are results of the interplay between hydrodynamic and time-averaged magnetic interactions, rather than just "the interplay between a high-frequency horizontal oscillating field and a static vertical field… ". Some intuitive insights into how frequency changes that interplay (and as a result the whole dynamics of the system) would be valuable for the reader. For instance, rapid oscillations (above critical frequency where particles develop a phase lag with the field) probably lead to the suppression of the hydrodynamic interactions and time-averaged magnetic interactions become dominant in the structure formation, etc.

Response: We sincerely thank the reviewer for this insightful and constructive comment, which significantly sharpens the physical description of our system's assembly mechanism. We fully agree that the dynamics are fundamentally governed by the interplay between hydrodynamic and time-averaged magnetic interactions, as mediated by the applied magnetic fields. Our original description focusing on the "interplay between the oscillating and static fields" was intended to describe the external actuation method that gives rise to these underlying interactions. The reviewer's suggestion to explicitly frame the mechanism in terms of hydrodynamic and time-averaged magnetic interactions provides a more fundamental and intuitive physical picture, which we believe will greatly benefit the readers.

As suggested, we have substantially revised the "Growth mechanisms and segmented morphology" section to frame the entire growth process within this fundamental physical picture. We now explicitly describe how frequency acts as the control parameter that shifts the balance from hydrodynamic interaction-dominated (low frequency, causing tilt and fragmentation) to time-averaged magnetic interaction-dominated (high frequency, enabling vertical growth) regimes. This provides an intuitive, general insight into the system dynamics.

Furthermore, to address the reviewer's earlier point regarding the need to draw deeper insights from our supplementary analysis and simulations into the main text, we have also integrated the key implications of those studies throughout this revised section. The revised section now explicitly connects the phase-lag analysis (Supplementary Note S1) to the onset of time-averaged interactions, leverages the collision-growth model (Supplementary Note S2) to explain growth kinetics, and uses the resonance (Supplementary Note S3) and coupled-oscillator (Supplementary Note S4) analyses to explain the segmented morphology and its dynamic coordination. These integrations ensure the main text now provides a physics-driven narrative

that moves beyond description to elucidate the underlying principles governing M-Seaweed morphogenesis.

Revised section of "Growth mechanisms and segmented morphology"

The vertical growth of M-Seaweed architectures emerges from a dual-field strategy that exploits the frequency-dependent competition between time-averaged magnetic and hydrodynamic interactions to overcome gravitational sedimentation and shear-induced fragmentation. This non-equilibrium self-assembly is governed by this interplay, tuned by the combined static (B_z) and oscillating (B_x) fields. The system exhibits a fundamental frequency-dependent transition: at low frequencies, magnetic units closely follow the field, generating large-amplitude motion that excites strong, long-range hydrodynamic interactions, leading to lateral instabilities and tilt. Above a frequency threshold, the rapid oscillation averages out the transverse magnetic interaction. This shift suppresses hydrodynamic interaction and makes time-averaged magnetic interactions dominant, which reinforce alignment and attraction along the static B_z axis. The resulting balance provides (i) dynamic stabilization (suppressed hydrodynamic interaction reduces lateral fluctuations) and (ii) directional guidance (enhanced time-averaged magnetic interaction promotes vertical dipole-dipole cohesion), enabling sustained vertical growth.

To elucidate this mechanism, we conducted a detailed micro-scale analysis. Combined simulations and experiments across frequencies (30–90 Hz) show high consistency (Fig. 4a, b and Supplementary Movie 6). At lower frequencies (30 and 50 Hz), hydrodynamic interaction-dominated dynamics cause magnetic pillars to undergo cyclic fragmentation and reorganization, forming tilted, X-shaped structures that appeared as staggered stacks in superimposed images (Fig. 4c). Quantitative analysis confirms an inverse relationship between frequency and tilt angle (Fig. 4d): low frequencies favor horizontally expanded assemblies (purple region), while high frequencies (70 and 90 Hz), where time-averaged magnetic interactions dominate, promote vertical elongation (green region). Furthermore, high-frequency conditions generate more, smaller assemblies (Fig. 4e), an outcome that arises from the competition between the driving and resisting torques[45]. This configuration promotes the collision-induced merging key to high-aspect-ratio M-Seaweed growth.

Based on the above analyses, we present a schematic of the dynamic assembly process for the M-Seaweed architectures (Fig. 4f). In the high-frequency regime ($f_x > f_s$), the transverse magnetic moment component exhibits a decaying response, which is characterized by a frequency-dependent phase lag relative to the driving field (Supplementary Fig. S15 and Supplementary Note S1). This dynamic response underpins the time-averaged magnetic interaction-dominated state, suppressing large-scale oscillations and governing the nucleation of transient structures (Fig. 4f(ii)). This process is further corroborated by our simulations, which capture the vertical growth dynamics of the assembling particles (Supplementary Fig. S16). Under high-frequency excitation, the particle-assembled primary structures undergo sustained low-amplitude vibrations, where their inherent structural asymmetries give rise to asymmetric flow fields

through the fluid-structure interaction near the substrate[46,47]. The resulting flow asymmetry produces a net lateral driving force that propels the structures into directed translational motion (Fig. 4f(iii) and top view in Supplementary Fig. S17), comparable to the wobbling of nanoeels induced by structural oscillation[48]. This autonomous mobility facilitates collision-induced coalescence between neighboring architectures (Fig. 4f(iv) and Supplementary Fig. S17, S18). Through successive collision and merging events, the system achieves progressive vertical elongation of the structural units, ultimately leading to the formation of dynamically stabilized M-Seaweed assemblies.

Guided by these mechanistic insights, we developed a frequency-modulated vertical growth model, which describes how the frequency-dependent collision-coalescence governs the distinct growth dynamics (e.g., growth velocity and final height) of M-Seaweed structures (Supplementary Note S2). The time-lapse trajectory overlays visualize the distinct microscopic collision-fusion activity patterns at different frequencies (Supplementary Fig. S19). Crucially, the net decrease in areal density from initial to equilibrium state, which peaks at the critical frequency (Supplementary Fig. S20), directly quantifies the resultant trend of structural consolidation across frequencies. By integrating this model with the experimental characterization of vertical growth below and above the critical frequency (Supplementary Fig. S21a-e), we established the frequency-dependent exponential scaling laws governing the assembly (Supplementary Fig. S21f). The results reveal that dynamic equilibration accelerates at subcritical frequencies, corresponding to reduced growth capability, whereas frequencies exceeding the critical value sustain stable equilibration timescales (Supplementary Fig. S22a-c). Moreover, the average vertical growth velocity follows an inverse frequency dependence, with a distinct growth-phase transition emerging at the critical frequency (Supplementary Fig. S22d-f).

Notably, the vertical extension of M-Seaweed structures exhibits a unique segmented morphology, where the superimposed images clearly reveal a series of vertically stacked X-shaped units (Fig. 4g, h, Supplementary Fig. S23, and Supplementary Movie 6), distinct from continuous pillar-like architectures. As the structure elongates vertically, high-frequency excitation induces progressive nodal formation along its longitudinal axis, driven by a dynamic mismatch between relatively stabilized basal anchoring and inertia-dominated apical oscillations (Fig. 4f(vi)). This behavior resembles the beating motion of magnetically driven artificial microswimmers[49] or elastic filaments[50], where internal stress propagation generates bending waves toward the tip. During elongation, its natural frequency decreases until it matches the driving frequency, triggering resonance. This excites higher-order vibration modes that concentrate shear stress (τ_{shear}) at nodal points, where τ_{shear} exceeds the magnetic adhesion strength (τ_{adh}), resulting in localized shear banding and subsequent segmentation into shorter sections[51]. These segments subsequently stabilize by detuning from resonance (Supplementary Note S3), which reveals a general dynamic adaptation strategy: the growing structure self-organizes into discrete units to avoid destructive resonance, thereby maintaining mechanical stability under persistent external actuation. Throughout this evolution, each stabilized segment

acts as an independent magnetic domain, exhibiting local high-frequency oscillations phase-locked to the external field while displaying inter-segment phase desynchronization due to mechanical decoupling (Fig. 4i-k). Despite local decoupling, long-range magnetic interactions maintain global coupling among segments (Supplementary Note S4). This results in a design principle of localized mechanical flexibility combined with global magnetic coherence, enabling the architecture to withstand localized fluid disturbances while maintaining its overall structural integrity.

The following Supplementary Figures (S19 and S20) have been added to the revised manuscript, which respectively visualize the distinct microscopic collision-fusion activity patterns at different frequencies and quantify the resultant trend of structural consolidation across frequencies.

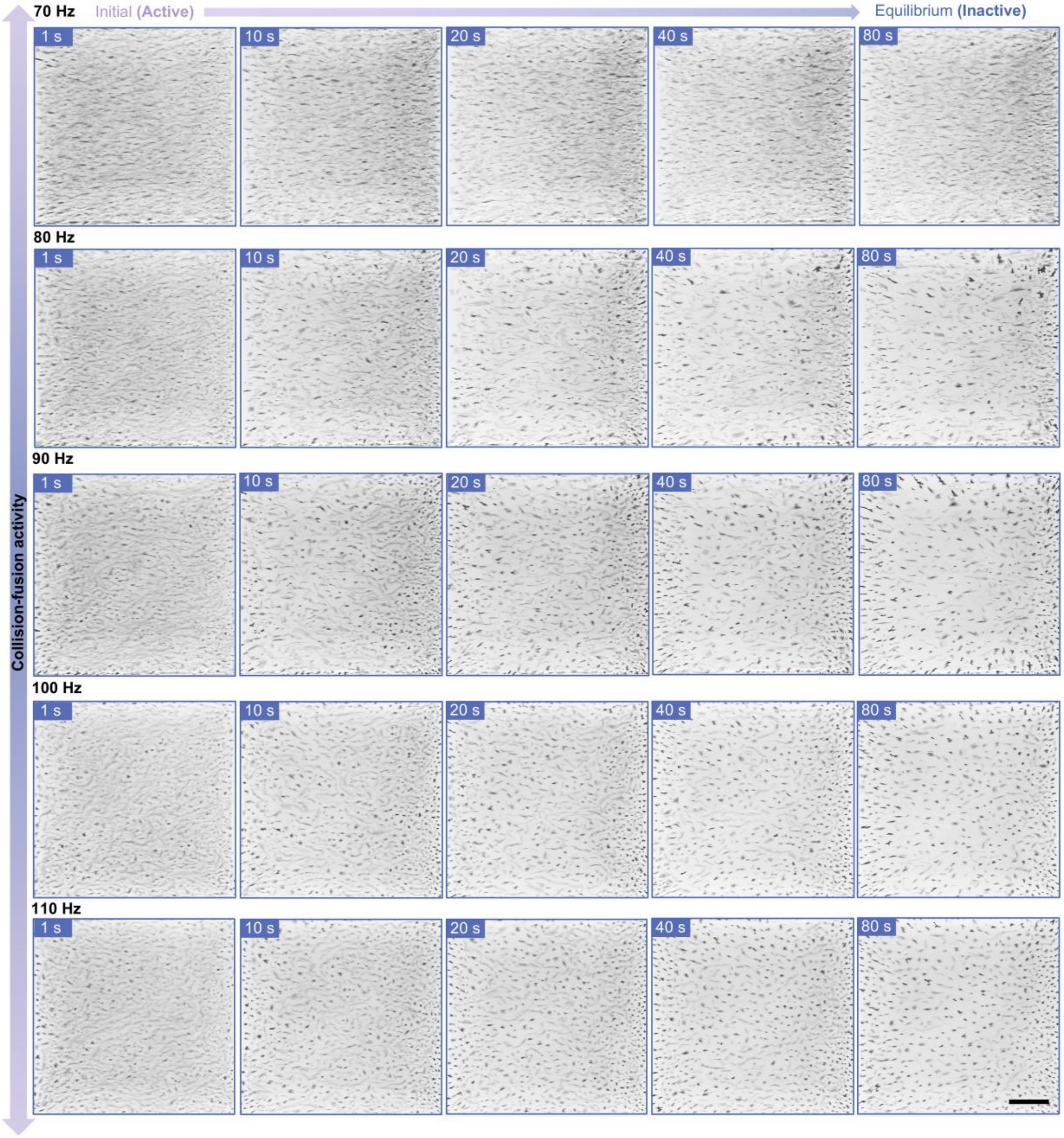

Fig. S19. Time-lapse trajectory overlays of M-Seaweed units at varying frequencies, illustrating the evolution of microscopic activity (γ = 3, B_z = 5 mT). Superimposed images (60 frames over 1 second) of M-Seaweed units at different time points (1 s, 10 s, 20 s, 40 s, and 80 s after magnetic field application) under driving frequencies of 70 Hz, 80 Hz, 90 Hz, 100 Hz, and 110 Hz. The trajectories visualize the distinct microscopic collision-fusion activity patterns, transitioning from the initial response to a dynamic equilibrium state. Scale bar, 2 mm.

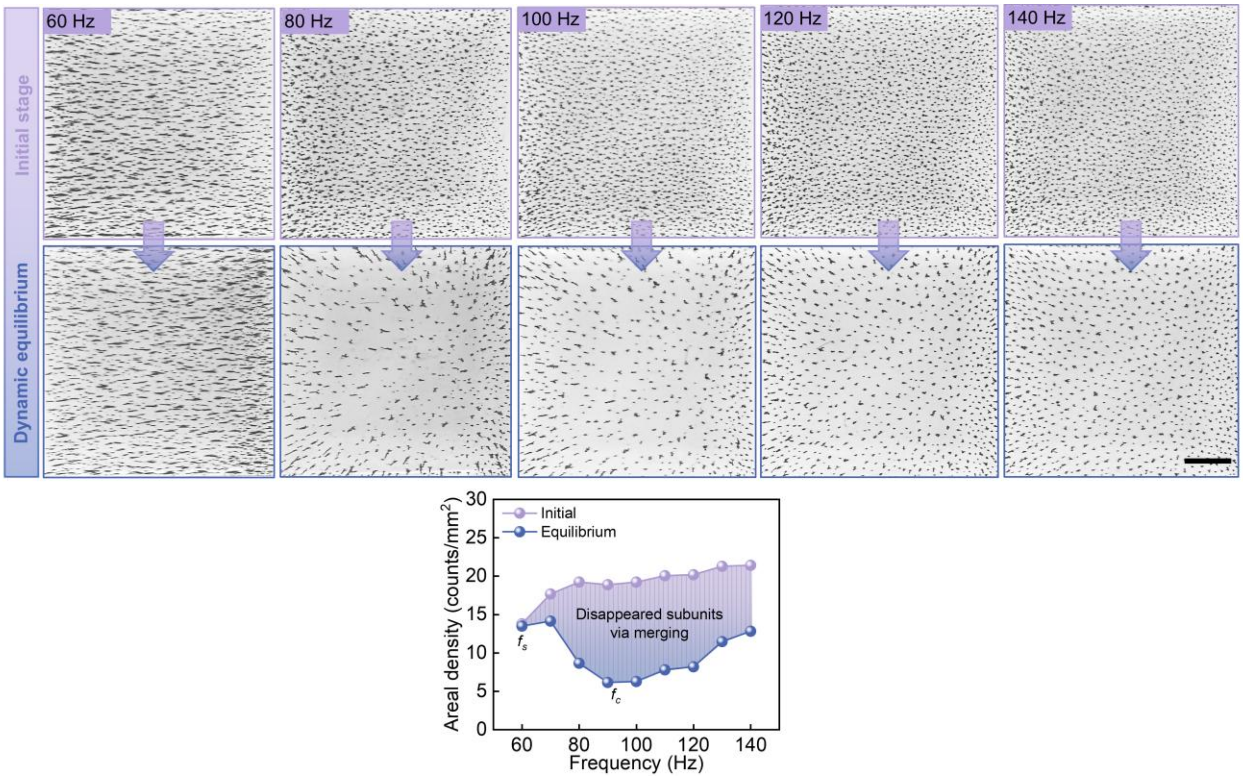

Fig. S20. Evolution of M-Seaweed unit area density across frequencies, quantifying the phase transition dynamics. Statistical analysis of the area density of magnetic structural units at the moment of magnetic field application (initial state) and at dynamic equilibrium (final state) across a frequency spectrum from 60 Hz to 140 Hz. The data reveal a pronounced reduction in unit density precisely at the critical frequency ($\gamma = 3$, $B_z = 5$ mT), providing direct evidence for the occurrence of high-frequency collision-fusion events at this threshold, which drives the M-Seaweed growth. Scale bar, 2 mm.

The following references have been added to the main text:

45. Gao, Y., Hulsen M.A., Kang T.G., den Toonder J.M.J. Numerical and experimental study of a rotating magnetic particle chain in a viscous fluid. Phys. Rev. E. 86, 041503 (2012).

46. Lauga, E., Powers T.R. The hydrodynamics of swimming microorganisms. Rep. Prog. Phys 72, 096601 (2009).

47. Jang, B. et al. Undulatory locomotion of magnetic multilink nanoswimmers. Nano Lett. 15, 4829-4833 (2015).

48. Mushtaq, F. et al. Motile piezoelectric nanoeels for targeted drug delivery. Adv. Funct. Mater. 29, 1808135 (2019).

49. Dreyfus, R., Baudry J., Roper M.L., Fermigier M., Stone H.A., Bibette J. Microscopic artificial swimmers. Nature 437, 862-865 (2005).

50. Zheng, E., Brandenbourger M., Robinet L., Schall P., Lerner E., Coulais C. Self-oscillation and synchronization transitions in elastoactive structures. Phys. Rev. Lett. 130, 178202 (2023).

51. Cecconi, F., Puglisi A., Viale M., Lucente D. Active-like dynamics of worm-like chains driven by an external traveling-wave force. Soft Matter 21, 5138-5152 (2025).

5. What is f_s in the experiment and how does it relate to the cutoff frequency (critical frequency),f_c ?

Response: We thank the reviewer for raising this point for clarification. In our experimental system, f_s (the onset frequency) is defined as the driving frequency threshold above which sustained vertical growth of the M-Seaweed structure is initiated. It marks the transition boundary in the phase diagram (Phase II to Phase III) where field-induced interactions overcome the transient, abortive growth observed at slightly lower frequencies (described in the cited Supplementary Fig. S7 and Movie 4). Above this frequency threshold (f_s), the magnetic and hydrodynamic interactions become sufficiently coordinated to drive the spontaneous, persistent, bottom-up assembly that characterizes the M-Seaweed morphology.

We have clarified this definition in the revised manuscript where this threshold is first mentioned (now marked as above this frequency threshold (f_s)) and thank the reviewer for prompting this important specification.

Revised main text on page 7:

Near the Phase II-III transition boundary, a transient growth phenomenon occurs: adjacent pillars fuse temporarily with the primary structure, then abruptly detach, ultimately leading to a cessation of structural evolution (Supplementary Fig. S7 and Supplementary Movie 4). Above this frequency threshold (f_s), field-induced magnetic interactions drive spontaneous self-assembly, enabling stable vertical growth of M-Seaweed structures.

Regarding the relationship between f_s and f_c:

As detailed in the newly added Supplementary Fig. S12b, both f_s and f_c have been systematically mapped as functions of the two key control parameters: the static field strength B_z and the amplitude ratio γ. The relationship between them is thus defined by their shared and systematic dependence on these parameters, as visualized in this figure.

The figure demonstrates that f_s and f_c exhibit correlated trends, as both increase with increasing B_z and γ. This parallel dependence indicates that the frequency window for stable growth (bounded by f_s below and f_c above) shifts coherently across the parameter space while preserving their relative spacing. We thank the reviewer for this insightful question again, which has

prompted a clearer exposition of this relationship, and we have incorporated the corresponding analysis into the revised manuscript.

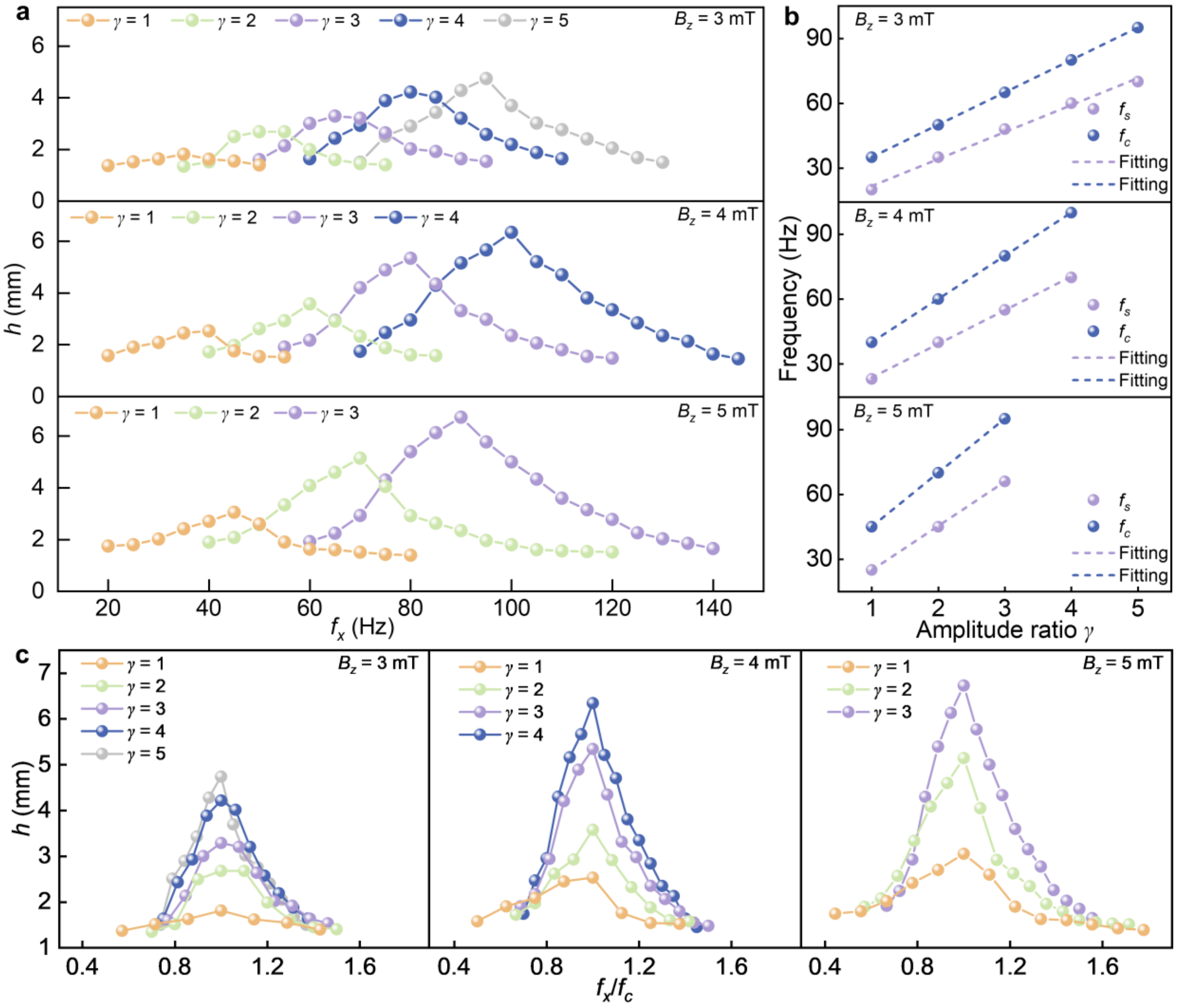

Fig. S12. Parametric study and normalized scaling of M-Seaweed growth. a M-Seaweed height as a function of driving frequency f_x, measured across static field strengths $B_z = 3, 4$, and 5 mT and a range of amplitude ratios γ. **b** Onset frequency (f_s) and critical frequency (f_c) extracted for each parameter set (B_z, γ). **c** Normalized diagrams obtained by scaling the driving frequency f_x with the corresponding f_c. The growth region collapses to the normalized window $0.4 < f_x / f_c < 2.0$, revealing a universal scaling law that links the extent of the operational frequency range to the resulting growth height across different field conditions.

Revised main text on page 10:

…These results not only confirm the regulatory role of vertical magnetic field strength (B_z) in structural morphology but also highlight the superior effectiveness of oscillating fields in facilitating vertically extended architectures.

To establish a generalized scaling relationship for this dynamic assembly, we systematically

extended the parametric study to include the vertical field strength B_z (3, 4, and 5 mT) across a range of amplitude ratios γ (Supplementary Fig. S12a). The extracted onset (f_s) and critical (f_c) frequencies for each condition (B_z, γ) are mapped in Supplementary Fig. S12b, illustrating their systematic dependence on the control parameters. Normalizing the driving frequency f_x by its corresponding f_c reveals a clear scaling behavior: the operational window for M-Seaweed growth collapses to the normalized range of $0.4 < f_x / f_c < 2.0$ (Supplementary Fig. S12c). This generalized scaling law links the extent of the operational frequency range to the resulting growth height across different field conditions, providing a universal criterion for this non-equilibrium morphogenesis.

Beyond magnetic field effects, we systematically examined three non-magnetic parameters governing vertical M-Seaweed growth…

6. What are "supercritical frequencies" introduced on page 15?

Response: We thank the reviewer for pointing this out. The reviewer is correct that the term "supercritical frequencies" may not be the standard or most precise terminology in this context. Our intended meaning is simply "frequencies above the critical frequency" of the structure. To avoid any potential confusion, we have revised the manuscript to replace "supercritical frequencies" with the clearer description "frequencies exceeding the critical value" (on page 15). We believe this change enhances the clarity and accuracy of our presentation.

Revised main text on page 15:

The results reveal that dynamic equilibration accelerates at subcritical frequencies, corresponding to reduced growth capability, whereas ~~supercritical frequencies~~ frequencies exceeding the critical value sustain stable equilibration timescales (Supplementary Fig. S19a-c).

7. On page 22 authors claim that the system "… sustained high-turbulence flows below 3Hz". I suspect the authors mean "chaotic flows" instead of "turbulence". To support the claim of the turbulence, authors should provide the energy spectra for the flows with characteristic exponent analysis.

Response: We appreciate the reviewer's expert comment regarding the term "turbulence", , which prompted us to refine our description of the flow state. To ensure our language is both precise and strictly aligned with our experimental observations, we have revised the phrasing to state "…progressing to sustained, **highly disturbed** flows beyond 3 Hz." We appreciate the reviewer's attention to terminological precision, which has helped enhance the clarity of our manuscript.

Revised main text on page 22:

Increasing the amplitude to 2 mT induced structural metamorphosis, initiating with tip detachment at 1 Hz and progressing to sustained ~~high-turbulence~~, highly disturbed flows beyond 3 Hz.

Reviewer #2:

Review for:

Gravity-resisting morphogenesis of magnetic seaweed-like structures

In this paper, the authors propose an innovation in the common approach for magnetic field assisted self-assembly of colloidal particles. The authors expose the particles to a complex field that features a permanent component in the Z direction and a fluctuating component on the XY plane.

The resultant structure is a linear array of particles with periodic kinks they dub an “M-Seaweed”. The addition of the fluctuating component enables the structure to reach far greater heights than one that forms under a permanent uniaxial field.

An additional advantage of modulation in the permanent and fluctuating components is for modification of the M-Seaweeds properties. Several examples include an overall structure transition between three distinct morphologies, height and density modifications and the creation of a controlled slanted angle between the structures and the substrate.

The manuscript is rich with information, giving a full description of the “M-Seaweed” structures and their potential.

Response: We thank the reviewer for the comment.

The following notes arose while reading the manuscript and require improvements from the authors:

MAJOR:

At its current state, the paper reads as a part of an internal conversation inside the self-assembly field. The broader implications of this work beyond its immediate community do not arise from the experiments themselves, and receive very little mention during the discussion. While there are some technical advances, It is unclear what the major impact of the work is. This has not been demonstrated. The authors are required to meaningfully link their work to the broader scientific world, where one would expect a convincing demonstration of the work.

Response: We sincerely thank the reviewer for the insightful and constructive comment regarding the broader implications of our work. We fully agree that demonstrating the impact of a technical advance beyond its immediate field is crucial. In direct response, we have significantly reframed the narrative of our manuscript and, most importantly, conducted a new, functionally demonstrative experiment that moves beyond structural novelty to showcase a tangible, function-oriented capability.

Our revisions are built upon three core actions:

1. Performed a new, functionally demonstrative experiment: We have integrated a function-oriented experiment that moves beyond structural formation to show active, targeted chemical functionality. Magnetic particles were decorated with the enzyme horseradish peroxidase (HRP) and assembled into M-Seaweed in a lower perfluorohexane phase. The upper aqueous phase was replaced with a solution containing H_2O_2 and the chromogenic substrate ABTS. As shown in the new **Fig. 7f** and **Supplementary Movie 14** (now included in the Results section), the field-guided vertical growth of the M-Seaweed across the immiscible interface delivers the HRP-coated particles into the upper phase, triggering a localized enzymatic reaction. This results in a clear, macroscopic color change (green) in the upper solution, providing direct visual evidence of spatiotemporally controlled material transport and reaction initiation across a boundary. This experiment transforms the M-Seaweed from a structural curiosity into a programmable micro-transporter and interfacial reaction initiator.

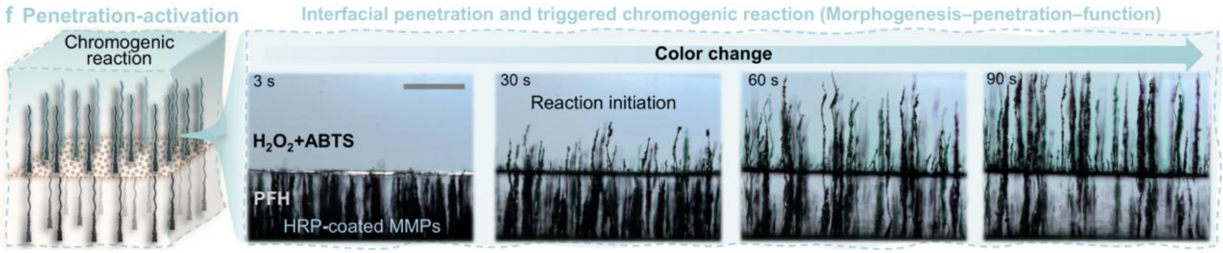

Fig. 7f | Localized chromogenic reaction triggered by the interfacial penetration of M-Seaweed structures, using HRP-modified particles and an H_2O_2/ABTS upper phase. Scale bar, 2 mm.

2. Reframed the Introduction with a broader, bio-inspired vision: We have completely restructured the Introduction to begin not with the technical field of self-assembly, but with the broader scientific challenge inspired by nature. We now open with concrete examples of biological systems (pollen tubes, fungal hyphae, bacterial pili) that master the art of growing high-aspect-ratio structures across interfaces to perform targeted delivery and localized reactions. This articulates a well-defined and persuasive motivation that is fundamentally interdisciplinary, setting a strong framework for the study. We then position field-driven colloidal self-assembly as a promising tool to mimic this capability, critically analyze the current limitations (confinement to 2D, inability for sustained 3D growth across interfaces), and

explicitly state the unmet need our work addresses: creating a synthetic system that integrates continuous growth, interface penetration, and triggered function.

In addition, we have revised the article's title, abstract, Figure 1, Figure 7, and Section 6 of the results accordingly.

Revised Title:

Gravity-resisting morphogenesis of magnetic seaweed-like structures for functional delivery across liquid-liquid interfaces

Revised Abstract:

The ability to actively traverse phase boundaries to perform functions, a hallmark of many biological systems, inspires the design of synthetic counterparts, which hold promise for applications in targeted delivery and adaptive sensing. However, achieving such systems, particularly within stratified liquids, presents a significant challenge, demanding dynamic architectures that combine continuous gravity-resisting growth, interfacial penetration, and post-crossing action. Here, we report "Magnetic Seaweed" (M-Seaweed), a field-driven microparticle system that integrates this entire functional cascade. Using a synergistic combination of static and oscillating magnetic fields, we guide magnetic microparticles to assemble into dynamic, high-aspect-ratio, seaweed-like structures that exhibit gravity-resisting growth upward from a substrate. These dynamic architectures are reconfigurable, stabilizable, and programmably actuatable, and can be steered to penetrate a perfluorohexane-water interface. By functionalizing particles with an enzyme, the growing M-Seaweed structures act as micro-transporters, delivering catalytic activity across the interface to trigger a localized, macroscopic chromogenic reaction upon penetration. Our work establishes a field-driven morphogenesis-to-function paradigm, bridging structural growth, phase-boundary penetration, and triggered function toward intelligent microsystems for targeted delivery and adaptive sensing.

Revised Introduction:

The ability to actively traverse phase boundaries and perform functions on demand is a hallmark of many biological systems. This capability enables precise operations such as targeted material delivery, interfacial sensing, and localized reaction initiation, processes that are fundamental to life and highly desirable in synthetic microsystems. For example, pollen tubes directionally grow through tissues to deliver genetic material[1], fungal hyphae extend across air gaps to secrete enzymes at new substrates[2], and bacterial pili probe surfaces to facilitate adhesion and DNA uptake[3]. Translating this cross-interface function execution into synthetic platforms, particularly across stratified liquid environments, requires the creation of dynamic structures that can directionally grow with gravity-resisting capability, penetrate defined interfaces, and subsequently trigger specific actions. Achieving such an integrated system would open new

avenues in areas like targeted therapeutic delivery, compartmentalized catalysis, and adaptive environmental sensing.

Field-driven self-assembly of colloidal particles offers a powerful route to mimic such natural systems. By harnessing external fields (e.g., magnetic[4-7], electric[8-11], acoustic[12-14], optical[15-17], or chemical[18]), researchers can guide components into organized structures. These approaches are broadly classified into two regimes: static self-assembly and dynamic self-assembly[19,20]. The former is governed by equilibrium thermodynamics and yields stable architectures (e.g., crystalline superlattices) but inherently lacks the dynamic responsiveness required for continuous growth or interfacial penetration. In contrast, the latter operates under non-equilibrium conditions, dissipating energy to form transient, reconfigurable structures capable of complex behaviors such as collective motion and task execution[20,21]. This paradigm has enabled remarkable advances in microrobotics[22-25], reconfigurable metamaterials[26], adaptive chemical networks[27], and biomedical engineering[28-32].

Although dynamic colloidal systems have achieved sophisticated organization, their behavior remains largely confined to two-dimensional or quasi-two-dimensional landscapes, forming in-plane vortices[22,33-35], chains[11,23,36], or ribbon-like assemblies[24,37-39] on planar substrates or at liquid–air interfaces. These configurations inherently lack the out-of-plane structural integrity and the fundamental assembly pathways needed for sustained vertical extension. Even when three-dimensional dynamic assemblies are achieved, they typically either form within a bulk phase (e.g., from particles suspended in a polymer solution[40-42]) rather than growing upward from a substrate while resisting gravity, or they lack the robustness and control required to penetrate a distinct liquid phase. Therefore, the integrated realization of continuous gravity-resisting growth, controlled interfacial penetration, and subsequent functional triggering within one platform remains a systems-level challenge.

Here, we report “Magnetic Seaweed” (M-Seaweed): a field-driven microparticle system that achieves gravity-resisting morphogenesis of segmented seaweed-like structures, active interfacial penetration, and post-penetration functional triggering (Fig. 1). By synergistically combining static and oscillating magnetic fields, the system drives spontaneous sprouting-like growth, ultimately forming high-aspect-ratio architectures in dynamic equilibrium (Fig. 1a). These dynamic architectures are reconfigurable, stabilizable (i.e., lockable into static configurations), and programmably actuatable through field modulation (Fig. 1b). They can also be steered to penetrate a perfluorohexane (PFH)-water interface, thereby transferring the majority of the constituent microparticles from the substrate into the upper phase (Fig. 1c). Crucially, we demonstrate that this interfacial crossing can be functionally harnessed by decorating particles with the enzyme horseradish peroxidase (HRP): the growing M-Seaweed structures act as micro-transporters, delivering catalytic activity into a target phase containing H_2O_2 and ABTS (a chromogenic substrate) to trigger a localized, macroscopic color change upon penetration. Our work thus establishes a new paradigm for field-driven dynamic materials, bridging structural growth, phase boundary penetration, and on-demand functional execution,

and opening pathways toward intelligent microsystems capable of operating across stratified environments.

Revised Fig. 1

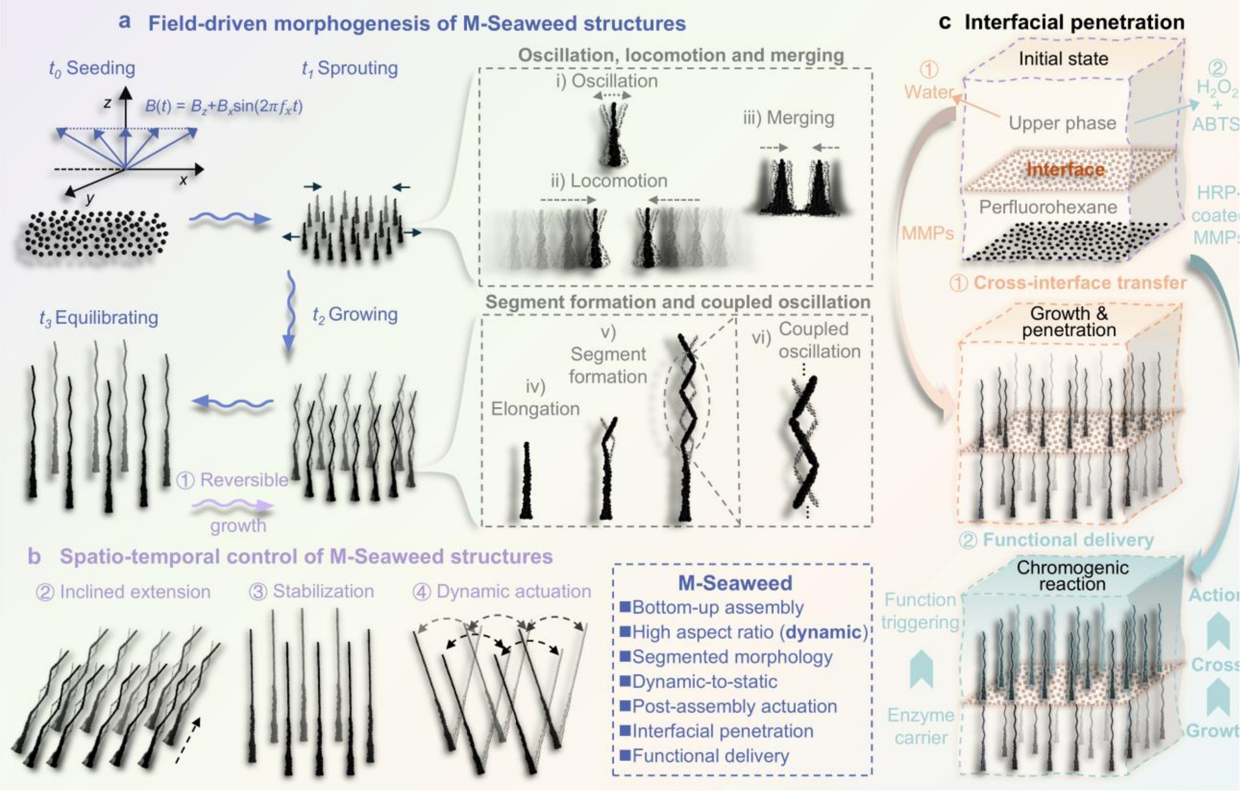

Fig. 1 | Magnetic field-modulated gravity-resisting morphogenesis, dynamic control, and functional delivery of M-Seaweed. a Dynamic self-assembly and vertical growth of M-Seaweed structures under the oscillating magnetic field, with key morphological transitions including locomotion, merging, elongation, and segment formation. **b** Programmable structural control via magnetic field modulation, demonstrating four operational modes: reversible growth, inclined extension, post-assembly stabilization, and dynamic actuation. **c** Interfacial penetration across a water/PFH interface (orange arrow), and the triggered chromogenic reaction using HRP-modified particles with an H_2O_2/ABTS upper phase (green arrow).

Revised section 6 of the Results

Interfacial penetration and functional delivery of M-Seaweed

To demonstrate the complete morphogenesis-to-function sequence (i.e., growth, interfacial penetration, and functional execution) of M-Seaweed, we employed an immiscible bilayer system of perfluorohexane (PFH) and an aqueous phase. Initially, we focused on its penetration

and transfer capabilities (Fig. 7a). Dried magnetic particles were dispersed in the lower PFH phase,…

…

To directly couple interfacial penetration with a tangible functional output, we engineered M-Seaweed to act as a biocatalytic micro-transporter. Particles were functionalized with horseradish peroxidase (HRP), and the upper aqueous phase was replaced with a solution containing H_2O_2 and the chromogenic substrate ABTS. Upon field-controlled penetration of the PFH/(H_2O_2-ABTS) interface, the HRP-loaded M-Seaweed delivered enzymatic activity into the target phase. This immediate delivery of the enzyme triggered a localized, macroscopic chromogenic reaction, manifesting as a sharp color change precisely co-localized with the penetration site (Fig. 7f and Supplementary Movie 14). This experiment demonstrates that interfacial penetration is not merely a physical transfer but can be programmably linked to a subsequent chemical function. It validates the complete morphogenesis-to-function paradigm: a field-driven structure can grow, penetrate a phase boundary, and upon arrival, execute a pre-defined task, thereby closing the loop from structural dynamics to embodied functionality.

Revised Fig. 7

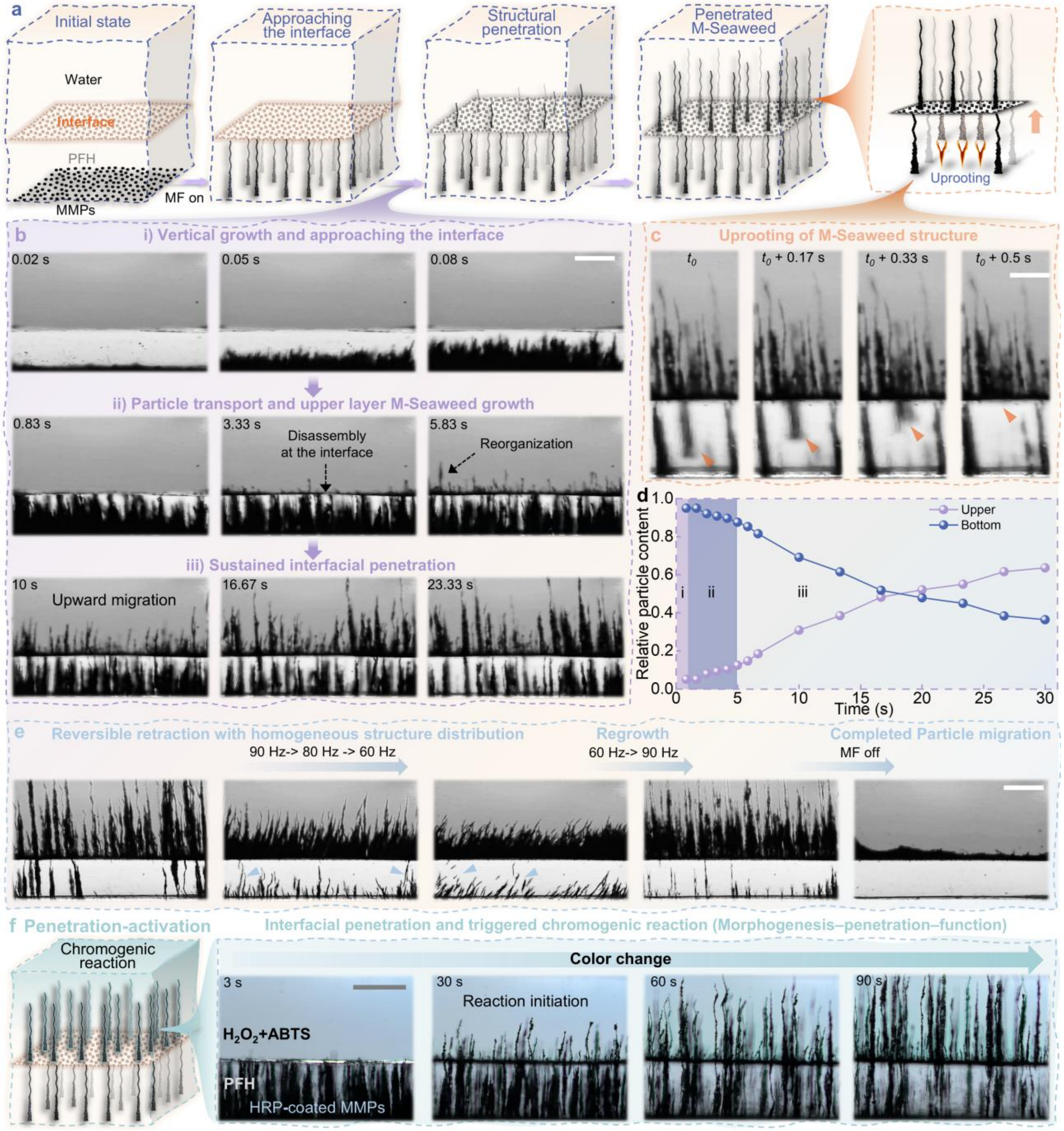

Fig.7 | Interfacial penetration and functional delivery of M-Seaweed. a Schematic of the M-Seaweed…, **b**… **e**…**f** Localized chromogenic reaction triggered by the interfacial penetration of M-Seaweed structures, using HRP-modified particles and an H_2O_2/ABTS upper phase. Scale bar, 2 mm.

3. Substantially expanded the Discussion to highlight impact: We have rewritten the Discussion to explicitly articulate the new paradigm enabled by our work and its potential ramifications across multiple fields. The new HRP experiment serves as the central proof-of-concept for this paradigm shift.

Summary of the major impact now clearly demonstrated:

The major impact of this work is now articulated as the establishment of a "morphogenesis-to-function" paradigm in active matter. We demonstrate a seamless integration of three capabilities previously disconnected in synthetic colloidal systems:

-Gravity-resisting 3D morphogenesis: Programmable growth of high-aspect-ratio, reconfigurable structures from a substrate, overcoming limitations related to gravitational collapse and instability.

-Active interfacial penetration: Directed crossing of an immiscible liquid-liquid interface by the growing structure itself.

-Functional execution: Using the structure as a vehicle to deliver a specific function (catalysis) at a precise location and time, triggered by the penetration event.

This integrated capability, demonstrated through the HRP experiment, directly links our magnetic control strategy to potential applications with broad relevance:

-Targeted delivery & microreactors: For spatiotemporally controlled delivery of catalysts, drugs, or reagents across biological or chemical barriers (e.g., tissue layers, emulsion droplets).

-Adaptive sensing & signal transduction: Converting a microscopic mechanical event (particle arrival) into an amplified, macroscopic chemical signal.

-Microrobotics in stratified environments: Informing the design of soft microrobots that can navigate and perform tasks across complex, multiphase media.

We believe these comprehensive revisions directly address the reviewer's concern by moving the manuscript beyond an "internal conversation" and positioning it as a cross-disciplinary advance that provides both a fundamental new strategy for dynamic self-assembly and a convincing demonstration of its utility for functional microsystems.

Revised Discussion:

This work introduces the Magnetic Seaweed (M-Seaweed) system, which achieves a bio-inspired integration of three-dimensional morphogenesis, active interfacial penetration, and triggered functional execution via field-driven dynamic self-assembly. By synergistically combining static and oscillating magnetic fields, we guide magnetic microparticles to form dynamic, high-aspect-

ratio structures that exhibit gravity resistance and reconfigurability; can be post-assembly stabilized or dynamically actuated; and are capable of field-guided penetration across an immiscible liquid-liquid interface for functional delivery. The principal advance reported herein thus represents a field-directed paradigm that fundamentally bridges the gap between structural morphogenesis and functional execution in active materials.

Our approach enables sustained, directional growth that bridges distinct phases, a capability that has been largely inaccessible to field-driven dynamic assemblies. Whereas prior systems, which often leverage time-averaged interactions from multi-axial[58], time-dependent fields to create complex patterns within a bulk fluid or at an interface, have largely been confined to two-dimensional organization or to three-dimensional aggregates within a single medium under negligible gravitational influence, they have generally lacked the capacity for persistent vertical extension from a substrate across interfacial boundaries. In contrast, the assembly of M-Seaweed applies this principle of field-programmed time-averaged interactions to a distinct paradigm: the bottom-up, persistent morphogenesis of structures directly from a dense particle layer against the dominant influence of gravity. These tuned interactions, mediated hydrodynamically and magnetically via the oscillating field frequency, simultaneously promote magnetic units bonding for elongation and confer sufficient structural stiffness to resist buckling under gravitational and hydrodynamic loads, enabling the growth of dynamic, high-aspect-ratio, segmented architectures not accessible in suspension-based or interface-confined systems.

Crucially, our system establishes a programmable, non-equilibrium morphogenesis pathway that fundamentally differs from classical field-induced structuring phenomena, such as the Rosensweig instability in ferrofluids[59] or the static-field-driven bottom-up assembly of pillars from sedimented microparticles[43]. While these equilibrium or quasi-equilibrium processes are limited to forming structures with low aspect ratios, our synergistic use of static and oscillating fields drives continuous, energy-dissipative growth from the substrate. This enables the creation of segmented, high-aspect-ratio architectures not constrained by the static force balances that cap conventional assemblies. Furthermore, this dynamic growth phase is seamlessly integrated with post-assembly functionality. The resulting structure can be transitioned into a stable, locked configuration, which could serve as a static sensor probe[60], and then dynamically re-actuated on demand. Thus, within a single experimental setup, identical field modalities first govern the bottom-up assembly, can then be reconfigured to stabilize the structure, and can subsequently be used to actuate the entire architecture as a coherent, functional entity for tasks such as fluid manipulation.

The functional utility of this platform is demonstrated by employing HRP-functionalized particles to execute a defined chemical task. This serves as a paradigm for controlled distribution of chemical reagents across an interface to drive heterogeneous reactions. Here, the field-guided growth process itself orchestrates a spatiotemporally controlled operation: the vertical extension precisely dictates the kinetics of interfacial crossing, transforming the M-Seaweed into a programmable microscale transporter that delivers the enzyme (HRP) into a compartment

containing its substrates (H_2O_2/ABTS). The subsequent triggering of a macroscopic colorimetric change provides direct evidence of a localized reaction initiated by this programmed reagent delivery. This demonstration highlights key functional principles: spatiotemporal targeting, where field parameters govern catalytic delivery across the boundary; amplified macroscopic output, whereby a microscale event yields a detectable signal; and operation in compartmentalized systems, enabling selective activation in one phase from another, a feature pertinent to multiphase reaction engineering. The fundamental strategy, field-guided material delivery across an interface to trigger a localized response, provides a versatile platform that could be extended to other systems, such as the tailored delivery of biomolecular cues (e.g., growth factors) across biological membranes for targeted biological modulation.

In summary, we have developed a colloidal system that integrates structural morphogenesis, phase-boundary penetration, and functional execution. The M-Seaweed platform thereby advances the field of active matter toward the creation of intelligent microsystems capable of growing, adapting, and performing precise tasks within multiphase environments.

MINOR:

There is significant imbalance between the “results” and “discussion” segments. While the results are comprehensive, the discussion is relatively information poor and serves mostly to present the study’s conclusions.

Furthermore, there are multiple claims presented in the results that are made without experimental or literary substantiation.

To remedy both issues in the same time, the authors should move the following claims to the discussion segment and include supporting citations (and/or present theoretical frameworks).

The claims are as follows:

- The various sentences scattered throughout the paper describing the advantages of the M-Seaweed should be combined in the discussion and presented in relation to existing structures.

Response: We thank the reviewer for the suggestion to consolidate the discussion of the M-Seaweed's advantages and present them in relation to existing structures. We agree that this strengthens the manuscript's narrative and contextualizes our advancement.

As suggested, we have thoroughly revised the Discussion section. The advantages of the M-Seaweed system, specifically its gravity-resisting vertical growth from a substrate, high-aspect-ratio segmented architecture, seamless integration of dynamic assembly with static stabilization and re-actuation, and capability for active interfacial penetration-activation, are now synthesized and presented cohesively in the revised Discussion.

Crucially, these advantages are explicitly framed in contrast to prior systems, as recommended. We now compare our platform to:

1. Classical field-driven dynamic assemblies (often operating in bulk or at interfaces), highlighting our system's unique capacity for persistent vertical extension from a substrate across phase boundaries.

2. Equilibrium field-induced structures, such as pillars formed via static-field-driven bottom-up assembly and spikes from the Rosensweig instability, emphasizing the fundamental distinction afforded by our non-equilibrium, programmable morphogenesis pathway which overcomes the low-aspect-ratio limitation of these classical phenomena.

We believe this restructured discussion directly addresses the reviewer's point, providing a clearer and more compelling narrative that positions the novelty and functional utility of the M-Seaweed within the broader field.

Revised Discussion:

This work introduces the Magnetic Seaweed (M-Seaweed) system, which achieves a bio-inspired integration of three-dimensional morphogenesis, active interfacial penetration, and triggered functional execution via field-driven dynamic self-assembly. By synergistically combining static and oscillating magnetic fields, we guide magnetic microparticles to form dynamic, high-aspect-ratio structures that exhibit gravity resistance and reconfigurability; can be post-assembly stabilized or dynamically actuated; and are capable of field-guided penetration across an immiscible liquid-liquid interface for functional delivery. The principal advance reported herein thus represents a field-directed paradigm that fundamentally bridges the gap between structural morphogenesis and functional execution in active materials.

Our approach enables sustained, directional growth that bridges distinct phases, a capability that has been largely inaccessible to field-driven dynamic assemblies. Whereas prior systems, which often leverage time-averaged interactions from multi-axial[58], time-dependent fields to create complex patterns within a bulk fluid or at an interface, have largely been confined to two-dimensional organization or to three-dimensional aggregates within a single medium under

negligible gravitational influence, they have generally lacked the capacity for persistent vertical extension from a substrate across interfacial boundaries. In contrast, the assembly of M-Seaweed applies this principle of field-programmed time-averaged interactions to a distinct paradigm: the bottom-up, persistent morphogenesis of structures directly from a dense particle layer against the dominant influence of gravity. These tuned interactions, mediated hydrodynamically and magnetically via the oscillating field frequency, simultaneously promote magnetic units bonding for elongation and confer sufficient structural stiffness to resist buckling under gravitational and hydrodynamic loads, enabling the growth of dynamic, high-aspect-ratio, segmented architectures not accessible in suspension-based or interface-confined systems.

Crucially, our system establishes a programmable, non-equilibrium morphogenesis pathway that fundamentally differs from classical field-induced structuring phenomena, such as the Rosensweig instability in ferrofluids[59] or the static-field-driven bottom-up assembly of pillars from sedimented microparticles[43]. While these equilibrium or quasi-equilibrium processes are limited to forming structures with low aspect ratios, our synergistic use of static and oscillating fields drives continuous, energy-dissipative growth from the substrate. This enables the creation of segmented, high-aspect-ratio architectures not constrained by the static force balances that cap conventional assemblies. Furthermore, this dynamic growth phase is seamlessly integrated with post-assembly functionality. The resulting structure can be transitioned into a stable, locked configuration, which could serve as a static sensor probe[60], and then dynamically re-actuated on demand. Thus, within a single experimental setup, identical field modalities first govern the bottom-up assembly, can then be reconfigured to stabilize the structure, and can subsequently be used to actuate the entire architecture as a coherent, functional entity for tasks such as fluid manipulation.

The functional utility of this platform is demonstrated by employing HRP-functionalized particles to execute a defined chemical task. This serves as a paradigm for controlled distribution of chemical reagents across an interface to drive heterogeneous reactions. Here, the field-guided growth process itself orchestrates a spatiotemporally controlled operation: the vertical extension precisely dictates the kinetics of interfacial crossing, transforming the M-Seaweed into a programmable microscale transporter that delivers the enzyme (HRP) into a compartment containing its substrates (H_2O_2/ABTS). The subsequent triggering of a macroscopic colorimetric change provides direct evidence of a localized reaction initiated by this programmed reagent delivery. This demonstration highlights key functional principles: spatiotemporal targeting, where field parameters govern catalytic delivery across the boundary; amplified macroscopic output, whereby a microscale event yields a detectable signal; and operation in compartmentalized systems, enabling selective activation in one phase from another, a feature pertinent to multiphase reaction engineering. The fundamental strategy, field-guided material delivery across an interface to trigger a localized response, provides a versatile platform that could be extended to other systems, such as the tailored delivery of biomolecular cues (e.g., growth factors) across biological membranes for targeted biological modulation.

In summary, we have developed a colloidal system that integrates structural morphogenesis, phase-boundary penetration, and functional execution. The M-Seaweed platform thereby advances the field of active matter toward the creation of intelligent microsystems capable of growing, adapting, and performing precise tasks within multiphase environments.

• "Growth mechanism and segmented morphology" subsection: Most of this subsection constitutes discussion and would benefit greatly from additional citations.

Response: We thank the reviewer for the valuable comment, and we fully agree that the "Growth mechanism and segmented morphology" subsection would benefit from additional citations to better situate our findings within the broader literature and strengthen the discussion.

In direct response to the reviewer's suggestion, we have thoroughly revised this subsection. The key enhancements are as follows:

1. Added citations to core mechanistic concepts: We have integrated relevant references at critical points explaining the frequency-dependent dynamic transition, non-equilibrium self-assembly, and the competition between hydrodynamic and time-averaged magnetic interactions. New citations (e.g., [45-47]) now provide a foundational context for the physical mechanisms observed in our work.

2. Provided references for analogies and comparisons: Where we draw parallels—such as comparing the autonomous motion of M-Seaweed structures to the wobbling of "nanoeels" or relating the segmented morphology to vibration modes in magnetically driven microswimmers or elastic structures—we have now cited representative studies (e.g., new citations [48-50]). This helps clarify the conceptual links between the phenomena in our system and those in other established physical systems.

3. Strengthened the theoretical background: In describing concepts like shear banding, resonance-driven segmentation, and mechanical decoupling, we have added citations to literature on soft matter dynamics and structural instabilities (e.g., new citation [51]). This provides a more robust theoretical foundation for our interpretation of the morphology formation mechanism.

We believe the revised text now more effectively connects our novel findings to the existing body of knowledge. Thank you again for this insightful feedback, which has been instrumental in improving the manuscript.

Revised section 3 of the Results

Growth mechanisms and segmented morphology

…

To elucidate this mechanism, … Furthermore, high-frequency conditions generate more, smaller assemblies (Fig. 4e), an outcome that arises from the competition between the driving and resisting torques[45]. This configuration promotes the collision-induced merging key to high-aspect-ratio M-Seaweed growth.

Based on the above analyses, … Under high-frequency excitation, the particle-assembled primary structures undergo sustained low-amplitude vibrations, where their inherent structural asymmetries give rise to asymmetric flow fields through the fluid-structure interaction near the substrate[46,47]. The resulting flow asymmetry produces a net lateral driving force that propels the structures into directed translational motion (Fig. 4f(iii) and top view in Supplementary Fig. S17), comparable to the wobbling of nanoeels induced by structural oscillation[48]. This autonomous mobility facilitates collision-induced coalescence between neighboring architectures (Fig. 4f(iv) and Supplementary Fig. S17, S18). …

…

Notably, … As the structure elongates vertically, high-frequency excitation induces progressive nodal formation along its longitudinal axis, driven by a dynamic mismatch between relatively stabilized basal anchoring and inertia-dominated apical oscillations (Fig. 4f(vi)). This behavior resembles the beating motion of magnetically driven artificial microswimmers[49] or elastic structures[50], where internal stress propagation generates bending waves toward the tip. During elongation, its natural frequency decreases until it matches the driving frequency, triggering resonance. This excites higher-order vibration modes that concentrate shear stress (τ_{shear}) at nodal points, where τ_{shear} exceeds the magnetic adhesion strength (τ_{adh}), resulting in localized shear banding and subsequent segmentation into shorter sections[51]. …

The following references have been added to the main text:

45. Gao, Y., Hulsen M.A., Kang T.G., den Toonder J.M.J. Numerical and experimental study of a rotating magnetic particle chain in a viscous fluid. Phys. Rev. E. 86, 041503 (2012).

46. Lauga, E., Powers T.R. The hydrodynamics of swimming microorganisms. Rep. Prog. Phys 72, 096601 (2009).

47. Jang, B. et al. Undulatory locomotion of magnetic multilink nanoswimmers. Nano Lett. 15, 4829-4833 (2015).

48. Mushtaq, F. et al. Motile piezoelectric nanoeels for targeted drug delivery. Adv. Funct. Mater. 29, 1808135 (2019).

49. Dreyfus, R., Baudry J., Roper M.L., Fermigier M., Stone H.A., Bibette J. Microscopic artificial swimmers. Nature 437, 862-865 (2005).

50. Zheng, E., Brandenbourger M., Robinet L., Schall P., Lerner E., Coulais C. Self-oscillation and synchronization transitions in elastoactive structures. Phys. Rev. Lett. 130, 178202 (2023).

51. Cecconi, F., Puglisi A., Viale M., Lucente D. Active-like dynamics of worm-like chains driven by an external traveling-wave force. Soft Matter 21, 5138-5152 (2025).

- Line 445 "As demonstrated in previous studies" – cite the studies

Response: We thank the reviewer for pointing out the need for clarification. The phrase "As demonstrated in previous studies" was intended to refer to the results presented earlier in this manuscript, specifically in Fig. 2 and the related discussion on low-frequency regimes. We have revised the sentence to explicitly cite these internal results to avoid any ambiguity.

Revised manuscript text:

~~As demonstrated in previous studies,~~ As shown in our experiments (Fig. 2e, f), low-frequency regimes produce field-responsive magnetic pillars with constrained height limitations. To enable large-scale structural actuation, we implemented our vertical dynamic growth strategy and subsequently validated the dynamic actuation capabilities of stabilized M-Seaweed structures (Fig. 6h, i and Supplementary Movie 10).

- Line 509, sentence that starts with "This transition…" – back this claim with a citation or a relevant calculation.

Response: We thank the reviewer for raising this point, which has helped us clarify the physical interpretation of the transition toward coherent interfacial transport. In response, we have refined the relevant sentence to more precisely articulate the proposed mechanism and to integrate supporting citations where appropriate.

Revised manuscript text:

Remarkably, when interfacial particle coverage reached a critical threshold, two concurrent phenomena emerged: (1) intact M-Seaweed structures from the lower phase were dragged upward without fragmentation, maintaining their structural integrity, and (2) when these rising structures encountered pre-existing M-Seaweed assemblies at the interface, they directly merged, forming thicker and more robust architectures. ~~This transition was driven by a combination of interfacial tension gradients and magnetic dipolar forces, marking a shift from fragmentation-dominated dynamics toward long-range magnetic coherence and interfacial stress coupling.~~ This transition was facilitated by magnetic dipolar interactions, which, operating within an interface

whose local properties and energy landscape were progressively altered by accumulating particles[55,56], enabled coherent upward motion and merging rather than fragmentation. During this process, over half of the magnetic particles from the lower phase were rapidly transferred (t = 23 s in Fig. 7b(iii) and Fig. 7d), and over time, the vast majority of particles originally dispersed in the PFH phase were transported to the upper aqueous layer, leaving only a residual fraction in the lower phase.

The following references have been added to the main text:

55. Binks, B.P. Particles as surfactants—similarities and differences. Curr. Opin. Colloid. In. 7, 21-41 (2002).

56. Pawar, A.B., Caggioni M., Ergun R., Hartel R.W., Spicer P.T. Arrested coalescence in pickering emulsions. Soft Matter 7, 7710-7716 (2011).

• From line 522 until the end of the results – this entire section needs to be backed by relevant citations.

Response: We sincerely thank the reviewer for the constructive suggestion regarding the need for supporting citations in the section from line 522 to the end of the Results. We fully agree that strengthening the argument with relevant literature is essential scholarly rigor.

In direct response, we have substantially revised this section. Key changes include:

1. Integrating foundational references that support the core physical concepts invoked for each proposed mechanism.

2. Refining the narrative to more clearly articulate the three synergistic properties (namely, sustained actuation, geometric energy focusing, and self-amplifying interfacial recruitment) and their roles in interfacial crossing.

3. Enhancing clarity and flow, ensuring a logical distinction between established principles (now cited) and the novel synergistic integration demonstrated by our system.

We believe that incorporating the suggested citations has provided an opportunity to deepen the scholarly context of our findings. The revised discussion now presents a more rigorously grounded and precise interpretation of the mechanisms, thereby enhancing the overall academic quality of the manuscript. Thank you again for this insightful comment.

Revised manuscript text:

The interfacial crossing capability of the M-Seaweed system can be attributed to three synergistic properties emerging from its dynamic self-assembly: sustained actuation from continuous

growth, efficient energy focusing via high-aspect-ratio geometry, and self-amplifying interfacial recruitment. First, sustained actuation is provided by the continuous, field-driven growth. Unlike static structures, M-Seaweed structures dynamically elongate under the magnetic field. Such out-of-equilibrium assembly process can act as integrated actuators[22,57], whereby continuous growth converts field energy into a sustained mechanical thrust directed against the interface. Concurrently, high-frequency tip oscillations generate localized shear that actively perturbs the interfacial layer, further facilitating penetration. Second, the high-aspect-ratio geometry focuses the actuation energy. This slender form converts magnetic energy predominantly into vertical displacement, concentrating stress at the advancing tip while minimizing lateral dissipation. This directed progress is reminiscent of principles from the hydrodynamics of slender bodies at low Reynolds numbers, where geometric anisotropy favors directional motion[46]. Third, and most distinctively, the system features a self-amplifying, positive-feedback loop at the interface. The initial transfer of particles to the interface alters its local properties. Since adsorbed colloidal particles are known to reduce effective interfacial tension and create energy gradients[55,56], this primary event lowers the local energy barrier for crossing. Consequently, subsequent transfers are progressively facilitated, driving the observed cascade of near-complete particle recruitment from the lower phase (Supplementary Fig. S29). This synergy establishes a new paradigm for designing active systems capable of programmed traversal across immiscible interfaces.

The following references have been added to the main text:

22. Xie, H. et al. Reconfigurable magnetic microrobot swarm: Multimode transformation, locomotion, and manipulation. Sci. Robot. 4, eaav8006 (2019).

46. Lauga, E., Powers T.R. The hydrodynamics of swimming microorganisms. Rep. Prog. Phys 72, 096601 (2009).

55. Binks, B.P. Particles as surfactants—similarities and differences. Curr. Opin. Colloid. In. 7, 21-41 (2002).

56. Pawar, A.B., Caggioni M., Ergun R., Hartel R.W., Spicer P.T. Arrested coalescence in pickering emulsions. Soft Matter 7, 7710-7716 (2011).

57. Xu, T., Hao Z., Huang C., Yu J., Zhang L., Wu X. Multimodal locomotion control of needle-like microrobots assembled by ferromagnetic nanoparticles. IEEE-ASME Trans. Mechatron. 27, 4327-4338 (2022).

Response Letter

Reviewer #1

The revised manuscript is improved. In response to the reviewers' recommendations, the authors have included an overview of the relevant field, further elaborated on their results in the revised manuscript, and offered at least some insight into the underlying observations. As pointed out in my previous report (with corresponding references), similar in nature structures were previously observed in 2 and 3-axial fields, albeit without involving a penetration through an interface. The results, nevertheless, are interesting and contribute to the field of out-of-equilibrium magnetic colloidal assembly. In my opinion, the level of novelty does not meet the standard expected for Nature Communications. The manuscript is better suited for Comm. Materials or Comm. Physics.

Response: We sincerely thank you for acknowledging the improvements in our revised manuscript and for your thoughtful assessment regarding the level of novelty. We have carefully considered your perspective.

We respectfully consider that the novelty and significance of our work lie not merely in the use of multi-axial time-dependent fields, an established principle, but in leveraging this principle to **achieve a previously inaccessible structural and functional capabilities**. Specifically, our work introduces and demonstrates **three integrated features that, to our knowledge, represent a distinct advance beyond prior art in out-of-equilibrium magnetic colloidal assembly:**

1. Gravity-resisting, substrate-originated 3D growth of dynamic, high-aspect-ratio architectures: While prior dynamic assemblies using time-dependent fields have produced remarkable patterns, they are largely confined to 2D landscapes on substrates or 3D aggregates within a bulk phase where gravity is negligible. Our specific field protocol (static vertical + high-frequency horizontal oscillation) enables the **continuous, bottom-up assembly of persistent, segmented "seaweed-like" structures directly from a sedimented particle layer, actively countering gravitational collapse and fluid shear.** This represents a shift from in-plane or suspended assembly to programmable vertical extension from a planar foundation, a capability that is a prerequisite for creating synthetic systems that can bridge distinct phases.

2. Seamless integration of dynamic growth, static stabilization, and re-actuation within a single platform: Beyond growth, our system uniquely provides **programmable control over the operational state** of these high-aspect-ratio structures. Through simple modulation of the oscillating field parameters, we achieve: (i) dynamic growth and reconfiguration (high frequency), (ii) "locking" into a stable static configuration (field off), and (iii) reversible re-actuation into dynamic motion (low frequency). This **unified control over assembly, stabilization, and actuation** in a single system represents a distinct operational integration.

3. Functional execution via active interfacial penetration and validated utility in a compartmentalized system: This point addresses the core of moving from interesting assembly to demonstrable function. We move beyond structural pattern formation to demonstrate active, field-guided traversal of an immiscible liquid-liquid interface coupled with triggered function. By functionalizing particles with an enzyme (HRP), **the growing M-Seaweed acts as a micro-transporter**, delivering catalytic activity across the interface to initiate a localized macroscopic reaction. **Most importantly, we have now validated this functional utility in a model lab-on-a-chip system (new Fig. 8).** In this experiment, the system acts as a **field-gated "enzymatic switch."** Its interfacial penetration delivers HRP, and this catalytic cargo is then transported via microfluidic flow to a physically separate chamber, where it initiates and spatially modulates a colorimetric reaction.

This chip-based experiment concretely closes the loop from structural assembly to embodied functionality. It demonstrates a complete "delivery-and-reaction" sequence in a compartmentalized microsystem, a key step toward intelligent microsystems for applications such as interfacial sensing or controlled reaction engineering. **Using a field-driven dynamic structure to actively gate a biochemical process across an interface and within a networked device,** this capability has not, to our knowledge, been achieved in previous studies of magnetic colloidal assembly.

In summary, we agree that the principle of using time-averaged interactions is established. However, the novelty of our work resides in the specific synergy of field parameters that unlocks the above capabilities, culminating in a demonstrated functional platform for controlled delivery and reaction initiation in microfluidic environments. We believe this integrated advance, which combines a new approach to vertical growth from a substrate with state programmability and validated functional utility in a chip, provides a fuller picture of the work's contribution and helps to convey its significance.

We are grateful for your insightful critique, which prompted us to strengthen our manuscript with the key microfluidic demonstration. We hope this clarification and the new data convincingly articulate the scope and novelty of our contribution.

To further clarify the novelty and contribution of our work, we have undertaken a comprehensive revision to strengthen the focus on the unique growth and assembly process and to present the functional demonstrations in a measured manner. These revisions include:

Title: Revised to "*Gravity-resisting assembly of magnetic seaweed-like structures enabling functional delivery across liquid-liquid interfaces*," positioning the structural assembly as the primary focus, with functional delivery presented as a capability that this unique structure enables.

Abstract and Introduction: Restructured around a unified logical framework that places the unique growth and assembly process at the center, with functional demonstrations presented as capabilities enabled by this structural advance.

Revised Abstract:

Biological systems dynamically grow high-aspect-ratio architectures from a site, enabling traversal of phase boundaries and functional execution. Emulating this growth strategy in synthetic systems could yield functional microsystems for operation across interfaces. However, engineering such bio-inspired growth to proceed out of plane from a substrate in synthetic colloidal assemblies remains challenging, as it requires overcoming gravitational collapse while maintaining structural coherence during extension. Here, we present "Magnetic Seaweed" (M-Seaweed), a field-driven microparticle system that achieves gravity-resisting growth of high-aspect-ratio structures via frequency-modulated magnetic and hydrodynamic interactions. This growth is enabled by combining static and oscillating magnetic fields, which guide the assembly of magnetic particles into dynamic structures exhibiting a distinct segmented, seaweed-like morphology. These architectures are reconfigurable, stabilizable, and programmably actuatable, capable of penetrating a perfluorohexane–water interface. When functionalized with enzymes, the growing M-Seaweed structures act as micro-transporters, delivering catalytic activity across the interface and triggering detectable reactions in both bulk two-phase and microfluidic chip systems. This work establishes a field-driven assembly-to-function approach that integrates structural growth, phase-boundary penetration, and triggered functionality, paving the way for active microsystems capable of interfacial transport and functional execution.

Revised Introduction:

The dynamic growth of high-aspect-ratio structures from a site is a widespread strategy in biology, enabling organisms to traverse phase boundaries and execute critical functions. Pollen tubes extend from germination sites on pollen grains to deliver genetic material through tissues[1]; fungal hyphae elongate from growing tips to secrete enzymes across air gaps onto new substrates[2]; bacterial pili assemble from membrane sites to probe surfaces for adhesion and DNA uptake[3]. Engineering such bio-inspired growth to proceed out of plane from a substrate in synthetic systems would represent a powerful route toward functional microsystems capable of operating across interfaces and within stratified environments.

Field-driven self-assembly of colloidal particles offers a versatile platform to realize such bio-inspired growth. By harnessing external fields (e.g., magnetic[4-7], electric[8-11], acoustic[12-14], optical[15-17], or chemical[18]), researchers can guide components into organized structures. These approaches are broadly classified into two regimes: static self-assembly and dynamic self-assembly[19,20]. The former is governed by equilibrium thermodynamics and yields stable architectures (e.g., crystalline superlattices) but inherently lacks the dynamic responsiveness required for continuous structural evolution. In contrast, the latter operates under non-equilibrium conditions, dissipating energy to form transient, reconfigurable structures capable of

complex behaviors such as collective motion and task execution[20,21]. This paradigm has enabled remarkable advances in microrobotics[22-25], reconfigurable metamaterials[26], adaptive chemical networks[27], and biomedical engineering[28-32].

Despite this progress, dynamic colloidal systems have remained largely confined to two dimensions, forming structures such as in-plane vortices[22,33-35], chains[11,23,36], or ribbon-like assemblies[24,37-39] on planar substrates or at interfaces. This spatial limitation originates from the inherent absence of out-of-plane interactions and the corresponding assembly pathways. Even when three-dimensional (3D) dynamic assemblies are achieved, they typically form within a bulk phase from suspended particles[40-42], rather than through programmable growth upward from a substrate, a process which is essential for creating synthetic systems that can bridge and interact across distinct phases. Consequently, for systems starting with sedimented particles, achieving programmable 3D assembly introduces the fundamental challenge of overcoming gravitational collapse. Recent advances using modulated magnetic fields have enabled controlled vertical assembly[43], yet resulting structures remain compact with limited aspect ratios. Scaling to high-aspect-ratio regimes introduces further complexity: gravitational forces impede continuous vertical elongation, while the increasing aspect ratio itself amplifies mechanical instabilities during dynamic reconfiguration. Together, these factors make the gravity-resisting growth of dynamic, high-aspect-ratio architectures from a substrate remain a challenge in active colloidal matter.

In this work, we report "Magnetic Seaweed" (M-Seaweed): a field-driven microparticle system that achieves gravity-resisting growth of high-aspect-ratio structures and enables functional delivery across liquid-liquid interfaces (Fig. 1). By synergistically combining static and oscillating magnetic fields, the system drives spontaneous growth, ultimately forming segmented seaweed-like architectures in dynamic equilibrium. These architectures are reconfigurable, stabilizable into static configurations, and programmably actuatable through field modulation. Furthermore, they can be steered to penetrate a perfluorohexane (PFH)-water interface, thereby transferring the majority of the constituent microparticles from the substrate into the upper phase. Notably, by decorating particles with the enzyme horseradish peroxidase (HRP), the growing M-Seaweed structures act as micro-transporters that deliver catalytic activity into a target phase containing H_2O_2 and ABTS, triggering a localized macroscopic color change upon penetration. Extending this capability to a microfluidic chip, we further demonstrate that M-Seaweed can serve as a switch for remote reaction initiation in a chip-integrated format. Our work thus establishes a field-driven assembly-to-function platform that integrates structural growth, phase boundary penetration, and functional execution, opening pathways toward intelligent microsystems capable of operating across stratified environments.

Discussion: Reorganized to first establish the structural achievement and its distinction from prior assembly studies, followed by functional demonstrations as examples of what this capability enables.

Revised Discussion:

This work introduces the "Magnetic Seaweed" (M-Seaweed), a system that achieves gravity-resisting growth of high-aspect-ratio structures and enables functional delivery across immiscible liquid-liquid interfaces. By leveraging frequency-modulated magnetic and hydrodynamic interactions, the system guides magnetic microparticles into dynamic, seaweed-like architectures capable of withstanding gravitational collapse and fluid disturbances. While prior systems have utilized time-averaged interactions from multi-axis[58], time-dependent fields to generate complex patterns within a bulk fluid or at an interface, they largely remain confined to 2D organization or to 3D aggregates within a single medium under negligible gravitational influence. These systems generally lack the ability for persistent vertical extension from a substrate. In contrast, M-Seaweed applies the principle of field-programmed time-averaged interactions to enable the bottom-up, persistent assembly of structures directly from a dense particle layer against the influences of gravity and fluid disturbances. Through magneto-hydrodynamic tuning via oscillating field frequency, these interactions simultaneously promote magnetic particle bonding for elongation and confer sufficient structural stiffness to resist buckling, enabling the growth of dynamic, high-aspect-ratio, segmented architectures unattainable in suspension-based or interface-confined systems.

Furthermore, our system establishes a programmable, non-equilibrium assembly pathway that differs from classical field-induced structuring phenomena, such as the Rosensweig instability in ferrofluids[59] or the static-field-driven bottom-up assembly of pillars from sedimented microparticles[44]. Whereas such equilibrium or quasi-equilibrium processes are constrained by static force balances, limiting structures to low aspect ratios, our synergistic use of static and oscillating fields drives continuous, energy-dissipative growth from the substrate. Importantly, this dynamic growth phase is seamlessly integrated with post-assembly functionality. By removing the oscillating field, the resulting structure can be transitioned into a stable, locked configuration (a potential sensor array[60]), and subsequently re-actuated on demand by reapplying a low-frequency oscillation. Thus, within a single experimental setup, the same field modalities (i.e., a static vertical field and a horizontal oscillating field) support three distinct operating regimes through variation of the oscillating field parameters: dynamic growth (high frequency, high amplitude), static stabilization (oscillating field off), and dynamic actuation (low frequency, low amplitude). This unified framework enables a seamless transition from assembly to stabilization to dynamic actuation.

Beyond structural control, the M-Seaweed system accomplishes field-guided penetration across immiscible liquid-liquid interfaces for functional delivery. In a bulk two-phase system, HRP-functionalized particles enable the growing structures to act as micro-transporters, delivering catalytic activity across the PFH-water interface to trigger a localized macroscopic chromogenic reaction. This capability is further extended to a microfluidic chip, where M-Seaweed functions as an enzymatic switch, enabling controlled reaction initiation using TMB as a chromogenic substrate to gate cargo delivery into a separate compartment. These demonstrations collectively

embody functional delivery, amplified macroscopic output, and operation in compartmentalized systems, suggesting potential applications in multiphase reaction engineering, microreactor design, and lab-on-a-chip systems. The strategy of field-guided material delivery across an interface to trigger a localized response provides a conceptual framework that could inspire future designs for functional delivery in more complex environments.

In summary, we have developed a colloidal system that integrates structural assembly, phase-boundary penetration, and functional execution. The M-Seaweed platform thereby advances the field of active matter toward the creation of intelligent microsystems capable of integrating structural growth with functional execution in multiphase environments.

New microfluidic application demonstration: To concretely showcase the system's utility, we have integrated it into a compartmentalized microfluidic chip. This new experiment demonstrates the ability to execute a complete, functional sequence: the field-guided interfacial penetration of the M-Seaweed, followed by the transport of its enzymatic cargo to a separate reaction chamber to initiate a colorimetric assay. This is detailed in the new Fig. 8, Supplementary Fig. 35, Supplementary Movie 15, and the corresponding results section.

Microfluidic integration for controlled enzymatic reactions

To further demonstrate the functional utility of M-Seaweed, we integrated the system into a two-chamber microfluidic chip (Fig. 8a, b and Supplementary Fig. 34). The chip featured a left chamber containing the PFH/aqueous two-phase system with HRP-functionalized particles, a right chamber containing the chromogenic substrate TMB, and a connecting microchannel. This design enabled the investigation of M-Seaweed-mediated enzyme delivery for controlled reactions in a physically separate compartment.

We first conducted a control experiment without applying a magnetic field to prevent M-Seaweed formation or interfacial penetration. Under continuous perfusion at 500 μL/min, the TMB solution in the right chamber gradually lightened due to dilution, with no colorimetric reaction observed (Supplementary Fig. 35a). Quantitative color intensity analysis confirmed this dilution trend in the absence of enzymatic activity (Supplementary Fig. 35b), establishing that no non-specific enzyme transfer occurred.

Next, we examined the full functional sequence under magnetic field guidance, following the timeline illustrated in Fig. 8c. During the initial perfusion period (0-28 s, perfusion on, magnetic field off), M-Seaweed remained inactive, and no color change was detected in the right chamber (Fig. 8d and Supplementary Movie 15). Upon activating the magnetic field (28–45 s, perfusion on, field on), M-Seaweed initiated grow and began penetrating the interface. However, even with ongoing perfusion, no reaction was observed in the right chamber at this early stage (Fig. 8d, 0 s and 34 s). This lack of response is attributed to the limited quantity of HRP delivered during the initial phase of interfacial crossing; only a small number of particles had crossed into the

upper aqueous phase, resulting in enzyme concentrations below the detection threshold upon perfusion.

To allow sufficient enzyme accumulation, we temporarily paused perfusion while maintaining the magnetic field (45–66 s, perfusion off, field on). During this interval, M-Seaweed structures continued to grow and traverse the interface, progressively releasing HRP into the upper aqueous phase of the left chamber. When perfusion was resumed (66 s to 3 min 24 s, perfusion on, field on), a distinct color change emerged in the right chamber. A light blue color first appeared at the chamber bottom (Fig. 8d, 1 min 58 s), signaling the arrival of enzyme-containing solution. Subsequently, a vertical color gradient developed along the channel, marking the spatial progression of the enzymatic reaction (Fig. 8d, 2 min 24 s). As M-Seaweed structures continued to transfer into the upper phase and release enzyme, the color intensity in the right chamber progressively deepened (Fig. 8d, 2 min 57 s). The reaction progression was marked by four distinct stages in the vertical color intensity profile: baseline (no color change), reaction initiation (color change at the bottom), color gradient formation (spatial variation), and gradual darkening (increasing product concentration) (Fig. 8e). These results demonstrate that M-Seaweed acts as an enzymatic switch: its magnetic-field-triggered interfacial penetration gates the delivery of biochemical cargo, and the accumulated enzyme can be transported through microfluidic flow to initiate and modulate reactions in a remote compartment.

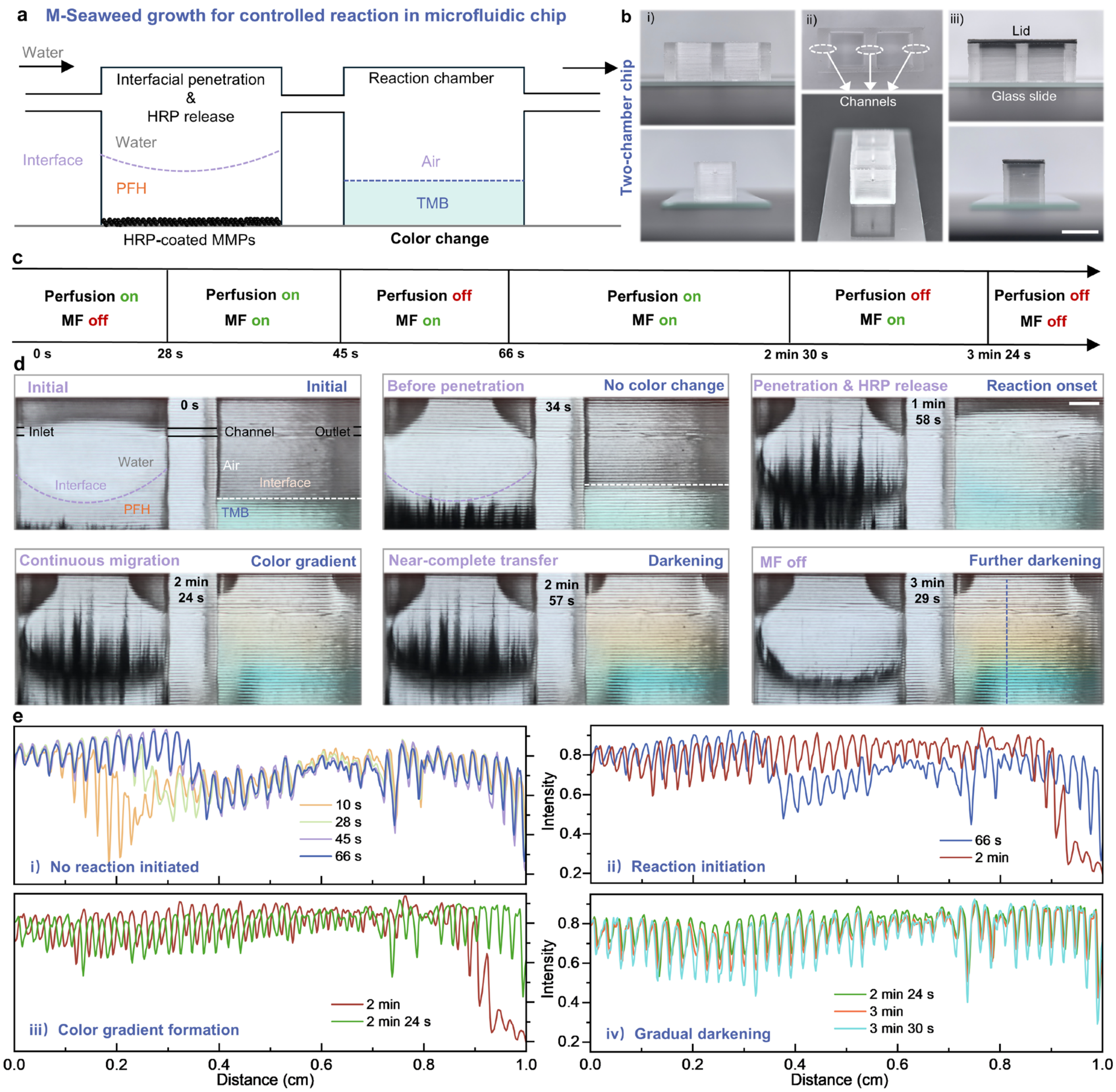

Fig. 8 | Microfluidic integration of M-Seaweed for controlled enzymatic reactions. a Schematic of the two-chamber chip. The left chamber contains the PFH phase with HRP-functionalized magnetic particles and an upper aqueous phase; the right chamber contains the TMB substrate. The chambers are connected by a microchannel. **b** Photograph of the fabricated two-chamber microfluidic chip. Scale bar, 1 cm. **c** Experimental timeline showing the control strategy of perfusion (syringe pump on/off) and magnetic field application (on/off). **d** Time-lapse images showing the reaction progression in the right chamber according to the timeline in (**c**). Before or shortly after M-Seaweed penetration, no reaction occurs in the right chamber upon perfusion. Following successful interfacial penetration, a progressive color change emerges in the right chamber, subsequently forming a vertical gradient (from light green to blue to yellow) and intensifying over time, indicating sustained enzyme delivery and reaction. Scale bar, 2 mm. **e** Vertical color intensity profiles along the dashed line (purple) in (**d**) at selected time points,

capturing four key stages: no reaction initiated, reaction initiation, gradient formation, and gradual darkening.

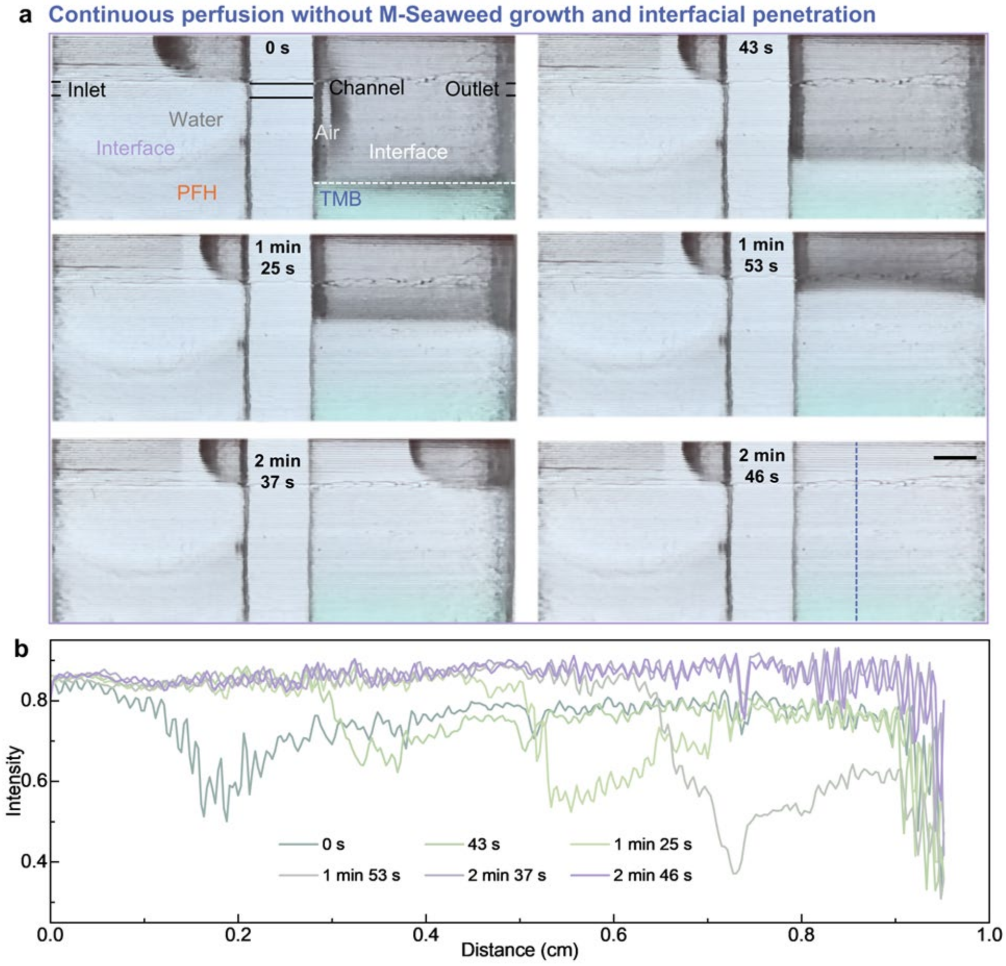

Fig. S35. **Continuous perfusion without M-Seaweed growth and interfacial penetration. a** Time-lapse images of the right chamber containing TMB substrate under continuous perfusion at a flow rate of 500 μL/min, in the absence of M-Seaweed growth and interfacial penetration. The TMB solution gradually lightens over time due to dilution, with no colorimetric reaction observed. **b** Quantitative color intensity profiles along the vertical dashed line (purple) in (**a**) at corresponding time points, showing the progressive dilution of the TMB substrate under continuous perfusion without enzymatic reaction.

We are deeply grateful for your continued engagement and for the thoughtful guidance, which has significantly improved our manuscript. We hope that the revised work now offers a clearer picture of its contribution.

Reviewer #2:

I appreciate that the authors have made revisions and substantially improved the manuscript. In the original version, it was not clearly explained what specific problem the work addresses. The authors have now expanded on their initial study, and several sections have been rewritten to respond to the comments from the first round of review.

Response: We sincerely thank you for your positive feedback.

My main concern is that the revisions have introduced a number of overstated claims, such as "Our work thus establishes a new paradigm for field-driven magnetic materials" or "a field-driven morphogenesis-to-function paradigm." Phrases like "on-demand functional execution" read more like marketing language than scientific description.

Response: Regarding your concern about overstated claims introduced in the revisions, we fully agree with your assessment. In response to your comment, we have carefully reviewed the manuscript and systematically tempered the language to ensure it reflects the scientific content accurately. We have removed or revised phrases such as "establishes a new paradigm," "a field-driven morphogenesis-to-function paradigm," "on-demand …" and similar expressions that could be perceived as marketing language (**see our response to Comment 3 for details**).

Along similar lines, while the method used to form the structures and their potential applications are interesting, it seems overstated to claim that these structures are promising for targeted delivery. In practice, one would first need to deliver the magnetic particles and any associated cargo, and then implement a magnetic field system to achieve interfacial penetration and delivery.

Response: Thank you for your insightful comment regarding the use of "targeted delivery". We appreciate your careful reading and agree that the phrasing could be interpreted as overstating the current capabilities of our system.

As you correctly note, practical implementation would require additional steps, such as the initial delivery of the magnetic particles and cargo, before our interfacial penetration and delivery mechanism could be deployed. We acknowledge that our current demonstration focuses on the interfacial transport and localized triggering aspects of a delivery process, rather than the complete "targeted delivery" pipeline.

To address this concern, we have revised the relevant sentence to more accurately reflect the scope of our work:

Original: Our work establishes a field-driven morphogenesis-to-function paradigm, bridging structural growth, phase-boundary penetration, and triggered function toward intelligent microsystems for targeted delivery and adaptive sensing.

Revised: This work establishes a field-driven assembly-to-function approach that integrates structural growth, phase-boundary penetration, and triggered functionality, paving the way for active microsystems capable of interfacial transport and functional execution.

We believe this revised phrasing more faithfully represents the current state of our work while preserving the scientific interest of the system.

I also find the term "gravity-resisting morphogenesis" misleading. "Morphogenesis" is a well-established term primarily used in developmental biology, and the present work is not related to biological morphogenesis.

I would recommend that the authors:

- Avoid using the term "morphogenesis."
- Tone down the claims about establishing a new paradigm and broad utility.
- Focus instead on what is unique about the growth and assembly process, how it differs from previous assembly studies, and then present the interesting example without exaggeration.

Finally, since the revised manuscript emphasizes assembly across liquid–liquid interfaces and presents a compelling potential application in the conclusions—specifically, a "platform that could be extended to … the tailored delivery of biomolecular cues (e.g., growth factors) across biological membranes for targeted biological modulation"—I recommend that the authors demonstrate such an application, perhaps in a model lab-on-a-chip system. Including such a proof-of-concept experiment would significantly strengthen the paper. As it currently stands, the magnetic assembly is interesting, but the reader is left uncertain about its practical utility.

Response: Thank you for these constructive suggestions. We have revised the terminology, moderated our claims, clarified the uniqueness of the growth and assembly process, and addressed the need for a demonstration of utility in the revised manuscript and our responses below.

1. Avoid using the term "morphogenesis."

Response: Thank you for your careful reading and for raising the point regarding the use of the term "*morphogenesis*." You are absolutely correct that this term carries specific connotations in

developmental biology, and we appreciate your guidance toward more precise scientific language.

In response to your recommendation, we have systematically replaced all instances of "*morphogenesis*" throughout the manuscript with the more accurate term "***assembly***" or "***growth***". We believe this change better reflects the physical nature of our system.

2. Tone down the claims about establishing a new paradigm and broad utility.

Response: We sincerely thank you for the careful and constructive comments on our revised manuscript. Your insights have been invaluable in helping us improve the clarity and scientific rigor of our work. We particularly appreciate your observation regarding overstated claims, and we have carefully revised the manuscript accordingly.

In response to your suggestion to "tone down the claims about establishing a new paradigm and broad utility," we have systematically modified the abstract, introduction, and discussion to adopt more measured and scientifically precise language (**see our response to Comment 3 for details**).

We believe these revisions have appropriately tempered the claims while preserving the core scientific message of our work. The manuscript now presents our findings in a more measured and academically rigorous tone, in accordance with your valuable suggestion.

3. Focus instead on what is unique about the growth and assembly process, how it differs from previous assembly studies, and then present the interesting example without exaggeration.

Response: We sincerely thank you for this insightful guidance. The suggestion to "focus instead on what is unique about the growth and assembly process, how it differs from previous assembly studies, and then present the interesting example without exaggeration" has been instrumental in reshaping the core narrative of our manuscript.

In response, we have undertaken a comprehensive revision to focus the narrative on the unique assembly of M-Seaweed structures, with functional demonstrations presented as capabilities enabled by this structural advance. The key modifications are as follows:

1. Title: We have subtly but significantly modified the title to reflect this refocused emphasis. The original title "*for functional delivery*" has been revised to "*enabling functional delivery*." This single word change shifts the framing: *the gravity-resisting assembly of magnetic seaweed-like structures* is now positioned as the primary scientific focus, with functional delivery presented as a capability that this unique structure enables. The revised title reads: "***Gravity-resisting assembly of magnetic seaweed-like structures enabling functional delivery across liquid-liquid interfaces***."

2. Abstract and Introduction: Both sections have been restructured around a unified logical framework that now places the unique growth and assembly process at the center. The narrative now flows as follows: biological systems dynamically grow high-aspect-ratio architectures from a site, enabling traversal of phase boundaries and functional execution; emulating this growth strategy in synthetic systems could yield functional microsystems for operation across interfaces; however, engineering such bio-inspired growth to proceed out of plane from a substrate in synthetic colloidal assemblies remains challenging, as it requires overcoming gravitational collapse while maintaining structural coherence during extension; here, we introduce M-Seaweed as a system that achieves this gravity-resisting growth through frequency-modulated magnetic and hydrodynamic interactions. This reframing ensures that the structural achievement serves as the central contribution, with functional demonstrations (the ABTS-based macroscopic reaction and the **newly added microfluidic chip experiment—see Comment 4**) presented as capabilities enabled by this structural advance.

Revised Abstract:

Biological systems dynamically grow high-aspect-ratio architectures from a site, enabling traversal of phase boundaries and functional execution. Emulating this growth strategy in synthetic systems could yield functional microsystems for operation across interfaces. However, engineering such bio-inspired growth to proceed out of plane from a substrate in synthetic colloidal assemblies remains challenging, as it requires overcoming gravitational collapse while maintaining structural coherence during extension. Here, we present "Magnetic Seaweed" (M-Seaweed), a field-driven microparticle system that achieves gravity-resisting growth of high-aspect-ratio structures via frequency-modulated magnetic and hydrodynamic interactions. This growth is enabled by combining static and oscillating magnetic fields, which guide the assembly of magnetic particles into dynamic structures exhibiting a distinct segmented, seaweed-like morphology. These architectures are reconfigurable, stabilizable, and programmably actuatable, capable of penetrating a perfluorohexane–water interface. When functionalized with enzymes, the growing M-Seaweed structures act as micro-transporters, delivering catalytic activity across the interface and triggering detectable reactions in both bulk two-phase and microfluidic chip systems. This work establishes a field-driven assembly-to-function approach that integrates structural growth, phase-boundary penetration, and triggered functionality, paving the way for active microsystems capable of interfacial transport and functional execution.

Revised Introduction:

The dynamic growth of high-aspect-ratio structures from a site is a widespread strategy in biology, enabling organisms to traverse phase boundaries and execute critical functions. Pollen tubes extend from germination sites on pollen grains to deliver genetic material through tissues[1]; fungal hyphae elongate from growing tips to secrete enzymes across air gaps onto new substrates[2]; bacterial pili assemble from membrane sites to probe surfaces for adhesion and DNA uptake[3]. Engineering such bio-inspired growth to proceed out of plane from a substrate in

synthetic systems would represent a powerful route toward functional microsystems capable of operating across interfaces and within stratified environments.

Field-driven self-assembly of colloidal particles offers a versatile platform to realize such bio-inspired growth. By harnessing external fields (e.g., magnetic[4-7], electric[8-11], acoustic[12-14], optical[15-17], or chemical[18]), researchers can guide components into organized structures. These approaches are broadly classified into two regimes: static self-assembly and dynamic self-assembly[19,20]. The former is governed by equilibrium thermodynamics and yields stable architectures (e.g., crystalline superlattices) but inherently lacks the dynamic responsiveness required for continuous structural evolution. In contrast, the latter operates under non-equilibrium conditions, dissipating energy to form transient, reconfigurable structures capable of complex behaviors such as collective motion and task execution[20,21]. This paradigm has enabled remarkable advances in microrobotics[22-25], reconfigurable metamaterials[26], adaptive chemical networks[27], and biomedical engineering[28-32].

Despite this progress, dynamic colloidal systems have remained largely confined to two dimensions, forming structures such as in-plane vortices[22,33-35], chains[11,23,36], or ribbon-like assemblies[24,37-39] on planar substrates or at interfaces. This spatial limitation originates from the inherent absence of out-of-plane interactions and the corresponding assembly pathways. Even when three-dimensional (3D) dynamic assemblies are achieved, they typically form within a bulk phase from suspended particles[40-42], rather than through programmable growth upward from a substrate, a process which is essential for creating synthetic systems that can bridge and interact across distinct phases. Consequently, for systems starting with sedimented particles, achieving programmable 3D assembly introduces the fundamental challenge of overcoming gravitational collapse. Recent advances using modulated magnetic fields have enabled controlled vertical assembly[43], yet resulting structures remain compact with limited aspect ratios. Scaling to high-aspect-ratio regimes introduces further complexity: gravitational forces impede continuous vertical elongation, while the increasing aspect ratio itself amplifies mechanical instabilities during dynamic reconfiguration. Together, these factors make the gravity-resisting growth of dynamic, high-aspect-ratio architectures from a substrate remain a challenge in active colloidal matter.

In this work, we report "Magnetic Seaweed" (M-Seaweed): a field-driven microparticle system that achieves gravity-resisting growth of high-aspect-ratio structures and enables functional delivery across liquid-liquid interfaces (Fig. 1). By synergistically combining static and oscillating magnetic fields, the system drives spontaneous growth, ultimately forming segmented seaweed-like architectures in dynamic equilibrium. These architectures are reconfigurable, stabilizable into static configurations, and programmably actuatable through field modulation. Furthermore, they can be steered to penetrate a perfluorohexane (PFH)-water interface, thereby transferring the majority of the constituent microparticles from the substrate into the upper phase. Notably, by decorating particles with the enzyme horseradish peroxidase (HRP), the growing M-Seaweed structures act as micro-transporters that deliver catalytic activity into a target phase

containing H_2O_2 and ABTS, triggering a localized macroscopic color change upon penetration. Extending this capability to a microfluidic chip, we further demonstrate that M-Seaweed can serve as a switch for remote reaction initiation in a chip-integrated format. Our work thus establishes a field-driven assembly-to-function platform that integrates structural growth, phase boundary penetration, and functional execution, opening pathways toward intelligent microsystems capable of operating across stratified environments.

3. New functional demonstration (the "interesting example"): In response to your suggestion regarding lab-on-a-chip demonstration, we added a new experiment integrating M-Seaweed into a two-chamber microfluidic chip. This experiment demonstrates controlled enzymatic reaction initiation in a separate compartment (Fig. 8 and Supplementary Movie 15, **see Comment 4 for more detail**). We present this as an extension of the structural capability rather than an overreaching claim, thereby adhering to your advice to "present the interesting example without exaggeration."

4. Discussion: The Discussion has been reorganized to first establish the structural achievement (i.e., gravity-resisting growth of high-aspect-ratio, segmented architectures) and its distinction from prior assembly studies (e.g., Rosensweig instability, static-field-driven pillar assemblies). The functional demonstrations are then presented as examples of what this unique structural capability enables, without exaggeration.

Revised Discussion:

This work introduces the "Magnetic Seaweed" (M-Seaweed), a system that achieves gravity-resisting growth of high-aspect-ratio structures and enables functional delivery across immiscible liquid-liquid interfaces. By leveraging frequency-modulated magnetic and hydrodynamic interactions, the system guides magnetic microparticles into dynamic, seaweed-like architectures capable of withstanding gravitational collapse and fluid disturbances. While prior systems have utilized time-averaged interactions from multi-axis[58], time-dependent fields to generate complex patterns within a bulk fluid or at an interface, they largely remain confined to 2D organization or to 3D aggregates within a single medium under negligible gravitational influence. These systems generally lack the ability for persistent vertical extension from a substrate. In contrast, M-Seaweed applies the principle of field-programmed time-averaged interactions to enable the bottom-up, persistent assembly of structures directly from a dense particle layer against the influences of gravity and fluid disturbances. Through magneto-hydrodynamic tuning via oscillating field frequency, these interactions simultaneously promote magnetic particle bonding for elongation and confer sufficient structural stiffness to resist buckling, enabling the growth of dynamic, high-aspect-ratio, segmented architectures unattainable in suspension-based or interface-confined systems.

Furthermore, our system establishes a programmable, non-equilibrium assembly pathway that differs from classical field-induced structuring phenomena, such as the Rosensweig instability in ferrofluids[59] or the static-field-driven bottom-up assembly of pillars from sedimented

microparticles[44]. Whereas such equilibrium or quasi-equilibrium processes are constrained by static force balances, limiting structures to low aspect ratios, our synergistic use of static and oscillating fields drives continuous, energy-dissipative growth from the substrate. Importantly, this dynamic growth phase is seamlessly integrated with post-assembly functionality. By removing the oscillating field, the resulting structure can be transitioned into a stable, locked configuration (a potential sensor array[60]), and subsequently re-actuated on demand by reapplying a low-frequency oscillation. Thus, within a single experimental setup, the same field modalities (i.e., a static vertical field and a horizontal oscillating field) support three distinct operating regimes through variation of the oscillating field parameters: dynamic growth (high frequency, high amplitude), static stabilization (oscillating field off), and dynamic actuation (low frequency, low amplitude). This unified framework enables a seamless transition from assembly to stabilization to dynamic actuation.

Beyond structural control, the M-Seaweed system accomplishes field-guided penetration across immiscible liquid-liquid interfaces for functional delivery. In a bulk two-phase system, HRP-functionalized particles enable the growing structures to act as micro-transporters, delivering catalytic activity across the PFH-water interface to trigger a localized macroscopic chromogenic reaction. This capability is further extended to a microfluidic chip, where M-Seaweed functions as an enzymatic switch, enabling controlled reaction initiation using TMB as a chromogenic substrate to gate cargo delivery into a separate compartment. These demonstrations collectively embody functional delivery, amplified macroscopic output, and operation in compartmentalized systems, suggesting potential applications in multiphase reaction engineering, microreactor design, and lab-on-a-chip systems. The strategy of field-guided material delivery across an interface to trigger a localized response provides a conceptual framework that could inspire future designs for functional delivery in more complex environments.

In summary, we have developed a colloidal system that integrates structural assembly, phase-boundary penetration, and functional execution. The M-Seaweed platform thereby advances the field of active matter toward the creation of intelligent microsystems capable of integrating structural growth with functional execution in multiphase environments.

We hope that our revisions have adequately addressed the valuable points raised in your guidance. We believe the manuscript now offers a clearer and more coherent narrative that focuses on the unique growth and assembly process, while the functional studies hopefully demonstrate the potential of this new class of structures in a balanced manner.

Thank you again for this invaluable perspective, which has significantly strengthened the clarity and scientific rigor of our work. We appreciate your continued guidance in refining the precision and tone of our work. We hope that the revised manuscript now meets the standards of scientific rigor.

4. Finally, since the revised manuscript emphasizes assembly across liquid–liquid interfaces and presents a compelling potential application in the conclusions—specifically, a "platform that could be extended to … the tailored delivery of biomolecular cues (e.g., growth factors) across biological membranes for targeted biological modulation"—I recommend that the authors demonstrate such an application, perhaps in a model lab-on-a-chip system. Including such a proof-of-concept experiment would significantly strengthen the paper. As it currently stands, the magnetic assembly is interesting, but the reader is left uncertain about its practical utility.

Response: We sincerely appreciate your thoughtful suggestion regarding the potential application of our system. We fully agree that demonstrating the practical utility of our platform in a relevant context is essential and would strengthen the manuscript, and we appreciate your specific recommendation of a lab-on-a-chip system.

In response, initially, we attempted to use a phospholipid monolayer assembled at the PFH-water interface to mimic a biological membrane. However, we encountered unexpected difficulties: the M-Seaweed structures became trapped by the adhesive nature of the lipid layer, preventing successful traversal. This highlights the complex interplay between dynamic structures and soft interfacial layers, which we believe warrants a separate investigation beyond the scope of the current work.

Nevertheless, to provide a concrete demonstration of the system's potential utility, we have designed and executed an alternative experiment that directly integrates the M-Seaweed system into a microfluidic chip. This new data is presented as **Fig. 8** (**Supplementary Movie 15; Supplementary Figs. 34** and **35**) in the revised manuscript.

In this experiment, we constructed a two-chamber microfluidic device connected by a microchannel (**Fig. 8a, b**). The left chamber is based on our established bi-phase system (PFH bottom phase with HRP-coated particles, and an upper aqueous phase). The right chamber contains a solution of TMB, a sensitive chromogenic substrate. The experimental design allows us to test whether the enzyme delivery by M-Seaweed can be harnessed for controlled reactions in a separate compartment:

Control condition: When the M-Seaweed in the left chamber has not yet penetrated the interface, activating the syringe pump to flow the upper aqueous phase into the right chamber results only in slight dilution of the TMB substrate, with no detectable colorimetric reaction. This confirms that without interfacial penetration, no functional cargo is delivered (**Fig. S35**).

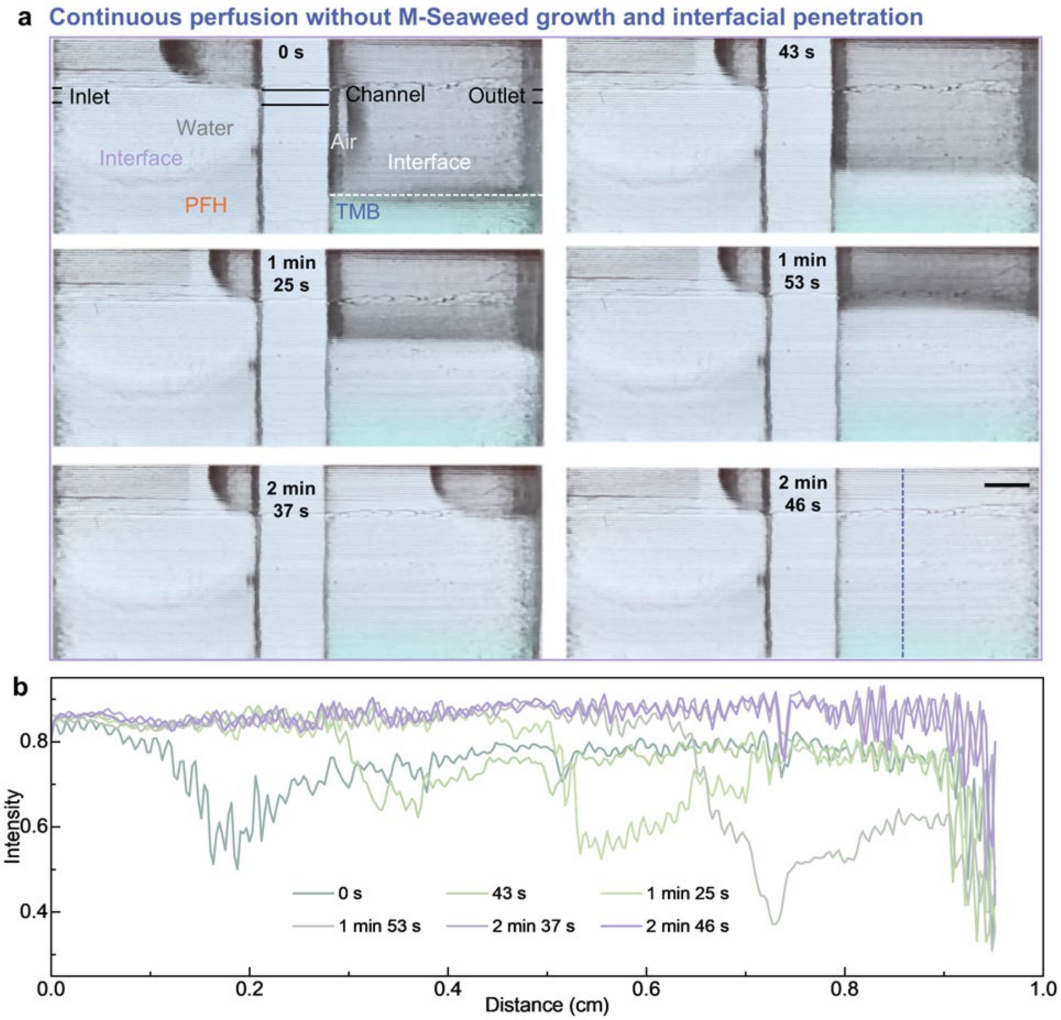

Fig. S35. **Continuous perfusion without M-Seaweed growth and interfacial penetration. a** Time-lapse images of the right chamber containing TMB substrate under continuous perfusion at a flow rate of 500 μL/min, in the absence of M-Seaweed growth and interfacial penetration. The TMB solution gradually lightens over time due to dilution, with no colorimetric reaction observed. **b** Quantitative color intensity profiles along the vertical dashed line (purple) in (**a**) at corresponding time points, showing the progressive dilution of the TMB substrate under continuous perfusion without enzymatic reaction.

Functional condition: After the M-Seaweed successfully penetrates the interface and delivers HRP into the upper aqueous phase, activating the syringe pump now causes a distinct and progressive color change in the right chamber. A striking color gradient (from light green to blue to yellow) develops over time, demonstrating that the enzyme delivered by the M-Seaweed has been transported through the microchannel to catalyze a reaction with the TMB substrate. The reaction intensity increases over time, reflecting continuous enzyme delivery (**Fig. 8d, e and Supplementary Movie 15**).

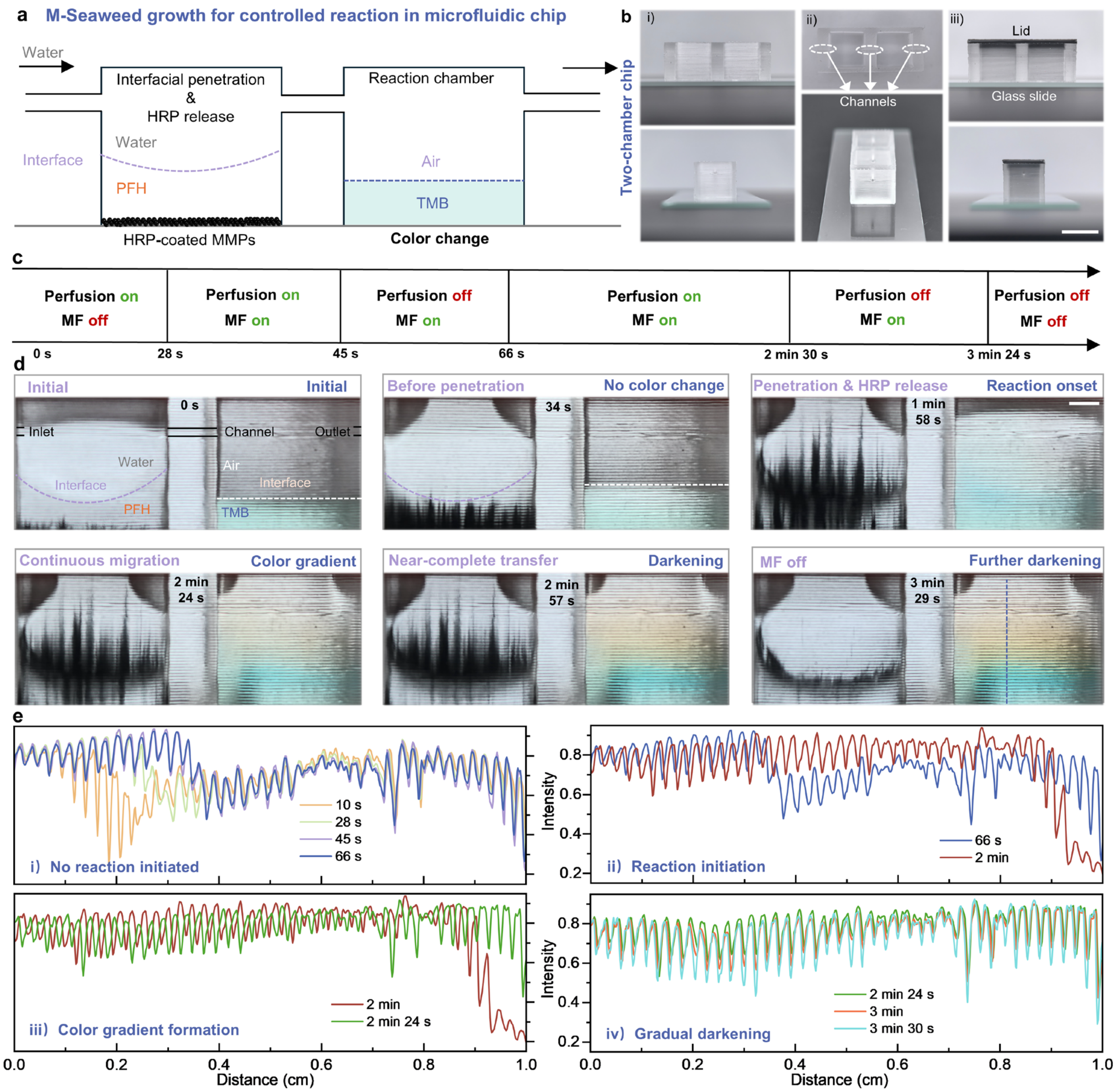

Fig. 8 | Microfluidic integration of M-Seaweed for controlled enzymatic reactions. a Schematic of the two-chamber chip. The left chamber contains the PFH phase with HRP-functionalized magnetic particles and an upper aqueous phase; the right chamber contains the TMB substrate. The chambers are connected by a microchannel. **b** Photograph of the fabricated two-chamber microfluidic chip. Scale bar, 1 cm. **c** Experimental timeline showing the control strategy of perfusion (syringe pump on/off) and magnetic field application (on/off). **d** Time-lapse images showing the reaction progression in the right chamber according to the timeline in (**c**). Before or shortly after M-Seaweed penetration, no reaction occurs in the right chamber upon perfusion. Following successful interfacial penetration, a progressive color change emerges in the right chamber, subsequently forming a vertical gradient (from light green to blue to yellow) and intensifying over time, indicating sustained enzyme delivery and reaction. Scale bar, 2 mm. **e** Vertical color intensity profiles along the dashed line (purple) in (**d**) at selected time points,

capturing four key stages: no reaction initiated, reaction initiation, gradient formation, and gradual darkening.

This experiment directly demonstrates, within a relevant lab-on-a-chip platform, that the M-Seaweed can function as an "enzymatic switch" for initiating chemical reactions in a separate, controlled compartment. It shows a clear and tangible application: controlled delivery of biochemical cargo to trigger reactions in microfluidic networks.

Regarding the broader biological application mentioned in our original Discussion, we believe that a direct demonstration across biological membranes lies beyond the scope of the current study. We have therefore revised the relevant sentence in the Discussion to more accurately reflect this boundary: "*The strategy of field-guided material delivery across an interface to trigger a localized response provides a conceptual framework that could inspire future designs for functional delivery in more complex environments.*"

We believe this revision appropriately tempers the claim while preserving the forward-looking nature of the discussion, and together with the new microfluidic experiment, significantly strengthens the manuscript by providing a clear, experimentally grounded demonstration of the platform's capabilities.

We thank you again for your insightful guidance, which has significantly enhanced the practical relevance and scientific rigor of our work.

The following changes have been made in our revised manuscript:

1. Fig. 8, Supplementary Fig. 35, Supplementary Movie 15, and related results section have been added in the main text:

Microfluidic integration for controlled enzymatic reactions

To further demonstrate the functional utility of M-Seaweed, we integrated the system into a two-chamber microfluidic chip (Fig. 8a, b and Supplementary Fig. 34). The chip featured a left chamber containing the PFH/aqueous two-phase system with HRP-functionalized particles, a right chamber containing the chromogenic substrate TMB, and a connecting microchannel. This design enabled the investigation of M-Seaweed-mediated enzyme delivery for controlled reactions in a physically separate compartment.

We first conducted a control experiment without applying a magnetic field to prevent M-Seaweed formation or interfacial penetration. Under continuous perfusion at 500 μL/min, the TMB solution in the right chamber gradually lightened due to dilution, with no colorimetric reaction observed (Supplementary Fig. 35a). Quantitative color intensity analysis confirmed this dilution trend in the absence of enzymatic activity (Supplementary Fig. 35b), establishing that no non-specific enzyme transfer occurred.

Next, we examined the full functional sequence under magnetic field guidance, following the timeline illustrated in Fig. 8c. During the initial perfusion period (0-28 s, perfusion on, magnetic field off), M-Seaweed remained inactive, and no color change was detected in the right chamber (Fig. 8d and Supplementary Movie 15). Upon activating the magnetic field (28–45 s, perfusion on, field on), M-Seaweed initiated grow and began penetrating the interface. However, even with ongoing perfusion, no reaction was observed in the right chamber at this early stage (Fig. 8d, 0 s and 34 s). This lack of response is attributed to the limited quantity of HRP delivered during the initial phase of interfacial crossing; only a small number of particles had crossed into the upper aqueous phase, resulting in enzyme concentrations below the detection threshold upon perfusion.

To allow sufficient enzyme accumulation, we temporarily paused perfusion while maintaining the magnetic field (45–66 s, perfusion off, field on). During this interval, M-Seaweed structures continued to grow and traverse the interface, progressively releasing HRP into the upper aqueous phase of the left chamber. When perfusion was resumed (66 s to 3 min 24 s, perfusion on, field on), a distinct color change emerged in the right chamber. A light blue color first appeared at the chamber bottom (Fig. 8d, 1 min 58 s), signaling the arrival of enzyme-containing solution. Subsequently, a vertical color gradient developed along the channel, marking the spatial progression of the enzymatic reaction (Fig. 8d, 2 min 24 s). As M-Seaweed structures continued to transfer into the upper phase and release enzyme, the color intensity in the right chamber progressively deepened (Fig. 8d, 2 min 57 s). The reaction progression was marked by four distinct stages in the vertical color intensity profile: baseline (no color change), reaction initiation (color change at the bottom), color gradient formation (spatial variation), and gradual darkening (increasing product concentration) (Fig. 8e). These results demonstrate that M-Seaweed acts as an enzymatic switch: its magnetic-field-triggered interfacial penetration gates the delivery of biochemical cargo, and the accumulated enzyme can be transported through microfluidic flow to initiate and modulate reactions in a remote compartment.

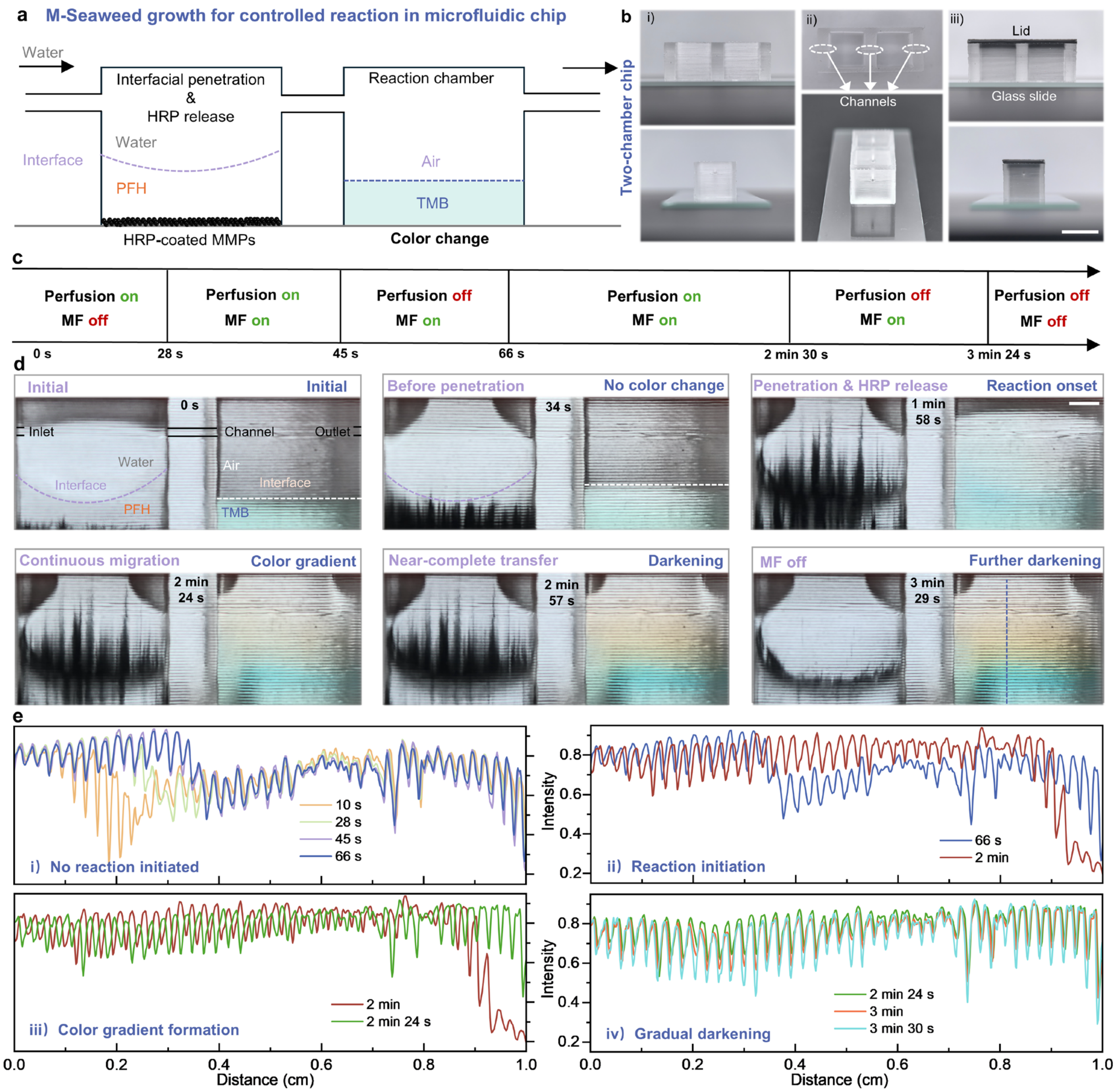

Fig. 8 | Microfluidic integration of M-Seaweed for controlled enzymatic reactions. a Schematic of the two-chamber chip. The left chamber contains the PFH phase with HRP-functionalized magnetic particles and an upper aqueous phase; the right chamber contains the TMB substrate. The chambers are connected by a microchannel. **b** Photograph of the fabricated two-chamber microfluidic chip. Scale bar, 1 cm. **c** Experimental timeline showing the control strategy of perfusion (syringe pump on/off) and magnetic field application (on/off). **d** Time-lapse images showing the reaction progression in the right chamber according to the timeline in (**c**). Before or shortly after M-Seaweed penetration, no reaction occurs in the right chamber upon perfusion. Following successful interfacial penetration, a progressive color change emerges in the right chamber, subsequently forming a vertical gradient (from light green to blue to yellow) and intensifying over time, indicating sustained enzyme delivery and reaction. Scale bar, 2 mm. **e** Vertical color intensity profiles along the dashed line (purple) in (**d**) at selected time points,

capturing four key stages: no reaction initiated, reaction initiation, gradient formation, and gradual darkening.

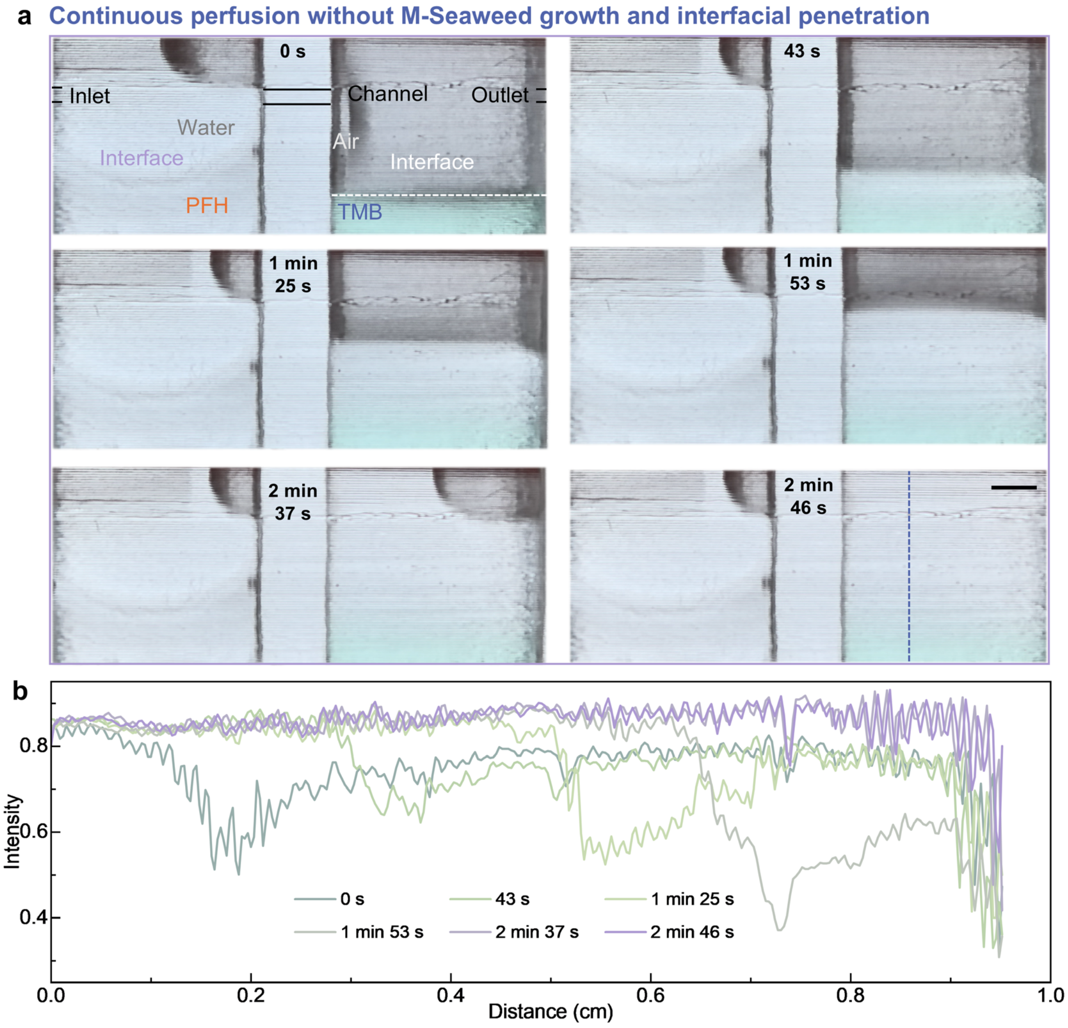

Fig. S35. **Continuous perfusion without M-Seaweed growth and interfacial penetration. a** Time-lapse images of the right chamber containing TMB substrate under continuous perfusion at a flow rate of 500 μL/min, in the absence of M-Seaweed growth and interfacial penetration. The TMB solution gradually lightens over time due to dilution, with no colorimetric reaction observed. **b** Quantitative color intensity profiles along the vertical dashed line (purple) in (**a**) at corresponding time points, showing the progressive dilution of the TMB substrate under continuous perfusion without enzymatic reaction.

2. The experimental methods for the newly added studies have been incorporated into the main text:

Fabrication of the two-chamber chip

The two-chamber microfluidic chip was fabricated using standard soft lithography techniques (Supplementary Fig. 34). A master mold with channel features (channel diameter: 0.8 mm) was designed using computer-aided design software and fabricated by 3D printing. To create inlet/outlet channels, stainless steel needles were inserted into the mold prior to Polydimethylsiloxane (PDMS) casting. PDMS prepolymer mixed with a curing agent (10:1 ratio) was degassed, poured onto the mold, and cured at 60°C for 3 hours. After curing, the needles were removed to form through-hole channels, and the PDMS replica was peeled off from the mold. The PDMS slab and a glass slide were treated with oxygen plasma and bonded together to form an irreversible seal. Finally, a 3D-printed lid was placed onto the PDMS slab to create a relatively enclosed two-chamber configuration for fluidic experiments, with the more sensitive chromogenic substrate TMB (3,3',5,5'-Tetramethylbenzidine) injected into the right chamber.

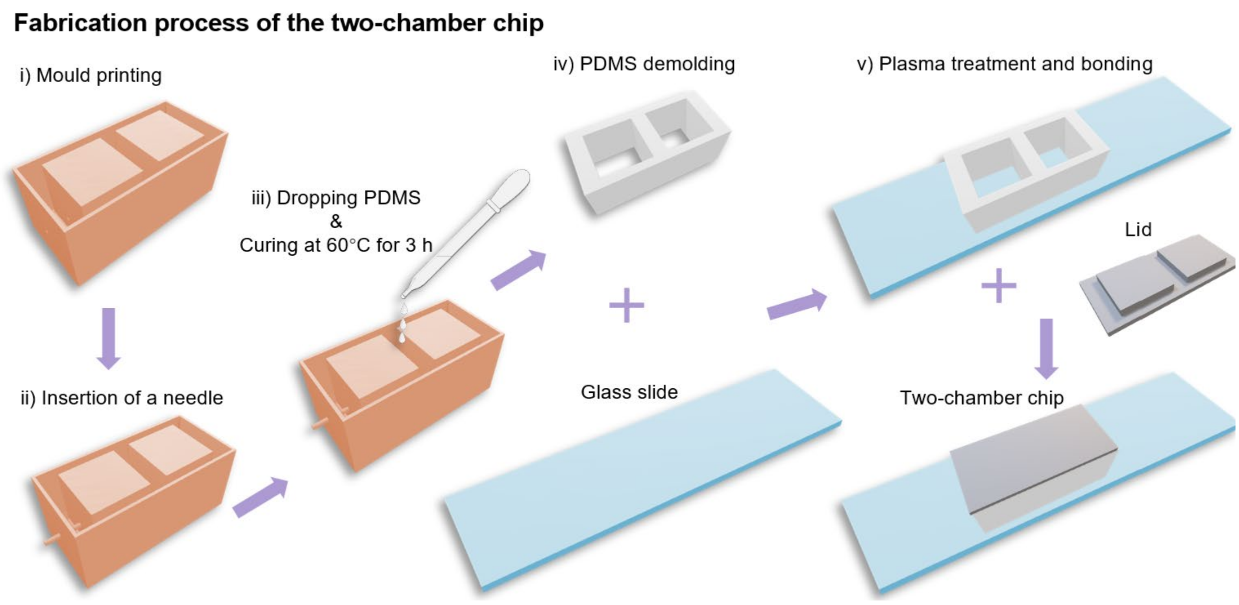

Fig. S34. Schematic illustration of the fabrication process for the two-chamber microfluidic chip. A master mold with channels (0.8 mm diameter) was designed and 3D-printed. Stainless steel needles were inserted into the mold to form inlet/outlet channels prior to PDMS casting. After PDMS pouring and curing at 60°C for 3 hours, the PDMS replica was peeled off and bonded to a glass slide via oxygen plasma treatment. A 3D-printed lid was then assembled onto the PDMS slab to form the final enclosed two-chamber chip.

3. Optimization of the discussion regarding biological membrane-related statements:

The Discussion has been revised to temper the speculative claim regarding delivery across biological membranes. The original phrasing has been replaced with a more conservative and experimentally grounded forward-looking statement as follows:

Beyond structural control, the M-Seaweed system accomplishes field-guided penetration across immiscible liquid-liquid interfaces for functional delivery. In a bulk two-phase system, HRP-

functionalized particles enable the growing structures to act as micro-transporters, delivering catalytic activity across the PFH-water interface to trigger a localized macroscopic chromogenic reaction. This capability is further extended to a microfluidic chip, where M-Seaweed functions as an enzymatic switch, enabling controlled reaction initiation using TMB as a chromogenic substrate to gate cargo delivery into a separate compartment. These demonstrations collectively embody functional delivery, amplified macroscopic output, and operation in compartmentalized systems, suggesting potential applications in multiphase reaction engineering, microreactor design, and lab-on-a-chip systems. *The strategy of field-guided material delivery across an interface to trigger a localized response provides a conceptual framework that could inspire future designs for functional delivery in more complex environments.*

Response Letter

Reviewer #1

The authors have further improved the manuscript following the latest revision. With the added experiments and data, the paper now meets the bar for publication in Nature Communications.

Response: We are sincerely grateful for your rigorous and insightful evaluation throughout the review process. Your deep expertise in field-driven dynamic assembly was evident in every round of comments, and your detailed suggestions significantly strengthened the physical depth of this work. We also appreciate your constructive criticism of the overly descriptive style, which prompted us to provide more intuitive physical insights in the main text. We are truly grateful for the time and expertise you devoted to reviewing our work.

Reviewer #2:

The authors have revised their manuscript. I am grateful that the authors have toned down the hyperbole that accompanied several overstated claims in the previous version of their manuscript. The title has also been adapted and the confusing terminology concerning morphogenesis has been removed. This is a major improvement. The authors have also taken my suggestion to heart to demonstrate that the magnetic structures can indeed deliver something 'useful' across an interface. Originally, the paper contained claims of growth factors in biological systems and/or delivery in the context of therapeutics or "targeted biological modulation". The demonstrated example falls short of the originally claimed ability of M-seaweed to modulate biological function. I still do not really see that the method will be useful for biology, given its complexity and the magnetic colloidal structures that are most likely not desirable in most biological experiments. Nevertheless, I commend the authors for including the demonstration and for undertaking a string of experiments that are now presented in a new section of the paper. The authors have in their latest revision shown that their method permits the delivery of an enzyme across an interface. This demonstration is a valuable addition to the paper. I have no further comments and I think it is now time for a wider audience to judge the merits of the presented method.

Response: We are deeply grateful for your rigorous and thoughtful evaluation throughout the review process. Your guidance on tempering overstated claims, adopting a more literal physical terminology, and demonstrating the utility led to a fundamental strengthening of the paper. The experimental demonstration of enzyme delivery, in particular, would not have been included without your suggestion, and we believe it makes the work substantially more convincing and broadly relevant. We also appreciate your frank assessment of the current limitations for direct biological application; your perspective helped us frame our contributions with appropriate

scientific restraint. We thank you wholeheartedly for your invaluable contribution in shaping the final manuscript.
